# Hurricane Harvey Impacts on Water Quality and Microbial Communities in Houston, TX Waterbodies

**DOI:** 10.3389/fmicb.2022.875234

**Published:** 2022-06-14

**Authors:** Michael G. LaMontagne, Yan Zhang, George J. Guillen, Terry J. Gentry, Michael S. Allen

**Affiliations:** ^1^Department of Biology and Biotechnology, University of Houston – Clear Lake, Houston, TX, United States; ^2^Department of Microbiology, Immunology and Genetics, University of North Texas Health Science Center, Fort Worth, TX, United States; ^3^Department of Soil and Crop Sciences, Texas A&M University, College Station, TX, United States

**Keywords:** tropical storms, metagenomic, nutrient, fecal indicator bacteria, PICRUSt (phylogenetic investigation of communities by reconstruction of unobserved states), antibiotic resistant bacteria (ARB), NMDS

## Abstract

Extreme weather events can temporarily alter the structure of coastal systems and generate floodwaters that are contaminated with fecal indicator bacteria (FIB); however, every coastal system is unique, so identification of trends and commonalities in these episodic events is challenging. To improve our understanding of the resilience of coastal systems to the disturbance of extreme weather events, we monitored water quality, FIB at three stations within Clear Lake, an estuary between Houston and Galveston, and three stations in bayous that feed into the estuary. Water samples were collected immediately before and after Hurricane Harvey (HH) and then throughout the fall of 2017. FIB levels were monitored by culturing *E. coli* and *Enterococci.* Microbial community structure was profiled by high throughput sequencing of PCR-amplified 16S rRNA gene fragments. Water quality and FIB data were also compared to historical data for these water body segments. Before HH, salinity within Clear Lake ranged from 9 to 11 practical salinity units (PSU). Immediately after the storm, salinity dropped to < 1 PSU and then gradually increased to historical levels over 2 months. Dissolved inorganic nutrient levels were also relatively low immediately after HH and returned, within a couple of months, to historical levels. FIB levels were elevated immediately after the storm; however, after 1 week, *E. coli* levels had decreased to what would be acceptable levels for freshwater. *Enterococci* levels collected several weeks after the storm were within the range of historical levels. Microbial community structure shifted from a system dominated by *Cyanobacteria* sp. before HH to a system dominated by *Proteobacteria* and *Bacteroidetes* immediately after. Several sequences observed only in floodwater showed similarity to sequences previously reported for samples collected following Hurricane Irene. These changes in beta diversity corresponded to salinity and nitrate/nitrite concentrations. Differential abundance analysis of metabolic pathways, predicted from 16S sequences, suggested that pathways associated with virulence and antibiotic resistance were elevated in floodwater. Overall, these results suggest that floodwater generated from these extreme events may have high levels of fecal contamination, antibiotic resistant bacteria and bacteria rarely observed in other systems.

## Introduction

Hurricane Harvey deluged the Houston metropolitan area in August of 2017 with over a meter of rain in less than 48 h. This rainfall set a record for the continental United States (Cappucci, 2017), and exposed thousands, perhaps millions, of citizens and first responders to potentially contaminated floodwaters. In rural regions typical of areas north of Houston, flooding of agricultural land could release animal waste associated with areas used for animal grazing (Gentry et al., 2007). In suburban watersheds typical of the greater Houston-Galveston area, rainfall could accelerate the resuspension and transport of waste from onsite sewage facilities, such as residential septic tanks (Morrison et al., 2017). Indeed, waterways in the Houston-Galveston area frequently exceed fecal indicator bacteria (FIB) criteria during high flow and flood events (Petersen et al., 2006; TCEQ, 2013). Little is known about the health risks associated with exposure to sewage and other human waste in floodwaters in urban, industrialized watersheds (Ahern et al., 2005). Human waste presents a particular health threat (Soller et al., 2014) and the perception that floodwater is contaminated with sewage could further alarm and mentally traumatize the public and hamper recovery efforts (Few and Matthies, 2006; Du et al., 2012). These threats to public health are expected to worsen, as several models predict that the intensity, if not the frequency, of tropical cyclones and hurricanes will increase over the next few decades (Webster et al., 2005; Knutson et al., 2008).

Extreme weather events could also alter the quality of receiving waters. Flooding can result in release of petroleum products and other hazardous materials that could stress aquatic systems (Rozas et al., 2000; O’Donnell, 2005; Girgin and Krausmann, 2016). This environmental risk is high in the Galveston Bay systems; the Houston Ship Channel is the largest petrochemical complex in the United States (Bridges, 2019). Floodwaters can also temporarily alter nutrient cycles. For example, Hurricane Bob, a category 3 storm when it landed on Cape Cod, increased nutrient loading to estuaries in Cape Cod, Massachusetts, but the system appeared to recover rapidly (Valiela et al., 1998). Hurricane Ivan exacerbated eutrophication in Pensacola Bay, Florida temporarily, but the system recovered in a few days (Hagy et al., 2006). The extent to which these few studies can be extrapolated to other areas with unique geographies reflects the paucity of data and inherent challenges of quantifying multiple stressors during extreme, yet ephemeral, events (Córdova-Kreylos et al., 2006). Metagenomic methods have the potential to provide additional insight the health of aquatic systems, particularly with respect to extreme weather events (Ghaju Shrestha et al., 2017).

Here we apply metagenomics to determine the impact of Hurricane Harvey (HH) on the health of Clear Lake, an estuary between Houston and Galveston that connects with upper Galveston Bay. This estuary is popular with anglers and boaters and is routinely monitored by a consortium of state agencies, non-profits and academic institutions. We collected water samples at stations within well-defined water body segments that represent a range of salinity (fresh to brackish) and nutrients. Stations were sampled immediately before and after landfall of Hurricane Harvey, and then weekly into the fall. These samples were analyzed for fecal indicator bacteria (FIB), dissolved inorganic nutrients (DIN) and microbial community structure, as assessed by targeted metagenomic analysis of 16S rRNA gene fragment amplicons. FIB counts, DIN concentrations and other environmental parameters were compared to data mined from public archives. Relative to pre-storm levels, and values typical for waterbodies sampled herein, HH elevated FIB counts and lowered DIN and salinity concentrations. The structure of the community shifted from a community dominated by *Cyanobacteria* and A*ctinobacteria* before the storm to a community dominated by the phyla *Proteobacteria* and *Bacteroidetes* immediately after the event. Shifts in the microbiological community structure corresponded to changes in salinity and NO_*x*_ concentrations.

## Materials and Methods

### Sampling Locations and Collection

**FIGURE 1:**
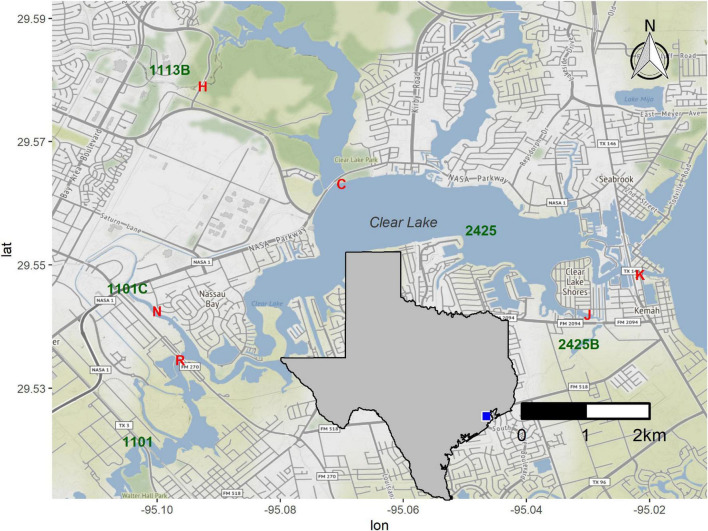
Sampling stations around Clear Lake. Stations sampled in this study are indicated by red letters (H, C, K, J, R, and N). Water body segments as defined by the TCEQ (1113B, 2424, etc.) are indicated in green. Inset indicates study area within a map of Texas. Figure was generated with scripts in Supplementary File 1.

We selected sites around Clear Lake, an estuary between Houston and Galveston (Figure 1), based on the availability of long-term water quality data for these locations and ease of access. Water samples were collected on the afternoon of August 24th, 2017, 1 day before Hurricane Harvey landed in the Corpus Christi area. These samples, designated “pre” in this work, were collected at baseflow conditions (Supplementary Figure 1). A second set of samples was collected on August 30th. These samples, designated “HH” throughout, correspond to Hurricane Harvey samples and were collect hours after flow of a major tributary into Clear Lake peaked (Supplementary Figure 1). We added a second sampling site (N) when collecting the HH set to collect floodwater received by segment 1101C (Figure 1). Starting on September 8th we sampled six times to generate a “post” sample set; all post-HH samples were collected during baseflow conditions (Supplementary Figure 1). Samples collected in August and September were designated as summer season. Samples collected in October were designated as fall season. We also generated a mock sample by mixing raw sewage, collected as described previously (Amaral-Zettler et al., 2008), and surface water collected from station H (Figure 1) in March of 2018. The sewage and water were mixed at a ratio of 1 part sewage with 9 parts surface water.

At each sampling station, we collected surface water samples with a bucket lowered from an overpass or dock. Temperature and dissolved oxygen were measured *in situ* at 3–5 cm beneath the surface with a YSI model 55 dissolved oxygen (DO) probe (YSI Inc., Young Spring, OH). Water samples were split in the field for FIB (*E. coli* and *Enterococci*), metagenomic and nutrient analysis. For FIB analysis, unfiltered samples were transported on wet ice and stored at 4°C. Incubations for quantification of FIB were initiated within 24 h of sampling. FIB samples from pre-HH samples were discarded because we were locked out of our laboratory for several days and sampling holding times were exceeded.

Water samples collected before HH were stored on wet ice, returned to the laboratory and filtered to collect microbial samples for metagenomic analysis and to archive nutrient samples within 24 h of collection. Following HH, all samples were filtered in the field immediately upon collection. For metagenomic analysis, water samples were pulled through a Sterivex SVGPL10RC 0.2 μm cartridge (EMD Millipore, Billerica MA) until refusal (no flow at 15 psi), with a hand vacuum pump, as described previously (LaMontagne and Holden, 2003). The volume filtered, which ranged from 75 and 300 ml, was measured with a graduated cylinder. Sample filtrates and cartridges were transported on wet ice, temporarily stored at −20°C, and archived at −80°C. Two technical replicates were generated by collecting duplicate samples from station J on the eve of the storm and from the sewage-spiked samples described above.

### Laboratory Methods

Dissolved inorganic nutrient analysis for ammonium, orthophosphate, and nitrate were done by colorimetric analysis in microplates, as described previously (Ringuet et al., 2011). *E. coli* and *Enterococci* were enumerated using Colilert and Enterolert in the Quanti-Tray/2000 format following manufacturer recommendations (IDEXX, Westbrook, ME). Microbial community DNA for metagenomic analysis was recovered from the Sterivex cartridges as described previously (Amaral-Zettler et al., 2008) and assessed for molecular weight by agarose gel electrophoresis. These crude extracts were subsequently purified by passage through a OneStep PCR Inhibitor Removal column (Zymo, D6030) and purity was assessed by UV-spectra. Metagenomic analysis followed protocols outlined in the Earth Microbiome Project (Caporaso et al., 2011). Briefly, the V4 region of the 16S rRNA gene was amplified to generate an amplicon library. This library was multiplexed using Illumina designed indices, pooled with equal amounts, and sequenced on an Illumina MiSeq instrument as described by Caporaso et al. (2012).

### Data Analysis

Water quality data were analyzed and figures were generated with custom scripts presented in Supplementary Files 2, 3. These scripts included functions from packages from ggplot2 (Wickham, 2009). This data set included water quality data collected as described above and public data previously collected by the Texas Commission for Environmental Quality (TCEQ) and cooperating organizations. Data from the TCEQ archive was limited to samples collected between January 1st, 2011 and May 6th, 2021. For this time period, the mean for each of these five segments was calculated by grouping by segment, month and year. This data range was then merged with water quality data generated from samples collected in this study to generate Supplementary File 4.

MiSeq data were processed to determine alpha diversity of the microbial community using functions from DADA2 v. 1.20.0 (Callahan et al., 2016), with custom scripts presented in Supplementary File 5. Briefly, reads were filtered, trimmed, denoised, and merged to yield sequences from 251 to 253 nucleotides long. Chimeras were then removed with the function removeBimeraDenovo in DADA2 and putative non-chimeric sequences were assigned taxonomy and aligned with the functions IdTaxa and AlignSeqs in Decipher v 2.20.0 (Wright et al., 2012). Amplicon sequence variants (ASVs) and taxonomic identifications were merged to create a phylogseq-class object—available as Supplementary File 6—with functions in phyloseq v 1.36.0 (McMurdie and Holmes, 2013). ASVs with uncertain taxonomic identification at the phylum level were then removed before fitting the alignments into a phylogentic tree with functions in phangom v 2.7.1 (Schliep, 2010). Technical replicates (two samples collected at the same time and place but processed independently) were then merged and meta-data (volume filtered, environmental conditions, FIB counts, DIN, etc.) and reference sequences were combined to create a phylogseq-class object—available as Supplementary File 7—with functions in phyloseq and Biostrings (v 2.60.2 Pagès et al., 2021). Reference sequences were also exported in fasta format and compared to public sequences with the Seqmatch application (RDP Taxonomy 18) available through the Ribosomal Database Project (Cole et al., 2013). Default settings were used in Seqmatch. MiSeq data is available in the NCBI SRA under accession number/Bioproject ID: PRJNA795782.

Alpha diversity (richness and Shannon indices) was estimated with the plot_richness function after sewage-spiked samples were removed. Analysis of variance, calculated with a core function in R version 4.1.1, was used to test for significance of differences between samples collected before HH made land fall (sampled August 25th), vs. samples collected immediately after the storm (August 30th) and in September and October. Significance of differences was assessed with a Tukey test using the function HSD.test in the R package agricoloe v 1.3.5 (de Mendiburu, 2020). Coverage of the library of reads used for diversity analysis was visualized with the function rarecure in vegan v 2.5.7 (Oksanen et al., 2020).

The relationship between microbial community structure and nutrient levels was determined with correspondence analysis with custom scripts presented in R markdown in Supplementary File 8. This workflow started with a phyloseq object (S7). A prevalence threshold of 10% was set to remove rare taxa. Counts of the remaining 1,616 ASVs were transformed with Hellinger option prior to non-metric multidimensional scaling analysis (NMDS) with functions in vegan v 2.5.7 (Oksanen et al., 2020). NMDS was first run with all 44 samples, prior to removal of sewage-spiked samples. Goodness of fit of NMDS ordination was visualized with a Shepard plot generated prior to fitting meta-data to the ordination with functions in vegan. The resulting ordination plots were visualized with functions available in R package ggordiplots v 0.4.0 (Quensen, 2020).

Functional composition was predicted from the 1,616 ASVs used in NMDS analysis (above) with PICRUSt2 v 2.3.0-b (Douglas et al., 2020). To prepare the data, an ASV abundance table and fasta files were exported from a phyloseq object (S09) to a biom file (S10) and a sequence file (S11) using R package biomformat v 1.20.0 (McMurdie and Paulson, 2021). This pipeline, including bash scripts used in PICRUCSt2 analysis, are presented in Supplementary File 12.

Differential abundance of pathways (Supplementary Files 13, 14) predicted from ASVs and ASVs themselves was assessed using functions in R package ANCOM-BC v 1.2.2 (Lin and Peddada, 2020), following scripts presented in Supplementary File 15. Pathways that were differentially abundant were plotted with functions available in R package Heatplus v 3.0.0 (Ploner, 2021), following scripts presented in Supplementary File 16. Pathway functions and expected taxonomic range associated with them were identified with the web application MetaCyc v 25.5 (Caspi et al., 2013).

## Results

### Environmental Conditions

**FIGURE 2:**
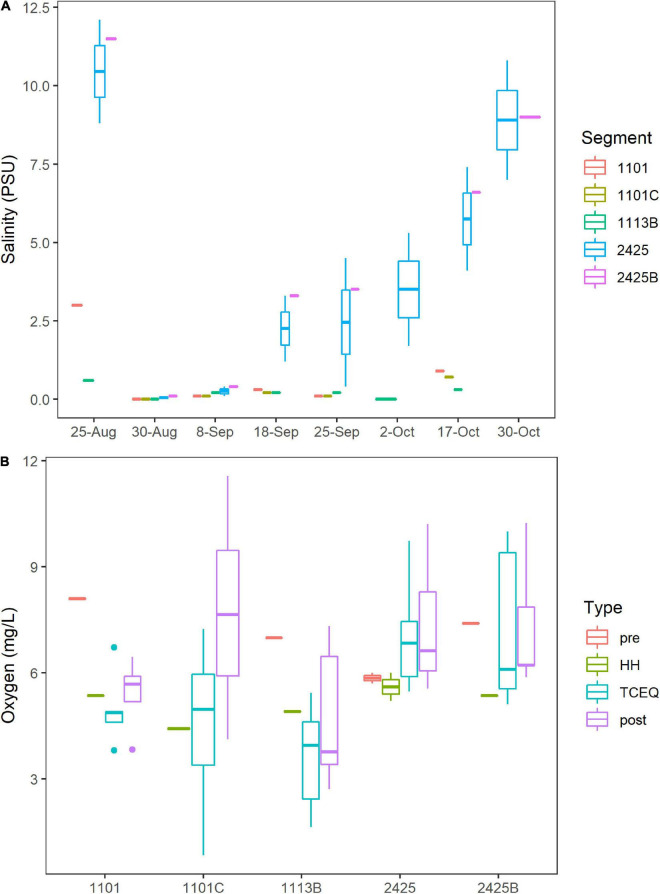
Salinity and Oxygen. Water body segments are as Figure 1. Boxes indicate 25 and 75% quantiles. Whiskers indicate range. Horizontal lines indicate median or, for segments where only one sample was taken, the value for that individual sample. Figure was generated with scripts in Supplementary File 3. **(A)** Salinity concentrations in practical salinity units (PSU). All dates are 2017. **(B)** Boxplots of oxygen levels in the Clear Lake system. Sample type “pre” indicates samples collected on August 25th (before HH). Type “HH” indicates samples collected on August 30th (immediately after HH). Type “post” indicates samples collected from September 8th to October 30th. Type “TCEQ” indicates historical data collected by TCEQ and partner agencies during 2011–2021.

Hurricane Harvey lowered the salinity for Clear Lake. On the eve of HH (August 25th, 2017), surface salinity at stations C, K, and J, which correspond to water body segments 2425 and 2425B (Figure 1), ranged from 9 to 12 practical salinity units (PSU, Figure 2A). These pre-HH salinity levels are within the 95% confidence interval of the 10-year average for salinity for records for these two water body segments (2425 and 2425B, Supplementary Figure 1). Immediately after the storm, salinity dropped to < 1 PSU at all stations sampled herein and then gradually increased to pre-storm levels over the next 2 months (Figure 2A).

Oxygen levels in Clear Lake and tributaries to that system did not show a strong response to HH. Across all segments, oxygen levels averaged 6.8 mg/L before HH and 5.2 mg/L after. This temporal difference was not significant (*p* = 0.131); however, spatial differences between segments were significant (*p* = 0.0003). Oxygen levels averaged 6.9–7.1 mg/L for segments 2425 and 2425B, respectively, and less than 6 mg/L for stations 1101C, 1101, and 1113B (Figure 2B). Lowest oxygen concentrations were observed at station H in waterbody segment 1113B, where DIN concentrations are relatively high (see below).

Hurricane Harvey lowered the concentration of dissolved inorganic nutrients (DIN). On the eve of the storm, nitrate/nitrite (NO_*x*_) ranged from 1 to 59 μM (Supplementary Figure 3). These pre-HH NO_*x*_ levels are in the range for records for the last 10 years for these segments (Supplementary Figure 3). Immediately after the storm, NO_*x*_ ranged from 1 to 9 μM and varied significantly between segments (*p* < 0.001) and type (pre-storm, HH, and post-storm). Highest levels of DIN were observed for samples collected from segment 1113B, which is approximately 50 m downstream of the outfall pipe of a wastewater treatment plant.

**FIGURE 3:**
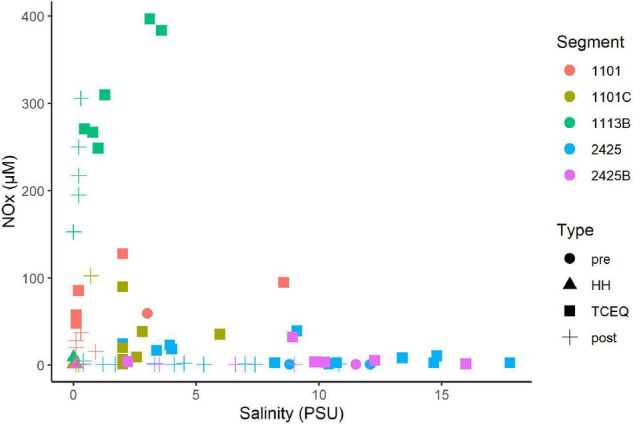
Mixing diagram of salinity vs. nitrate/nitrite for the Clear Lake system. Note DIN data is not available for the sample collected before HH from waterbody segment 1113B. Segments and types are as Figure 2. Figure was generated with scripts in Supplementary File 3.

NO_*x*_ showed a non-conservative mixing relationship with salinity (Figure 3). That is the system is a sink NO_*x*_. High concentrations (>10 μM) were associated with samples that showed salinities of 3 PSU or less. In contrast, samples with higher salinities (>3 PSU), typically showed NO_*x*_ levels of 3 μM or less, which suggests a freshwater source. Ammonium and phosphate levels showed a similar pattern with salinity as NO_*x*_. High concentrations of ammonium (Supplementary Figure 4) and phosphate (Supplementary Figure 5) were associated with low salinities. DIN/P ratios were generally below 16 (Supplementary Figure 6). These ratios were on average highest (7–8) for segments 1101C and 1113B, respectively, and < 2 for segments 2425 and 2425B.

### Fecal Indicator Bacteria

**FIGURE 4:**
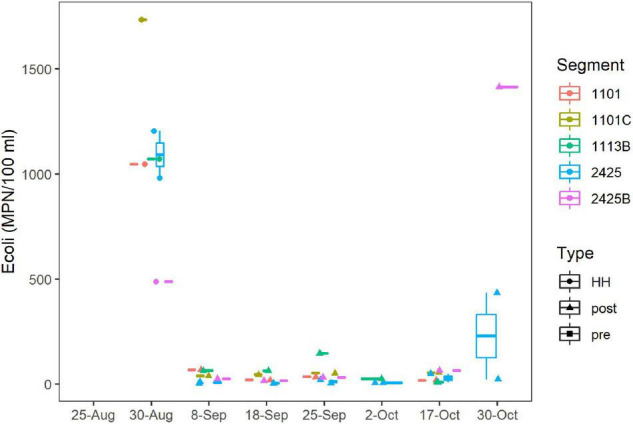
*E. coli* concentrations over time. MPN/100 ml were assessed by culturing as described in Methods. Dates are as Figure 2A. Figure was generated with scripts in Supplementary File 3.

*E. coli* levels ranged from 488 to 1,733 MPN/100 ml for the six stations sampled on September 1st, 2017 (Figure 4), which was 72 h after HH pasted over the study area. The geometric mean (GM) for this set of samples was 1,018 MPN/100 ml. These values exceeded the statistical threshold value (STV) for single samples and GM recommended for water contact by the EPA (USEPA, 2012) and by the TCEQ for these particular water body segments (TCEQ, 2013). After 1 week, the *E. coli* levels had decreased to < 100 MPN/100 ml and remained relatively low until the end of October, when levels spiked again.

*Enterococci* levels ranged from 63 to 3,050 MPN/100 ml for samples collected in the fall of 2017; however, because of logistical issues, *Enterococci* levels were not measured until September 18th. For this period (post-HH), *Enterococci* levels did not differ between segments sampled (Supplementary Figure 7), and 22 of 24 samples exceeded the STV for single samples recommended for recreational water contact by the EPA (USEPA, 2012); GM (495 MPN/100 ml) exceeded, by an order of magnitude, the GM recommended for recreational water contact by the EPA (USEPA, 2012). FIB counts of samples taken in the post-HH period, were significantly higher (*p* < 0.001) than counts for the same segments collected over the last decade, where the GM of *Enterococci* was 74 MPN/100 ml.

### Microbial Diversity

**FIGURE 5:**
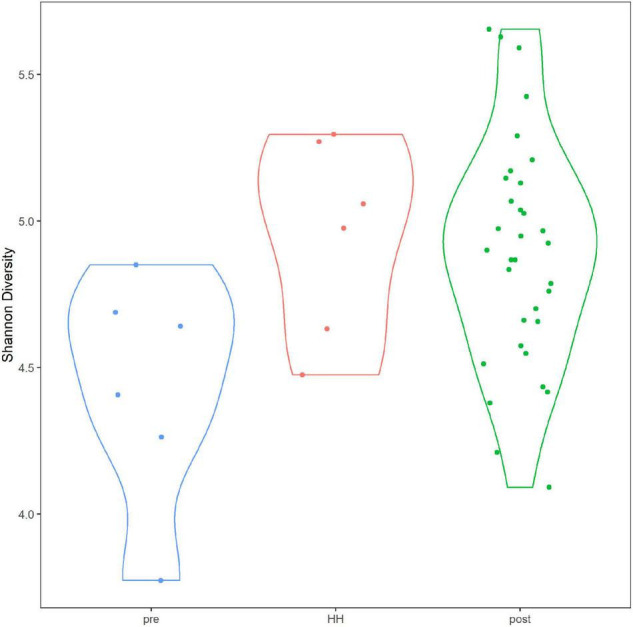
Alpha diversity in Clear Lake system before and after Hurricane Harvey. *Y*-axis indicates Shannon diversity indices. Categories correspond to before (pre), immediately after (HH) and more than a week after (post). Figure was generated with scripts in Supplementary File 5.

Alpha diversity of the bacterial and archaeal community did not differ significantly between samples collected before and after HH (Figure 5). Average Shannon diversity indices ranged from 4.95 to 4.89 for samples after the event and averaged 4.44 for samples collected immediately before the storm. Diversity was relatively lower for samples collected before HH at stations 1101 and 1113B but only one sample was collected at that time point (Supplementary Figure 8). Average richness ranged from 761 to 633 for samples collected after the event and 488 for samples collected before (Supplementary Figure 9). Rarefaction analysis suggested the sequence library appeared to have the depth to describe alpha diversity (Supplementary Figure 10). After quality control, which included removing sequences that did not classify at the phylum level, average depth of the library was 112,178 reads. In other words, all 44 samples reached an asymptote.

**FIGURE 6:**
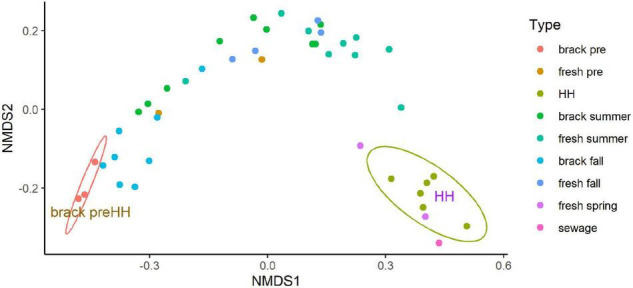
Non-metric multidimensional scaling analysis (NMDS) of microbial community structure for samples collected before and after Hurricane Harvey. NMDS was run on the abundance of amplicon sequence variants as described in Methods. Type indicates segment (see Figures 1, 2) sampled and season: “brack” corresponds to segments 2245 and 2245B, “fresh” corresponds to segments 1101, 1101C, and 1113B, “sewage” indicates a sewage-spiked sample and “HH” indicates samples collected immediately after HH. Eclipses were drawn to highlight indicated two coherent clusters supported by 95% confidence intervals. Figure was generated with scripts in Supplementary File 8.

Beta diversity of the bacterial and archaeal community structure, as assessed by NMDS, differed significantly between samples collected before and immediately after HH (Figure 6). The good fit (*r*^2^ = 0.999) of a stressplot (Supplementary Figure 11), and low stress value (0.036), suggest this model is an excellent fit (Dexter et al., 2018). NMDS showed two clear clusters. Three samples collected before HH in segments (2425 and 2425B), that showed brackish salinities (8–12 PSU), formed a coherent cluster, with similarity to samples collected in the same segments in fall. Samples collected immediately after HH also formed a coherent cluster, with similarity to a sample spiked with sewage. One sample collected in March 2018 in segment 1113B clustered with the HH samples.

Recovery of Clear Lake progressed from the summer through the fall in segments (2425 and 2425B). These segments typically have brackish conditions. In the summer following HH the microbial community within these stations within Clear Lake looked similar, in terms of NMDS, to communities sampled from freshwater tributaries to the estuary (Figure 6). This recovery of the estuary’s microbiome appeared driven by salinity (Supplementary Figure 12). Salinity appeared strongly (*p* = 0.001) associated with pre-HH samples. NO_*x*_ and oxygen appeared strongly (*p* = 0.016 and 0.020, respectively) associated with post-HH samples. Phosphate also appeared associated with post-HH samples but the significance was weak (*P* = 0.086).

**FIGURE 7:**
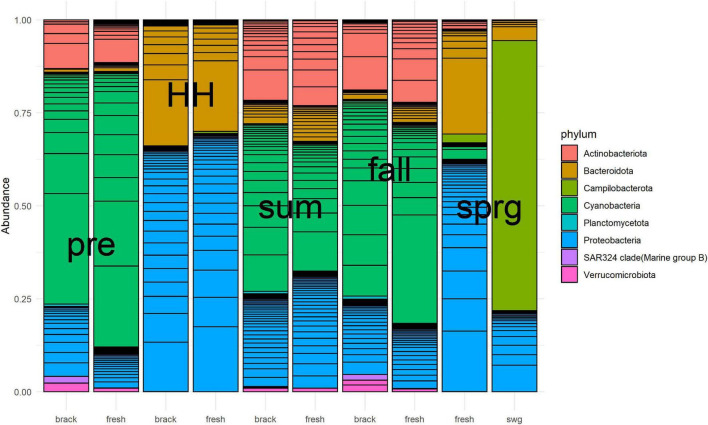
Relative abundance of numerically dominant microbes in Clear Lake for samples collected from August 2017 to March 2018. Samples were merged by baseline salinity of segment (brack: 2425 and 2425B, fresh: 1101, 1101C, and 1113B) and type [pre-HH (pre), HH, summer (sum), fall and spring (sprg)]. A sewage-spiked (swg) sample is included for comparison. The hundred most abundant ASV are shown. Figure was generated with scripts in Supplementary File 8.

Bacterial community structure of the Clear Lake system shifted from a system dominated by Cyanobacteria before HH to a system dominated by *Proteobacteria* and *Bacteroidetes* immediately after (Figure 7). SAR324 clade (Marine group) was relatively abundant before HH and in the fall in segments we defined as brackish (2425 and 2425B). A total of 59 phyla were detected in 7,491 ASVs generated from 44 samples. Almost all of these ASVs (7,410/7,491) classified as bacteria. After removing taxa with relatively low (<10%) prevalence, almost all of the ASVs (1,534/1,617) were found to be differentially abundant, at a significance threshold of *P* < 0.05, in a model that tested the factors: salinity, NOx, and sample type (pre, HH, and post). In other words, these factors predicted the abundance of 95% ASVs.

Sample type predicted the majority of abundances. For example, the abundance of 819 (51%) ASVs differed between pre-HH and HH samples and the abundance of 1,007 (62%) ASVs differed between pre-HH and post-HH samples. Of the 10 most abundant ASVs observed in samples collected immediately after HH, nine classified as γ-*Proteobacteria*. Most of these (7/9) classified within the family *Comamonadaceae* and showed similarity to bacteria typically observed in freshwater systems. For example, ASV18 showed similarity to *Limnohabitans curvus* MWH-C1a, which was isolated from a lake (Hahn et al., 2010). The other two highly abundant ASVs (ASV64 and ASV23) showed similarity to γ*-Proteobacteria* isolated from rhizosphere soil (Jung et al., 2007) and freshwater systems (Hahn, 2003), respectively. ASV6, the most abundant ASV in libraries generated from floodwater samples, accounted for 5–17% of the reads generated in those six libraries. This ASV showed similarity to *Aquirufa* strains isolated from lakes (Hahn, 2006; Lee et al., 2018). The ASV showed the greatest differential abundance between pre-HH and HH samples (ASV103) showed high similarity to two uncultured bacteria (KP686762 and KP686755) generated from floodwater collected in North Carolina immediately after Hurricane Irene (Balmonte et al., 2016).

**FIGURE 8:**
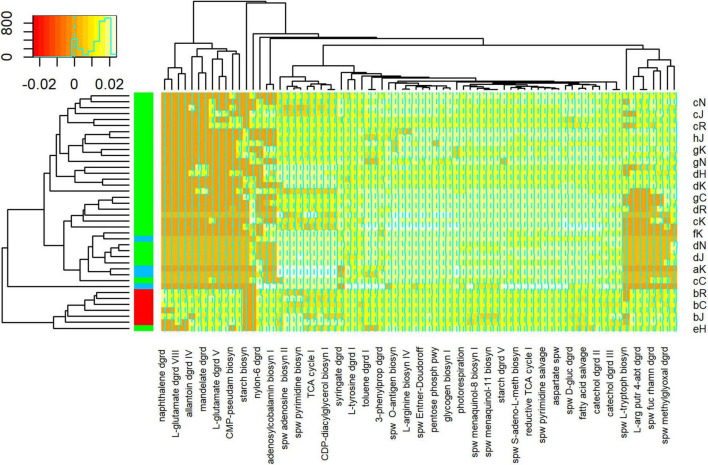
Heatmap of Metabolic Pathway Abundances. Pathways on lower horizontal axis were predicted from ASVs with PICRUSt2 as described in Methods. Representative sample codes are indicated on right vertical axis, where lower case letter indicates date (see Supplementary Figure 1) and upper case letter indicates station (see Figure 1). Sample types are indicated by colors in block on left vertical axis, where blue = pre, red = HH, and green = post. Inset shows key for counts of pathways in colors and a histogram. Figure was generated with scripts in Supplementary File 15.

PICRUSt2 analysis predicted the abundance of 418 metabolic pathways from 1,616 ASVs generated from 44 samples. Differential abundance analysis suggested that 76 of these pathways were significantly different between samples. Cluster analysis, based on the relative abundance of these 76 pathways, suggested that floodwater samples formed a coherent group (Figure 8). That is, with one exception (sample eH), floodwater samples were relatively similar to each other in terms of predicted pathways. The outlying sample was also similar to samples collected immediately after HH in terms of numerically abundant phyla. In particular, eH and floodwater samples showed relatively high proportions of *Proteobacteria* and *Bacteroidetes*.

Comparison of pathways predicted from samples collected immediately before and after HH, identified 29 differentially abundant pathways (Supplementary Figure 12); 14 of these were significantly higher in samples collected before HH and 15 were significantly higher after the storm. Half (7/14) of the pathways associated with samples collected before HH were biosynthesis pathways. These include PWY-5347, which produces methionine and PWY-5840, which produces menaquinol-7. In contrast, only 3 of 14 pathways that were differentially abundant in samples collected immediately after HH were biosynthesis pathways, and two of these biosynthetic pathways are associated with virulence. PWY0-1338 confers resistance to the antibiotic polymyxin and PWY-6143 produces pseudaminic acid, which is associated with pathogenic Gram negative bacteria (Schirm et al., 2003). The vast majority (11/15) of pathways that were more abundant in samples collected immediately after HH were degradation pathways. These include pathways ORNDEG-PWY, ARGDEG-PWY, and ORNARGDEG-PWY, which are associated with degradation of L-arginine, putrescine, 4-aminobutanoate, and L-ornithine (Caspi et al., 2013).

Salinity and NO_*x*_ levels appeared associated with the abundance of 30 pathways. Of these 12 of were associated positively with salinity and 13 were associated negatively (Supplementary Figure 13). All but one of the pathways positively associated with salinity were biosynthesis pathways. These included five pathways (PWY-6165, 6349, −6350, −6654, −6167) associated with archaea and PWY-622, which is associated with starch biosynthesis by photoautotrophs (Caspi et al., 2013). In contrast, 6 of 13 pathways negatively associated with salinity were degradation pathways. These included two pathways (PWY-5427, −6956) associated with naphthalene degradation by bacteria and PWY-5088, which is associated with glutamate degradation by members of the Firmicutes phylum (Caspi et al., 2013). NO_*x*_ concentrations appeared associated with the abundance of five pathways (Supplementary Figure 14). The two positively associated pathways were degradation pathways; both are associated with mandelate degradation by *Proteobacteria*. Pathways negatively associated with NO_*x*_ concentrations include PWY-6174, which is associated with the mevalonate pathway in archaea, and PWY-5183, which is associated with toluene degradation by *Proteobacteria* (Caspi et al., 2013).

## Discussion

Rising sea levels and warming waters, associated with global warming, are predicted to increase the frequency of coastal flooding (Vitousek et al., 2017). Global warming is also expected to increase the severity of hurricanes (Knutson et al., 2021). These climate driven changes could alter the structure of coastal systems and offshore systems (Shore et al., 2021) and more frequently bring many people into contact with floodwater. This creates a public health risk (Cann et al., 2012; Du et al., 2012). The response of the system and risks to the populace will vary depending on the system and storm. Here we studied the water quality and microbial communities of samples collected from the Clear Lake system, a rapidly developing area between Houston and Galveston. Hurricane Harvey temporarily shifted the structure of the Clear Lake system from a brackish (∼ 10 PSU), estuary, fed by eutrophic, fresh tributaries, to a freshwater system, with little difference between the lake and tributaries in terms of salinity, nutrients and other chemical parameters.

The temporary shift to a freshwater system was accompanied with a dramatic, temporary, decrease in cyanobacteria. In parallel, γ*-Proteobacteria*, which are typically observed in soils and freshwater systems, increased. This pattern of dilution and recovery is consistent with a model of the recovery time for salinity in that system (Du and Park, 2019), but is a few weeks slower for the time reported for salinity recovery for Galveston Bay (Steichen et al., 2020). Overall, the recovery of the Galveston Bay system appears slower than estuaries impacted by Hurricane Bob (Valiela et al., 1998) and estuaries impacted by multiple hurricanes in North Carolina (Peierls et al., 2003) and the shift in bacterial community structure is consistent with changes reported following HH for Galveston Bay (Yan et al., 2020).

Bacteria dominated this system, as assessed by metagenomic analysis of PCR-amplified 16S rRNA gene fragments, and the structure of this community corresponded to salinity, DIN and oxygen concentrations. The relationship between salinity and bacterial community structure parallels a report that salinity corresponded to changes in viral community structure in Galveston Bay following HH (Woods et al., 2022). These results also agree with a previous study of systems in Louisiana impacted by Hurricanes Katrina and Rita (Amaral-Zettler et al., 2008) and with previous reports for estuaries in general (Tee et al., 2021).

The strong influence of DIN corresponds to the dogma that nitrogen limits productivity in coastal systems. That is, if nitrogen limits primary production, a change in nitrogen availability would change the entire system. Indeed, low N/P ratios suggests that nitrogen limits productivity in Clear Lake, which is consistent with Ryther and Dunstan’s dogma (Ryther and Dunstan, 1971); however, only inorganic nutrients were measured herein. Organic matter also contains significant pools of nitrogen and phosphate. For example, in Galveston Bay total nitrogen concentrations were about 5X higher than DIN concentrations for samples collected following HH (Steichen et al., 2020).

Oxygen was not depleted significantly in water segments sampled herein following HH, relative to pre-storm levels and historical records, but oxygen levels did relate to microbial community structure (Supplementary Figure 12). Hypoxic conditions (<3 mg/L) were only observed once in this study. This agrees with a previous report for Bayous in the Houston-Galveston area, where relatively rural watershed receiving waters, like Peach Creek, did not go hypoxic, with the exception of the headwaters of Clear Creek (Kiaghadi and Rifai, 2019). The general lack of hypoxia in this system contrasts with previous reports for other systems in the Gulf of Mexico. For example, hypoxia persisted in Pensacola Bay for months following Hurricane Ivan (Hagy et al., 2006) and floodwaters overlying New Orleans were hypoxic following Hurricane Katrina (Pardue et al., 2005).

High *E. coli* MPNs for samples collected immediately after HH, suggests that floodwaters were contaminated with fecal matter. These elevated MPNs agree with previous reports for Bayous within the Galveston Bay system (Yu et al., 2018; Kiaghadi and Rifai, 2019; Yang et al., 2021), for the Guadalupe River (Kapoor et al., 2018), which was also in the path of HH, and the report of Enterobacteriaceae in marine sponges offshore of Galveston Bay (Shore et al., 2021). The EPA and TCEQ (2013) recommend *Enterococci* for estuaries and coastal waters; however, because of logistical issues, *Enterococci* MPNs were not available for several weeks after HH. Levels of these FIB remained elevated relative to typical levels for this system for weeks (Supplementary Figure 7). These high MPNs agree with the observation that bacteria typically observed in human waste, such as *Bacteroides* spp., abounded in libraries generated from all samples collected immediately following HH (Figure 7).

PICRUSt2 analysis suggested that flooding also enriched for antibiotic resistant genes (ARG), virulence factors and carbon cycling pathways. These predictions of functional genes from rRNA sequences, and the inference of microbial community structure from targeted metagenomic analysis in general, should be treated with caution. Every step in targeted metagenomic analysis, from sampling to data analysis is fraught with bias (Pollock et al., 2018). In particular, PICRUSt2 depends on reference genomes, which are largely derived from the human gut microbiome. This creates a bias depending on the sample type (Sun et al., 2020). For example, PICRUSt2 underestimates certain pathways in soil systems (Toole et al., 2021).

Prediction of increase in carbon cycling bacteria agrees with reports that loading of dissolved organic carbon (DOC) during extreme weather events can enhance carbon cycling by bacterial communities in receiving waters (Balmonte et al., 2016) and high DOC levels in Galveston Bay following HH (Steichen et al., 2020; Yan et al., 2020). Because of the velocity of water moving through the system, metabolic pathways associated with floodwaters sampled herein were ephemeral and do not suggest long term changes in microbial community functions for the Clear Lake system. Nevertheless, prediction of ARG and virulence factors with PICRUSt2 in samples collected immediately following HH suggests that these floodwaters could pose a public health risk. The abundance of these virulence factors agrees with previously published qPCR measurements of ARG in samples collected from soils flooded during HH (Pérez-Valdespino et al., 2021), in samples collected within Galveston Bay 2 weeks after HH (Yang et al., 2021), and ARG and pathogens in floodwaters and bayous following HH (Yu et al., 2018).

## Conclusion

The massive influx of freshwater from Hurricane Harvey into the Clear Lake system temporarily changed the system from a brackish estuary with relatively low levels of FIB and a microbial community dominated by primary producers, to a freshwater system with high levels of FIB. The microbial community observed immediately following the hurricane included bacteria that have also been reported in estuaries following hurricanes, but rarely elsewhere, and enrichment of antibiotic resistant bacteria. Recovery of the system to pre-storm conditions, in terms of nutrients and salinity, exceeded 2 months.

## Data Availability Statement

The datasets presented in this study can be found in online repositories. The names of the repository/repositories and accession number(s) can be found in the article/Supplementary Material.

## Author Contributions

ML, GG, TG, and MA conceived of the study. ML and GG selected the sampling locations and collaborated with TG on initial sample processing. ML prepared the samples for metagenomic analysis. YZ and MA conducted the metagenomic analysis and the conducted initial bioinformatics. ML wrote the scripts for bioinformatics and generated the figures. ML, YZ, GG, TG, and MA wrote the manuscript. All authors contributed to the article and approved the submitted version.

## Conflict of Interest

The authors declare that the research was conducted in the absence of any commercial or financial relationships that could be construed as a potential conflict of interest.

## Publisher’s Note

All claims expressed in this article are solely those of the authors and do not necessarily represent those of their affiliated organizations, or those of the publisher, the editors and the reviewers. Any product that may be evaluated in this article, or claim that may be made by its manufacturer, is not guaranteed or endorsed by the publisher.

## Supplementary Material

Supplementary Figure 1 Stream flow for Clear Creek. Data was downloaded for USGS station 08076997 from https://waterdata.usgs.gov/nwis/inventory?agency_code=USGS&site_no=08076997. Vertical lines indicate sampling dates. Lower case letters next to lines indicates dates, where a = August 25th, b = August 30th etc. Figure was generated with scripts in Supplementary File 15.

Supplementary Figure 2 Historical salinity data for Clear Lake system. Stations correspond to segments in Figure 1. Data is from 2011 to 2021. Figure was generated with scripts in Supplementary File 2.

Supplementary Figure 3 Nitrate/nitrite concentration by water body segments and sample type. Type and segment are as Figure 2. Figure was generated with scripts in Supplementary File 3

Supplementary Figure 4 Mixing diagram of salinity vs. ammonium for the Clear Lake system. Symbols are as Figure 3. Figure was generated with scripts in Supplementary File 3.

Supplementary Figure 5 Mixing diagram of salinity vs. phosphate for the Clear Lake system. Symbols are as Figure 3. Figure was generated with scripts in Supplementary File 3.

Supplementary Figure 6 Mixing diagram of N/P ratio vs. phosphate for the Clear Lake system. Symbols are as Figure 3. Figure was generated with scripts in Supplementary File 3.

Supplementary Figure 7 Comparison of historical *Enterococci* concentrations vs. concentrations following Hurricane Harvey. Symbols are as Figure 3. Figure was generated with scripts in Supplementary File 3.

Supplementary Figure 8 Alpha diversity of bacterial communities in the Clear Lake system before and after Hurricane Harvey. Shannon diversity indices were calculated by targeted metagenomic analysis, as described in Methods. Boxes, whiskers and horizontal lines are described in Figure 2. Sample types are described in Figure 3. Figure was generated with scripts in Supplementary File 5.

Supplementary Figure 9 Microbial richness in Clear Lake system before and after Hurricane Harvey. *X*-axis indicates number of amplicon sequence variants. Categories correspond to before (pre), immediately after (HH) and more than a week after (post). Figure was generated with scripts in Supplementary File 5.

Supplementary Figure 10 Rarefaction analysis of richness by number of reads. Symbols are as **Figure 9**. Figure was generated with scripts in Supplementary File 5.

Supplementary Figure 11 Shepards diagram showing fit of NMDS to dissimilarity of any two pairs of samples. Figure was generated with scripts in Supplementary File 8.

Supplementary Figure 12 Fit of environmental data to NMDS model of microbial community structure. Stress value for model was 0.032, which suggests an excellent fit. Environmental variables that showed a significant (*P* < 0.10) relationship with community structure are shown. Only samples with DIN data available are shown. Note conductivity and salinity vectors were practically identical to each other and would overlap, so only salinity vector is shown. Figure was generated with scripts in Supplementary File 8.

Supplementary Figure 13 Waterfall plot showing pathways enriched (red) or depleted (teal) in comparison of samples collected before or immediately after Hurricane Harvey. Figure was generated with scripts in Supplementary File 15.

Supplementary Figure 14 Waterfall plot showing pathways enriched (red) or depleted (teal) as function of salinity concentration. Figure was generated with scripts in Supplementary File 15.

Supplementary Figure 15 Waterfall plot showing pathways enriched (red) or depleted (teal) as function of nitrate concentration. Figure was generated with scripts in Supplementary File 15.
