# Supplementary material for: Hurricane Harvey Impacts on Water Quality and Microbial Communities in Houston, TX Waterbodies
Source: Front Microbiol. 2022 Jun 14;13:875234. doi: 10.3389/fmicb.2022.875234 (PMC9239555; doi:10.3389/fmicb.2022.875234)
Supplement: Supplementary file 7 [file Data_Sheet_7.ZIP › S05_HHdada.html]

Alpha Diversity Hurricane Harvey UHCL


# Alpha Diversity Hurricane Harvey UHCL

#### Michael G. LaMontagne

#### 12/31/2021

## R Markdown

This is R Markdown document generates ASVs from 16S amplicon reads in DADA2 and generates a phyloseq object that is passed off to phyloseq. That phyloseq object will be used in correspondence analysis to determine environmental variable that correspond to bacteria communities associated with floodwaters.

```
library(philentropy)
packageVersion("philentropy")
```

```
## [1] '0.5.0'
```

```
library(dada2)
```

```
## Loading required package: Rcpp
```

```
packageVersion("dada2")
```

```
## [1] '1.20.0'
```

```
library(gridExtra)
packageVersion("gridExtra")
```

```
## [1] '2.3'
```

```
library(ggplot2)
packageVersion("ggplot2")
```

```
## [1] '3.3.5'
```

```
library(phyloseq)
```

```
## 
## Attaching package: 'phyloseq'
```

```
## The following object is masked from 'package:philentropy':
## 
##     distance
```

```
packageVersion("phyloseq")
```

```
## [1] '1.36.0'
```

```
library(DECIPHER); packageVersion("DECIPHER")
```

```
## Loading required package: Biostrings
```

```
## Loading required package: BiocGenerics
```

```
## Loading required package: parallel
```

```
## 
## Attaching package: 'BiocGenerics'
```

```
## The following objects are masked from 'package:parallel':
## 
##     clusterApply, clusterApplyLB, clusterCall, clusterEvalQ,
##     clusterExport, clusterMap, parApply, parCapply, parLapply,
##     parLapplyLB, parRapply, parSapply, parSapplyLB
```

```
## The following object is masked from 'package:gridExtra':
## 
##     combine
```

```
## The following objects are masked from 'package:stats':
## 
##     IQR, mad, sd, var, xtabs
```

```
## The following objects are masked from 'package:base':
## 
##     anyDuplicated, append, as.data.frame, basename, cbind, colnames,
##     dirname, do.call, duplicated, eval, evalq, Filter, Find, get, grep,
##     grepl, intersect, is.unsorted, lapply, Map, mapply, match, mget,
##     order, paste, pmax, pmax.int, pmin, pmin.int, Position, rank,
##     rbind, Reduce, rownames, sapply, setdiff, sort, table, tapply,
##     union, unique, unsplit, which.max, which.min
```

```
## Loading required package: S4Vectors
```

```
## Loading required package: stats4
```

```
## 
## Attaching package: 'S4Vectors'
```

```
## The following objects are masked from 'package:base':
## 
##     expand.grid, I, unname
```

```
## Loading required package: IRanges
```

```
## 
## Attaching package: 'IRanges'
```

```
## The following object is masked from 'package:phyloseq':
## 
##     distance
```

```
## The following object is masked from 'package:philentropy':
## 
##     distance
```

```
## The following object is masked from 'package:grDevices':
## 
##     windows
```

```
## Loading required package: XVector
```

```
## Loading required package: GenomeInfoDb
```

```
## 
## Attaching package: 'Biostrings'
```

```
## The following object is masked from 'package:base':
## 
##     strsplit
```

```
## Loading required package: RSQLite
```

```
## [1] '2.20.0'
```

```
library(phangorn)
```

```
## Loading required package: ape
```

```
## 
## Attaching package: 'ape'
```

```
## The following object is masked from 'package:Biostrings':
## 
##     complement
```

```
packageVersion("phangorn")
```

```
## [1] '2.7.1'
```

```
library(vegan)
```

```
## Loading required package: permute
```

```
## Loading required package: lattice
```

```
## This is vegan 2.5-7
```

```
## 
## Attaching package: 'vegan'
```

```
## The following objects are masked from 'package:phangorn':
## 
##     diversity, treedist
```

```
packageVersion("vegan")
```

```
## [1] '2.5.7'
```

```
library(agricolae)
```

```
## Registered S3 methods overwritten by 'klaR':
##   method      from 
##   predict.rda vegan
##   print.rda   vegan
##   plot.rda    vegan
```

```
## 
## Attaching package: 'agricolae'
```

```
## The following object is masked from 'package:ape':
## 
##     consensus
```

```
packageVersion("agricolae")
```

```
## [1] '1.3.5'
```

```
library(dplyr)
```

```
## 
## Attaching package: 'dplyr'
```

```
## The following objects are masked from 'package:Biostrings':
## 
##     collapse, intersect, setdiff, setequal, union
```

```
## The following object is masked from 'package:GenomeInfoDb':
## 
##     intersect
```

```
## The following object is masked from 'package:XVector':
## 
##     slice
```

```
## The following objects are masked from 'package:IRanges':
## 
##     collapse, desc, intersect, setdiff, slice, union
```

```
## The following objects are masked from 'package:S4Vectors':
## 
##     first, intersect, rename, setdiff, setequal, union
```

```
## The following objects are masked from 'package:BiocGenerics':
## 
##     combine, intersect, setdiff, union
```

```
## The following object is masked from 'package:gridExtra':
## 
##     combine
```

```
## The following objects are masked from 'package:stats':
## 
##     filter, lag
```

```
## The following objects are masked from 'package:base':
## 
##     intersect, setdiff, setequal, union
```

```
packageVersion("dplyr")
```

```
## [1] '1.0.7'
```

```
sessionInfo()
```

```
## R version 4.1.1 (2021-08-10)
## Platform: x86_64-w64-mingw32/x64 (64-bit)
## Running under: Windows 10 x64 (build 22000)
## 
## Matrix products: default
## 
## locale:
## [1] LC_COLLATE=English_United States.1252 
## [2] LC_CTYPE=English_United States.1252   
## [3] LC_MONETARY=English_United States.1252
## [4] LC_NUMERIC=C                          
## [5] LC_TIME=English_United States.1252    
## 
## attached base packages:
## [1] stats4    parallel  stats     graphics  grDevices utils     datasets 
## [8] methods   base     
## 
## other attached packages:
##  [1] dplyr_1.0.7         agricolae_1.3-5     vegan_2.5-7        
##  [4] lattice_0.20-44     permute_0.9-5       phangorn_2.7.1     
##  [7] ape_5.5             DECIPHER_2.20.0     RSQLite_2.2.8      
## [10] Biostrings_2.60.2   GenomeInfoDb_1.28.2 XVector_0.32.0     
## [13] IRanges_2.26.0      S4Vectors_0.30.0    BiocGenerics_0.38.0
## [16] phyloseq_1.36.0     ggplot2_3.3.5       gridExtra_2.3      
## [19] dada2_1.20.0        Rcpp_1.0.7          philentropy_0.5.0  
## 
## loaded via a namespace (and not attached):
##   [1] colorspace_2.0-2            hwriter_1.3.2              
##   [3] ellipsis_0.3.2              GenomicRanges_1.44.0       
##   [5] rstudioapi_0.13             bit64_4.0.5                
##   [7] fansi_0.5.0                 codetools_0.2-18           
##   [9] splines_4.1.1               cachem_1.0.6               
##  [11] knitr_1.36                  ade4_1.7-18                
##  [13] jsonlite_1.7.2              Rsamtools_2.8.0            
##  [15] cluster_2.1.2               png_0.1-7                  
##  [17] shiny_1.7.1                 compiler_4.1.1             
##  [19] assertthat_0.2.1            Matrix_1.3-4               
##  [21] fastmap_1.1.0               later_1.3.0                
##  [23] htmltools_0.5.2             tools_4.1.1                
##  [25] igraph_1.2.7                gtable_0.3.0               
##  [27] glue_1.4.2                  GenomeInfoDbData_1.2.6     
##  [29] reshape2_1.4.4              ShortRead_1.50.0           
##  [31] fastmatch_1.1-3             Biobase_2.52.0             
##  [33] jquerylib_0.1.4             vctrs_0.3.8                
##  [35] rhdf5filters_1.4.0          multtest_2.48.0            
##  [37] nlme_3.1-152                iterators_1.0.13           
##  [39] xfun_0.25                   stringr_1.4.0              
##  [41] mime_0.12                   miniUI_0.1.1.1             
##  [43] lifecycle_1.0.1             zlibbioc_1.38.0            
##  [45] MASS_7.3-54                 scales_1.1.1               
##  [47] hms_1.1.1                   promises_1.2.0.1           
##  [49] MatrixGenerics_1.4.3        SummarizedExperiment_1.22.0
##  [51] biomformat_1.20.0           rhdf5_2.36.0               
##  [53] RColorBrewer_1.1-2          yaml_2.2.1                 
##  [55] memoise_2.0.0               sass_0.4.0                 
##  [57] labelled_2.8.0              latticeExtra_0.6-29        
##  [59] stringi_1.7.5               AlgDesign_1.2.0            
##  [61] highr_0.9                   klaR_0.6-15                
##  [63] foreach_1.5.1               BiocParallel_1.26.2        
##  [65] rlang_0.4.11                pkgconfig_2.0.3            
##  [67] matrixStats_0.61.0          bitops_1.0-7               
##  [69] evaluate_0.14               purrr_0.3.4                
##  [71] Rhdf5lib_1.14.2             GenomicAlignments_1.28.0   
##  [73] bit_4.0.4                   tidyselect_1.1.1           
##  [75] plyr_1.8.6                  magrittr_2.0.1             
##  [77] R6_2.5.1                    generics_0.1.1             
##  [79] combinat_0.0-8              DelayedArray_0.18.0        
##  [81] DBI_1.1.1                   haven_2.4.3                
##  [83] pillar_1.6.4                withr_2.4.2                
##  [85] mgcv_1.8-36                 survival_3.2-11            
##  [87] RCurl_1.98-1.5              tibble_3.1.4               
##  [89] crayon_1.4.1                questionr_0.7.5            
##  [91] utf8_1.2.2                  rmarkdown_2.11             
##  [93] jpeg_0.1-9                  grid_4.1.1                 
##  [95] data.table_1.14.2           blob_1.2.2                 
##  [97] forcats_0.5.1               digest_0.6.27              
##  [99] xtable_1.8-4                httpuv_1.6.3               
## [101] RcppParallel_5.1.4          munsell_0.5.0              
## [103] bslib_0.3.1                 quadprog_1.5-8
```

## list fastq

```
path <- "C:/Users/mglam/Dropbox/BIOINFO/Rscripts/HHdada/UNTreads" 
head(list.files(path))
```

```
## [1] "A6A_S33_L001_R1_001.fastq.gz"      "A6A_S33_L001_R2_001.fastq.gz"     
## [3] "C34SEP25_S40_L001_R1_001.fastq.gz" "C34SEP25_S40_L001_R2_001.fastq.gz"
## [5] "C35OCT2_S46_L001_R1_001.fastq.gz"  "C35OCT2_S46_L001_R2_001.fastq.gz"
```

## Including Plots

```
# Forward and reverse fastq filenames have format: SAMPLENAME_R1_001.fastq and SAMPLENAME_R2_001.fastq
fnFs <- sort(list.files(path, pattern="_R1_001.fastq.gz", full.names = TRUE))
fnRs <- sort(list.files(path, pattern="_R2_001.fastq.gz", full.names = TRUE))
# Extract sample names, assuming filenames have format: SAMPLENAME_XXX.fastq
sample.names <- sapply(strsplit(basename(fnFs), "_"), `[`, 1)
## plot some quality profiles from forward
plotQualityProfile(fnFs[1:2])
```

```
#saveRDS(fnFs, "HHfnFs.rds")
## and reverse
plotQualityProfile(fnRs[1:2])
```

```
#saveRDS(fnRs, "HHfnRs.rds")
```

## Filter reads

```
# Place filtered files in filtered/ subdirectory
filtFs <- file.path(path, "filtered", paste0(sample.names, "_F_filt.fastq.gz"))
filtRs <- file.path(path, "filtered", paste0(sample.names, "_R_filt.fastq.gz"))
head(filtFs)
```

```
## [1] "C:/Users/mglam/Dropbox/BIOINFO/Rscripts/HHdada/UNTreads/filtered/A6A_F_filt.fastq.gz"     
## [2] "C:/Users/mglam/Dropbox/BIOINFO/Rscripts/HHdada/UNTreads/filtered/C34SEP25_F_filt.fastq.gz"
## [3] "C:/Users/mglam/Dropbox/BIOINFO/Rscripts/HHdada/UNTreads/filtered/C35OCT2_F_filt.fastq.gz" 
## [4] "C:/Users/mglam/Dropbox/BIOINFO/Rscripts/HHdada/UNTreads/filtered/C6C_F_filt.fastq.gz"     
## [5] "C:/Users/mglam/Dropbox/BIOINFO/Rscripts/HHdada/UNTreads/filtered/CA25_F_filt.fastq.gz"    
## [6] "C:/Users/mglam/Dropbox/BIOINFO/Rscripts/HHdada/UNTreads/filtered/CA30_F_filt.fastq.gz"
```

## Filter and trim (skip in markdown)

```
t1 <- 219
t2 <- 160
t3 <- 20
#outHH <- filterAndTrim(fnFs, filtFs, fnRs, filtRs, trimLeft=t3, truncLen=c(t1,t2),
#                     maxN=0, maxEE=c(2,2), truncQ=2, rm.phix=TRUE,
#                     compress=TRUE, multithread=FALSE)
# saveRDS(outHH, "outHH.rds")
outHH <- readRDS("outHH.rds")
head(outHH)
```

```
##                                   reads.in reads.out
## A6A_S33_L001_R1_001.fastq.gz        151699    139654
## C34SEP25_S40_L001_R1_001.fastq.gz   142049    132852
## C35OCT2_S46_L001_R1_001.fastq.gz    164534    152672
## C6C_S34_L001_R1_001.fastq.gz        188069    176819
## CA25_S2_L001_R1_001.fastq.gz        191428    178167
## CA30_S8_L001_R1_001.fastq.gz        153976    140764
```

## Learn error rates (skip in markdown)

```
#errR <- learnErrors(filtRs, randomize = TRUE, multithread=TRUE, nbases = 1e8)
#errF <- learnErrors(filtFs, randomize = TRUE, multithread=TRUE, nbases = 1e8)
#saveRDS(errR, "errRhh.rds")
#saveRDS(errF, "errFhh.rds")
errR <- readRDS("errRhh.rds")
errF <- readRDS("errFhh.rds")
plotErrors(errF, nominalQ=TRUE)
```

## Dereplication

```
#derepFs <- derepFastq(filtFs, verbose=TRUE)
#derepRs <- derepFastq(filtRs, verbose=TRUE)
## Name the derep-class objects by the sample names
#names(derepFs) <- sample.names
#names(derepRs) <- sample.names
## run the core sample inference program
#dadaFs <- dada(derepFs, err=errF, multithread=TRUE)
#dadaRs <- dada(derepRs, err=errR, multithread=TRUE)
## insepct one
#dadaFs[[1]]
## merge paired reads
#mergers <- mergePairs(dadaFs, derepFs, dadaRs, derepRs, verbose=TRUE)
# Inspect the merger data.frame from the first sample
#saveRDS(mergers, "mergersHH.rds")
mergers <- readRDS("mergersHH.rds")
head(mergers[1])
```

```
## $A6A
##                                                                                                                                                                                                                                                                                                                  sequence
## 1                                                            ACGTAGGGTGCAAGCGTTAATCGGAATTACTGGGCGTAAAGCGTGCGCAGGCGGACTTTTAAGCCAGATGTGAAAGCCCCGAGCTTAACTTGGGAATTGCGTTTGGAACTGGGAGTCTAGAGTCTGTCAGAGGGGGATGGAATTCCACGTGTAGCAGTGAAATGCGTAGAGATGTGGAGGAACACCGATGGCGAAGGCAGTCCCCTGGGATAAGACTGACGCTCATGCACGAAAGCGTGGGGAGCAAACAGG
## 2                                                            ACAGAGGATGCAAGCGTTATCCGGAATCACTGGGCATAAAGCGTCTGTAGGTGGTTTGGTAAGTCTGCTGTTAAAGACTGGGGCTCAACCCCAGAAAAGCAGTGGAAACTGCTAGACTTGAGTGTGGTAGAGGTAGAGGGAATTCCTAGTGTAGCGGTGAAATGCGTAGATATTAGGAAGAACACCAATGGCGAAGGCACTCTACTGGACCATAACTGACACTGAGAGACGACAGCTAGGGGAGCAAATGGG
## 3                                                            ACGAAGGGTGCAAGCGTTATTCGGAATAATTGGGCGTAAAGGGTTCGTAGGTTGGATTGTAAGTCAAGTGTGAAATCCCCAGGCTTAACCTGGGACGTGCATTTGAAACTGTGATTCTTGAGTATTGGAGAGGGTAGTGGAATTGTAGGTGTAGGAGTGACATCCGTAGAGATCTGCAGGAACATCAGAGGCGAAGGCGACTACCTGGCCGATTACTGACACTGAGGAACGAAAGCGTGGGGAGCAAACAGG
## 4                                                            ACGGGGGGTGCAAGCGTTACTCGGAATCACTGGGCGTAAAGAGCATGTAGGCGGATTAATAAGTTTGAAGTGAAATCCTATAGCTTAACTATAGAACTGCTTTGAAAACTGTTAATCTAGAATGTGGGAGAGGTAGATGGAATTTCTGGTGTAGGGGTAAAATCCGTAGAGATCAGAAGGAATACCGATTGCGAAGGCGATCTACTGGAACATTATTGACGCTGAGATGCGAAAGCGTGGGGAGCAAACAGG
## 5                                                            ACGTAGGGTGCAAGCGTTAATCGGAATTACTGGGCGTAAAGCGTGCGCAGGCGGTTATATAAGTCAGATGTGAAATCCCCGGGCTCAACCTGGGAACTGCATTTGAGACTGTATAGCTAGAGTACGGTAGAGGGGGATGGAATTCCGCGTGTAGCAGTGAAATGCGTAGATATGCGGAGGAACACCGATGGCGAAGGCAATCCCCTGGACCTGTACTGACGCTCATGCACGAAAGCGTGGGGAGCAAACAGG
## 6                                                            ACATAGGGTGCAAGCGTTGTCCGGAATTATTGGGCGTAAAGAGCTCGTAGGTGGTTCGTCACGTCGGATGTGAAACTCTGGGGCTTAACCCCAGACCTGCATTCGATACGGGCGAGCTTGAGTATGGTAGGGGAGTCTGGAATTCCTGGTGTAGCGGTGGAATGCGCAGATATCAGGAGGAACACCAATGGCGAAGGCAGGACTCTGGGCCATTACTGACACTGAGGAGCGAAAGCGTGGGGAGCGAACAGG
## 7                                                            ACGTAGGGTGCGAGCGTTAATCGGAATTACTGGGCGTAAAGCGTGCGCAGGCGGTTTCGTAAGACAGAGGTGAAATCCCCGGGCTCAACCTGGGAACTGCCTTTGTGACTGCGAGGCTAGAGTATGGCAGAGGGGGGTGGAATTCCACGTGTAGCAGTGAAATGCGTAGAGATGTGGAGGAACACCGATGGCGAAGGCAGCCCCCTGGGCCAATACTGACGCTCATGCACGAAAGCGTGGGGAGCAAACAGG
## 8                                                            ACGTAGGGTGCAAGCGTTAATCGGAATTACTGGGCGTAAAGCGTGCGCAGGCGGTTATGTAAGACAGAGGTGAAATCCCCGGGCTCAACCTGGGAACTGCCTTTGTGACTGCATAGCTGGAGTGCGGCAGAGGGGGATGGAATTCCGCGTGTAGCAGTGAAATGCGTAGATATGCGGAGGAACACCGATGGCGAAGGCAATCCCCTGGGCCTGCACTGACGCTCATGCACGAAAGCGTGGGGAGCAAACAGG
## 9                                                            ACGAGTGCCTCAAGCGTTACCCGGAATCACTGGGCGTAAAGGTTGTGTAGGTGGTAACATTAGTCTTTTGTGAAAGCCCGCGGCTCAACCGCGGATCCGCAAAGGAAACGGTGTAACTAGAGGATGCAAGAGGTGTGCGGAACTCATAGTGTAGCGGTGAAATGCGTTGATATTATGGGGAACACCAAAAGCGAAGGCAGCACACTGGTGCACTCCTGACACTGAAACAAGAAAGCGTAGGTAGCGAATGGG
## 10                                                           ACGTAGGGTGCAAGCGTTAATCGGAATTACTGGGCGTAAAGCGTGCGCAGGCGGTTATGCAAGACAGAGGTGAAATCCCCGGGCTCAACCTGGGAACTGCCTTTGTGACTGCATAGCTAGAGTACGGCAGAGGGGGATGGAATTCCGCGTGTAGCAGTGAAATGCGTAGATATGCGGAGGAACACCGATGGCGAAGGCAATCCCCTGGGCCTGTACTGACGCTCATGCACGAAAGCGTGGGGAGCAAACAGG
## 11                                                           ACGTAGGGTGCAAGCGTTAATCGGAATTACTGGGCGTAAAGCGTGCGCAGGCGGCTTTGCAAGACAGATGTGAAATCCCCGGGCTCAACCTGGGAACTGCATTTGTGACTGCAAGGCTAGAGTACGGTAGAGGGGGATGGAATTCCGCGTGTAGCAGTGAAATGCGTAGATATGCGGAGGAACACCAATGGCGAAGGCAATCCCCTGGACCTGTACTGACGCTCATGCACGAAAGCGTGGGGAGCAAACAGG
## 12                                                           ACGTAGGGTGCAAGCGTTAATCGGAATTACTGGGCGTAAAGCGTGCGCAGGCGGTTATGTAAGACAGAGGTGAAATCCCCGGGCTCAACCTGGGAACTGCCTTTGTGACTGCATAGCTAGAGTACGGCAGAGGGGGATGGAATTCCGCGTGTAGCAGTGAAATGCGTAGATATGCGGAGGAACACCGATGGCGAAGGCAATCCCCTGGGCCTGTACTGACGCTCATGCACGAAAGCGTGGGGAGCAAACAGG
## 13                                                           ACGTAGGGTGCAAGCGTTAATCGGAATTACTGGGCGTAAAGCGTGCGCAGGCGGTTATGTAAGACAGAGGTGAAATCCCCGGGCTCAACCTGGGAACTGCCTTTGTGACTGCATAGCTAGAGTACGGTAGAGGGGGATGGAATTCCGCGTGTAGCAGTGAAATGCGTAGATATGCGGAGGAACACCGATGGCGAAGGCAATCCCCTGGACCTGTACTGACGCTCATGCACGAAAGCGTGGGGAGCAAACAGG
## 14                                                           ACATAGGGTGCAAGCGTTGTCCGGAATTATTGGGCGTAAAGAGCTCGTAGGTCGTTTGTTACGTCGGATGTGAAAACCTGAGGCTCAACCTCAGGCCTGCATTCGATACGGGCAAACTAGAGTTTGGTAGGGGAGACTGGAATTCCTGGTGTAGCGGTGGAATGCGCAGATATCAGGAGGAACACCAATGGCGAAGGCAGGTCTCTGGGCCAATACTGACACTGAGGAGCGAAAGTCTGGGGAGCGAACAGG
## 15                                                           ACGTAGGGTGCGAGCGTTAATCGGAATTACTGGGCGTAAAGCGTGCGCAGGCGGTTATACAAGACAGGCGTGAAATCCCCGGGCTTAACCTGGGAATGGCGTCTGTGACTGTATAGCTAGAGTGTGTCAGAGGGGGGTGGAATTCCACGTGTAGCAGTGAAATGCGTAGATATGTGGAGGAACACCAATGGCGAAGGCAGCCCCCTGGGATAACACTGACGCTCATGCACGAAAGCGTGGGGAGCAAACAGG
## 16                                                           ACGGAGGGTGCAAGCGTTGTCCGGATTTATTGGGTTTAAAGGGTGCGCAGGTGGTTTATTAAGTCAGTGGTGAAAGACGGTCGCTCAACGATTGCAGTGCCATTGAAACTGGTAGACTTGAGTAAAGTAGAGGTGGGCGGAATTGATAGTGTAGCGGTGAAATGCATAGATATTATCAAGAACTCCAATTGCGTAGGCAGCTCACTTGGCTTTTACTGACACTCATGCACGAAAGTGTGGGTATCAAACAGG
## 17                                                           ACGGAGGGTGCAAGCGTTATCCGGATTCACTGGGTTTAAAGGGTGCGTAGGCGGGTAGGTAAGTCAGTGGTGAAATCCTGGAGCTCAACTCCAGAACTGCCATTGATACTATCTATCTTGAATATTGTGGAGGTAAGCGGAATATGTCATGTAGCGGTGAAATGCTTAGATATGACATAGAACACCCATTGCGAAGGCAGCTTACTACGCATATATTGACGCTGAGGCACGAAAGCGTGGGGATCAAACAGG
## 18                                                           ACGTAGGGTGCAAGCGTTAATCGGAATTACTGGGCGTAAAGCGTGCGCAGGCGGTTATATAAGACAGATGTGAAATCCCCGGGCTCAACCTGGGAACTGCATTTGTGACTGTATAGCTAGAGTACGGTAGAGGGGGATGGAATTCCGCGTGTAGCAGTGAAATGCGTAGATATGCGGAGGAACACCGATGGCGAAGGCAATCCCCTGGACCTGTACTGACGCTCATGCACGAAAGCGTGGGGAGCAAACAGG
## 19                                                           ACGTAGGGTGCAAGCGTTAATCGGAATTACTGGGCGTAAAGCGTGCGCAGGCGGTGATGTAAGACAGATGTGAAATCCCCGGGCTCAACCTGGGAACTGCATTTGTGACTGCATCGCTGGAGTGCGGCAGAGGGGGATGGAATTCCGCGTGTAGCAGTGAAATGCGTAGATATGCGGAGGAACACCGATGGCGAAGGCAATCCCCTGGGCCTGCACTGACGCTCATGCACGAAAGCGTGGGGAGCAAACAGG
## 20                                                           ACGTAGGGTGCGAGCGTTAATCGGAATTACTGGGCGTAAAGCGTGCGCAGGCGGTTTCGTAAGACAGACGTGAAATCCCCGGGCTCAACCTGGGAACTGCGTTTGTGACTGCGAGGCTAGAGTATGGCAGAGGGGGGTGGAATTCCACGTGTAGCAGTGAAATGCGTAGAGATGTGGAGGAACACCGATGGCGAAGGCAGCCCCCTGGGCCAATACTGACGCTCATGCACGAAAGCGTGGGGAGCAAACAGG
## 21                                                           ACGAAGGGTGCTAGCGTTATTCGGAATCACTGGGCGTAAAGAGCGTGTAGGCGGTTTGTTAAGTCAACTGTTAAAGCACCCAGCTTAACTGGGTAAAGGCAGGTGAAACTGGCAGGCTAGAGTGCAAGAGAGAGAAGTGGAATTGTCGGAGTAGCGGTAAAATGCGTAGATCTCGACAGGAACACCGGTTGCGAAGGCGGCTTCTTGGCTTGTAACTGACGCTAAAGCGCGAAAGCGTGGGGAGCAAACAGG
## 22                                                           ACATAGGGTGCAAGCGTTGTCCGGAATTATTGGGCGTAAAGAGCTCGTAGGTCGTTTGTCGCGTCGATTGTGAAAATCTGAGGCTCAACCTCAGACCTGCAGTCGATACGGGCAAACTAGAGTGTGGTAGGGGAGACTGGAATTCCTGGTGTAGCGGTGGAATGCGCAGATATCAGGAGGAACACCAATGGCGAAGGCAGGTCTCTGGGCCATAACTGACACTGAGGAGCGAAAGTGCGGGGAGCGAACAGG
## 23                                                           ACGTAGGGTGCGAGCGTTAATCGGAATTACTGGGCGTAAAGCGTGCGCAGGCGGTTTTGTAAGTCGGATGTGAAATCCCCGGGCTCAACCTGGGAACTGCGTTCGAAACTGCAAGGCTAGAGTGTGTCAGAGGGGGGTAGAATTCCACGTGTAGCAGTGAAATGCGTAGAGATGTGGAGGAATACCAATGGCGAAGGCAGCCCCCTGGGATAACACTGACGCTCATGCACGAAAGCGTGGGGAGCAAACAGG
## 24                                                           ACGTAGGGTGCAAGCGTTAATCGGAATTACTGGGCGTAAAGCGTGCGCAGGCGGTTATATAAGTCAGATGTGAAATCCCCGGGCTCAACCTGGGAACTGCATTTGAGACTGTATAGCTAGAGTACGGCAGAGGGGGATGGAATTCCGCGTGTAGCAGTGAAATGCGTAGATATGCGGAGGAACACCGATGGCGAAGGCAATCCCCTGGGCCTGTACTGACGCTCATGCACGAAAGCGTGGGGAGCAAACAGG
## 25                                                           ACGTAGGGTGCAAGCGTTAATCGGAATTACTGGGCGTAAAGCGTGCGCAGGCGGATGTTTAAGCCAGATGTGAAAGCCCCGAGCTTAACTTGGGAATTGCGTTTGGAACTGAGCATCTAGAGTCTGTCAGAGGGGGATGGAATTCCACGTGTAGCAGTGAAATGCGTAGAGATGTGGAGGAACACCGATGGCGAAGGCAGTCCCCTGGGATAAGACTGACGCTCATGCACGAAAGCGTGGGGAGCAAACAGG
## 26                                                           ACGTAGGGTGCAAGCGTTAATCGGAATTACTGGGCGTAAAGCGTGCGCAGGCGGTTCGGAAAGAAAGGTGTGAAATCCCAGGGCTTAACCTTGGAACTGCACTTTTAACTACCGAGCTAGAGTATGTCAGAGGGGGGTGGAATTCCGCGTGTAGCAGTGAAATGCGTAGATATGCGGAGGAACACCGATGGCGAAGGCAGCCCCCTGGGATAATACTGACGCTCATGCACGAAAGCGTGGGGAGCAAACAGG
## 27                                                           ACAGAGGGTGCAAGCGTTAATCGGAATTACTGGGCGTAAAGCGTGCGTAGACGGTTATCTAAGTCGGATGTGAAATCCCCGGGCTCAACCTGGGAATTGCATTCGAGACTGAATAGCTAGGGTGCGGAAGAGGGAAGCGGAATTTCCGGTGTAGCGGTGAAATGCGTAGATATCGGAAGGAACATCAGTGGCGAAAGCGGCTTCCTGGTCCAGCACCGACGTTCAGGCACGAAAGCGTGGGGAGCAAACAGG
## 28                                                           ACATAGGGTGCAAGCGTTGTCCGGAATTATTGGGCGTAAAGAGCTCGTAGGTGGTTCGATACGTCGGATGTGAAAATCAGGGGCTCAACCCCTGACCTGCATCCGATACGGTCGAGCTAGAGTTTGGTAGGGGAGACTGGAATTCCTGGTGTAGCGGTGGAATGCGCAGATATCAGGAGGAACACCGATGGCGAAGGCAGGTCTCTGGGCCAATACTGACACTGAGGAGCGAAAGCGTGGGGAGCGAACAGG
## 29                                                           ACGGAGGATGCAAGTGTTATCCGGAATCACTGGGCGTAAAGCGTCTGTAGGTGGTTTAATAAGTCAACTGTTAAATCTTGAGGCTCAACTTCAAAATCGCAGCTGAAACTATTAGACTAGAGTATAGTAGAGGTAAAGGGAATTTCCAGTGGAGCGGTGAAATGCGTAGATATTGGAAAGAACACCGATGGCGAAAGCACTTTACTGGGCTATTACTAACACTCAGAGACGAAAGCTAGGGTAGCAAATGGG
## 30                                                           ACGTAGGGTGCGAGCGTTAATCGGAATTACTGGGCGTAAAGCGTGCGCAGGCGGTTATACAAGACAGGCGTGAAATCCCCGGGCTTAACCTGGGAATGGCGCCTGTGACTGTATAGCTAGAGTGTGTCAGAGGGGGGTAGAATTCCACGTGTAGCAGTGAAATGCGTAGATATGTGGAGGAATACCAATGGCGAAGGCAGCCCCCTGGGATAACACTGACGCTCATGCACGAAAGCGTGGGGAGCAAACAGG
## 31                                                           ACGGAGGATCCAAGCGTTATCCGGATTTATTGGGTTTAAAGGGTCCGTAGGCGGGTCTTTAAGTCAGTGGTGAAAGCCCACAGCTCAACTGTGGAACTGCCATTGATACTGGAGATCTTGAGTGTAGTAGAAGTAGGCGGAATAGGGCATGTAGCGGTGAAATGCATAGATATGCCCTAGAACACCGATTGCGAAGGCAGCTTACTATGTTACAACTGACGCTGAGGGACGAAAGCGTGGGGAGCAAACAGG
## 32                                                           ACGGAGGATCCAAGCGTTATCCGGATTTATTGGGTTTAAAGGGAGCGTAGGTTGACATATAAGTCAGCTGTGAAAGTTTACGGCTCAACCGTGAAATTGCAGTTGATACTGTATGTCTTGAGTGTACAAGAGGTGGGCGGAATTCGTGGTGTAGCGGTGAAATGCTTAGATATCACGAAGAACTCCAATTGCGAAGGCAGCTCACTGGGGTACAACTGACACTGAGGCTCGAAAGTGTGGGTATCAAACAGG
## 33                                                           ACGGAGGGTGCAAGCGTTATCCGGATTCACTGGGTTTAAAGGGTGCGTAGGCGGGCAGGTAAGTCAGTGGTGAAATCCCCGAGCTTAACTCGGGAACTGCCGTTGATACTATCTGTCTTGAATACTGTGGAGGTAAGCGGAATATGTCATGTAGCGGTGAAATGCTTAGATATGACATAGAACACCAATTGCGAAGGCAGCTTGCTACACGGTCATTGACGCTGAGGCACGAAAGCGTGGGGATCAAACAGG
## 34                                                           ACGGAGGGTGCAAGCGTTAATCGGAATTACTGGGCGTAAAGCGCACGCAGGCGGTTGGATAAGTTAGATGTGAAAGCCCCGGGCTCAACCTGGGAATTGCATTTAAAACTGTCCAGCTAGAGTCTTGTAGAGGGGGGTAGAATTCCAGGTGTAGCGGTGAAATGCGTAGAGATCTGGAGGAATACCGGTGGCGAAGGCGGCCCCCTGGACAAAGACTGACGCTCAGGTGCGAAAGCGTGGGGAGCAAACAGG
## 35                                                           ACAGAGGATGCAAGCGTTATCCGGAATCACTGGGCATAAAGCGTCTGTAGGTGGTTTGGTAAGTCTGCTGTTAAAGACTGGGGCTCAACCCCAGAAAAGCAGTGGAAACTGCCAGACTTGAGTGTGGTAGAGGTAAAGGGAATTCCTAGTGTAGCGGTGAAATGCGTAGATATTAGGAAGAACACCAATGGCGAAGGCACTTTACTGGGCCATAACTGACACTGAGAGACGACAGCTAGGGGAGCAAATGGG
## 36                                                           ACGGAGGGTGCAAGCGTTAATCGGAATAACTGGGCGTAAAGCGCACGCAGGCGGTTAGATAAGTCAGATGTGAAATCCCCGGGCTCAACCTGGGAACTGCATTTGAAACTGTCTGACTAGAGTCTTGTAGAGGGGGGTAGAATTCCAGGTGTAGCGGTGAAATGCGTAGAGATCTGGAGGAATACCGGTGGCGAAGGCGGCCCCCTGGACAAAGACTGACGCTCAGGTGCGAAAGCGTGGGGAGCAAACAGG
## 37                                                           ACGTAGGGTGCAAGCGTTAATCGGAATTACTGGGCGTAAAGCGTGCGCAGGCGGTTATATAAGACAGATGTGAAATCCCCGGGCTCAACCTGGGAACTGCATTTGTGACTGTATAGCTAGAGTACGGCAGAGGGGGATGGAATTCCGCGTGTAGCAGTGAAATGCGTAGATATGCGGAGGAACACCGATGGCGAAGGCAATCCCCTGGGCCTGTACTGACGCTCATGCACGAAAGCGTGGGGAGCAAACAGG
## 38                                                           ACGGAGGGTGCAAGCGTTAATCGGAATAACTGGGCGTAAAGCGCACGCAGGCGGTTAGATAAGTCAGATGTGAAAGCCCCGGGCTCAACCTGGGAACTGCATTTGAAACTGTCTGACTAGAGTCTTGTAGAGGGGGGTAGAATTCCAGGTGTAGCGGTGAAATGCGTAGAGATCTGGAGGAATACCGGTGGCGAAGGCGGCCCCCTGGACAAAGACTGACGCTCAGGTGCGAAAGCGTGGGGAGCAAACAGG
## 39                                                           ACATAGGGTGCAAGCGTTGTCCGGAATTATTGGGCGTAAAGAGCTCGTAGGTGGTTCGATACGTCGGATGTGAAAATCAGGGGCTCAACCCCTGACCTGCATTCGATACGGTCGAGCTAGAGTTTGGTAGGGGAGACTGGAATTCCTGGTGTAGCGGTGGAATGCGCAGATATCAGGAGGAACACCGATGGCGAAGGCAGGTCTCTGGGCCAATACTGACACTGAGGAGCGAAAGCGTGGGGAGCGAACAGG
## 40                                                           ACGGAGGGTCCAAGCGTTAATCGGAATTACTGGGCGTAAAGCGCGCGTAGGCGGTTAATTAAGCGAGATGTGAAATCCCTGGGCTCAACCTAGGAATTGCGTTTCGAACTGATTAGCTAGAGTGCAGTAGAGGGTGGTGGAATTTCCGGTGTAGCGGTGAAATGCGTAGAGATCGGAAGGAACATCAGTGGCGAAGGCGACTGCCTGGACTGACACTGACGCTGAGGTGCGAAAGCGTGGGGAGCAAACAGG
## 41                                                           ACGTAGGGAGCAAGCGTTGTCCGGAATTACTGGGTGTAAAGGGAGCGTAGGCGGGACGGCAAGTCAGATGTGAAATTTATGGGCTCAACTCATAACCTGCATTTGAAACTGCTGTTCTTGAGTGAAGTAGAGGTAAGCGGAATTCCTGGTGTAGCGGTGAAATGCGTAGATATCAGGAGGAACATCGGTGGCGAAGGCGGCTTACTGGGCTTTTACTGACGCTGAGGCTCGAAAGCGTGGGGAGCAAACAGG
## 42                                                           ACGGAGGGTGCAAGCGTTATCCGGATTCACTGGGTTTAAAGGGTGCGTAGGCGGGCAGGTAAGTCAGTGGTGAAATCCTGGAGCTCAACTCCAGAACTGCCATTGATACTATCTGTCTTGAATATTGTGGAGGTAAGCGGAATATGTCATGTAGCGGTGAAATGCTTAGATATGACATAGAACACCCATTGCGAAGGCAGCTTACTACGCATATATTGACGCTGAGGCACGAAAGCGTGGGGATCAAACAGG
## 43                                                           ACGTAGGATCCGAGCGTTGTCCGGAGTTACTGGGTGTAAAGGGTGCGTAGGCTGTTCTGTGCGTCAGAGGTGAAATCCACGGGCTTAACTCGTGGGGTGCCTTTGATACGGCAGGGCTTGAGTACGAGAGAGGTGCGTGGAATTCCTGGTGTAGCAGTGAAATGCGTAGATATCAGGAGGAACACCGGTGGCGAAGGCGGCGCACTGGCTCGTAACTGACGCTGAGGCACGAAAGCGTGGGGATCAAACAGG
## 44                                                           ACGAAAGGTGCAAGCGTTGTTCGGATTGACTGGGCGTAAAGAGCACGTAGGTGGGTCTTTAAGTCGATTGTGAAATCCCGGAGCTCAACTTCGGACTGGCAATCGAAACTGTAGATCTTGAATACCCAAGGGGTTGGTGGAATTGCACATGTAGCAGTGAAATGCGTAGATATGTGCAGGAACACCGGAGGCGAAGGCGGCCAACTGGTGGTGTATTGACGCTGAGGTGCGAAAGCGCGGGGAGCAAACAGG
## 45                                                           ACGGAGGATCCGAGCGTTATCCGGATTTATTGGGTTTAAAGGGTGCGTAGGTGGTTTGATAAGTCAGCGGTGAAAGTTTGCAGCTTAACTGTAAAAATGCCGTTGAAACTGTCGGACTTGAGTGTAAATGAGGTAGGCGGAATGCGTGGTGTAGCGGTGAAATGCATAGATATCACGCAGAACTCCGATTGCGAAGGCAGCTTACTAAGCTACAACTGACACTGAAGCACGAAAGCGTGGGGATCAAACAGG
## 46                                                           ACGTAGGGTGCAAGCGTTAATCGGAATTACTGGGCGTAAAGCGTGCGCAGGCGGTTATGTAAGACAGGCGTGAAATCCCCGGGCTTAACCTGGGAATTGCGCTTGTGACTGCATAGCTAGAGTATGTCAGAGGGGGGTAGAATTCCACGTGTAGCAGTGAAATGCGTAGAGATGTGGAGGAATACCAATGGCGAAGGCAGCCCCCTGGGATAATACTGACGCTCATGCACGAAAGCGTGGGGAGCAAACAGG
## 47                                                           ACGGAGGGAGCTAGCGTTGTTCGGAATCACTGGGCGTAAAGCGTGCGTAGGCGGTGACTCAAGTCAGAGGTGAAAGCCTGGAGCTCAACTCCAGAACTGCCTTTGAAACTAGGTCGCTAGAACATCGGAGAGGTGAGTGGAATTCCGAGTGTAGAGGTGAAATTCGTAGATATTCGGAAGAACACCAGTGGCGAAGGCGGCTCACTGGACGATTGTTGACGCTGAGGCACGAAAGCGTGGGGAGCAAACAGG
## 48                                                           ACGTAGGGTGCAAGCGTTAATCGGAATTACTGGGCGTAAAGCGTGCGCAGGCGGTTGTGCAAGACAGGTGTGAAATCCCCGGGCTTAACCTGGGAACTGCACTTGTGACTGCACGGCTTGAGTGCGGCAGAGGGGGATGGAATTCCGCGTGTAGCAGTGAAATGCGTAGATATGCGGAGGAACACCGATGGCGAAGGCAATCCCCTGGGCCTGCACTGACGCTCATGCACGAAAGCGTGGGGAGCAAACAGG
## 49                                                           ACGAGTGCCCCGAGCGTTATCCGGAATCATTGGGCGTAAAGGGTGTGTAGGTGGTCTTGTTAGTCAATTGTTAAAACCCGGGGCTTAACCCCGGAAGTGCGATTGAAACGGCAAAACTAGAGGATGGCAGGGATGTATGGAACTCATGGAGTAGGGGTGAAATCCGTTGATATCATGGGGAACACCAAAAGCGAAGGCAGTACATTGGGCCACTCCTGACACTGAAACACGAAAGCGTGGGTAGCGAATGGG
## 50                                                           ACGTAGGGTGCAAGCGTTAATCGGAATTACTGGGCGTAAAGCGTGCGCAGGCGGTTATGCAAGACAGGTGTGAAATCCCCGAGCTCAACTTGGGAACTGCACTTGTGACTGCATAGCTAGAGTACGGTAGAGGGGAGTGGAATTCCGCGTGTAGCAGTGAAATGCGTAGATATGCGGAGGAACACCGATGGCGAAGGCAGCTCCCTGGACCTGTACTGACGCTCATGCACGAAAGCGTGGGGAGCAAACAGG
## 51                                                           ACGTAGGGTGCGAGCGTTAATCGGAATTACTGGGCGTAAAGCGTGCGCAGGCGGTTATGTAAGACAGATGTGAAATCCCCGGGCTCAACCTGGGAACTGCGTTTGTGACTGCATAACTAGAGTACGGCAGAGGGAGGTGGAATTCCGCGTGTAGCAGTGAAATGCGTAGATATGCGGAGGAACACCGATGGCGAAGGCAGCCTCCTGGGCCAGTACTGACGCTCATGCACGAAAGCGTGGGGAGCAAACAGG
## 52                                                           ACGGAGGGTGCAAGCGTTAATCGGAATTACTGGGCGTAAAGCGCGCGTAGGCGGCTAGTTAAGCTAGATGTGAAATCCCTGGGCTCAACCTGGGAACTGCATTTAGAACTGACTGGCTAGAGTGCAGAAGAGGAGTGTGGAATTTCCGGTGTAGCGGTGAAATGCGTAGAGATCGGAAGGAACATCAGTGGCGAAGGCGACACTCTGGTCTGACACTGACGCTGAGGTGCGAAAGCGTGGGTAGCAAACAGG
## 53                                                           ACGTAGGGTGCAAGCGTTAATCGGAATTACTGGGCGTAAAGCGTGCGCAGGCGGTTTTATAAGACAGAGGTGAAATCCCCGGGCTCAACCTGGGAACTGCCTTTGTGACTGTAAGGCTAGAGTGTGTCAGAGGGGGGTAGAATTCCACGTGTAGCAGTGAAATGCGTAGAGATGTGGAGGAATACCGATGGCGAAGGCAGCCCCCTGGGATAACACTGACGCTCATGCACGAAAGCGTGGGGAGCAAACAGG
## 54                                                           ACGGAGGGTGCAAGCGTTGTCCGGATTTATTGGGTTTAAAGGGTGCGTAGGCGGCTGAATAAGTCAGCGGTGAAAGACTTCGGCTTAACCGGAGCAGTGCCGTTGATACTGTTTGGCTTGAGTGTTGGAGGGGTACATGGAATTGATGGTGTAGCGGTGAAATGCATAGATACCATCAGGAACACCGATAGCGAAGGCATTGTACTGGCCAACAACTGACGCTGAGGCACGAAAGTGTGGGGATCGAACAGG
## 55                                                           ACGGAGGATGCAAGTGTTATCCGGAATCACTGGGCGTAAAGCGTCTGTAGGTGGTTTGATAAGTCAACTGTTAAATCTTGAGGCTCAACTTCAAAATCGCAGTCGAAACTATTAGACTAGAGTATAGTAGAGGTAAAGGGAATTTCCAGTGGAGCGGTGAAATGCGTAGATATTGGAAAGAACACCGATGGCGAAAGCACTTTACTGGGCTATTACTAACACTCAGAGACGAAAGCTAGGGTAGCAAATGGG
## 56                                                           ACGTAGGGTGCAAGCGTTAATCGGAATTACTGGGCGTAAAGCGTGCGCAGGCGGTTGATTAAGTTTGATGTGAAATCCCCGGGCTTAACCTGGGAACTGCATTGAATACTGGTCAGCTAGAGTGTGTCAGAGGGGGGTAGAATTCCACGTGTAGCAGTGAAATGCGTAGAGATGTGGAGGAATACCGATGGCGAAGGCAGCCCCCTGGGATAACACTGACGCTCATGCACGAAAGCGTGGGGAGCAAACAGG
## 57                                                           ACGGAGGGTGCAAGCGTTATCCGGATTCACTGGGTTTAAAGGGTGCGTAGGCGGGCAGGTAAGTCAGTGGTGAAATCCTGGAGCTCAACTCCAGAACTGCCATTGATACTATCTGTCTTGAATATTGTGGAGGTAAGCGGAATATGTCATGTAGCGGTGAAATGCTTAGATATGACATAGAACACCTATTGCGAAGGCAGCTTACTACGCATATATTGACGCTGAGGCACGAAAGCGTGGGGATCAAACAGG
## 58                                                           ACGTAGGGTGCGAGCGTTAATCGGAATTACTGGGCGTAAAGCGTGCGCAGGCGGTTGTGCAAGACAGATGTGAAATCCCCGGGCTTAACCTGGGAATTGCATTTGTGACTGCACGGCTAGAGTGTGTCAGAGGGGGGTAGAATTCCACGTGTAGCAGTGAAATGCGTAGATATGTGGAGGAATACCGATGGCGAAGGCAGCCCCCTGGGATAACACTGACGCTCATGCACGAAAGCGTGGGGAGCAAACAGG
## 59                                                           ACATAGGGTGCAAGCGTTGTCCGGAATTATTGGGCGTAAAGAGCTCGTAGGTTGTTTGTTACGTCGGATGTGAAAACCTGAGGCTCAACCTCAGGCCTGCATTCGATACGGGCAAACTAGAGTTTGGTAGGGGAGACTGGAATTCCTGGTGTAGCGGTGGAATGCGCAGATATCAGGAGGAACACCAATGGCGAAGGCAGGTCTCTGGGCCAATACTGACACTGAGGAGCGAAAGTCTGGGGAGCGAACAGG
## 60                                                           ACGAAGGTGGCAAGCGTTGTTCGGATTCACTGGGCGTACAGGGTGTGTAGGCGGTTTGGTAAGCCTTCTGTTAAAGCTTCGGGCCCAACCCGGAAAGCGCAGAGGGTACTGCCAGGCTAGAGGGTGGGAGAGGAGCGCGGAATTCCCGGTGTAGCGGTGAAATGCGTAGAGATCGGGAGGAAGGCCGGTGGCGAAGGCGGCGCTCTGGAACATACCTGACGCTGAGACACGAAAGCGTGGGGAGCAAACAGG
## 61                                                           ACGTAGGGTGCGAGCGTTAATCGGAATTACTGGGCGTAAAGCGTGCGCAGGCGGTTTTGTAAGACAGATGTGAAATCCCCGGGCTTAACCTGGGAACTGCATTTGTGACTGCAAGACTAGAGTGTGTCAGAGGGAGGTGGAATTCCGCGTGTAGCAGTGAAATGCGTAGAGATGCGGAAGAACACCGATGGCGAAGGCAGCCTCCTGGGATAACACTGACGCTCAGGCACGAAAGCGTGGGGAGCAAACAGG
## 62                                                           ACAGAGGATGCAAGCGTTATCCGGAATCACTGGGCATAAAGCGTCTGTAGGTTGCTTGCCAAGTCTGCTGTTAAAGACTAGGGCCTAACCCTGGGAAAGCAGTGGAAACTAGCAGGCTTGAGTGTGGTAGAGGTAGAGGGAATTCCTGGTGTAGCGGTGAAATGCGTAGATATTAGGAAGAACACCAATGGCGAAAGCACTCTGCTGGGCCACAACTGACACTGAGAGACGACAGCTAGGGGAGCAAATGGG
## 63                                                           ACATAGGGTGCAAGCGTTGTCCGGAATTATTGGGCGTAAAGAGCTCGTAGGTGGTTCGATACGTCGGATGTGAAAATCAGGGGCTCAACCCCTGACCTGCATTCGATACGGTCGAGCTAGAGTGTGGTAGGGGAGACTGGAATTCCTGGTGTAGCGGTGGAATGCGCAGATATCAGGAGGAACACCGATGGCGAAGGCAGGTCTCTGGGCCATTACTGACACTGAGGAGCGAAAGCGTGGGGAGCGAACAGG
## 64                                                           ACGTAGGGTGCGAGCGTTAATCGGAATTACTGGGCGTAAAGCGTGCGCAGGCGGTTTTGTAAGACAGGCGTGAAATCCCCGGGCTCAACCTGGGAACTGCGCTTGTGACTGCAAGGCTAGAGTACGGCAGAGGGGGGTGGAATTCCACGTGTAGCAGTGAAATGCGTAGAGATGTGGAGGAACACCAATGGCGAAGGCAGCCCCCTGGGTCGATACTGACGCTCATGCACGAAAGCGTGGGTAGCAAACAGG
## 65                                                          ACGGAGGATCCAAGCGTTATCCGGAATTACTGGGCGTAAAGAGTTGCGTAGGTGGCATTGTAAGTCTGTAGTGAAAGCGTGGGGCTCAACCCCATACACATTATGGAAACTGCAAAGCTAGAGGACAAGAGAGGTTATTGGAATTCCTAGTGTAGGAGTGAAATCCGTAGATATTAGGAGGAACACCGATGGCGTAGGCAGATAACTGGCTTGTTCCTGACACTAAGGCACGAAAGCATGGGTAGCAAACGGG
## 66                                                           ACGGAGGGTGCAAGCGTTATCCGGAATCATTGGGTTTAAAGGGTCCGCAGGCGGTCTGATAAGTCAGTGGTGAAAGCCTATTGCTCAACAATAGAACTGCCATTGATACTGTTAGACTTGAATTAGGTCGGAGTGGGCGGAATATGACATGTAGCGGTGAAATGCTTAGATATGTCATAGAACACCGATAGCGAAGGCAGCTCACTAGCCCTAAATTGACGCTCATGGACGAAAGCGTGGGGAGCAAACAGG
## 67                                                          ACGAGTGCTTCAAGCGTTATCCGGAATCATTGGGCGTAAAGGGTGTGTAGGCGGTTATGTTAGTCTCTCGTCAAATCTTTCGGCTCAACCGAGAGTCCGCGAGGGAAACGGCATGACTAAGAGGTTGGAAGAGGTCTCTGGAACTCATGGTGTAGCGGTGAAATGCGTTGATATCATGGGGAACACCAAAAGCGAAGGCAAGAGACTGGTCCATACCTGACGCTGAAACACGAAAGCGTGGGTCGCGAATGGG
## 68                                                           ACGGAGGATGCAAGTGTTATCCGGAATCACTGGGCGTAAAGCGTCTGTAGGTGGTTTAATAAGTCAACTGTTAAATCTTGAGGCTTAACTTCAAAATCGCAGTCGAAACTATTAGACTAGAGTATAGTAGGGGTAAAGGGAATTTCCAGTGGAGCGGTGAAATGCGTAGATATTGGAAAGAACACCGATGGCGAAAGCACTTTACTGGGCTATTACTAACACTCAGAGACGAAAGCTAGGGTAGCAAATGGG
## 69                                                           ACGTAGGGTGCAAGCGTTAATCGGAATTACTGGGCGTAAAGCGTGCGCAGGCGGTTATGTAAGACAGATGTGAAATCCCCGGGCTCAACCTGGGAACTGCATTTGTGACTGCATAGCTAGAGTACGGTAGAGGGGGATGGAATTCCGCGTGTAGCAGTGAAATGCGTAGATATGCGGAGGAACACCGATGGCGAAGGCAATCCCCTGGACCTGTACTGACGCTCATGCACGAAAGCGTGGGGAGCAAACAGG
## 70                                                           ACGGAGGGTGCAAGCGTTATCCGGATTCACTGGGTTTAAAGGGTGCGTAGGCGGGCTAGTAAGTCAGTGGTGAAATCCCCGAGCTTAACTTGGGAACTGCCGTTGATACTATTAGTCTTGAATATCGTGGAGGTAAGCGGAATATGTCATGTAGCGGTGAAATGCTTAGATATGACATAGAACACCAATTGCGAAGGCAGCTTGCTACACGATTATTGACGCTGAGGCACGAAAGCGTGGGGATCAAACAGG
## 71                                                           ACGTAGGGTGCGAGCGTTAATCGGAATTACTGGGCGTAAAGCGTGCGCAGGCGGTTACATAAGACAGATGTGAAATCCCCGGGCTTAACCTGGGAACTGCGTTTGTGACTGTGTGACTAGAGTACGGTAGAGGGGGGTGGAATTCCTGGTGTAGCAGTGAAATGCGTAGATATCAGGAGGAACATCGATGGCGAAGGCAGCCCCCTGGACCTGTACTGACGCTCATGCACGAAAGCGTGGGGAGCAAACAGG
## 72                                                           ACGTAGGGTGCAAGCGTTAATCGGAATTACTGGGCGTAAAGCGTGCGCAGGCGGTTTCGTAAGACAGAGGTGAAATCCCCGGGCTCAACCTGGGAACTGCCTTTGTGACTGCGAGGCTAGAGTATGGCAGAGGGGGGTGGAATTCCACGTGTAGCAGTGAAATGCGTAGAGATGTGGAGGAACACCGATGGCGAAGGCAGCCCCCTGGGCCAATACTGACGCTCATGCACGAAAGCGTGGGGAGCAAACAGG
## 73                                                           ACGTAGGGTGCGAGCGTTAATCGGAATTACTGGGCGTAAAGCGTGCGCAGGCGGTTTTGTAAGTCAGATGTGAAATCCCCGGGCTCAACCTGGGAACTGCGTTTGAAACTACAAGGCTAGAGTATGGTAGAGGGGGGTAGAATTCCACGTGTAGCAGTGAAATGCGTAGAGATGTGGAGGAATACCAATGGCGAAGGCAGCCCCCTGGACTAATACTGACGCTCATGCACGAAAGCGTGGGGAGCAAACAGG
## 74                                                           ACGGAGGGTGCGAGCGTTATCCGGATTCACTGGGTTTAAAGGGTGCGTAGGCGGGCTAGTAAGTCAGTGGTGAAATCCCCGAGCTTAACTTGGGAACTGCCGTTGATACTATTAGTCTTGAATATCGTGGAGGTAAGCGGAATATGTCATGTAGCGGTGAAATGCTTAGATATGACATAGAACACCAATTGCGAAGGCAGCTTGCTACACGATTATTGACGCTGAGGCACGAAAGCGTGGGGATCAAACAGG
## 75                                                           ACGTAGGGTGCAAGCGTTAATCGGAATTACTGGGCGTAAAGCGTGCGCAGGCGGTTATGCAAGACAGAGGTGAAATCCCCGGGCTCAACCTGGGAACTGCCTTTGTGACTGCATGACTAGAGTACGGCAGAGGGGGATGGAATTCCGCGTGTAGCAGTGAAATGCGTAGATATGCGGAGGAACACCGATGGCGAAGGCAATCCCCTGGGCCTGTACTGACGCTCATGCACGAAAGCGTGGGGAGCAAACAGG
## 76                                                           ACAGAGAGGGCAAGCGTTGTCCGGAATGACTGGGCGTAAAGCGCGCGCAGGCGGTTTTTTAAGTTGGATGTGAAATCCCCGGGCTTAACCTGGGAACTGCATACGATACTGGGAAACTGGAGGCCGAGAGAGGAAAGCGGAATGACGAGTGTAGAGGTGAAATTCGTAGATATTCGTCGGAACACCGGAGGCGAAGGCGGCTTTCTGGCTCGGACCTGACGCTGAGGCGCGAAAGCGTGGGGAGCAAACAGG
## 77                                                           ACGTAGGGTGCAAGCGTTAATCGGAATTACTGGGCGTAAAGCGTGCGCAGGCGGTTATGTAAGACAGAGGTGAAATCCCCGGGCTCAACCTGGGAACGGCCTTTGTGACTGCATAGCTAGAGTACGGTAGAGGGGGATGGAATTCCGCGTGTAGCAGTGAAATGCGTAGATATGCGGAGGAACACCGATGGCGAAGGCAATCCCCTGGACCTGTACTGACGCTCATGCACGAAAGCGTGGGGAGCAAACAGG
## 78                                                           ACGTAGGGTGCGAGCGTTAATCGGAATTACTGGGCGTAAAGCGTGCGCAGGCGGTCTTGTAAGACAGATGTGAAATCCCCGGGCTCAACCTGGGACCTGCATTTGTGACTGCAAGGCTGGAGTACGGCAGAGGGGGATGGAATTCCGCGTGTAGCAGTGAAATGCGTAGATATGCGGAGGAACACCGATGGCGAAGGCAATCCCCTGGGCCTGTACTGACGCTCATGCACGAAAGCGTGGGGAGCAAACAGG
## 79                                                           ACGGAGGATCCAAGCGTTATCCGGAATCATTGGGTTTAAAGGGTCCGTAGGCGGTTTATTAAGTCAGTGGTGAAATCTGGTCGCTCAACGATCAAACGGCCATTGATACTGATAGACTTGAATTACTGGGAAGTAACTAGAATATGTAGTGTAGCGGTGAAATGCTTAGATATTACATGGAATACCAATTGCGAAGGCAGGTTACTACCAGTGGATTGACGCTGATGGACGAAAGCGTGGGGAGCGAACAGG
## 80                                                          ACGAACCAAGCGAACGTTATTCGGAATTACTGGGCTTAAAGCGCGTGTAGGCGGACTGCCACGTCGGTTGTTGAAATCCCCCGGCTTAACCGGGGAACAGGCACCGATACGAGTAGTCTTGAGGTGGGTAGGGGAGACTGGAACTTCCGGTGGAGCGGTGAAATGCGTTGAGATCGGAAGGAACGCCCGCGGCGAAAGCGAGTCTCTGGCCCCATACTGACGCTGAGACGCGAAAGCCAGGGGAGCAAACGGG
## 81                                                           ACGGAGGGTGCAAGCGTTATCCGGAATCATTGGGTTTAAAGGGTCCGCAGGCGGATTTATAAGTCAGTGGTGAAATCCTATCGCTTAACGATAGAACTGCCATTGATACTGTAAGTCTTGAATTCGGTCGGAGTGGGCGGAATGTGTAGTGTAGCGGTGAAATGCATAGATATTACACAGAACACCGATAGCGAAGGCAGCTCACTAGGCCTGAATTGACGCTCAGGGACGAAAGCGTGGGGATCAAACAGG
## 82                                                           ACGGAGGATCCGAGCGTTATCCGGATTTATTGGGTTTAAAGGGTGCGTAGGCGGCCTATTAAGTCAGGGGTGAAAACTTGCAGCTTAACTGTAAGCGTGCCTTTGATACTGATGGGCTTGAATTAAGATGAAGTAGGCGGAATGTGACAAGTAGCGGTGAAATGCATAGATATGTCACAGAACACCAATTGCGAAGGCAGCTTACTAAGGTTAAATTGACGCTGAGGCACGAAAGCGTGGGGATCAAACAGG
## 83                                                          ACGTAGGGGGCGAGCGTTGTCCGAAGTTACTGGGCGTAAAGCGCGCGTAGGCGGTTATTCAAGTCTGGGGTGAAAGGTTCAGCGCTCAACGTGAACAGTGCCTTGGATACTGGATGACTTGAGTCTGGAAGGGGGAAGGGGAATTCCTGGTGTAGCGGTGAAATGCGTAGATATCAGGAGGAACACCGGTGGCGAAGGCGCCTTCCTGGTCTAAGACTGACGCTGAGGTGCGAAAGCGTGGGGAGCAAACGGG
## 84                                                           ACGAGTGCCTCAAGCGTTATCCGGAATCATTGGGCGTAAAGGTTGTGTAGGTGGTTATATTAGTCTCTTGTTAAATTCTTCGGCTTAACCGGGGACATGCAAGAGAAACGGTATGACTAGAGGATGCGAGGGGTCTGCGGAATTCATAGTGTAGCGGTAAAATGCGTTGATATTATGGAGAACACCAAAAGCGAAGGCAGCAGACTGGAGCACTCCTGACACTGAAACAAGAAAGCGTGGGTCGCGAATGGG
## 85                                                           ACAGAGACCTCAAGCGTTATCCGGATTTATTGGGCGTAAAGCGTCCGCAGGTGGTTCGTTAAGTCAATGGTCAAATCACGGGGCTCAACCCCGCAACTGCCGTTGATACTGGCGAACTTGAGGCCGGTAGAGGTAAGCGGAATTACAGGTGTAGCGGTAAAATGCGTTTATATCTGTAAGAACACCAAAGGCGAAAGCAGCTTACTGGAACGGTCCTGACACTCATGGACGAAAGCGTGGGGAGCGAAAAGG
## 86                                                           ACATAGGGTGCAAGCGTTGTCCGGAATTATTGGGCGTAAAGAGCTCGTAGGTGGTTCGATACGTCGGATGTGAAAATCAGGGGCTCAACCCCTGACCTGCATTCGATACGGTCGAGCTGGAGTTTGGTAGGGGAGACTGGAATTCCTGGTGTAGCGGTGGAATGCGCAGATATCAGGAGGAACACCGATGGCGAAGGCAGGTCTCTGGGCCAATACTGACACTGAGGAGCGAAAGCGTGGGGAGCGAACAGG
## 87                                                           ACGAGGGGTGCAAACGTTATTCGGAATTACTGGGTGTAAAGGGTACGTAGACGGCATCTTAAGTCAATTGTTAAATTCCCCGGCCTAACTGGGGGTAGGCGATTGAAACTAAGAAGCTAGAGAGTGGAAGAGAGAAGTGGAATTCTCGGAGTAGCGGTAAAATGCGTAGATCTCGAGAGGAACACCGATGGCGAAGGCAGCTTCTTGGTCCATATCTGACGTTGAGGTACGAAAGCGTGGGGAGCAAACAGG
## 88                                                           ACGGAGGGTGCAAGCGTTGTCCGGATTTATTGGGTTTAAAGGGTGCGTAGGCGGCTGAATAAGTCAGCGGTGAAAGACTTCGGCTTAACCGGAGCAGTGCCGTTGATACTGTTTAGCTTGAGTGTTGGAGGGGTACATGGAATTGATGGTGTAGCGGTGAAATGCATAGATACCATCAGGAACACCGATAGCGAAGGCATTGTACTGGCCAACAACTGACGCTGAGGCACGAAAGTGTGGGGATCGAACAGG
## 89                                                           ACGGAGGGTGCGAGCGTTAATCGGAATTACTGGGCGTAAAGCGCGCGTAGGCGGTTGGCTAAGTTTGCTGTGAAAGCCCCGGGCTTAACCTGGGAACTGCAGTGAATACTGGTCAGCTAGAGTATGGTAGAGGGTAGTGGAATTTCCGGTGTAGCAGTGAAATGCGTAGAGATCGGAAGGAACACCAGTGGCGAAGGCGGCTATCTGGACCAATACTGACGCTGAGGTGCGAAAGCGTGGGGAGCAAACAGG
## 90                                                           ACGTAGGGTGCAAGCGTTAATCGGAATTACTGGGCGTAAAGCGTGCGCAGGCGGTTTTGTAAGACAGAGGTGAAATCCCCGGGCTCAACCTGGGAACTGCCTTTGTGACTGCAAGGCTGGAGTACGGCAGAGGGGGATGGAATTCCGCGTGTAGCAGTGAAATGCGTAGATATGCGGAGGAACACCGATGGCGAAGGCAATCCCCTGGGCCTGTACTGACGCTCATGCACGAAAGCGTGGGGAGCAAACAGG
## 91                                                           ACGTAGGGTGCAAGCGTTAATCGGAATTACTGGGCGTAAAGGGTGCGCAGGCGGTATTGTAAGCCAGATGTGAAATCCCCGGGCTCAACCTGGGAACTGCGTTTGGAACTGCAATGCTAGAGTGTGTCAGAGGGGGGTGGAATTCCACGTGTAGCAGTGAAATGCGTAGAGATGTGGAGGAACACCGATGGCGAAGGCAGCCCCCTGGGATAACACTGACGCTCAGGCACGAAAGCGTGGGGAGCAAACAGG
## 92                                                           ACGTAGGGTGCAAGCGTTAATCGGAATTACTGGGCGTAAAGCGTGCGCAGGCGGTTATATAAGACAGATGTGAAATCCCCGGGCTCAACCTGGGAACTGCATTTGTGACTGTATAGCTAGAGTGCGGCAGAGGGGGATGGAATTCCGCGTGTAGCAGTGAAATGCGTAGATATGCGGAGGAACACCGATGGCGAAGGCAATCCCCTGGGCCTGCACTGACGCTCATGCACGAAAGCGTGGGGAGCAAACAGG
## 93                                                           ACGGAGGGTGCAAGCGTTAATCGGAATTACTGGGCGTAAAGCGCACGTAGGCTGTAGTGTAAGTCAGGGGTGAAATCCCACGGCTCAACCGTGGAACTGCCTTTGATACTGCACAACTTGAATCCGGGAGAGGGTGGCGGAATTCCAGGTGTAGGAGTGAAATCCGTAGATATCTGGAGGAACATCAGTGGCGAAGGCGGCCACCTGGACCGGTATTGACGCTGAGGTGCGAAAGCGTGGGGAGCAAACAGG
## 94                                                           ACGTAGGGTGCGAGCGTTAATCGGAATTACTGGGCGTAAAGCGTGCGCAGGCGGTTGTGCAAGACAGATGTGAAATCCCCGGGCTCAACCTGGGAATTGCATTTGTGACTGCACGGCTAGAGTGTGTCAGAGGGGGGTAGAATTCCACGTGTAGCAGTGAAATGCGTAGATATGTGGAGGAATACCGATGGCGAAGGCAGCCCCCTGGGATAACACTGACGCTCATGCACGAAAGCGTGGGGAGCAAACAGG
## 95                                                           ACGAAGGATCCAAGCGTTGTCCGGATTTACTGGGTTTAAAGGGTGCGTAGGCGGAAAATTAAGTCAGTGGTGAAAGCCCGCAGCTCAACTGTGGAACTGCCATTGAAACTGATTTTCTTGAATATAGCTGAGGCAGATGGAATATAACATGTAGCGGTGAAATGCTTAGATATGTTATAGAACACCGATTGCGAAGGCAGTCTGCTAAACTATTATTGACGCTGAGGCACGAAAGCGTGGGGAGCGAACAGG
## 96                                                           ACGTAGGGTGCAAGCGTTAATCGGAATTACTGGGCGTAAAGCGTGCGCAGGTGGTTCGTTAAGACAGTTGTGAAATCCCCGGGCTTAACCTGGGAACTGCAATTGTGACTGGCGGACTAGAGTTTGGCAGAGGGGGGTGGAATTCCTGGTGTAGCAGTGAAATGCGTAGATATCAGGAGGAACACCGATGGCGAAGGCAGCCCCCTGGGCCATGACTGACACTCATGCACGAAAGCGTGGGGAGCAAACAGG
## 97                                                           ACGTAGGGTGCGAGCGTTAATCGGAATTACTGGGCGTAAAGCGTGCGCAGGCGGACTTTTAAGCCAGATGTGAAAGCCCCGAGCTTAACTTGGGAATTGCGTTTGGAACTGGGAGTCTAGAGTCTGTCAGAGGGGGATGGAATTCCACGTGTAGCAGTGAAATGCGTAGAGATGTGGAGGAACACCGATGGCGAAGGCAGTCCCCTGGGATAAGACTGACGCTCATGCACGAAAGCGTGGGGAGCAAACAGG
## 98                                                           ACATAGGGTGCAAGCGTTGTCCGGAATTATTGGGCGTAAAGAGCTCGTAGGTTGTTTGTCGCGTCGATTGTGAAAATCTGAGGCTCAACCTCAGACCTGCAGTCGATACGGGCAAACTAGAGTGTGGTAGGGGAGACTGGAATTCCTGGTGTAGCGGTGGAATGCGCAGATATCAGGAGGAACACCAATGGCGAAGGCAGGTCTCTGGGCCATAACTGACACTGAGGAGCGAAAGTGCGGGGAGCGAACAGG
## 99                                                           ACGGAGGGTGCAAGCGTTATCCGGATTTATTGGGTTTAAAGGGTCCGTAGGCGGATCTGTAAGTCAGTGGTGAAATCTCACAGCTTAACTGTGAAACTGCCATTGATACTGCAGGTCTTGAGTGTTGTTGAAGTAGCTGGAATAAGTAGTGTAGCGGTGAAATGCATAGATATTACTTAGAACACCAATTGCGAAGGCAGGTTACTAAGCAACAACTGACGCTGATGGACGAAAGCGTGGGGAGCGAACAGG
## 100                                                          ACGAAGGGGGCTAGCGTTGCTCGGAATTACTGGGCGTAAAGGGAGCGTAGGCGGATAGTTTAGTCAGAGGTGAAAGCCCAGGGCTCAACCTTGGAAATGCCTTTGATACTGGCTATCTTGAGTATGGGAGAGGTAAGCGGAATTCCGAGTGTAGAGGTGAAATTCGTAGATATTCGGAAGAACACCAGTGGCGAAGGCGGCTTACTGGCCCATTACTGACGCTGAGGCTCGAAAGCGTGGGGAGCAAACAGG
## 101                                                          ACGTAGGGTGCAAGCGTTAATCGGAATTACTGGGCGTAAAGCGTGCGCAGGCGGTTTGTTAAGACAGTCGTGAAATCCCCGGGCTTAACCTGGGAACTGCGATTGTGACTGGCAAGCTAGAGTACGGCAGAGGGGGGTGGAATTCCTGGTGTAGCAGTGAAATGCGTAGAGATCAGGAGGAACACCGATGGCGAAGGCAGCCCCCTGGGCTAGTACTGACGCTCATGCACGAAAGCGTGGGGAGCAAACAGG
## 102                                                          ACGTAGGGTGCAAGCGTTAATCGGAATTACTGGGCGTAAAGCGTGCGCAGGCGGTTATATAAGACAGATGTGAAATCCCCGGGCTCAACCTGGGAACTGCATTTGTGACTGTATAGCTGGAGTGCGGCAGAGGGGGATGGAATTCCGCGTGTAGCAGTGAAATGCGTAGATATGCGGAGGAACACCGATGGCGAAGGCAATCCCCTGGGCCTGCACTGACGCTCATGCACGAAAGCGTGGGGAGCAAACAGG
## 103                                                          ACGGAGGATCCGAGCGTTATCCGGATTTATTGGGTTTAAAGGGAGCGTAGGTTGACGTATAAGTCAGCTGTGAAAGTTTACGGCTCAACCGTGAAATTGCAGTTGATACTGTATGTCTTGAGTGTACAAGAGGTGGGCGGAATTCGTGGTGTAGCGGTGAAATGCTTAGATATCACGAAGAACTCCAATTGCGAAGGCAGCTCACTGGGGTACAACTGACACTGAGGCTCGAAAGTGTGGGTATCAAACAGG
## 104                                                          ACGGAGGATCCAAGCGTTATCCGGAATCATTGGGTTTAAAGGGTCCGTAGGCGGTCTTATAAGTCAGTGGTGAAATCCCATCGCTCAACGATGGAACTGCCATTGATACTGTAAGACTTGAATGCTTAGGAAGTAACTAGAATATGTAGTGTAGCGGTGAAATGCTTAGATATTACATGGAATACCAATTGCGAAGGCAGGTTACTACTAAGTGATTGACGCTGATGGACGAAAGCGTGGGGAGCGAACAGG
## 105                                                        ACGTAGGGTACAAGCATTAATCGGAATGATTGGGCGTAAAGGGCGCGTAGGCGGCCTTGTAAGTCAGGTGTGAAATTCCAAAGCTCAACTTTGGAGCTGCACTTGAAACTACAAGGCTAAAGAGGTAAGACGGAGAAAGCGGAATTCCACGTGTAGCGGTGAAATGCGTAGATATGTGGAGGAACACCTGTGGCGAAAGCGGCTTTCTAGTTTTAACCTGACGCTGAGGCGCGAGAGCAAGGGGAGCAAACAGG
## 106                                                          ACGTAGGTGACAAGCGTTATCCGGATTTACTGGGTGTAAAGGGCGTGTAGGCGGTTTTGCAAGTCAGATGTGAAATTCCCGGGCTCAACTCGGGCGCTGCATCTGAAACTGTAAAACTTGAGTACTGGAGAGGATAGCGGAATTCCTAGTGTAGCGGTAAAATGCGCAGATATTAGGAGGAACACCGGTGGCGAAGGCGGCTATCTGGACAGTAACTGACGCTGAGGCGCGAAAGCGTGGGGAGCAAACAGG
## 107                                                          ACGAAGGGTGCGAGCGTTGTTCGGAATTACTGGGCGTAAAGGGTTCGTAGGCGGGAATGCAAGTCAAGTGTGAAATCCCCAGGCTTAACCTGGGACGTGCATTTGAGACTGTGTTTCTTGAGTTTCGGAGAGGGTGGTGGAATTGCTGGTGTAGGAGTGACATCCGTAGAGATCAGCAGGAACACCGGAGGCGAAGGCGACCACCTGGCCGGATACTGACGCTGAGGAACGAAAGCGTGGGGAGCAAACAGG
## 108                                                          ACGGAGGATCCGAGCGTTATCCGGATTTATTGGGTTTAAAGGGAGCGTAGATGGATGTTTAAGTCAGTTGTGAAAGTTTGCGGCTCAACCGTAAAATTGCAGTTGATACTGGATATCTTGAGTGCAGTTGAGGCAGGCGGAATTCGTGGTGTAGCGGTGAAATGCTTAGATATCACGAAGAACTCCGATTGCGAAGGCAGCCTGCTAAGCTGCAACTGACATTGAGGCTCGAAAGTGTGGGTATCAAACAGG
## 109                                                          ACGTAGGGTGCGAGCGTTAATCGGAATTACTGGGCGTAAAGCGTGCGCAGGTGGTTGTTTAAGACAGATGTGAAATCCCCGGGCTTAACCTGGGAACTGCGTTTGTGACTGGACAGCTAGAGTGCGGCAGAGGGGGGTGGAATTCCACGTGTAGCAGTGAAATGCGTAGAGATGTGGAGGAACACCGATGGCGAAGGCAGCCCCCTGGGCCAGCACTGACGCTCATGCACGAAAGCGTGGGGAGCAAACAGG
## 110                                                         ACGTAGGCTCCAAGCGTTGTCCGGATTTATTGGGCGTAAAGAGCTCGTAGGCGGTTGAGTAAGTCGGGTGTGAAAACTCTGGGCTTAACCCGGAGACGCCATCCGATACTGCTCTGACTAGAGTTCAGGAGGGGAGTGGGGAATTCCTAGTGTAGCGGTGAAATGCGCAGATATTAGGAGGAACACCGGTGGCGAAGGCGCCACTCTGGACTGAAACTGACGCTGAGGAGCGAAAGCATGGGTAGCAAACAGG
## 111                                                          ACGGAGGATGCAAGTGTTATCCGGAATCACTGGGCGTAAAGCGTCTGTAGGTGGTTTAATAAGTCAACTGTTAAATCTTGAGGCTCAACTTCAAAATCGCAGTCGAAACTATTAGACTAGAGTATAGTAGAGGTAAAGGGAATTTCCAGTGGAGCGGTGAAATGCGTAGATATTGGAAAGAACACCGATGGCGAAAGCACTTTACTGGGCTATTACTAACACTCAGAGACGAAAGCTAGGGTAGCAAATGGG
## 112                                                          ACGTAGGGTGCGAGCGTTAATCGGAATTACTGGGCGTAAAGCGTGCGCAGGCGGTTATATAAGACAGTTGTGAAATCCCCGGGCTCAACCTGGGAATTGCATCTGTGACTGTATAGCTAGAGTACGGTAGAGGGGGATGGAATTCCGCGTGTAGCAGTGAAATGCGTAGATATGCGGAGGAACACCGATGGCGAAGGCAATCCCCTGGACCTGTACTGACGCTCATGCACGAAAGCGTGGGGAGCAAACAGG
## 113                                                          ACGTAGGGTGCAAGCGTTAATCGGAATTACTGGGCGTAAAGCGTGCGCAGGCGGTTATACAAGACAGGCGTGAAATCCCCGGGCTTAACCTGGGAATGGCGTCTGTGACTGTATGACTAGAGTGTGTCAGAGGGGGGTAGAATTCCACGTGTAGCAGTGAAATGCGTAGATATGTGGAGGAATACCAATGGCGAAGGCAGCCCCCTGGGATAACACTGACGCTCATGCACGAAAGCGTGGGGAGCAAACAGG
## 114                                                          ACGAAGGGTGCAAGCGTTAATCGGAATTACTGGGCGTAAAGCGCGCGTAGGCGGTTTGGTTAGTTGGATGTGAAAGCCCCGGGCTCAACCTGGGAACTGCATCCAATACTGCCAAGCTAGAGTACGGTAGAGGGGGGTAGAATTCCACGTGTAGCGGTGAAATGCGTAGAGATGTGGAGGAATACCAGTGGCGAAGGCGGCCCCCTGGATCGATACTGACGCTGAGGTGCGAAAGCGTGGGGAGCAAACAGG
## 115                                                          ACGTAGGGTGCGAGCGTTAATCGGAATTACTGGGCGTAAAGCGTGCGCAGGCGGCTTTGCAAGACAGAGGTGAAATCCCCGGGCTCAACCTGGGAACTGCCTTTGTGACTGCAAGGCTAGAGTACGGCAGAGGGAGATGGAATTCCGCGTGTAGCAGTGAAATGCGTAGATATGCGGAGGAACACCGATGGCGAAGGCAATCTCCTGGGCCTGTACTGACGCTCATGCACGAAAGCGTGGGGAGCAAACAGG
## 116                                                          ACGTAGGGTGCAAGCGTTAATCGGAATTACTGGGCGTAAAGCGTGCGCAGGCGGTTTGAAAAGTCAGCTGTGAAAGCCCCGGGCTCAACCTGGGAACTGCGGTTGAAACTCTCAAGCTAGAGTGCGTCAGAGGGGGGTGGAATTCCACGTGTAGCAGTGAAATGCGTAGAGATGTGGAGGAACACCAATGGCGAAGGCAGCCCCCTGGGATGACACTGACGCTCATGCACGAAAGCGTGGGGAGCAAACAGG
## 117                                                          ACGTAGGGTGCAAGCGTTAATCGGAATTACTGGGCGTAAAGCGTGCGCAGGCGGTTATGTAAGACAGAGGTGAAATCCCCGGGCTCAACCTGGGAACTGCCTTTGTGACTGCGAGGCTAGAGTATGGCAGAGGGGGGTGGAATTCCACGTGTAGCAGTGAAATGCGTAGAGATGTGGAGGAACACCGATGGCGAAGGCAGCCCCCTGGGCCAATACTGACGCTCATGCACGAAAGCGTGGGGAGCAAACAGG
## 118                                                          ACAGAGGTCTCAAGCGTTGTTCGGAATCACTGGGCGTAAAGGGTGCGTAGGTTGCGTGGTAAGTCAGATGTGAAAGCCTGGGGCTCAACCTCAGAATTGCATCCGATACTGCCGTGCTAGAGTACTGAAGAGGTGACTAGAATTCTAGGTGTAGCAGTGAAATGCGTAGATATCTAGAGGAATACCAAAGGCGTAGGCAGGTCACTGGGCAGTTACTGACACTGAGGCACGAAGGCCAGGGGAGCAAACGGG
## 119                                                          ACGGAGGGTGCAAGCGTTATCCGGATTTACTGGGTTTAAAGGGTGCGTAGGCGGACAGGTAAGTCAGTGGTGAAATCCCCGAGCTTAACTCGGGAACTGCCGTTGATACTATCTGTCTTGAATATCGTGGAGGTAAGCGGAATATGTCATGTAGCGGTGAAATGCTTAGATATGACATAGAACACCAATTGCGAAGGCAGCTTGCTACACGGTCATTGACGCTGAGGCACGAAAGCGTGGGGATCAAACAGG
## 120                                                          ACGTAGGGTGCAAGCGTTAATCGGAATTACTGGGCGTAAAGCGTGCGCAGGCGGTTATGCAAGACAGATGTGAAATCCCCGGGCTCAACCTGGGAACTGCATTTGTGACTGCATAGCTAGAGTACGGTAGAGGGGGATGGAATTCCGCGTGTAGCAGTGAAATGCGTAGATATGCGGAGGAACACCGATGGCGAAGGCAATCCCCTGGACCTGTACTGACGCTCATGCACGAAAGCGTGGGGAGCAAACAGG
## 121                                                          ACGTAGGGTGCGAGCGTTAATCGGAATTACTGGGCGTAAAGCGTGCGCAGGCGGTTATGTAAGACAGATGTGAAATCCCCGGGCTCAACCTGGGAACTGCATTTGTGACTGCATAGCTTGAGTGCGGCAGAGGGGGATGGAATTCCGCGTGTAGCAGTGAAATGCGTAGATATGCGGAGGAACACCGATGGCGAAGGCAATCCCCTGGGCCTGCACTGACGCTCATGCACGAAAGCGTGGGGAGCAAACAGG
## 122                                                          ACGTAGGGTGCAGGCGTTAATCGGAATTACTGGGCGTAAAGCGTGCGCAGGCGGTTTGCTAAGACAGGTGTGAAATCCCCGGGCTTAACCTGGGAATTGCGCTTGTAACTGGCAAGCTAGAGTGTGGCAGAGGGGGGTGGAATTCCACGTGTAGCAGTGAAATGCGTAGATATGTGGAGGAACACCGATGGCGAAGGCAGCCCCCTGGGTTAACACTGACGCTCAGGCACGAAAGCGTGGGGAGCAAACAGG
## 123                                                          ACGTAGGGTGCGAGCGTTAATCGGAATTACTGGGCGTAAAGCGTGCGCAGGCGGTTTTGTAAGACAGAGGTGAAATCCCCGGGCTCAACCTGGGAACTGCCTTTGTGACTGCAAGGCTAGAGTGCGGCAGAGGGGGATGGAATTCCGCGTGTAGCAGTGAAATGCGTAGATATGCGGAGGAACACCGATGGCGAAGGCAATCCCCTGGGCCTGCACTGACGCTCATGCACGAAAGCGTGGGGAGCAAACAGG
## 124                                                          ACAGAGGGTGCAAGCGTTAATCGGAATTACTGGGCGTAAAGCGCGCGTAGGCGGTTGTGTAAGTTGGATGTGAAATCCCCGGGCTTAACCTGGGCACTGCATTCAAAACTGCACGGCTAGAGTATGGGAGAGGAAGGTAGAATTCCAGGTGTAGCGGTGAAATGCGTAGAGATCTGGAGGAATACCGATGGCGAAGGCAGCCTTCTGGCCTAATACTGACGCTGAGGTGCGAAAGCATGGGGAGCAAACAGG
## 125                                                          ACGTAGGGTGCAAGCGTTAATCGGAATTACTGGGCGTAAAGCGTGCGCAGGCGGTTATGTAAGACAGAGGTGAAATCCCCGGGCTCAACCTGGGAACTGCCTTTGTGACTGCATGACTAGAGTACGGCAGAGGGGGATGGAATTCCGCGTGTAGCAGTGAAATGCGTAGATATGCGGAGGAACACCGATGGCGAAGGCAATCCCCTGGGCCTGTACTGACGCTCATGCACGAAAGCGTGGGGAGCAAACAGG
## 126                                                          ACAGAGGGTGCGAGCGTTAATCGGAATTACTGGGCGTAAAGCGCATGCAGATGGTTTGACAAGTCGGATGTGAAAGCCCCGGGCTTAACCTGGGAACGGCACTCGAAACTGTCAGACTAGAATGTGGAAGAGGGAAGTGGAATTCCGGGTGTAGCAGTGAAATGCGTAGATATCCGGAGGAACATCAGTGGCGAAGGCGACTTCCTGGTCCAACATTGACATTCAGATGCGAAAGCGTGGGGAGCAAACAGG
## 127                                                          ACGTAGGGTGCAAGCGTTAATCGGAATTACTGGGCGTAAAGCGTGCGCAGGCGGTTTCGTAAGACAGACGTGAAATCCCCGGGCTCAACCTGGGAACTGCGTTTGTGACTGCGAGGCTAGAGTATGGCAGAGGGGGGTGGAATTCCACGTGTAGCAGTGAAATGCGTAGAGATGTGGAGGAACACCGATGGCGAAGGCAGCCCCCTGGGCCAATACTGACGCTCATGCACGAAAGCGTGGGGAGCAAACAGG
## 128                                                          ACGTAGGGTGCAAGCGTTAATCGGAATTACTGGGCGTAAAGCGTGCGCAGGCGGTGATGTAAGACAGGTGTGAAATCCCCGGGCTTAACCTGGGAACTGCACTTGTGACTGCATCGCTGGAGTACGGCAGAGGGGGATGGAATTCCGCGTGTAGCAGTGAAATGCGTAGATATGCGGAGGAACACCGATGGCGAAGGCAATCCCCTGGGCCTGTACTGACGCTCATGCACGAAAGCGTGGGGAGCAAACAGG
## 129                                                          ACGAAGGGTGCAAGCGTTACTCGGAATTACTGGGCGTAAAGCGTGCGTAGGTGGTTTGTTAAGTCAGATGTGAAAGCCCCGGGCTCAACCTGGGAATTGCATTTGATACTGGCAGACTAGAGTGCGGTAGAGGAGAGTGGAATTCCCGGTGTAGCAGTGAAATGCGTAGAGATCGGGAGGAACATCAGTTGCGAAGGCGGCTCTCTGGACCAGCACTGACACTGAGGCACGAAAGCGTGGGGAGCAAACAGG
## 130                                                          ACGTAGGGTGCGAGCGTTAATCGGAATTACTGGGCGTAAAGCGTGCGCAGGCGGTTTTGAAAGTCAGATGTGAAATCCCCGGGCTCAACCTGGGAACTGCGTTTGAAACTCCAAAGCTAGAGTATGGGAGAGGGAGGTAGAATTCCACGTGTAGCAGTGAAATGCGTAGAGATGTGGAGGAATACCAATGGCGAAGGCAGCCTCCTGGCCTAATACTGACGCTCATGCACGAAAGCGTGGGGAGCAAACAGG
## 131                                                          ACGGAGGATCCAAGCGTTATCCGGAATCATTGGGTTTAAAGGGTCCGTAGGCGGTTTAATAAGTCAGTGGTGAAATCTGGTCGCTCAACGATCAAACGGCCATTGATACTGTTAGACTTGAATTACTGGGAAGTAACTAGAATATGTAGTGTAGCGGTGAAATGCTTAGATATTACATGGAATACCAATTGCGAAGGCAGGTTACTACCAGTGGATTGACGCTGATGGACGAAAGCGTGGGGAGCGAACAGG
## 132                                                          ACGTAGGGTGCAAGCGTTAATCGGAATTACTGGGCGTAAAGCGTGCGCAGGCGGTTCGGAAAGAGAGGTGTGAAATCCCAGGGCTCAACCTTGGAACTGCACTTTTAACTACCGAGCTAGAGTATGTCAGAGGGGGGTGGAATTCCGCGTGTAGCAGTGAAATGCGTAGATATGCGGAGGAACACCGATGGCGAAGGCAGCCCCCTGGGATAATACTGACGCTCATGCACGAAAGCGTGGGGAGCAAACAGG
## 133                                                          ACGAAGGTGGCAAGCGTTACCCGGATTGATTGGGCGTAAAGCGTCCGCAGACTGTATGATAAGTTCTTGGTCAAATCCTGAAGCTCAACTTCAGGGCCGCTGAGAATACTGTCATGCTAGAGACTGGGAGAGGTAAGCGGAATTACCGGTGTAGCAGTAAAATGCGTTAAGATCGGTAGGAACACCAAATGCGAAGGCAGCTTACTGGAACAGTTCTGACGTTATAGGACGAAAGCGTGGGGAGCGAATGGG
## 134                                                          ACAGAGGTCTCAAGCGTTGTTCGGATTCATTGGGCGTAAAGGGTGCGTAGGTGGCAAGGTAAGTCAGATGTGAAATCCCCGGGCTCAACCTGGGAACTGCATTTGATACTGCCGTGCTCGAGTGCTGGAGGGGAGACTGGAATTCACGGTGTAGCAGTGAAATGCGTAGATATCGTGAGGAAGACCGGTGGCGAAGGCGAGTCTCTGGACAGCAACTGACACTGAGGCACGAAGGCTAGGGGAGCAAACGGG
## 135                                                          ACGGAGGGTGCAAGCGTTATCCGGAATCATTGGGTTTAAAGGGTCCGCAGGCGGTCTTATAAGTCAGTGGTGAAATCTCGTAGCTTAACTATGAAACTGCCATTGATACTGTAGGACTTGAATTCGGTCGAAGTGGGCGGAATATGACATGTAGCGGTGAAATGCGTAGATATGTCATAGAACACCGATAGCGAAGGCAGCTCACTAGGCCTGGATTGACGCTCAGGGACGAAAGCGTGGGGAGCAAACAGG
## 136                                                          ACGTAGGGTGCAAGCGTTAATCGGAATTACTGGGCGTAAAGCGTGCGCAGGCGGTTTTGTAAGACAGGCGTGAAATCCCCGGGCTCAACCTGGGAATGGCGCTTGTGACTGCAAAGCTGGAGTGCGGCAGAGGGGGATGGAATTCCGCGTGTAGCAGTGAAATGCGTAGATATGCGGAGGAACACCGATGGCGAAGGCAATCCCCTGGGCCTGCACTGACGCTCATGCACGAAAGCGTGGGGAGCAAACAGG
## 137                                                          ACGGAGGGTGCAAGCGTTAATCGGAATTACTGGGCGTAAAGCGCACGCAGGCGGCTTGTTAAGTCAGATGTGAAAGCCCCGGGCTCAACCTGGGAACTGCATTTGAAACTGACAAGCTAGAGTCTTGTAGAGGGGGGTAGAATTCCAGGTGTAGCGGTGAAATGCGTAGAGATCTGGAGGAATACCGGTGGCGAAGGCGGCCCCCTGGACAAAGACTGACGCTCAGGTGCGAAAGCGTGGGGAGCAAACAGG
## 138                                                          ACAGAGGATGCAAGCGTTATCCGGAATCACTGGGCATAAAGCGTCTGTAGGTTGCTTGTCAAGTCTGCTGTTAAAGATCAGGGCCTAACCCTGGAAAAGCAGTGGAAACTAATAGGCTTGAGTGTGGTAGAGGTAGAGGGAATTCCTGGTGTAGCGGTGAAATGCGTAGATATTAGGAAGAACACCAATGGCGAAAGCACTCTACTGGGCCATAACTGACACTGAGAGACGACAGCTAGGGGAGCAAATGGG
## 139                                                          ACAGAGACTGCAAGCGTTATTCGGATTCACTGGGCGTAAAGGGTGCGCAGGCGGCCGTGTGTGTTAGGCGTGAAAGCCCGAGGCTCAACCTCGGAATTGCACCTAAAACTACACGGCTAGAGTACTGGAGAGGGTAGCGGAATTCACGGTGTAGCAGTGAAATGCGTAGATATCGTGAGGAACACCAATGGCGAAGGCAGCTACCTGGACAGTAACTGACGCTCAGGCACGAAAGCGTGGGGAGCAAAAGGG
## 140                                                          ACGAAGGATCCAAGCGTTGTCCGGATTTACTGGGTTTAAAGGGTGCGTAGGCGGAAAATTAAGTCAGTGGTGAAAGCCCGCAGCTCAACTGCGGAACTGCCATTGAAACTGATTTTCTTGAATACGGTTGAGGTAGATGGAATATAACATGTAGCGGTGAAATGCTTAGATATGTTATAGAACACCAATTGCGAAGGCAGTCTACTAAGCCGTGATTGACGCTGAGGCACGAAAGCGTGGGGAGCGAACAGG
## 141                                                          ACGTAGGGTGCGAGCGTTAATCGGAATTACTGGGCGTAAAGCGTGCGCAGGTGGTTTTGTAAGCTTGATGTGAAATCCCCGGGCTCAACCTGGGAACTGCATTGAGGACTGCAAGACTAGAGTGTGGCAGAGGGGGGTGGAATTCCACGTGTAGCAGTGAAATGCGTAGAGATGTGGAGGAACACCGATGGCGAAGGCAGCCCCCTGGGCTAACACTGACACTCATGCACGAAAGCGTGGGGAGCAAACAGG
## 142                                                          ACGTAGGGTGCGAGCGTTAATCGGAATTACTGGGCGTAAAGGGTGCGCAGGCGGCGCCATAAGACAGCTGTGAAATCCCCGGGCTTAACCTGGGAACTGCGGTTGTGACTGTGGTGCTTGAGTGCGGCAGAGGGGGGTGGAATTCCACGTGTAGCAGTGAAATGCGTAGAGATGTGGAGGAACACCGATGGCGAAGGCAGCCCCCTGGGTCGACACTGACGCTCATGCACGAAAGCGTGGGGAGCAAACAGG
## 143                                                          ACGGAGGATCCAAGCGTTATCCGGAATCATTGGGTTTAAAGGGTCCGTAGGCGGTCTAATAAGTCAGTGGTGAAAGCCCATCGCTCAACGATGGAACGGCCATTGATACTGTTAGACTTGAATTACTGGGAAGTAACTAGAATATGTAGTGTAGCGGTGAAATGCTTAGATATTACATGGAATACCAATTGCGAAGGCAGGTTACTACCAGTGGATTGACGCTGATGGACGAAAGCGTGGGGAGCGAACAGG
## 144                                                          ACGGAGGGGGCTAGCGTTATTCGGAATTACTGGGCGTAAAGCGCACGTAGGCGGACCAGAAAGTCAGAGGTGAAAGCCCGGGGCTCAACCCCGGAACTGCCTTTGAAACTCCTGGTCTTGAGGTCGAGAGAGGTGAGTGGAATTCCGAGTGTAGAGGTGAAATTCGTAGATATTCGGAGGAACACCAGTGGCGAAGGCGGCTCACTGGCTCGATACTGACGCTGAGGTGCGAAAGCGTGGGGAGCAAACAGG
## 145                                                          ACAGAGGGTGCAAGCGTTATCCGGAATTATTGGGCGTAAAGCGTCCGCAGGTGGTTTGTTAAGTCGAGTATTAAAGACGGCAGCTCAACTGTCGGAATGTATTCGATACTGGCAAGCTAGAGTCTTGGAGGGGTGAGCAGAATTCTGCATGTAGGGGTAAAATCCGTAGATATGCAGAGGAATACCAAAAGCGAAGGCAGCTCACTGGCCAAGTACTGACACTCATGGACGAAAGCGTGGGGAGCAAACAGG
## 146                                                          ACGGAGGGTGCAAGCGTTAATCGGAATTACTGGGCGTAAAGCGCACGTAGGTGGTTTGTTAAGCCAGCTGTGAAATCCCCGGGCTCAACCTGGGCACTGCAGTTGGAACTGGCAAGCTAGAGTAGGGTAGAGGGGTGTGGAATTCCAGGTGTAGCGGTGAAATGCGTAGATATCTGGAGGAACATCAGTGGCGAAGGCGACACCCTGGACTCATACTGACACTGAGGTGCGAAAGCGTGGGGAGCAAACAGG
## 147                                                          ACGGAGGGAGCTAGCGTTGTTCGGAATTACTGGGCGTAAAGCGTGCGTAGGCGGCTATTCAAGTCAGAGGTGAAAGCCTGGAGCTCAACTCCAGAACTGCCTTTGAAACTAGATAGCTAGAATCTTGGAGAGGTGAGTGGAATTCCGAGTGTAGAGGTGAAATTCGTAGATATTCGGAAGAACACCAGTGGCGAAGGCGACTCACTGGACAAGTATTGACGCTGAGGTACGAAAGCGTGGGGAGCAAACAGG
## 148                                                          ACGGAGGGTGCAAGCGTTACTCGGAATCACTGGGCGTAAAGAGCGTGTAGGCGGGTATATAAGTCAGAAGTGAAATCCTATAGCTTAACTATAGAACTGCTTTTGAAACTGTATACCTAGAATGTGGGAGAGGTAGATGGAATTTCTGGTGTAGGGGTAAAATCCGTAGAGATCAGAAGGAATACCGATTGCGAAGGCGATCTACTGGAACATTATTGACGCTGAGACGCGAAAGCGTGGGGAGCAAACAGG
## 149                                                          ACGTAGGGTGCAAGCGTTAATCGGAATTACTGGGCGTAAAGCGTGCGCAGGTGGTTTGTTAAGACAGTTGTGAAATCCCCGGGCTTAACCTGGGAACTGCAATTGTGACTGGCAGACTAGAGTGAGGCAGAGGGGGGCGGAATTCCTGGTGTAGCAGTGAAATGCGTAGATATCAGGAGGAACACCGATGGCGAAGGCAGCCCCCTGGGCCATCACTGACACTCATGCACGAAAGCGTGGGGAGCAAACAGG
## 150                                                          ACGTAGGGTGCGAGCGTTAATCGGAATTACTGGGCGTAAAGCGTGCGCAGGCGGTTATATAAGACAGATGTGAAATCCCCGGGCTCAACCTGGGAACTGCATTTGTGACTGTATAGCTAGAGTGCGGCAGAGGGGGATGGAATTCCGCGTGTAGCAGTGAAATGCGTAGATATGCGGAGGAACACCGATGGCGAAGGCAATCCCCTGGGCCTGCACTGACGCTCATGCACGAAAGCGTGGGGAGCAAACAGG
## 151                                                          ACAGAGGTCCCGAGCGTTGTTCGGATTCACTGGGCGTAAAGGGTGCGTAGGTGGTTGGTTAAGTTTGAGGTGAAAGCTCCGAGCTCAACTCGGAAAATGCCTTGAAGACTATCTGACTAGAGGATTGGAGGGGAGATTGGAATTCTCGGTGTAGCAGTGAAATGCGTAGATATCGAGAGGAACACCAGTGGCGAAGGCGAATCTCTGGACAATTCCTGACACTGAGGCACGAAAGCCAAGGGAGCAAACGGG
## 152                                                          ACGTAGGGTGCGAGCGTTAATCGGAATTACTGGGCGTAAAGCGTGCGCAGGCGGTTTTGTAAGACAGATGTGAAATCCCCGGGCTCAACCTGGGACCTGCATTTGTGACTGCAAGGCTGGAGTACGGCAGAGGGGGATGGAATTCCGCGTGTAGCAGTGAAATGCGTAGATATGCGGAGGAACACCGATGGCGAAGGCAATCCCCTGGGCCTGTACTGACGCTCATGCACGAAAGCGTGGGGAGCAAACAGG
## 153                                                          CCCAGCGGTCCAAGTCGCAACCACAATTATTGGGTCTAAAACATCCGTAGCTTGCTTGATAAGTCTCTTGTGAAATCCGGCCTCTTAAGGGTCGAAGAGCGAGAGATACTGTCGGGCTAGAGACCGGGAGAGGTAAGAAGTACGGTCGGGGTACCGGTAAAATGGGTTGATCCCGTCTGGACTAACAACAGCGAAGGCATCTTACCAGCACGGATCTGACAGTAAGGGATGAAGGCTAGGGGCGCAAAAGGG
## 154                                                          ACATAGGGTGCAAGCGTTGTCCGGAATTATTGGGCGTAAAGAGCTCGTAGGTGGTTCGATACGTCGGATGTGAAAATCAGGGGCTCAACCCCTGACCTGCATCCGATACGGTCGAGCTGGAGTTTGGTAGGGGAGACTGGAATTCCTGGTGTAGCGGTGGAATGCGCAGATATCAGGAGGAACACCGATGGCGAAGGCAGGTCTCTGGGCCAATACTGACACTGAGGAGCGAAAGCGTGGGGAGCGAACAGG
## 155                                                          ACATAGGTCCCAAGCGTTATCCGGAATTACTGGGCGTAAAGCGTCTGCAGGTGGAAAAGTGTGTGAGATGTGAAAGACCGGGGCTCAACCCCGTGTTTGTGTCTCAAACTGCTTTTCTAGAGTGAGTAAGAGGTATGCGGAATTTATGGAGTAGGAGTGCAATCCGTTGACACCATAAAGAACACCAAAAGCGAAGGCAGCATACTGGGGCTCTACTGACACTCAGAGACGAAAGCGTGGGGAGCGAAAGGG
## 156                                                          ACGAAGGATCCAAGCGTTGTCCGGATTTACTGGGTTTAAAGGGTGCGTAGGCGGAAAATTAAGTCAGTGGTGAAAGCCCGCAGCTCAACTGTGGAACTGCCATTGAAACTGGTTTTCTTGAATATAGCTGAGGCAGATGGAATATAACATGTAGCGGTGAAATGCTTAGATATGTTATAGAACACCGATTGCGAAGGCAGTCTGCTAAACTATTATTGACGCTGAGGCACGAAAGCGTGGGGAGCGAACAGG
## 157                                                          ACGTAGGGTGCGAGCGTTAATCGGAATTACTGGGCGTAAAGCGTGCGCAGGCGGTGATGTAAGACAGATGTGAAATCCCCGGGCTCAACCTGGGAACTGCGTTTGTGACTGCATCACTCGAGTACGGCAGAGGGAGGTGGAATTCCACGTGTAGCAGTGAAATGCGTAGAGATGTGGAGGAACACCGATGGCGAAGGCAGCCTCCTGGGCCAGTACTGACGCTCATGCACGAAAGCGTGGGGAGCAAACAGG
## 158                                                          ACGGAGGGTGCAAGCGTTAATCGGAATTACTGGGCGTAAAGCGCACGCAGGCGGTTTGTTAAGTCAGATGTGAAATCCCCGGGCTCAACCTGGGAACTGCATCTGATACTGGCAAGCTTGAGTCTCGTAGAGGGGGGTAGAATTCCAGGTGTAGCGGTGAAATGCGTAGAGATCTGGAGGAATACCGGTGGCGAAGGCGGCCCCCTGGACGAAGACTGACGCTCAGGTGCGAAAGCGTGGGGAGCAAACAGG
## 159                                                          ACGTAGGGTGCAAGCGTTAATCGGAATTACTGGGCGTAAAGCGTGCGCAGGCGGTCTTGTAAGACAGATGTGAAATCCCCGGGCTCAACCTGGGACCTGCATTTGTGACTGCAAGGCTGGAGTACGGCAGAGGGGGATGGAATTCCGCGTGTAGCAGTGAAATGCGTAGATATGCGGAGGAACACCGATGGCGAAGGCAATCCCCTGGGCCTGTACTGACGCTCATGCACGAAAGCGTGGGGAGCAAACAGG
## 160                                                         ACGTGAGTGGCAAGCGTTATCCGGAATCATTGGGCGTAAAGCGTCCGTAGGGGGGTGTTTAAGTGAAATGTTAAATTCTTCGACCCAATCGAAGGCCTGCGTTTCATACTGGACACCTAGAAGATGGGAGAGGTAAATAGAATTTCTGGAGTAGGGGTAATATCCGTAGAGACCAGAAGGAATACCAATAGCGAAGGCAATTTACTGGCCCATTCTTGACCCTCAAGTGACGAAAGCGTGGGGAGCAAACAGG
## 161                                                          ACGAGTGCCCCAAGCGTTATCCGGAATTACTGGGCGTAAAGCGTGCGTAGGTGGCATGGTTAGTCTCCTGTTAAAGCTCTCGGCTTAACCGAGAAAGTGCGGGAGATACGGCCAAGCTCGAGGTGGTGAGAGGTCTATGGAACTCATGGTGTAGGGGTGAAATCCGTTGATATCATGGGGAACACCAAATGCGAAGGCAATAGACTGGCACCATACTGACACTGGAGCACGAAAGCGTGGGGATCAAACGGG
## 162                                                          ACGGAGGGTGCAAGCGTTATCCGGATTTATTGGGTTTAAAGGGTCCGTAGGCGGACTTATAAGTCAGTGGTGAAAGCCTGTCGCTTAACGATAGAACTGCCATTGATACTGTAAGTCTTGAGTATATTTGAGGTAGCTGGAATAAGTAGTGTAGCGGTGAAATGCATAGATATTACTTAGAACACCAATTGCGAAGGCAGGTTACCAAGATATAACTGACGCTGAGGGACGAAAGCGTGGGGAGCGAACAGG
## 163                                                          ACGGAGGGTGCAAGCGTTATCCGGATTCACTGGGTTTAAAGGGTGCGTAGGCGGGCAGGTAAGTCAGTGGTGAAATCCTAGAGCTTAACTCTAGAACTGCCATTGATACTATCTGTCTTGAATATTGTGGAGGTAAGCGGAATATGTCATGTAGCGGTGAAATGCTTAGATATGACATAGAACACCTATTGCGAAGGCAGCTTACTACGCATATATTGACGCTGAGGCACGAAAGCGTGGGGATCAAACAGG
## 164                                                          ACGTAGGGTGCAAGCGTTAATCGGAATTACTGGGCGTAAAGCGTGCGCAGGCGGACTTTTAAGCCAGATGTGAAAGCCCCGAGCTTAACTTGGGAATTGCGTTTGGAACTGGGAGTCTAGAGTCTGTCAGAGGGGGATGGAATTCCACGTGTAGCAGTGAAATGCGTAGATATGCGGAGGAACACCGATGGCGAAGGCAATCCCCTGGGCCTGTACTGACGCTCATGCACGAAAGCGTGGGGAGCAAACAGG
## 165                                                          ACGAAGGGTGCAAGCGTTAATCGGAATTACTGGGCGTAAAGCGTGCGTAGGTGGTTTGTTAAGTCAGATGTGAAAGCCCTGGGCTCAACCTGGGAACTGCATTTGATACTGGCAGGCTAGAGTGCGGTAGAGGGTGGTGGAATTCCCGGTGTAGCGGTGAAATGCGTAGAGATCGGGAGGAACATTCGTGGCGAAGGCGGCCACCTGGACCAGCACTGACACTGAGGCACGAAAGCGTGGGGAGCAAACAGG
## 166                                                          ACGGAGGATCCAAGCGTTGTCCGGATTTATTGGGTTTAAAGGGTGCGTAGGCGGATCTTTAAGTCAGTGGTGAAAGCCTGCAGCTTAACTGTAGAACAGCCATTGAAACTGAAGATCTTGAATTTGGTTAAAGTAGGCGGAATGTATCATGTAGCGGTGAAATGCTTAGATATGATACAGAACACCGATAGCGAAGGCAGCTTGCTGAACCAATATTGACGCTGAGGCACGAAAGCGTGGGGAGCGAACAGG
## 167                                                          ACGTAGGGTGCAAGCGTTAATCGGAATTACTGGGCGTAAAGCGTGCGCAGGCGGTTTTGTAAGTCGGATGTGAAATCCCCGGGCTCAACCTGGGAACTGCGTTCGAAACTGCAAGGCTAGAGTGTGTCAGAGGGGGGTAGAATTCCACGTGTAGCAGTGAAATGCGTAGAGATGTGGAGGAATACCAATGGCGAAGGCAGCCCCCTGGGATAACACTGACGCTCATGCACGAAAGCGTGGGGAGCAAACAGG
## 168                                                          ACGTAGGGTGCGAGCGTTAATCGGAATTACTGGGCGTAAAGCGTGCGCAGGCGGTTATATAAGACAGATGTGAAATCCCCGGGCTTAACCTGGGACCTGCATTTGTGACTGTATAGCTAGAGTACGGTAGAGGGGGATGGAATTCCGCGTGTAGCAGTGAAATGCGTAGATATGCGGAGGAACACCGATGGCGAAGGCAATCCCCTGGACCTGTACTGACGCTCATGCACGAAAGCGTGGGGAGCAAACAGG
## 169                                                          ACGGAGGGTGCAAGCGTTGTCCGGATTTATTGGGTTTAAAGGGTGCGCAGGTGGTTTATTAAGTCAGTGGTGAAAGACGGTCGCTCAACGATTGCAGTGCCATTGATACTGGTAGACTTGAGTAAAGTAGAGGTGGGCGGAATTGATAGTGTAGCGGTGAAATGCATAGATATTATCAAGAACTCCAATTGCGTAGGCAGCTCACTTGGCTTTTACTGACACTCATGCACGAAAGTGTGGGTATCAAACAGG
## 170                                                          ACAGAGGGTGCAAGCGTTAATCGGAATTACTGGGCGTAAAGCGCACGTAGGCGGTTTTTTAAGTCAGATGTGAAAGCCCCGGGCTCAACCTGGGAATTGCATTTGAAACTGGAAAACTAGAGTGTGTGAGAGGGGGGTAGAATTCCAAGTGTAGCGGTGAAATGCGTAGAGATTTGGAGGAATACCAGTGGCGAAGGCGGCCCCCTGGCACAACACTGACGCTCAGGTGCGAAAGCGTGGGGAGCAAACAGG
## 171                                                          ACGTAGGGTGCGAGCGTTAATCGGAATTACTGGGCGTAAAGCGTGCGCAGGCGGCTTTGCAAGACAGATGTGAAATCCCCGGGCTCAACCTGGGAACTGCATTTGTGACTGCAAGGCTAGAGTACGGTAGAGGGGGATGGAATTCCGCGTGTAGCAGTGAAATGCGTAGATATGCGGAGGAACACCAATGGCGAAGGCAATCCCCTGGACCTGTACTGACGCTCATGCACGAAAGCGTGGGGAGCAAACAGG
## 172                                                          ACGTAGGGTGCAAGCGTTAATCGGAATTACTGGGCGTAAAGCGTGCGCAGGCGGTTTTGTAAGCCAGATGTGAAATCCCCGAGCTTAACTTGGGAACTGCGTTTGGAACTACAAGACTAGAGTGTGTCAGAGGGGGGTAGAATTCCACGTGTAGCAGTGAAATGCGTAGAGATGTGGAGGAATACCGATGGCGAAGGCAGCCCCCTGGGATAACACTGACGCTCATGCACGAAAGCGTGGGGAGCAAACAGG
## 173                                                         ACGTAGGATCCAAGCGTTATCCGGAATTACTGGGCGTAAAGCGTGCGCAGACGGTTTGGTAGGTCTGGTGCGAAATCCGGTGGCTCAACCACCTGGACTGTACCAGAAACCCCCTGACTCGAGGTCGGTAGAGGCAAGTGGAATTGCTGGTGTAGGGGTGACATCCGTAGATATCAGCAGGAACACCAATGGCGAAGGCAGCTTGCTGGGCCGTACCTGACGTTCAGGCACGAAAGCGTGGGGAGCGAACAGG
## 174                                                          ACAGAGGATGCAAGCGTTATCCGGAATCACTGGGCATAAAGCGTCTGTAGGTTGCTTGCCAAGTCTGCTGTTAAAGACTAGGGCCTAACCCTGGGAAAGCAGTGGAAACTAGCAAGCTTGAGTGTGGTAGAGGTAGAGGGAATTCCTGGTGTAGCGGTGAAATGCGTAGATATTAGGAAGAACACCAATGGCGAAAGCACTCTGCTGGGCCACAACTGACACTGAGAGACGACAGCTAGGGGAGCAAATGGG
## 175                                                          ACGTAGGGTGCGAGCGTTAATCGGAATTACTGGGCGTAAAGCGTGCGCAGGTGGTTACGTAAGTCTGATGTGAAATCCCCGAGCTTAACTTGGGAACTGCATTGGAGACTGCGTGGCTAGAGTGTGTCAGAGGGGGGTGGAATTCCACGTGTAGCAGTGAAATGCGTAGAGATGTGGAGGAACACCGATGGCGAAGGCAGCCCCCTGGGATAACACTGACACTCATGCACGAAAGCGTGGGGAGCAAACAGG
## 176                                                          ACGTAGGGTGCGAGCGTTAATCGGAATTACTGGGCGTAAAGCGTGCGCAGGCGGCTTTTTAAGCCAGATGTGAAATCCCCGGGCTTAACCTGGGAACTGCATTTGGAACTGGAAGGCTAGAGTGCGGCAGAGGGGGGTAGAATTCCACGTGTAGCAGTGAAATGCGTAGAGATGTGGAGGAATACCGATGGCGAAGGCAGCCCCCTGGGTCGACACTGACGCTCATGCACGAAAGCGTGGGGAGCAAACAGG
## 177                                                          ACGGAGGGTGCAAGCGTTATCCGGAATCACTGGGTTTAAAGGGTGCGTAGGCGGCCAGATAAGTCAGAGGTGAAAGTTTCCGGCTCAACCGGGAGATTGCCTTTGATACTGTTTGGCTTGAATCAGATTGAGGTTGGCGGAATGTGACATGTAGCGGTGAAATGCATAGATATGTCATAGAACATCGATTGCGAAGGCAGCTGGCTGGATCTGTATTGACGCTGAGGCACGAAAGCGTGGGGAGCAAACAGG
## 178                                                          ACGGAGGGAGCTAGCGTTGTTCGGAATTACTGGGCGTAAAGAGTACGTAGGCGGTTATTCAAGTCAGAGGTGAAAGCCTGGAGCTCAACTCCAGAACTGCCTTTGAAACTAGATAGCTAGAATTATGGAGAGGTTAGTGGAATTCCGAGTGTAGAGGTGAAATTCGTAGATATTCGGAAGAACACCAGTGGCGAAGGCGACTAACTGGACATATATTGACGCTGAGGTACGAAAGCGTGGGGAGCAAACAGG
## 179                                                          ACGGAGGGTGCAAGCGTTAATCGGAATTACTGGGCGTAAAGCGCGCGTAGGCGGCTGATTAAGTCGGATGTGAAAGCCCCGGGCTCAACCTGGGAACTGCATACGATACTGATCGGCTAGAGTATGAGAGAGGGAGGTAGAATTCCACGTGTAGCGGTGAAATGCGTAGATATGTGGAGGAATACCGGTGGCGAAGGCGGCCTCCTGGCTTAATACTGACGCTGAGGTGCGAAAGCGTGGGGAGCAAACAGG
## 180                                                          ACGGAGGGTGCAAGCGTTACTCGGAATCACTGGGCGTAAAGGATGCGTAGGCTGGAAATCAAGTCGAGAGTGAAATCCAACGGCTCAACCGTTGAACTGCTCTCGAAACTGGTTACCTAGAATATGGGAGAGGTAGATGGAATTGGTGGTGTAGGGGTAAAATCCGTAGATATCACCAGGAATACCGATTGCGAAGGCGATCTACTGGAACATTATTGACGCTGAGGCATGAAAGCGTGGGGAGCAAACAGG
## 181                                                          ACGTAGGGTGCAAGCGTTAATCGGAATTACTGGGCGTAAAGCGTGCGCAGGCGGTTATATAAGTCAGATGTGAAATCCCCGGGCTCAACCTGGGAACTGCCTTTGTGACTGCATAGCTAGAGTACGGCAGAGGGGGATGGAATTCCGCGTGTAGCAGTGAAATGCGTAGATATGCGGAGGAACACCGATGGCGAAGGCAATCCCCTGGGCCTGTACTGACGCTCATGCACGAAAGCGTGGGGAGCAAACAGG
## 182                                                          ACGAAGGGTGCAAGCGTTAATCGGAATTACTGGGCGTAAAGCGCGCGTAGGTGGTTCAGCAAGTTGGAGGTGAAATCCCCGGGCTCAACCTGGGAACTGCCTCCAAAACTACTGAGCTAGAGTACGGTAGAGGGTAGTGGAATTTCCTGTGTAGCGGTGAAATGCGTAGATATAGGAAGGAACACCAGTGGCGAAGGCGACTACCTGGACTGATACTGACACTGAGGTGCGAAAGCGTGGGGAGCAAACAGG
## 183                                                          ACGTAGGGTGCAAGCGTTAATCGGAATTACTGGGCGTAAAGCGTGCGCAGGCGGTTATGTAAGACAGATGTGAAATCCCCGGGCTCAACCTGGGAACTGCATTTGTGACTGCATAGCTGGAGTGCGGCAGAGGGGGATGGAATTCCGCGTGTAGCAGTGAAATGCGTAGATATGCGGAGGAACACCGATGGCGAAGGCAATCCCCTGGGCCTGCACTGACGCTCATGCACGAAAGCGTGGGGAGCAAACAGG
## 184                                                          ACATAGGTCCCAAGCGTTATCCGGAATTACTGGGCGTAAAGCGTCTGCAGGTTGAAAAGTGTGTGAGATGTGAAAGACCGGGGCTCAACCCCGCGTTTGTGTCTCAAACTGCTTTTCTCGAGTGAGCAAGAGGTATGCGGAATTTATGGAGTAGGAGTGCAATCCGTTGACACCATAAAGAACACCAAAAGCGAAGGCAGCATACTGGGGCTCTACTGACACTCAGAGACGAAAGCGTGGGGAGCGAAAGGG
## 185                                                          ACGGAGGATCCAAGCGTTATCCGGAATCATTGGGTTTAAAGGGTCCGTAGGCGGTTTAATAAGTCAGTGGTGAAATCTGGTCGCTCAACGATCAAACGGCCATTGATACTGTTAGACTTGAATTACTAGGAAGTAACTAGAATATGTAGTGTAGCGGTGAAATGCTTAGATATTACATGGAATACCAATTGCGAAGGCAGGTTACTACTAGTGGATTGACGCTGATGGACGAAAGCGTGGGGAGCGAACAGG
## 186                                                          ACGTAGGGTGCGAGCGTTAATCGGAATTACTGGGCGTAAAGCGTGCGCAGGCGGTTGTGTAAGTCAGATGTGAAATCCCCGGGCTCAACCTGGGAACTGCATTTGAGACTGCACGGCTAGAGTGTGACAGAGGGGGGTAGAATTCCACGTGTAGCAGTGAAATGCGTAGAGATGTGGAGGAATACCGATGGCGAAGGCAGCCCCCTGGGTTACTACTGACGCTCATGCACGAAAGCGTGGGGAGCAAACAGG
## 187                                                          ACGGAGGGTGCAAGCGTTGTCCGGATTTATTGGGTTTAAAGGGTGCGCAGGTGGTTTATTAAGTCAGTGGTGAAAGACGGTCGCTCAACGATTGCAGTGCCATTGAAACTAGTAGACTTGAGTAAAGTAGAGGTGGGCGGAATTGATAGTGTAGCGGTGAAATGCATAGATATTATCAAGAACTCCAATTGCGTAGGCAGCTCACTTGGCTTTTACTGACACTCATGCACGAAAGTGTGGGTATCAAACAGG
## 188                                                          ACGGAGGGGGCTAGCGTTGTTCGGAATTACTGGGCGTAAAGGGCGCGTAGGCGGATTAGTAAGTTGGGAGTGAAAGCCCGGGGCTTAACCTCGGAACTGCTTTCAAAACTGCTAGTCTTGAGTGAAGTAGGGGATGATGGAATTCCTAGTGTAGAGGTGAAATTCTTAGATATTAGGAGGAACACCGGTGGCGAAGGCGGTCATCTGGACTTCAACTGACGCTGAGGCGCGAAAGCGTGGGGAGCAAACAGG
## 189                                                          ACGGAGGATCCGAGCGTTATCCGGATTTATTGGGTTTAAAGGGAGCGTAGGTTGACATATAAGTCAGCTGTGAAAGTTTACGGCTCAACCGTGAAATTGCAGTTGATACTGTATGTCTTGAGTGTACAAGAGGTGGGCGGAATTCGTGGTGTAGCGGTGAAATGCTTAGATATCACGAAGAACTCCAATTGCGAAGGCAGCTCACTGGGGTACAACTGACACTGAGGCTCGAAAGTGTGGGTATCAAACAGG
## 190                                                          ACATAGGGTGCAAGCGTTGTCCGGAATTATTGGGCGTAAAGAGCTCGTAGGTGGTTCGTTACGTCGGATGTGAAAATCTGGGGCTCAACCCCAGACCTGCATCCGATACGGTCGAGCTAGAGTTTGGTAGGGGAGACTGGAATTCCTGGTGTAGCGGTGGAATGCGCAGATATCAGGAGGAACACCAATGGCGAAGGCAGGTCTCTGGGCCAATACTGACACTGAGGAGCGAAAGCGTGGGGAGCGAACAGG
## 191                                                          ACAGAGACTGCAAGCGTTACTCGGATTCACTGGGCGTAAAGGGAGCGCAGGCGGGCGGGTGTGTCATATGTGAAATCCCGGGGCTCAACCCCGGGGCTGCATGTGAAACTACCTGTCTAGAGATTCGGAGGGGTAAGCGGAATTCCTGGTGGAGCAGTGAAATGCGTAGATATCAGGAGGAACACCAACGGCGAAGGCAGCTTACTGGACGAAATCTGACGCTCAGGCTCGAAAGCATGGGGAGCAAAAGGG
## 192                                                          ACGGAGGATCCAAGCGTTATCCGGATTTATTGGGTTTAAAGGGTCCGTAGGCGGGTCTTTAAGTCAGTGGTGAAAGCCTGCAGCTTAACTGTGGAAATGCCATTGATACTGGAGGCCTTGAGTGTAGTGGAAGTAGGCGGAATAGGGCATGTAGCGGTGAAATGCATAGATATGCCCTAGAACACCGATTGCGAAGGCAGCTTACTACGTTACAACTGACGCTGAGGGACGAAAGCGTGGGGAGCAAACAGG
## 193                                                          ACGGAGGGTGCAAGCGTTAATCGGAATTACTGGGCGTAAAGCGTGCGTAGGCGGCTCTTTAAGTCAGATGTGAAATCCCCGGGCTCAACCTGGGAACTGCATCTGATACTGGGGAGCTAGAATGTGGGAGAGGGAAGTGGAATTTCCGGTGTAGCGGTGAAATGCATAGAGATCGGAAGGAACATCAGTGGCGAAGGCGACTTCCTGGACCAACATTGACGCTGAGGCACGAAAGCGTGGGTAGCAAACAGG
## 194                                                          ACATAGGGTGCAAGCGTTGTCCGGAATTATTGGGCGTAAAGAGCTCGTAGGTGGTTTGTTACGTCGGTTGTGAAATTCAGGGGCTCAACCCCTGACTTGCAGCCGATACGGGCAAGCTAGAGTTTGGTAGGGGAGACTGGAATTCCTGGTGTAGCGGTGGAATGCGCAGATATCAGGAGGAACACCGATGGCGAAGGCAGGTCTCTGGGCCAATACTGACACTGAGGAGCGAAAGCGTGGGGAGCGAACAGG
## 195                                                          ACAGAGGGTGCAAGCGTTAATCGGAATTACTGGGCGTAAAGCGTGCGTAGACGGTTATTTAAGTCGGATGTGAAATCCCCGGGCTCAACCTGGGAATTGCATTCGAGACTGAATAGCTAGGGTGCGGAAGAGGGAAGCGGAATTTCCGGTGTAGCGGTGAAATGCGTAGATATCGGAAGGAACATCAGTGGCGAAAGCGGCTTCCTGGTCCAGCACCGACGTTCAGGCACGAAAGCGTGGGGAGCAAACAGG
## 196                                                          ACGGAGGGGGCTAGCGTTGTTCGGAATTACTGGGCGTAAAGCGCACGTAGGCGGACTGGAAAGTCAGAGGTGAAATCCCAGGGCTCAACCTTGGAACTGCCTTTGAAACTCCCAGTCTTGAGGTCGAGAGAGGTGAGTGGAATTCCGAGTGTAGAGGTGAAATTCGTAGATATTCGGAGGAACACCAGTGGCGAAGGCGGCTCACTGGCTCGATACTGACGCTGAGGTGCGAAAGCGTGGGGAGCAAACAGG
## 197                                                          ACGGAGGATCCAAGCGTTATCCGGAATCATTGGGTTTAAAGGGTCCGTAGGCGGCCTTATAAGTCAGTGGTGAAATCTCCCGGCTCAACCGGGAAATGGCCATTGATACTGTAGGGCTTGAATTATTAGGAAGTAACTAGAATATGTAGTGTAGCGGTGAAATGCTTAGAGATTACATGGAATACCAATTGCGAAGGCAGGTTACTACTAATGGATTGACGCTGATGGACGAAAGCGTGGGTAGCGAACAGG
## 198                                                          ACAGAGACTGCAAGCGTTACTCGGATTCACTGGGCGTAAAGGGAGCGCAGGCGGGCGGGTGTGTCATATGTGAAATCCCGGGGCTCAACCCCGGGGCTGCATCTGAAACTACCTGTCTAGAGATTCGGAGGGGTAAGCGGAATTCCTGGTGGAGCAGTGAAATGCGTAGATATCAGGAGGAACACCAACGGCGAAGGCAGCTTACTGGACGAAATCTGACGCTCAGGCTCGAAAGCATGGGGAGCAAAAGGG
## 199                                                          ACGAGTGGCCCAAGCGTTATCCGGAATTATTGGGCGTAAAGGATGTGTAGGTGGTTGTATTAGTCTTTGGTCTAACCTCTGTGCTTAACATGGAGACAGCCGAGGAAACGGTACGACTAGAGTAGAGTAGGGGTAAGCGGAACTCATAGTGTAGGGGTGAAATCCGTTGATATTATGGGGAACACCAAAAGCGAAGGCAGCTTACTGGACTCTTACTGACACTGAAACATGAAAGCGTGGGTAGCGAATGGG
## 200                                                          ACGAGTGCCTCAAGCGTTATCCGGAATCATTGGGCGTAAAGGATGTGTAGGTGGTCGTGTTAGTCTCTCGTTAAATTCTTCGGCTTAACCGGGGGCATGCGGGGGAAACGGCACGACTAGAGGGTGCAAGGGGTCTATGGAACTCATAGTGTAGCGGTGAAATGCGTTGATATTATGGGGAACACCAAAAGCGAAGGCAGTAGACTAGGGCACTCCTGACACTGAAACATGAAAGCGTGGGTAGCGAATGGG
## 201                                                          ACGGAGGGGGCTAGCGTTATTCGGAATTACTGGGCGTAAAGCGCACGTAGGCGGATTAGAAAGTCAGAGGTGAAATCCCAGGGCTCAACCTTGGAACTGCCTTTGAAACTCCTAGTCTTGAGGTCGAGAGAGGTGAGTGGAATTCCGAGTGTAGAGGTGAAATTCGTAGATATTCGGAGGAACACCAGTGGCGAAGGCGGCTCACTGGCTCGATACTGACGCTGAGGTGCGAAAGCGTGGGGAGCAAACAGG
## 202                                                          ACGGAGGATGCAAGCGTTATCCGGATTTATTGGGTTTAAAGGGTGCGTAGGCGGTTGGATAAGTCAGCGGTGAAAGTTTTCTGCTTAACAGGAAAAATGCCGTTGATACTGTCTGGCTAGAATATGGTTGCTGTGAGTGGAATGTGTGGTGTAGCGGTGAAATGCTTAGATATCACACAGAATATCGATTGCGAAGGCAGCTCACAAAGCCATTATTGACGCTGAGGCACGAAAGTGTGGGGATCAAACAGG
## 203                                                          ACGGAGGGTGCGAGCGTTGTCCGGAATCACTGGGCGTAAAGGGCGCGTAGGCGGTCTGCTAAGCGTGTGGTGAAAGCTCGGGGCTCAACCCTGAGTCGGCCATGCGAACTGGTGGACTTGAGCACTGTAGAGGCAGGTGGAATTCCGGGTGTAGCGGTGGAATGCGTAGAGATCCGGAAGAACACCGGTGGCGAAGGCGGCCTGCTGGGCAGTAGCTGACGCTGAGGCGCGACAGCGTGGGGAGCAAACAGG
## 204                                                          ACGAAGGGTGCAAGCGTTGTTCGGAATTACTGGGCGTAAAGCGCGCGTAGGCGGTCTACTAAGTCAGACGTGAAAGCCCTGGGCTTAACCCGGGAATGGCGTTTGATACTGGTAGACTAGAGATTCAGAGAGGTGAGTGGAATTCCTAGTGTAGAGGTGAAATTCGTAGATATTAGGAGGAACATCAGTGGCGAAGGCGGCTCACTGGCTGAATACTGACGCTAAGGCGCGAAAGCGTGGGGAGCAAACAGG
## 205                                                          ACGGAGGGTGCAAGCGTTAATCGGAATTACTGGGCGTAAAGCGCACGCAGGCGGTCTGTCAAGTCGGATGTGAAATCCCCGGGCTCAACCTGGGAACTGCATTCGAAACTGGCAGGCTAGAGTCTTGTAGAGGGGGGTAGAATTCCAGGTGTAGCGGTGAAATGCGTAGAGATCTGGAGGAATACCGGTGGCGAAGGCGGCCCCCTGGACAAAGACTGACGCTCAGGTGCGAAAGCGTGGGGAGCAAACAGG
## 206                                                          ACGTAGGGTGCGAGCGTTAATCGGAATTACTGGGCGTAAAGCGTGCGCAGGCGGTTATGCAAGACAGAGGTGAAATCCCCGGGCTCAACCTGGGAACTGCCTTTGTGACTGCATAGCTAGAGTACGGCAGAGGGGGATGGAATTCCGCGTGTAGCAGTGAAATGCGTAGATATGCGGAGGAACACCGATGGCGAAGGCAATCCCCTGGGCCTGTACTGACGCTCATGCACGAAAGCGTGGGGAGCAAACAGG
## 207                                                          ACGGAGGGTGCAAGCGTTATCCGGATTCACTGGGTTTAAAGGGAGCGTAGGTGGGCTTTTAAGTCAGTGGTGAAATCTTTGGGCTTAACCCGAAAACTGCCATTGATACTATTAGTCTTGAATTCTCTGGAGGTAAGCGGAATATGTCATGTAGCGGTGAAATGCTTAGATATGACATAGAACACCCATTGCGAAGGCAGCTTACTACGGAGACATTGACACTGAGGCTCGAAAGCGTGGGGATCAAACAGG
## 208                                                          ACGAAGGGTGCAAGCGTTAATCGGAATTACTGGGCGTAAAGCGCGCGTAGGTGGTTTTGTAAGTTGGAGGTGAAATCCCCGGGCTCAACCTGGGAACTGCCTCCAAAACTGCATGACTAGAGTACGGTAGAGGGTGGTGGAATTTCCTGTGTAGCGGTGAAATGCGTAGATATAGGAAGGAACACCAGTGGCGAAGGCGACCACCTGGACTGATACTGACACTGAGGTGCGAAAGCGTGGGGAGCAAACAGG
## 209                                                          ACGGAGGGTGCAAGCGTTAATCGGAATAACTGGGCGTAAAGCGCACGCAGGCGGTTGGATAAGTCAGATGTGAAAGCCCCGGGCTCAACCTGGGAACTGCATTTGAAACTGTCTGACTAGAGTCTTGTAGAGGGGGGTAGAATTCCAGGTGTAGCGGTGAAATGCGTAGAGATCTGGAGGAATACCGGTGGCGAAGGCGGCCCCCTGGACAAAGACTGACGCTCAGGTGCGAAAGCGTGGGGAGCAAACAGG
## 210                                                          ACAGAGGGTGCAAGCGTTAATCGGAATTACTGGGCGTAAAGCGCACGTAGGCGGTTGTTTAAGTTGGATGTGAAAGCCCCGGGCTCAACCTGGGAATTGCATTCAAAACTGGACAACTAGAGTGTGTGAGAGGGGGGTAGAATTCCAAGTGTAGCGGTGAAATGCGTAGAGATTTGGAGGAATACCAGTGGCGAAGGCGGCCCCCTGGCACAACACTGACGCTCAGGTGCGAAAGCGTGGGGAGCAAACAGG
## 211                                                          ACGTAGGGTGCGAGCGTTAATCGGAATTACTGGGCGTAAAGCGTGCGCAGGCGGTTATATAAGACAGTTGTGAAATCCCCGGGCTCAACCTGGGAATTGCATCTGTGACTGTGTAGCTAGAGTACGGTAGAGGGGGATGGAATTCCGCGTGTAGCAGTGAAATGCGTAGATATGCGGAGGAACACCGATGGCGAAGGCAATCCCCTGGACCTGTACTGACGCTCATGCACGAAAGCGTGGGGAGCAAACAGG
## 212                                                          ACATAGGGTGCAAGCGTTGTCCGGAATTATTGGGCGTAAAGAGCTCGTAGGTGGTTAGATACGTCGGATGTGAAAATCAGGGGCTCAACCCCTGACCTGCATTCGATACGGTCTAGCTAGAGTGTGGTAGGGGAGACTGGAATTCCTGGTGTAGCGGTGGAATGCGCAGATATCAGGAGGAACACCGATGGCGAAGGCAGGTCTCTGGGCCATTACTGACACTGAGGAGCGAAAGCGTGGGGAGCGAACAGG
## 213                                                          ACGAAGGATGCAAGCGTTATCCGGATTCATTGGGTTTAAAGGGTGCGTAGGCGGACTTGTAAGTCAGTGGTGAAATCTCTTTGCTTAACAAAGAAACTGCCATTGATACTGCAGGTCTAGAGTATAGATGACGTTGGCGGAATATGACATGTAGTGGTGAAATACTTAGATATGTCATAGAACACCGATTGCGAAGGCAGCTAACGAAACTATAACTGACGCTGAGGCACGAAAGTGCGGGGATCAAACAGG
## 214                                                          ACGTAGGGTGCAAGCGTTAATCGGAATTACTGGGCGTAAAGCGTGCGCAGGCGGTTATGTAAGACAGAGGTGAAATCCCCGGGCTCAACCTGGGAACTGCCTTTGTGACTGCATAGCTTGAGTGCGGCAGAGGGGGATGGAATTCCGCGTGTAGCAGTGAAATGCGTAGATATGCGGAGGAACACCGATGGCGAAGGCAATCCCCTGGGCCTGCACTGACGCTCATGCACGAAAGCGTGGGGAGCAAACAGG
## 215                                                          ACGGAGGGTGCAAGCGTTATCCGGATTTATTGGGTTTAAAGGGTCCGTAGGCGGGCCGATAAGTCAGTGGTGAAAGCCCATAGCTCAACTATGGAACTGCCATTGATACTGTCGGTCTTGAGTACAAGTGAGGTTGGCGGAATGTGTGGTGTAGCGGTGAAATGCTTAGATATCACACAGAACACCGATTGCGAAGGCAGCTGGCCAACTTGTAACTGACGCTGAGGGACGAAAGTGTGGGGATCAAACAGG
## 216                                                          ACGGAAGGTCCAGGCGTTATCCGGATTTATTGGGTTTAAAGGGAGCGTAGGCCGTCTGTTAAGCGTGTTGTGAAATGTAGATGCTCAACATCTGAATTGCAGCGCGAACTGGCAGACTTGAGTGTGCGCAACGTAGGCGGAATTCGTGGTGTAGCGGTGAAATGCTTAGATATCACGAAGAACTCCGATTGCGAAGGCAGCTTACGGGAGCACAACTGACGCTGAAGCTCGAAAGTGCGGGTATCGAACAGG
## 217                                                          ACGAAGGGTGCAAGCGTTGCTCGGAATTATTGGGCGTAAAGGGTAGGTAGGTGGTTGCACATGTCTGGGGTGAAATCCCTGAGCTTAACTCAGGAAGTGCCTTGGAAACGGTGTAACTAGAGTACTAGAGAGGTTCGTGGAATTCTTGGTGTAGCGGTGAAATGCGTAGAGATCAAGAGGAACATCAGCGGCGAAGGCGGCGAACTGGATAGTAACTGACACTCAACTACGAAAGCGTGGGGAGCAAACAGG
## 218                                                          ACGAGTGCCTCGAGCGTTATCCGGAATTATTGGGCGTAAAGGGTGTGTAGGTGGTTATGTTAGTCTCCTGTCAAATTCTTCGGCTTAACCGGGGGTCCGCAGGGGAAACGGCATGACTTGAGGATGCGAGAGGTAAATGGAACTCATAGTGTAGGGGTAAAATCCGTTGATATTATGGGGAACACCAAAAGCGAAGGCAATTTACTGGCGCACTCCTGACACTGAAACACGAAAGCGTGGGTAGCGAATGGG
## 219                                                          ACGAAGGGGGCTAGCGTTGCTCGGAATTACTGGGCGTAAAGGGCGCGTAGGCGGCTTAGTCAGTCAGGCGTGAAATTCCTGGGCTTAACCTGGGGTCTGCGTTTGAGACGGCTAGGCTAGAGGATAGGAGAGGGTCGTGGAATTCCCAGTGTAGAGGTGAAATTCGTAGATATTGGGAAGAACACCGGTGGCGAAGGCGGCGACCTGGCCTATTACTGACGCTGAGGCGCGACAGCGTGGGGAGCAAACAGG
## 220                                                          ACGTAGGGGGCAAGCGTTATCCGGATTTACTGGGTGTAAAGGGAGCGTAGACGGCGATGCAAGTCAGATGTGAAAACCCATGGCTCAACCATGGGATTGCATTTGAAACTGTGTTGCTAGAGTGCAGGAGAGGTAAGCGGAATTCCTGGTGTAGCGGTGAAATGCGTAGATATCAGGAGGAACACCGGTGGCGAAGGCGGCTTACTAGACTGTAACTGACGTTGAGGCTCGAAGGCGTGGGTAGCAAACAGG
## 221                                                          ACGTAGGGTGCGAGCGTTAATCGGAATTACTGGGCGTAAAGCGTGCGCAGGCGGCGTGACAAGTCAGATGTGAAATCCCCGAGCTCAACTTGGGAACTGCGTTTGAAACTGTCAGGCTAGAATATGTCAGAGGGGGGTAGAATTCCACGTGTAGCAGTGAAATGCGTAGAGATGTGGAGGAATACCAATGGCGAAGGCAGCCCCCTGGGATAATATTGACGCTCATGCACGAAAGCGTGGGGAGCAAACAGG
## 222                                                          ACGAAGGATCCAAGCGTTATCCGGATTCATTGGGTTTAAAGGGTGCGTAGGCGGATAGATAAGTCAGTGGTGAAAGCCGGTCGCTCAACGATCGAATTGCCATTGATACTGTTTATCTAGAATATAGATGATGTTGGCGGAATATGACATGTAGCGGTGAAATGCATAGATATGTCATAGAACACCTATTGCGAAGGCAGCTGACAAAACTATTATTGACGCTGATGCACGAAAGTGCGGGGATCAAACAGG
## 223                                                          ACGTAGGGTGCGAGCGTTAATCGGAATTACTGGGCGTAAAGCGTGCGCAGGCGGTTATGTAAGACAGAGGTGAAATCCCCGGGCTCAACCTGGGAACTGCCTTTGTGACTGCATAGCTGGAGTGCGGCAGAGGGGGATGGAATTCCGCGTGTAGCAGTGAAATGCGTAGATATGCGGAGGAACACCGATGGCGAAGGCAATCCCCTGGGCCTGCACTGACGCTCATGCACGAAAGCGTGGGGAGCAAACAGG
## 224                                                          ACGTAGGCTTCAAGCGTTATCCGGAATTACTGGGTGTAAAGCGTCTGTAGGCGGCAGAGTAAGTTTGGCATGAAAGACCGGGGCTTAACCCCGTGTTTGTGTCGAAAACTGCTCAGCTAGAATCTGGGAGAGGGAAGCAGAATGATGTGAGTAGGGGTGCAATCCGTTGATACACATCAGAATACCAAAAGCGAAGGCAGCTTCCTGGAACAGTATTGACGCTCAGAGACGAAAGCGTGGGGAGCGAAAAGG
## 225                                                          ACGGAGGGTGCAAGCGTTACTCGGAATCACTGGGCGTAAAGAGCGTGTAGGCGGATTGATAAGTTTGAAGTGAAATCCTATAGCTTAACTATAGAACTGCTTTGAAAACTGTTAATCTAGAATGTGGGAGAGGTAGATGGAATTTCTGGTGTAGGGGTAAAATCCGTAGAGATCAGAAGGAATACCGATTGCGAAGGCGATCTACTGGAACAATATTGACGCTGAGACGCGAAAGCGTGGGGAGCAAACAGG
## 226                                                          ACGGAGGGTGCAAGCGTTGTCCGGATTTATTGGGTTTAAAGGGTGCGTAGGCGGCTATTTAAGTCAGCGGTGAAAGACTTCAGCTTAACTGGAGCAGTGCCATTGATACTGGATAGCTTGAGTGTTGGAGGGGTACATGGAATTGATGGTGTAGCGGTGAAATGCATAGATACCATCAGGAACACCGATAGCGAAGGCATTGTACTGGCCAACAACTGACGCTGAGGCACGAAAGTGTGGGGATCGAACAGG
## 227                                                          ACGTAGGGTGCAAGCGTTAATCGGAATTACTGGGCGTAAAGCGTGCGTAGACGGTTATCTAAGTCGGATGTGAAATCCCCGGGCTCAACCTGGGAATTGCATTCGAGACTGAATAGCTAGGGTGCGGAAGAGGGAAGCGGAATTTCCGGTGTAGCGGTGAAATGCGTAGATATCGGAAGGAACATCAGTGGCGAAAGCGGCTTCCTGGTCCAGCACCGACGTTCAGGCACGAAAGCGTGGGGAGCAAACAGG
## 228                                                          ACGTAGGGTGCGAGCGTTAATCGGAATTACTGGGCGTAAAGCGTGCGCAGGCGGTTATGTAAGACAGATGTGAAATCCCCGGGCTCAACCTGGGACCTGCATTTGTGACTGCATAGCTAGAGTACGGTAGAGGGGGATGGAATTCCGCGTGTAGCAGTGAAATGCGTAGATATGCGGAGGAACACCGATGGCGAAGGCAATCCCCTGGACCTGTACTGACGCTCATGCACGAAAGCGTGGGGAGCAAACAGG
## 229                                                          ACGTAGGGTGCAAGCGTTAATCGGAATTACTGGGCGTAAAGCGTGCGCAGGTGGTTTGTTAAGACAGTTGTGAAATCCCCGGGCTCAACCTGGGAACTGCAATTGTGACTGACAGACTAGAGTTTGGCAGAGGGGGGTGGAATTCCTGGTGTAGCAGTGAAATGCGTAGATATCAGGAGGAACACCGATGGCGAAGGCAGCCCCCTGGGCCATGACTGACACTCATGCACGAAAGCGTGGGGAGCAAACAGG
## 230                                                          ACGGAAGGTCCGGGCGTTATCCGGATTTATTGGGTTTAAAGGGAGCGTAGGCGGAGCGTCAAGCCAGCAGTGAAATGTAGCGGCCCAACCGCTGCACTGCTGTTGGAACTGGCGCCCTTGAGTGCACACGAGGCATGCGGAATTTGTGGTGTAGCGGTGAAATGCTTAGATATCACGAAGAACTCCGATTGCGAAGGCAGCGTGCCGGAGTGCAACTGACGCTGAAGCTCGAAAGTGCGGGTATCGAACAGG
## 231                                                          ACGGAGGGTGCAAGCGTTATCCGGAATCATTGGGTTTAAAGGGTCCGCAGGCGGGCTTATAAGTCAGTGGTGAAAGCCTACAGCTTAACTGTAGAACTGCCATTGATACTGTAAGTCTTGAATTCGGTCGAAGTGGGCGGAATGTGTAGTGTAGCGGTGAAATGCTTAGATATTACACAGAACACCGATAGCGAAGGCAGCTCACTAGGCCTGAATTGACGCTCATGGACGAAAGCGTGGGGAGCAAACAGG
## 232                                                          ACGGGAGTGGCAAGCGTTATCCGGAATTATTGGGCGTAAAGCGTCCGCAGGCGGTCTTGTAAGTCTGTTGTTAAAGCGTGGAGCTTAACTCCATTTCAGCAATGGAAACTGTAAGACTAGAGTGTGGTAGGGGCAGAGGGAATTCCCGGTGTAGCGGTGAAATGCGTAGATATCGGGAAGAACACCAGTGGCGAAGGCGCTCTGCTGGGCCATAACTGACGCTCATGGACGAAAGCCAGGGGAGCGAAAGGG
## 233                                                          ACAGAGGGTGCGAGCGTTAATCGGAATTACTGGGCGTAAAGCGTGCGTAGGCGGTTTGATAAGCTGGATGTGAAATCCCCGGGCTTAACCTGGGAACTGCATTCAGGACTGTTTGACTAGAGTCTGGGAGAGGGTGGTGGAATTTCCTGTGTAGCGGTGAAATGCGTAGATATAGGAAGGAACATCAGTGGCGAAGGCGACTGCCTGGCCCAAGACTGACGCTGAGGCACGAAAGCGTGGGTAGCAAACAGG
## 234                                                          ACAGAGGGTGCAAGCGTTGTTCGGACTTACTGGGCGTAAAGCGCGTGTAGGCGGTCTTTTAAGTCTGGTGTGAAAGCCTGAGGCTTAACCTCAGAAGTGCACTGGATACTGGAAGACTAGAGGGTGAAAGAGGAGAGTGGAATTCATGGTGTAGGGGTGAAATCCGTAGATATCATGAGGAACATCAGTGGCGAAGGCGGCTCTCTGGTTCACTACTGACGCTCAGACGCGAAAGCATGGGGAGCAAACAGG
## 235                                                          ACGAGTGCTCCGAGCGTTATCCGGAATCATTGGGCGTAAAGGGTGTGTAGGTGGTAATACTAGTCTTCAGTTAAATTCTTCGGCTCAACCGGGGACATGCTGGGGAAACGGTATTACTAGAGGATGCGAGAGGTGTGTGGAACTCTATGTGTAGGGGTGAAATCCGTTGATATATAGGGGAACACCAAAAGCGAAGGCAGCACACTGGCGCACTCCTGACACTGAAACACTAAAGCGTGGGTAGCGAATGGG
## 236                                                          ACGGAGGGTGCAAGCGTTATCCGGATTTACTGGGTTTAAAGGGTGCGTAGGCGGGCAGGTAAGTCAGTGGTGAAATCCCCGAGCTTAACTCGGGAACTGCCGTTGATACTATCTGTCTTGAATATAGTGGAGGTAAGCGGAATATGTCATGTAGCGGTGAAATGCTTAGATATGACATAGAACACCAATTGCGAAGGCAGCTTGCTACACTATCATTGACGCTGAGGCACGAAAGCGTGGGGATCAAACAGG
## 237                                                          ACGTAGGGTGCGAGCGTTAATCGGAATTACTGGGCGTAAAGCGTGCGCAGGCGGTTTCGTAAGACAGACGTGAAATCCCCGGGCTCAACCTGGGAACTGCGTTTGTGACTGCGAGGCTAGAGTACGGCAGAGGGGGGTAGAATTCCACGTGTAGCAGTGAAATGCGTAGAGATGTGGAGGAATACCGATGGCGAAGGCAGCCCCCTGGGTTAGTACTGACGCTCATGCACGAAAGCGTGGGGAGCAAACAGG
## 238                                                          ACAGAGGGTGCAAGCGTTAATCGGAATGACTGGGCGTAAAGCGTGCGTAGGTGGCAAAATAAGTTAGTTGTGAAATCCCTGGGCTTAACCTAGGAACTGCAATTGATACTGTTTAGCTAGAGTATAGTAGAGGTAAGTGGAATTTCCGGTGTAGCGGTGAAATGCGTAGATATCGGAAGGAACACCAGTGGCGAAGGCGGCTTACTGGGCTAATACTGACACTGAGGCACGAAAGCGTGGGGAGCAAACAGG
## 239                                                          ACGTAGGGTGCGAGCGTTAATCGGAATTACTGGGCGTAAAGCGTGCGCAGGCGGTTATGTAAGACAGATGTGAAATCCCCGGGCTCAACCTGGGAACTGCGTTTGTGACTGCATAACTAGAGTACGGCAGAGGGAGGTGGAATTCCGCGTGTAGCAGTGAAATGCGTAGAGATGCGGAGGAACACCGATGGCGAAGGCAGCCTCCTGGGCCAGTACTGACGCTCATGCACGAAAGCGTGGGGAGCAAACAGG
## 240                                                          ACGTAGGGTCCAAGCGTTAATCGGAATTACTGGGCGTAAAGCGTGCGCAGGCGGCTGATTAAGCCAGATGTGAAATCCCCGGGCTCAACCTGGGAACTGCGTTTGGAACTGGTCAGCTAGAGTACGTCAGAGGGGGGTAGAATTCCACGTGTAGCAGTGAAATGCGTAGAGATGTGGAGGAATACCGATGGCGAAGGCAGCCCCCTGGGATGATACTGACGCTCATGCACGAAAGCGTGGGGAGCAAACAGG
## 241                                                          ACGTAGGGTGCGAGCGTTAATCGGAATTACTGGGCGTAAAGCGTGCGCAGGCGGTTTCTTAAGCCAGATGTGAAATCCCCGGGCTTAACCTGGGAACTGCATTTGGAACTGGGAGACTAGAGTGTGTCAGAGGGAGGTGGAATTCCACGTGTAGCAGTGAAATGCGTAGAGATGTGGAGGAACACCGATGGCGAAGGCAGCCTCCTGGGATGACACTGACGCTCATGCACGAAAGCGTGGGGAGCAAACAGG
## 242                                                          ACGTAGGGTGCAAGCGTTAATCGGAATTACTGGGCGTAAAGCGTGCGCAGGCGGTTTTGTAAGACAGGCGTGAAATCCCCGGGCTTAACCTGGGAATTGCGCTTGTGACTGCAAGGCTAGAGTACGGTAGAGGGGGGTAGAATTCCGCGTGTAGCAGTGAAATGCGTAGATATGCGGAGGAACACCGATGGCGAAGGCAGCCCCCTGGACCTGTACTGACGCTCATGCACGAAAGCGTGGGGAGCAAACAGG
## 243                                                      ACAGAGACCTCAAACGTTATCCGGAATCATTGGGCGTAAAGCGTACCGATAGGTGGTTTTATAAGTCAGAAGTGAAATCCTAAGGCTTAACCTTGGGACTGTCTTTTGAAACTATAAAACTAGAGGGGCAAAGAGGAAGCTGGAACAAACGGTGTAGTAGTGAAATGCGTTGATATCGTTTGGAACACCAATAGCGTAGGCAGGCTTCTGGGTGCCACCTGACACTGCTAGGACGAAAGCGTGGGGAGCGATAAGG
## 244                                                        ACGTAGGGTGCAAGCATTATCCGGAGTGACTGGGCGTAAAGAGTTGCGTAGGTGGTTATGTAAGTGAATAGTGAAACCTGGTGGCTCAACCATACAGACTATTATTCAAACTGCATAACTCGAGAGTGGTAGAGGTAACTGGAATTTCTTGTGTAGGAGTGAAATCCGTAGATATAAGAAGGAACACCAATGGCGTAGGCAGGTTACTGGACCATTTCTGACACTAAGGCACGAAAGCGTGGGGAGCGAACCGG
## 245                                                          ACGGAGGGTGCAAGCGTTATCCGGATTCACTGGGTTTAAAGGGTGCGTAGGCGGGCAGGTAAGTCAGTGGTGAAATCCCCGAGCTTAACTCGGGAACTGCCGTTGATACTATCTGTCTTGAATACCGTGGAGGTGAGCGGAATATGTCATGTAGCGGTGAAATGCTTAGATATGACATAGAACACCAATTGCGAAGGCAGCTCGCTACACGGTCATTGACGCTGAGGCACGAAAGCGTGGGGATCAAACAGG
## 246                                                          ACGTAGGGTGCGAGCGTTAATCGGAATTACTGGGCGTAAAGCGTGCGCAGGCGGTTTTGTAAGACAGGCGTGAAATCCCCGGGCTCAACCTGGGAACTGCGCTTGTGACTGCATCACTCGAGTACGGCAGAGGGGGGTGGAATTCCACGTGTAGCAGTGAAATGCGTAGAGATGTGGAGGAACACCGATGGCGAAGGCAGCCCCCTGGGCCGATACTGACGCTCATGCACGAAAGCGTGGGTAGCAAACAGG
## 247                                                          ACGGAGGATGCAAGTGTTATCCGGAATCACTGGGCGTAAAGCGTCTGTAGGTGGTTTGATAAGTCAACTGTTAAATCTTGAAGCTCAACTTCAAAATCGCAGTCGAAACTATTAGACTAGAGTATAGTAGGGGTAAAGGGAATTTCCAGTGGAGCGGTGAAATGCGTAGATATTGGAAAGAACACCGATGGCGAAGGCACTTTACTGGGCTATTACTGACACTCAGAGACGAAAGCTAGGGTAGCAAATGGG
## 248                                                          ACGGAGGGTGCGAGCGTTAATCGGAATTACTGGGCGTAAAGCGCACGTAGGCGGTTCTGTAAGTGGGATGTGAAACCCCCGGGCTTAACCTGGGAATTGCATACCAGACTGCAAAGCTAGAGTATGAGAGAGGGTGGTGGAATTTCCGGTGTAGCGGTGAAATGCGTAGAGATCGGAAGGAACATCAGTGGCGAAGGCGGCCACCTGGCTTAATACTGACGCTGAGGTGCGAAAGCGTGGGGATCAAACAGG
## 249                                                          ACAGAGACTGCAAGCGTTACTCGGATTCACTGGGCGTAAAGGGAGCGCAGGCGGACTCGTGTGTCGGACGTGAAATACCGGGGCTTAACCCCGGTGCTGCGTTCGAAACTACGAGTCTAGAGACTTGGAGGGGTAAGCGGAATTCTTGGTGGAGCAGTGAAATGCGTAGATATCAAGAGGAACACCAACGGCGAAGGCAGCTTACTGGACAAGATCTGACGCTCAGGCTCGAAAGCGTGGGGAGCAAAAGGG
## 250                                                          ACGTAGGGTGCGAGCGTTAATCGGAATTACTGGGCGTAAAGCGTGCGCAGGCGGTTTCGTAAGACAGAGGTGAAATCCCCGGGCTCAACCTGGGAACTGCCTTTGTGACTGCGAGGCTAGAGTATGGCAGAGGGGGGTGGAATTCCACGTGTAGCAGTGAAATGCGTAGATATGCGGAGGAACACCGATGGCGAAGGCAATCCCCTGGGCCTGTACTGACGCTCATGCACGAAAGCGTGGGGAGCAAACAGG
## 251                                                          ACGGAAGGTGCAAGCGTTAATCGGAATTACTGGGCGTAAAGCGCGCGTAGGCGGCTTGGTAAGTCGGATGTGAAAGCCCCGGGCTCAACCTGGGAACTGCATTCGATACTGCCGGGCTGGAGTACGAGAGAGGGGGGTGGAATTCCAGGTGTAGCGGTGAAATGCGTAGAGATCTGGAGGAACATCGGTGGCGAAGGCGGCCCCCTGGTTCGATACTGACGCTGAGGTGCGAAAGCGTGGGGAGCAAACAGG
## 252                                                          ACGTAGGGTGCGAGCGTTAATCGGAATTACTGGGCGTAAAGCGTGCGCAGGCGGTTGTGCAAGACAGATGTGAAATCCCCGGGCTCAACCTGGGAATGGCATTTGTGACTGCACGGCTAGAGTGTGTCAGAGGGGGGTAGAATTCCACGTGTAGCAGTGAAATGCGTAGATATGTGGAGGAATACCGATGGCGAAGGCAGCCCCCTGGGATAACACTGACGCTCATGCACGAAAGCGTGGGGAGCAAACAGG
## 253                                                      ACAGAGACCTCAAGCGTTATCCGGAATCATTGGGCGTAAAGCGTACCGACAGGTGGTCATGCAAGTCAGAGGTGAAATCTCCGAGCTTAACTCGGAATCTGTCCTTTGAAACTGCACGACTAGAGGGGCAAAGAGGAAGCTGGAACAAACGGTGTAGTAGTGAAATGCGTTGATATCGTTTGGAACACCAATAGCGAAGGCGGGCTTCTGGGTGCCACCTGACACTGCTAGGACGAAAGCGTGGGGAGCGAATGGG
## 254                                                          ACGGAGGGTGCAAGCGTTATCCGGATTCACTGGGTTTAAAGGGTGCGTAGGTGGATCTGTAAGTCAGAGGTGAAATCCCCGAGCTTAACTTGGGAACTGCCTTTGATACTATAGATCTTGAATATCGTGGAGGTAAGCGGAATATGTCATGTAGCGGTGAAATGCATAGAGATGACATAGAACACCAATTGCGAAGGCAGCTTGCTACACGATGATTGACACTGAGGCACGAAAGCGTGGGGAGCAAACAGG
## 255                                                          ACGTAGGGTGCGAGCGTTAATCGGAATTACTGGGCGTAAAGCGTGCGCAGGCGGTTCTATAAGACAGATGTGAAATCCCCGGGCTTAACCTGGGAACTGCGTTTGTGACTGTAGGACTCGAGTGTGGCAGAGGGGGGTGGAATTCCACGTGTAGCAGTGAAATGCGTAGAGATGTGGAGGAACACCGATGGCGAAGGCAGCCCCCTGGGTCAACACTGACGCTCATGCACGAAAGCGTGGGGAGCAAACAGG
## 256                                                          ACGGAGGGGGCTAGCGTTGTTCGGAATTACTGGGCGTAAAGCGCACGTAGGCGGATCAGAAAGTCAGAGGTGAAATCCCAGGGCTCAACCTTGGAACTGCCTTTGAAACTCCTGATCTTGAGGTCGAGAGAGGTGAGTGGAATTCCGAGTGTAGAGGTGAAATTCGTAGATATTCGGAGGAACACCAGTGGCGAAGGCGGCTCACTGGCTCGATACTGACGCTGAGGTGCGAAAGCGTGGGGAGCAAACAGG
## 257                                                          ACGGAGGGGGCTAGCGTTGTTCGGAATTACTGGGCGTAAAGCGCACGTAGGCGGACTGGAAAGTCAGAGGTGAAATCCCGGGGCTCAACCCCGGAACTGCCTTTGAAACTCCCAGTCTCGAGTCCGGGAGAGGTGAGTGGAATTCCGAGTGTAGAGGTGAAATTCGTAGATATTCGGAGGAACACCAGTGGCGAAGGCGGCTCACTGGCCCGGTACTGACGCTGAGGTGCGAAAGCGTGGGGAGCAAACAGG
## 258                                                          ACGAGGGGTGCAAGCGTTGTTCGGAATTACTGGGCGTAAAGCGCGTGTAGGTGGTTGAGTATGTCGAATGTGAAAGCCCCAGGCTTAACCTGGGAAGTGCGTTCGAAACTGCTTAACTCGAGTACGGGAGAGGAGGGCGGAATTCCCAGTGTAGAGGTGAAATTCGTAGATATTGGGAGGAACACCGGAGGCGAAAGCGGCTCTCTAGACCGTAACTGACACTGAGACGCGAAAGCGTGGGGATCAAACAGG
## 259                                                          ACGTAGGGTGCGAGCGTTAATCGGAATTACTGGGCGTAAAGCGTGCGCAGGCGGTTTTGAAAGTCAGATGTGAAATCCCCGAGCTCAACTTGGGAACTGCGTTTGAAACTCCAAAACTCGAATATGTCAGAGGGGGGTAGAATTCCACGTGTAGCAGTGAAATGCGTAGAGATGTGGAGGAATACCAATGGCGAAGGCAGCCCCCTGGGATAATATTGACGCTCATGCACGAAAGCGTGGGGAGCAAACAGG
## 260                                                          ACGTAGGGTGCAAGCGTTAATCGGAATTACTGGGCGTAAAGCGTGCGCAGGCGGACTTTTAAGCCAGATGTGAAAGCCCCGAGCTTAACTTGGGAATTGCGTTTGGAACTGGGAGTCTAGAGTCTGTCAGAGGGGGATGGAATTCCACGTGTAGCAGTGAAATGCGTAGAGATGTGGAGGAACACCGATGGCGAAGGCAGCCCCCTGGGCCAATACTGACGCTCATGCACGAAAGCGTGGGGAGCAAACAGG
## 261                                                           ACGGGAGGGGCAAGCGTTATCCAAAATTACTGGGCGTAAAGTGTCCGTAGACTGTAAATTAAGTTTTATGTTAAAATTTAAAGCTTAACTTTAAAAAAATATACAATACTGTTTTACTTGAGTTTTATACGGAAGAGTAGAATTTCATGTCAAAGAGTAAATTCTAAAAATACATGAAGGAATATCGAAAGCGAAGGCGACTCTTTAGTACAAACTGACGTTGAGGGACGAAAGTGTAGGGAGCGAACAGG
## 262                                                          ACGGAGGGTGCAAGCGTTATCCGGAATCATTGGGTTTAAAGGGTCCGCAGGCGGTCTTATAAGTCAGTGGTGAAAGCCTATTGCTCAACAATAGAACTGCCATTGATACTGTTAGACTTGAATTAGGTCGGAGTGGGCGGAATATGACATGTAGCGGTGAAATGCTTAGATATGTCATAGAACACCGATAGCGAAGGCAGCTCACTAGCCCTAAATTGACGCTCATGGACGAAAGCGTGGGGAGCAAACAGG
## 263                                                          ACAGAGGATGCAAGCGTTATCCGGAATCACTGGGCATAAAGCGTCTGTAGGTCGCCTACCAAGTCTGCTGTTAAAGATCAGGGCCTAACCCTGGGAAAGCAGTGGAAACTAGTAGGCTTGAGTATGGTAGAGGTAGAGGGAATTCCTGGTGTAGCGGTGAAATGCGTAGATATTAGGAAGAACACCAATGGCGAAAGCACTCTACTGGGCCATAACTGACACTGAGAGACGACAGCTAGGGGAGCAAATGGG
## 264                                                          ACGTGGAGTGCGAGCGTTATCCGGATTTACTGGGCGTAAAGAGTTCGTAGGCGTTTGATAAAGTTTCGTTTGAAATACCGAGGCTCAACTTCGGGAACGGACGAAATACTTATCAGATTGAGATATCTAGGGGGTACTGGAACTGACAGTGTAGCAGTGAAATGCGTTGATATTGTCAAGAACACCAAGGGCGAAGGCAGGTACCTGGGGATATTCTGACGCTGAGGAACGAAAGCTAGGGGAGCGAAAGGG
## 265                                                          ACGAAGGGGGCTAGCGTTGTTCGGAATTACTGGGCGTAAAGGGCGCGTAGGCGGCGCTGTAAGTGAGGTGTGAAAGACCTGGGCTCAACCTGGGAAGTGCATTTCAGACTGCAGTGCTAGAGTGCGAGAGAGGAAAGTGGAATGACGCGTGTAGAGGTGAAATTCGTAGATATGCGTTGGAACACCAGTGGCGAAGGCGACTTTCTGGCTCGTAACTGACGCTAAGGCGCGAAAGCGTGGGGAGCAAACAGG
## 266                                                          ACGTAGGATGCAAGCGTTATCCGGAATTATTGGGCGTAAAGCGTTCGTAGGTGGTTTGTTAAGTAGGGCGTTAAATCTTTGGGCTTAACCCAAAGCTGGCGTCCTAAACTGGCTCACTAGAGACAGGTAGAGGTAAGTGGAATTCTGTATGTAGGGGTAATATCCGTAGATATGCAGAGGAACACCAAAAGCGAAAGCAACTTACTGGACCTGTTCTGACACTCAAGAACGAAAGCGTGGGGAGCAAACGGG
## 267                                                          ACGTAGGGTGCAAGCATTAATCGGAATTATTGGGCGTAAAGGGCGCGTAGGCGGATTGATAAGTCAGATGTGAAATACCAAAGCTCAACTTTGGTGCTGCATTTGAAACTGTCTCTCTAGAGGATTGACGGAGAAAGGGGAATTCCACGTGTAGCGGTGAAATGCGTAGATACGTGGAGGAACACCGGTGGCGAAAGCGCCTTTCTAGTTTAGACCTGACGCTGAGGCGCGAGAGCAAGGGGAGCAAACAGG
## 268                                                          ACGGAGGATCCAAGCGTTATCCGGATTTATTGGGTTTAAAGGGAGCGTAGATGGATGTTTAAGTCAGTTGTGAAAGTTCGGGGCTCAACCCCGGAATTGCAGTTGATACTGGATATCTTGAGTACAGTTGAGGTAGGCGGAATTCGTGGTGTAGCGGTGAAATGCTTAGATATCACGAAGAACTCCGATTGCGAAGGCAGCTTACTAAGCTGTAACTGACATTGAGGCTCGAAAGTGTGGGTATCAAACAGG
## 269                                                          ACGAGTGCCTCAAGCGTTACCCGGAATCACTGGGCGTAAAGGTTGTGTAGGTGGTTGTATTAGTCTTTTGTTAAAGCCTGCGGCTTAACCGCAGAATCGCAAAGGAAACGGTACAACTAGAGGATGCAAGGGGTGTGTGGAACTCATAGTGTAGCGGTGAAATGCGTTGATATTATGGGGAACACCAAAAGCGAAGGCAGCACACTGGTGCACTCCTGACACTGAAACAAGAAAGCGTAGGTAGCGAATGGG
## 270                                                          ACAGAGGGTGCGAGCGTTAATCGGATTTACTGGGCGTAAAGCGTGCGTAGGCGGCTTTTTAAGTCGGATGTGAAATCCCTGAGCTTAACTTAGGAATTGCATTCGATACTGGGAAGCTAGAGTATGGGAGAGGATGGTAGAATTCCAGGTGTAGCGGTGAAATGCGTAGAGATCTGGAGGAATACCGATGGCGAAGGCAGCCATCTGGCCTAATACTGACGCTGAGGTACGAAAGCATGGGGAGCAAACAGG
## 271                                                          ACGGAGGGTGCAAGCGTTATCCGGATTTATTGGGTTTAAAGGGTCCGTAGGCGGATGTGTAAGTCAGTGGTGAAATCTCACAGCTTAACTGTGAAACTGCCATTGATACTGCATGTCTTGAGTGTTGTTGAAGTAGCTGGAATAAGTAGTGTAGCGGTGAAATGCATAGATATTACTTAGAACACCAATTGCGAAGGCAGGTTACTAAGCAACAACTGACGCTGATGGACGAAAGCGTGGGGAGCGAACAGG
## 272                                                         ACGAGGGCCCCGAGCGTTATCCGGAATTATTGGGCGTAAAGGGTGTGTAGGTGGTCATATTAGTCTTGTGTAAAAGCCTGTCGCCTAACGGCAGATCCGCATGGGAAACGGTATGACTGAGAGGGTGTAAGAGGTATATGGAACTCACGGTGTAGGGGTGAAATCCGTTGATATCGTGGGGAACGCCAAAAGCGAAAGCAATATACTGGTACATACCTGACACTGAAACACGAAAGCTAGGGTAGCGAATGGG
## 273                                                          ACGTAGGGTGCGAGCGTTAATCGGAATTACTGGGCGTAAAGCGTGCGCAGGCGGTTTTGTAAGACAGGCGTGAAATCCCCGGGCTCAACCTGGGAACTGCGTTTGTGACTGCAAGGCTAGAGTATGGCAGAGGGGGGTGGAATTCCACGTGTAGCAGTGAAATGCGTAGAGATGTGGAGGAACACCGATGGCGAAGGCAGCCCCCTGGGCCAATACTGACGCTCATGCACGAAAGCGTGGGGAGCAAACAGG
## 274                                                          ACGAAGGGGGCTAGCGTTGCTCGGAATCACTGGGCGTAAAGCGCACGTAGGCGGATCCTTAAGTCAGAGGTGAAATCCCAAGGCTCAACCTTGGAACGGCCTTTGATACTGGGGATCTCGAGTCCGGAAGAGGTTGGTGGAACTGCGAGTGTAGAGGTGAAATTCGTAGATATTCGCAAGAACACCAGTGGCGAAGGCGGCCAACTGGTCCGGCACTGACGCTGAGGTGCGAAAGCGTGGGGAGCAAACAGG
## 275                                                          ACGGAGGGTGCAAGCGTTGTTCGGAATTACTGGGCGTAAAGCGTGTCTAGGTGGATTTGTAAGTCAGGCGTGAAATCCCCCGGCTCAACCGGGGAGGGTCGCTTGATACTGCATATCTTGAGTTTCGGAGAGGTGGGCAGAATTCCCAGTGTAGAGGTGAAATTCGTAGAGATTGGGAGGAATACCGGAGACGAAGGTGGCTCACTAGCCGAATACTGACACTCAAGCACGAAAGCGTGGGTAGCAAACAGG
## 276                                                          ACAGAGGGTGCGAGCGTTAATCGGAATTACTGGGCGTAAAGCGCATGCAGATGGCTTTGTAAGTCGGATGTGAAATCCCCGGGCTTAACCTGGGAACTGCACTCGAAACTGCAAGGCTAGAATGTGGAAGAGGGAAGTGGAATTCCGGGTGTAGCAGTGAAATGCGTAGATATCCGGAGGAACATCAGTGGCGAAGGCGACTTCCTGGTCCAACATTGACATTCAGATGCGAAAGCGTGGGGAGCAAACAGG
## 277                                                          ACGTAGGGTGCAAGCGTTAATCGGAATTACTGGGCGTAAAGCGTGCGCAGGCGGTTATATAAGACAAATGTGAAATCCCCGGGCTCAACCTGGGAACTGCATATGTGACTGTATAGCTAGAGTGCGGCAGAGGGGGATGGAATTCCGCGTGTAGCAGTGAAATGCGTAGATATGCGGAGGAACACCGATGGCGAAGGCAATCCCCTGGGCCTGCACTGACGCTCATGCACGAAAGCGTGGGGAGCAAACAGG
## 278                                                          ACGTAGGGTGCAAGCGTTAATCGGAATTACTGGGCGTAAAGCGTGCGCAGGCGGTTATGCAAGACAGATGTGAAATCCCCGGGCTTAACCTGGGAACTGCATTTGTGACTGCATGGCTAGAGTGCGGCAGAGGGGGATGGAATTCCGCGTGTAGCAGTGAAATGCGTAGATATGCGGAGGAACACCGATGGCGAAGGCAATCCCCTGGGCCTGCACTGACGCTCATGCACGAAAGCGTGGGGAGCAAACAGG
## 279                                                         ACGAAGGGAGCAAGCGTTATCCTAAATGATTGGGCGTAAAGGGTTCGTAGGTTGTAATTGTTGTCGTATGTGAAATCTTGAAATTTTCTTTCAAATATGCATATGATACTCTATTACTTGAGTATATCAGGGGATAACAGAATGTTATATGGAGGAGTAAAATCCATTGAGCTATAATGGAATGCCAGTGGCGAAAGCGGTTATCTGGGATATATACTGACGCTGAGGAACGAAAGCGTGGGGAGCAAACAGG
## 280                                                          ACGTAGGGTGCGAGCGTTAATCGGAATTACTGGGCGTAAAGCGTGCGCAGGCGGTTTTGTAAGTCAGATGTGAAATCCCCGAGCTTAACTTGGGAACTGCGTTTGAAACTACAAGACTAGAGTGTGTCAGAGGGGGGTAGAATTCCACGTGTAGCAGTGAAATGCGTAGAGATGTGGAGGAATATCAATGGCGAAGGCAGCCCCCTGGGATAACACTGACGCTCATGCACGAAAGCGTGGGGAGCGAACAGG
## 281                                                          ACGTAGGGTGCGAGCGTTAATCGGAATTACTGGGCGTAAAGCGTGCGCAGGCGGCTTTGCAAGACAGATGTGAAATCCCCGGGCTCAACCTGGGAACTGCATTTGTGACTGCAAGGCTAGAGTACGGCAGAGGGGGATGGAATTCCGCGTGTAGCAGTGAAATGCGTAGATATGCGGAGGAACACCAATGGCGAAGGCAATCCCCTGGGCCTGTACTGACGCTCATGCACGAAAGCGTGGGGAGCAAACAGG
## 282                                                          ACGAGTGCTTCGAGCGTTATCCGGAATCATTGGGCGTAAAGGGTGTGTAGGTTGTTGTGTTAGTCTCTCGTCAAATCTTTCGGCTCAACCGAGAGTTCGCGAGGGAAACGGCACGACTAGAGGTTGGGAGAGGTTTCTGGAACTCATGGTGTAGCGGTGAAATGCGTTGATATCATGGGGAACACCAAAAGCGAAGGCAAGAAACTGGCCCATACCTGACACTGAAACACGAAAGCGTGGGTCGCGAATGGG
## 283                                                          ACGAAGGGTGCAAGCGTTGTTCGGATTTATTGGGCGTAAAGCGCGCGTAGGCGGACCTGTAAGTCAGATGTGAAATCTCGGTGCTCAACACCGAAACTGCGTCTGAAACTGCGGGTCTAGAATCTTGGAGGGGGAGTGGGAATTTCGCATGTAGGGGTAAAATCCGTAGAGATGCGAAGGAACACCAGAGGCGAAGGCGCACTCCTGGACAAGTATTGACGCTGAGGCGCGAAAGCGTGGGGATCAAACAGG
## 284                                                          ACGAAGGGGGCTAGCGTTGCTCGGAATCACTGGGCGTAAAGCGCACGTAGGCGGGCTTTTAAGTCGGAGGTGAAATCCCAGGGCTCAACCCTGGAACTGCCTTCGATACTGAAAGTCTTGAGTCCGGAAGAGGTAAGTGGAACTCCGAGTGTAGAGGTGAAATTCGTAGATATTCGGAAGAACACCAGTGGCGAAGGCGGCTTACTGGTCCGGTACTGACGCTGAGGTGCGAAAGCGTGGGGAGCAAACAGG
## 285                                                          ACGTAGGGAGCAAGCGTTATCCGGAATTACTGGGTGTAAAGGGAGTGTAGGCGGGATTACAAGTCAGATGTGAAAACTGTGGGCTCAACTCACAGATTGCATTTGAAACTGTAGTTCTTGAGTGAAGTAGAGGTAAGTGGAATTCCTAGTGTAGCGGTGAAATGCGTAGATATTAGGAGGAACATCAGTGGCGAAGGCGACTTACTGGGCTTTAACTGACGCTGAGGCTCGAAAGCGTGGGGAGCAAACAGG
## 286                                                          ACGGAGGATCCAAGCGTTATCCGGAATCATTGGGTTTAAAGGGTCCGTAGGCGGTTTAATAAGTCAGTGGTGAAAGCCCATCGCTCAACGGTGGAACGGCCATTGATACTGTTAAACTTGAATTATTAGGAAGTAACTAGAATATGTAGTGTAGCGGTGAAATGCTTAGAGATTACATGGAATACCAATTGCGAAGGCAGGTTACTACTAATGGATTGACGCTGATGGACGAAAGCGTGGGTAGCGAACAGG
## 287                                                         ACGAAGGGAGCAAGCGTTATCCTAAATGATTGGGCGTAAAGGGTTCGTAGGTTGTAATTGTTGTCGTATGTGAAATCTTGAAACTTTATTTCAAATATGCATATGATACTCTATTACTTGAGTATATCAGGGGATAACAGAATGTTATATGGAGGAGTAAAATCCATTGAGCTATAATGGAATGCCAGTGGCGAAAGCGGTTATCTGGGATATATACTGACGCTGAGGAACGAAAGCGTGGGGAGCAAACAGG
## 288                                                      ACAGAGACCTCAAGCGTTATCCGGAATCATTGGGCGTAAAGCGTACCGATAGGTGGCTTTACAAGTCAGAAGTGAAATCCTAAGGCTTAACTTTGGGACTGTCTTTTGAAACTGTAAAGCTAGAGGGGCAAAGAGGAAGCTGGAACGAACGGTGTAGTAGTGAAATGCGTTGATATCGTTCGGAACACCAATAGCGTAGGCAGGCTTCTGGGTGCCACCTGACACTGCTAGGACGAAAGCGTGGGTAGCGATAAGG
## 289                                                          ACGGAGGGTGCGAGCGTTGTCCGGAATCACTGGGCGTAAAGGGCGCGTAGGCGGTCTGCTAAGCGTGCGGTGAAAGCCCGGGGCTCAACCCCGGGTCGGCCGTGCGAACTGGTGGACTAGAGCACTGTAGAGGCAGGTGGAATTCCGGGTGTAGCGGTGGAATGCGTAGAGATCCGGAAGAACACCGGTGGCGAAGGCGGCCTGCTGGGCAGTAGCTGACGCTGAGGCGCGACAGCGTGGGGAGCAAACAGG
## 290                                                          ACGTAGGGTGCGAGCGTTAATCGGAATTACTGGGCGTAAAGCGTGCGCAGGCGGTTTCGTAAGACAGAGGTGAAATCCCCGGGCTCAACCTGGGAACTGCCTTTGTGACTGCATAGCTGGAGTGCGGCAGAGGGGGATGGAATTCCGCGTGTAGCAGTGAAATGCGTAGATATGCGGAGGAACACCGATGGCGAAGGCAATCCCCTGGGCCTGCACTGACGCTCATGCACGAAAGCGTGGGGAGCAAACAGG
## 291                                                          ACGAAGGATGCAAGCGTTATCCGGATTCATTGGGTTTAAAGGGTGCGTAGGCGGATTAGTAAGTCAGTGGTGAAATCCCATCGCTTAACGATGGTACTGCCATTGATACTGCTAGTCTTGAGTACATATGACGTTGGCGGAATATGACATGTAGCGGTGAAATGCTTAGATATGTCATAGAACACCGATTGCGAAGGCAGCTAACGAAAATGTAACTGACGCTGATGCACGAAAGTGCGGGGATCAAACAGG
## 293                                                          ACGGAGGGTGCAAGCGTTATCCGGAATCATTGGGTTTAAAGGGTCCGCAGGCGGATTTATAAGTCAGTGGTGAAAGCCTACAGCTCAACTGTAGAACTGCCATTGATACTGTAAGTCTTGAATTCGGTCGAAGTGGGCGGAATATGACATGTAGCGGTGAAATGCTTAGATATGTCATAGAACACCGATAGCGAAGGCAGCTCACTAGGCCTGGATTGACGCTCAGGGACGAAAGCGTGGGGAGCAAACAGG
## 294                                                          ACGGAGGGTGCGAGCGTTGTCCGGAATCATTGGGTGTAAAGGGTGCGTAGGCGGGTTCTTAAGTCTGGGGTGAAAGTCTGCGGCTCAACCGTAGGATTGCCTTGGATACTGGGAGTCTTGAGTATGACAGAGGCCGGTAGAATTCGTGGTGTAGCGGTGAAATGCATAGATATCACGAAGAATACCAGTGGCGTAGGCGGCCGGCTGGGTCAACACTGACGCTGAGGCACGAGAGTGTGGGGAGCAAACAGG
## 295                                                          ACATAGGGTGCAAGCGTTGTCCGGAATTATTGGGCGTAAAGAGCTCGTAGGTGGTTTGATACGTCGGATGTGAAATTCAGGGGCTCAACCCCTGACCTGCATTCGATACGGTCAAGCTAGAGTTTGGTAGGGGAGACTGGAATTCCTGGTGTAGCGGTGGAATGCGCAGATATCAGGAGGAACACCGATGGCGAAGGCAGGTCTCTGGGCCAATACTGACACTGAGGAGCGAAAGCGTGGGGAGCGAACAGG
## 296                                                          ACGTAGGGTGCGAGCGTTAATCGGAATTACTGGGCGTAAAGCGTGCGCAGGCGGCTTTGCAAGACAGATGTGAAATCCCCGGGCTCAACCTGGGAACTGCATTTGTGACTGCAAGGCTAGAGTACGGCAGAGGGGGATGGAATTCCGCGTGTAGCAGTGAAATGCGTAGATATGCGGAGGAACACCGATGGCGAAGGCAATCCCCTGGGCCTGTACTGACGCTCATGCACGAAAGCGTGGGGAGCAAACAGG
## 297                                                          ACAGAGGGTGCAAGCGTTAATCGGAATTACTGGGCGTAAAGCGCGCGTAGGCGGTTTGTTAAGTCGGATGTGAAATCCCTGGGCTCAACCTAGGCACTGCATCCGATACTGGCTAACTAGAGTGTGGGAGAGGAAGGTAGAATTCCAGGTGTAGCGGTGAAATGCGTAGAGATCTGGAGGAATACCGATGGCGAAGGCAGCCTTCTGGCCTAACACTGACGCTGAGGTGCGAAAGCATGGGGAGCAAACAGG
## 298                                                          ACGTAGGGTGCGAGCGTTAATCGGAATTACTGGGCGTAAAGCGTGCGCAGGCGGTCTTGCAAGACAGGTGTGAAATCCCCGGGCTTAACCTGGGAACTGCACTTGTGACTGCAAGGCTGGAGTACGGCAGAGGGGGATGGAATTCCGCGTGTAGCAGTGAAATGCGTAGATATGCGGAGGAACACCGATGGCGAAGGCAATCCCCTGGGCCTGTACTGACGCTCATGCACGAAAGCGTGGGGAGCAAACAGG
## 299                                                          ACGTAGGTGACAAGCGTTATCCGGATTTACTGGGCGTAAAGGGCGTGTAGGCGGCTTAGCAAGTCAGATGTGAAATTCTTGGGCTCAACCCGAGAGCTGCATCTGAAACTGCTGAGCTTGAGTGCTGGATGGGATAGCGGAATTCCTGGTGGAGCGGTAAAATGCGCAGATATCAGGAAGAACACCGGTGGCGAAGGCGGCTATCTGGACAGTAACTGACGCTGAGGCGCGAAAGCGTGGGGAGCAAACAGG
## 300                                                          ACGTAGGGTGCGAGCGTTAATCGGAATTACTGGGCGTAAAGCGTGCGCAGGCGGTTATATAAGACAGATGTGAAATCCCCGGGCTTAACCTGGGAACTGCGTTTGTGACTGTATAACTCGAGTGTGGCAGAGGGGGGTGGAATTCCACGTGTAGCAGTGAAATGCGTAGAGATGTGGAGGAACACCGATGGCGAAGGCAGCCCCCTGGGTCAACACTGACGCTCATGCACGAAAGCGTGGGGAGCAAACAGG
## 301                                                          ACGTAGGGTGCAAGCGTTAATCGGAATTACTGGGCGTAAAGCGTGCGCAGGCGGTTTTATAAGACAGAGGTGAAATCCCCGGGCTCAACCTGGGAACTGCCTTTGTGACTGTAAGGCTAGAGTGTGTCAGAGGGGGGTAGAATTCCACGTGTAGCAGTGAAATGCGTAGATATGTGGAGGAATACCGATGGCGAAGGCAGCCCCCTGGGATAACACTGACGCTCATGCACGAAAGCGTGGGGAGCAAACAGG
## 302                                                          ACAGAGGTGGCAAGCGTTGTTCGGATTTATTGGGTGTAAAGGGCAGGTAGGCGGTCAAGTAAGTCTGTTGTGAAATCCCGGGGCTCAACCCCGGAACTGCAACGGAAACTACTTGGCTGGAGCAATGTAGGGGTGAGGGGAATTCTCGGTGTAAGGGTGAAATCTGTAGATATCGAGAGGAACACCAATGGCGAAGGCACCTCACTGGACATTTGCTGACGCTGAGCTGCGAAAGTAGGGGGAGCAAACAGG
## 303                                                          ACGTAGGGTGCAAGCGTTAATCGGAATTACTGGGCGTAAAGCGTGCGCAGGCGGCTTTGCAAGACAGATGTGAAATCCCCGGGCTCAACCTGGGAACTGCATTTGTGACTGCATGGCTAGAGTGCGGCAGAGGGGGATGGAATTCCGCGTGTAGCAGTGAAATGCGTAGATATGCGGAGGAACACCGATGGCGAAGGCAATCCCCTGGGCCTGCACTGACGCTCATGCACGAAAGCGTGGGGAGCAAACAGG
## 304                                                          ACAGAGACCTCAAGCGTTATCCGGATTCATTGGGCGTAAAGCGTCCGCAGGTGGTTTTTCAAGTTGGGAGTCAAATCTTTGGGCTTAACCTAGAGACTGCTTCCAATACTGGGAAACTTGAGACTGGGAGAGGTATACGGAACTGTTGGTGTAGTAGTAAAATGCGTTGATATCAACAGGAACACCAAAGGCGAAAGCAGTATACTGGAACAGTTCTGACACTCATGGACGAAAGCGTGGGGAGCGAATGGG
## 305                                                          ACGTAGGGTGCGAGCGTTAATCGGAATTACTGGGCGTAAAGCGTGCGCAGGCGGCTTTGCAAGACAGAGGTGAAATCCCCGGGCTCAACCTGGGAACTGCCTTTGTGACTGCAAGGCTAGAGTACGGCAGAGGGGGATGGAATTCCGCGTGTAGCAGTGAAATGCGTAGATATGCGGAGGAACACCAATGGCGAAGGCAATCCCCTGGGCCTGTACTGACGCTCATGCACGAAAGCGTGGGGAGCAAACAGG
## 306                                                          ACGTAGGGTGCAAGCGTTGTCCGGAATTATTGGGCGTAAAGAGCTCGTAGGCGGTTTGTCGCGTCTGCTGTGAAAATCCGAGGCTCAACCTCGGACCTGCAGTGGGTACGGGCAAGCTAGAGTGCGGTAGGGGAGATGGGAATTCCTGGTGTAGCGGTGGAATGCGCAGATATCAGGAGGAACACCAATGGCGAAGGCACATCTCTGGGCCGTAACTGACGCTGAGGAGCGAAAGCGTGGGGAGCGAACAGG
## 307                                                          ACGTAGGGTGCAAGCGTTAATCGGAATTACTGGGCGTAAAGCGTGCGCAGGCGGTTATATAAGTCAGATGTGAAATCCCCGGGCTCAACCTGGGAACTGCATTTGAGACTGTATAGCTAGAGTACGGTAGAGGGGGATGGAATTCCGCGTGTAGCAGTGAAATGCGTAGATATGCGGAGGAACACCGATGGCGAAGGCAATCCCCTGGGCCTGTACTGACGCTCATGCACGAAAGCGTGGGGAGCAAACAGG
## 308                                                          ACGAAGGGGGCTAGCGTTGTTCGGAATTACTGGGCGTAAAGCGCACGTAGGCGGGTCGTTAAGTCAGGGGTGAAATCCCGGAGCTCAACTCCGGAACTGCCTTTGATACTGGCGATCTAGAGATCGGAAGAGGTGAGTGGAATTCCCAGTGTAGAGGTGAAATTCGTAGATATTGGGAAGAACACCAGTGGCGAAGGCGGCTCACTGGTCCGATACTGACGCTGAGGTGCGAAAGCGTGGGGAGCAAACAGG
## 309                                                          ACGAAGGGTGCAAGCGTTAATCGGAATTACTGGGCGTAAAGGGTGCGTAGGTGGTTTATTAAGTCGGATGTGAAATCCCTGGGCTTAACCTAGGAACTGCATACGATACTGGTAGACTAGAGTGCGGTAGAGGATGGTGGAATTCCCGGTGTAGCGGTGAAATGCGTAGAGATCGGGAGGAACATCAGTGGCGAAGGCGGCCATCTGGACCAGCACTGACACTAAAGCACGAAAGCGTGGGGAGCAAACAGG
## 310                                                          ACGAGGGATCCAAGCGTTGTTCGGAATCATTGGGCGTAAAGCGGGTGTAGGTGGCTATATAAGTCAGATGTGAAAGCCCAGGGCTTAACCCTGGAAGTGCATTTGATACTGTTTAGCTTGAGTGTGGGAGAGGTTACTGGAATTCCTGGTGTAGTGGTGAAATACGTAGATATCAGGAGGAATACCGGTGGCGAAGGCGGGTAACTGGCCCAACACTGACACTGAGACCCGAAAGCGTGGGGATCAAACAGG
## 311                                                          ACGGAGGGTGCAAGCGTTATTCGGAATTACTGGGCGTAAAGCGCACGTAGGCCGCTTTGTAAGTCAGGGGTGAAATCCCACGGCTCAACCGTGGAACTGCCCTTGAAACTGCAGAGCTCGAATCCTGGAGAGGGTGGCGGAATTCCTGGTGTAGGAGTGAAATCCGTAGATATCAGGAGGAACACCGGTGGCGAAGGCGGCCACCTGGACAGGTATTGACGCTGAGGTGCGAAAGTGTGGGGAGCAAACAGG
## 312                                                          ACGAAGGTGGCAAGCGTTGTTCGGATTTACTGGGCGTAAAGAGTGCGTAGGCGGTTTAATAAGTCAGGAGTGAAAGCCCAAGGCTCAACCTTGGAATTGCTTTTGAAACTATTTTACTTGAATCCGGTAGAGGTTGGCGGAATTCCCAGTGTAGAGGTGAAATTCGCAGATATTGGGAGGAACACCAGTGGCGTAAGCGGCCAACTGGACCGGTATTGACGCTGAGGCACGAAAGCGTGGGTAGCAAACAGG
## 313                                                          ACGAAGGGTGCTAGCGTTGTTCGGAATGACTGGGCGTAAAGGGTGCGTAGGCGGCCAGAAGCGTCTGATGTGAAATCCCTGGGCTTAACCTAGGAACTGCATTGGATACGGTCTGGCTAGAGTCCGCGAGAGGAAGATGGAATTGTGCGTGTAGAGGTGAAATTCGTAGATATGCACAAGAACACCGGTGGCGAAGGCGATCTTCTGGAGCGGTACTGACGCTAAGGCACGAAAGTGTGGGGAGCAAACAGG
## 314                                                          ACGAAGGGTGCTAGCGTTGTTCGGAATGACTGGGCGTAAAGGGCGCGTAGGCGGTCTTTTAAGTTAGGCGTGAAAGCCCTGGGCTTAACCCAGGACGTGCGCTTAAGACTGGAAGGCTTGAGTTTGGAAGAGGAAAGTGGAATTCCTAGTGTAGAGGTGAAATTCGTAGATATTAGGAAGAACACCAGTGGCGAAGGCGACTTTCTGGTCCAATACTGACGCTGAGGCGCGAAAGTGTGGGGAGCAAACAGG
## 315                                                          ACGTAGGGTGCGAGCGTTAATCGGAATTACTGGGCGTAAAGCGTGCGCAGGCGGTTTTGAAAGTCAGATGTGAAATCCCCGGGCTCAACCTGGGAACTGCGTTTGAAACTCCAAAGCTAGAGTATGGGAGAGGGAGGTAGAATTCCACGTGTAGCAGTGAAATGCGTAGAGATGTGGAGGAATACCAATGGCGAAGGCAGCCTCCTGGCTTAATACTGACGCTCATGCACGAAAGCGTGGGGAGCAAACAGG
## 316                                                          ACATAGGGTGCAAGCGTTGTCCGGAATTATTGGGCGTAAAGAGCTCGTAGGTCGTTTGACACGTCGGATGTGAAAATCTGAGGCTTAACCTCAGACCTGCATTCGATACGGTCAAACTAGAGTGTGGTAGGGGAGACTGGAATTCCTGGTGTAGCGGTGGAATGCGCAGATATCAGGAGGAACACCAATGGCGAAGGCAGGTCTCTGGGCCATTACTGACACTGAGGAGCGAAAGCGCGGGGAGCGAACAGG
## 317                                                          ACAGAGGGTGCAAGCGTTAATCGGAATTACTGGGCGTAAAGCGTGCGTAGGCGGTTGTTTAAGTCGGATGTGAAATCCCCGGGCTCAACCTGGGAATTGCATTCGATACTGGACAGCTAGAGTTCGGCAGAGGGAAGTGGAATTTCCGGTGTAGCGGTGAAATGCGTAGATATCGGAAGGAACATCAGTGGCGAAAGCGACTTCCTGGACCAGAACTGACGCTCAGGCACGAAAGCGTGGGGAGCAAACAGG
## 318                                                          ACGTAGGATGCAAGCGTTATCCGGAATCATTGGGCGTAAAGCGTCCGCAGGTGGAACAGTAAGTTGAGCGATAAATACCAGTGCCTAACATTGGTGCCTCGTTCAAAACTGCTGATCTAGAGGATGGGAGGGGCAGACGGAATTCCCGGTGTAGGGGTAAAATCCGTAGATATCGGGAAGAACACCAAAAGCGAAGGCAGTCTGCTGGAACATTCCTGACACTCATGGACGAAAGCGTGGGGAGCGAAAAGG
## 319                                                          ACGTAGGGTGCGAGCGTTAATCGGAATTACTGGGCGTAAAGCGTGCGCAGGCGGCCTTAAAAGTCAGATGTGAAATCCCCGAGCTCAACTTGGGAACTGCGTTTGAAACTCTAAGGCTAGAATATGTCAGAGGGGGGTAGAATTCCACGTGTAGCAGTGAAATGCGTAGAGATGTGGAGGAATACCAATGGCGAAGGCAGCCCCCTGGGATAATATTGACGCTCATGCACGAAAGCGTGGGGAGCAAACAGG
## 320                                                          ACGGAGGATCCAAGCGTTATCCGGAATCATTGGGTTTAAAGGGTCCGTAGGCGGTTTGGTAAGTCAGTGGTGAAAGCCCGCAGCTCAACTGTGGAACGGCCATTGATACTGCCAGACTTGAATTACAAGGAAGTAACTAGAATATGTAGTGTAGCGGTGAAATGCTTAGATATTACATGGAATACCAATTGCGAAGGCAGGTTACTACTTGTGGATTGACGCTGATGGACGAAAGCGTGGGGAGCGAACAGG
## 321                                                          ACGTAGGGTGCAAGCGTTAATCGGAATTACTGGGCGTAAAGCGTGCGCAGGCGGTCATGCAAGACAGATGTGAAATCCCCGGGCTCAACCTGGGAACTGCATTTGTGACTGCATGGCTTGAGTACGGCAGAGGGGGATGGAATTCCGCGTGTAGCAGTGAAATGCGTAGATATGCGGAGGAACACCGATGGCGAAGGCAATCCCCTGGGCCTGTACTGACGCTCATGCACGAAAGCGTGGGGAGCAAACAGG
## 322                                                          ACGTAGGGTGCGAGCGTTAATCGGAATTACTGGGCGTAAAGCGTGCGCAGGCGGTTTTGCAAGACAGTGGTGAAATCCCCGGGCTCAACCTGGGAACGGCCATTGTGACTGCAAGGCTGGAGTGCGGCAGAGGGGGATGGAATTTCGCGTGTAGCAGTGAAATGCGTAGATATGCGAAGGAACACCGATGGCGAAGGCAATCCCCTGGGCCTGCACTGACGCTCATGCACGAAAGCGTGGGGAGCAAACAGG
## 323                                                          ACGTAGGGTGCAAGCGTTAATCGGAATTACTGGGCGTAAAGCGTGCGCAGGCGGTTATATAAGTCAGATGTGAAATCCCCGGGCTCAACCTGGGAACTGCATTTGAGACTGTATAGCTAGAGTACGGTAGAGGGGGATGGAATTCCGCGTGTAGCAGTGAAATGCGTAGATATGCGGAGGAACACCGATGGCGAAGGCAATCCCCTGGGCCTGCACTGACGCTCATGCACGAAAGCGTGGGGAGCAAACAGG
## 324                                                          ACGTAGGGTGCAAGCGTTAATCGGAATTACTGGGCGTAAAGCGTGCGCAGGCGGTTTTGTAAGACAGAGGTGAAATCCCCGGGCTCAACCTGGGAACTGCCTTTGTGACTGCAAAGCTGGAGTACGGCAGAGGGGGATGGAATTCCGCGTGTAGCAGTGAAATGCGTAGATATGCGGAGGAACACCGATGGCGAAGGCAATCCCCTGGGCCTGTACTGACGCTCATGCACGAAAGCGTGGGGAGCAAACAGG
## 325                                                          ACGTAGGGTGCAAGCGTTAATCGGAATTACTGGGCGTAAAGCGTGCGCAGGCGGTTTTGTAAGACAGAGGTGAAATCCCCGGGCTCAACCTGGGAACTGCCTTTGTGACTGCAAAGCTGGAGTGCGGCAGAGGGGGATGGAATTCCGCGTGTAGCAGTGAAATGCGTAGATATGCGGAGGAACACCGATGGCGAAGGCAATCCCCTGGGCCTGCACTGACGCTCATGCACGAAAGCGTGGGGAGCAAACAGG
## 326                                                          ACGAAGGGTGCAAGCGTTGTTCGGAATCATTGGGCGTAAAGCGCGCGCAGGCGGATCAGCAAGTCAGATGTGAAATCTCGAAGCTCAACTTCGAAACTGCGTCTGAAACTGCTAGTCTAGAATGTCGGAGGGGGCAGGGGAATTTCACGTGTAGGGGTAAAATCCGTAGAGATGTGAAGGAACACCGGAGGCGAAGGCGCCTGCCTGGACGACTATTGACGCTGAGGCGCGAAAGCGTGGGGAGCAAACAGG
## 327                                                          ACGGAGGATCCAAGCGTTATCCGGAATCATTGGGTTTAAAGGGTCCGTAGGCGGTCTTATAAGTCAGTGGTGAAAGCCCATCGCTCAACGATGGAACTGCCATTGATACTGTAAGACTTGAATGCTTAGGAAGTAACTAGAATATGTAGTGTAGCGGTGAAATGCTTAGATATTACATGGAATACCAATTGCGAAGGCAGGTTACTACTAAGTGATTGACGCTGATGGACGAAAGCGTGGGGAGCGAACAGG
## 328                                                          ACGAAGGGTGTAAGCGTTGTTCGGATTTATTGGGCGTAAAGCGCGCGCAGGTGGATTTTTAAGTCAGATGTGAAATCTCGGGGCTCAACCCCGAACGTGCGTCTGAAACTGGAGATCTAGAATATTGGAGGGGGTAGAGGAATTTCACATGTAGGGGTAAAATCCGTAGAGATGTGAAGGAACACCAGAGGCGAAGGCGTCTACCTGGCCAACCATTGACACTGAGGCGCGAAAGCGTGGGGAGCAAACAGG
## 329                                                          ACGAAGGGTGCGAGCGTTGTTCGGAATGACTGGGCGTAAAGGGTTCGTAGGCGGGAGTGCAAGTCAAGTGTGAAATCCCCAGGCTTAACCTGGGACGTGCACTTGAAACTGCATTTCTTGAGTCGAGGAGAGGGTAATGGAATTGCTGGTGTAGGAGTGACATCCGTAGAGATCAGCAGGAACACCGGAGGCGAAGGCGATTACCTGGCCGAAGACTGACGCTGAGGAACGAAAGCGTGGGGAGCAAACAGG
## 330                                                          ACGGAGGATCCAAGCGTTATCCGGATTTATTGGGTTTAAAGGGTGCGTAGGCGGCTTATTAAGTCAGGGGTGAAAGACGGTGGCTCAACCATCGCAGTGCCCTTGATACTGATGAGCTTGAATACACTAGAGGTAGGCGGAATGAGACAAGTAGCGGTGAAATGCATAGATATGTCTCAGAACACCAATTGCGAAGGCAGCTTACTATGGTGTTATTGACGCTGAGGCACGAAAGCGTGGGGATCAAACAGG
## 331                                                          ACGGAGGGTGCAAGCGTTACTCGGAATCACTGGGCGTAAAGAGCGTGTAGGCGGGTGAATAAGTCAGAAGTGAAATCCAATAGCTCAACTATTGAACTGCTTTTGAAACTGTATACCTAGAATGTGGGAGAGGCAGATGGAATTTCTGGTGTAGGGGTAAAATCCGTAGAGATCAGAAGGAATACCGATTGCGAAGGCGATCTGCTGGAACATTATTGACGCTGAGACGCGAAAGCGTGGGGAGCAAACAGG
## 332                                                          ACGAAGGTGGCGAGCGTTACTCGGATTTACTAGGCGTAAAGCGTGGGCAGGCGGCTCGATTAGTCTCTTGTGAAAGCCTTGGGCTTAACCCAGGGAGGCCAAGAGATACTATCGGGCTTGGATGTGGGAGAGGAGACTGGAATTCCCGGTGTAGCGGTGAAATGCGTTGATATCGGGAGGAACACCAATGGCGAAAGCAAGTCTCTGGACCACCATCGACGCTCATCCACGAAAGCTGGGGGATCAAACAGG
## 333                                                          ACAGAGACCTCAAGCGTTATCCGGATTTATTGGGCGTAAAGCGCAGGTAGGCGGGTTTATTAGTCGGATGTCAAAGACCGGGGCTCAACCCCGGGAAGGCATTCGAAACGGTAAACCTAGAGAATGTGAGAGATGAGTGGAATTCATGGTGTAGTAGTGAAATGCGTTGATATCATGAGGAACACCGAAGGCGAAGGCAACTCATTGGCACATTTCTGACGCTGAGCTGCGAAAGCGTGGGGAGCGAATGGG
## 334                                                          ACGGAGGGTGCAAGCGTTATCCGGATTTATTGGGTTTAAAGGGTCCGTAGGCGGATCGTTAAGTCAGTGGTGAAAGCCTACCGCTCAACGGTAGAACTGCCATTGATACTGGCGATCTTGAATATGTATGAAGTTGGCGGAATGTGTGGTGTAGCGGTGAAATGCTTAGATATCACACAGAACACCGATTGCGAAGGCAGCTGACTAATACATGATTGACGCTGAGGGACGAAAGTGTGGGGATCAAACAGG
## 335                                                          ACGTAGGATGCAAGCGTTGTCCGGATTTATTGGGCGTAAAGAGTTCGTAGGTGGTTTGTTAAGTTTGGTGTTAAAGATTGGGGCTCAACCCTGAAACTGCACTGAATACTGGCAGACTCGAGTGTGGTAGAGGCTAGTGGAATTCCCAGTGTAGCGGTGAAATGCGTAGATATTGGGAAGAACACCGGTGGCGTAGGCGACTAGCTGGGCCATAACTGACACTGAGGAACGAAAGCCAGGGGAGCGAATGGG
## 336                                                          ACGTAGGATGCAAGCGTTGTCCGGATTTATTGGGCGTAAAGAGTTCGTAGGTGGTTTGTCAAGTCTGATGTTAAAGATCGGGGCTCAACCCTGGGACTGCATTGGATACTGGCAGACTTGAGTATGGTAGAGGCTAGTGGAATTCCCAGTGTAGCGGTGAAATGCGTAGATATTGGGAAGAACACCGGTGGCGTAGGCGACTAGCTGGGCCATAACTGACGCTGAGGAACGAAAGCCAGGGGAGCGAATGGG
## 337                                                          ACGGAGGATCCGAGCGTTATCCGGATTTATTGGGTTTAAAGGGTGCGCAGGCGGCTTTTTAAGTTGCCGGTGAAATGTCGGGGCTCAACTCCGTCACTGCCGGCGATACTGGAAGGCTTGAGAATGGAGGAGGATTCCGGAATGTGTGGTGTAGCGGTGAAATGCATAGATATCACACAGAACACCGATTGCGAAGGCAGGAGTCCACACCATATCTGACGCTGAGGCACGAAAGCGTGGGGATCGAACAGG
## 338                                                         ACGTAGGCACCAAGCGTTGTCCGGATTTATTGGGCGTAAAGAGCTCGTAGGCGGTTCAGCAAGTCGGGTGTGAAAACTCTGGGCTTAACCCAGAGCCTGCACCCGAAACTGCTGTGACTAGAGTTCGGTAGGGGAGCGGGGAATTCCTGGTGTAGCGGTGAAATGCGCAGATATCAGGAGGAACACCGGTGGCGAAGGCGCCGCTCTGGGCCGAAACTGACGCTGAGGAGCGAAAGCGTGGGTAGCAAACAGG
## 339                                                          ACGTAGGGTGCAAGCGTTAATCGGAATTACTGGGCGTAAAGCGTGCGCAGGCGGCTTTGCAAGACAGATGTGAAATCCCCGGGCTCAACCTGGGAACTGCATTTGAGACTGTATAGCTAGAGTACGGTAGAGGGGGATGGAATTCCGCGTGTAGCAGTGAAATGCGTAGATATGCGGAGGAACACCGATGGCGAAGGCAATCCCCTGGACCTGTACTGACGCTCATGCACGAAAGCGTGGGGAGCAAACAGG
## 340                                                          ACGTAGGGTGCGAGCGTTAATCGGAATTACTGGGCGTAAAGCGTGCGCAGGCGGTTTTGTAAGTCAGACGTGAAAGCCCCGGGCTTAACCTGGGAATTGCGTTTGAAACTGCAAGGCTAGAGTACGGCAGAGGGGGGTAGAATTCCACGTGTAGCAGTGAAATGCGTAGATATGTGGAGGAATACCGATGGCGAAGGCAGCCCCCTGGGCTAGTACTGACGCTCATGCACGAAAGCGTGGGGAGCAAACAGG
## 341                                                          ACGGGAGTGGCAAGCGTTATCCGGAATTATTGGGCGTAAAGCGTCCGCAGGCGGTCTTGTAAGTCTGTCGTTAAAGCGTGGAGCTTAACTCCATTTCAGCGATGGAAACTGCAAGACTAGAGTGTGGTAGGGGCAGAGGGAATTCCCGGTGTAGCGGTGAAATGCGTAGATATCGGGAAGAACACCAGTGGCGAAGGCGCTCTGCTGGGCCATAACTGACGCTCATGGACGAAAGCCAGGGGAGCGAAAGGG
## 342                                                          ACGAGGGATCCAAGCGTTGTTCGGAATCATTGGGCGTAAAGCGGGTGTAGGCGGCTCTATAAGTCAGATGTGAAAGCCCTGGGCTCAACCCGGGAAGTGCATTTGATACTGCAGAGCTTGAGTGTGGGAGAGGCTAGTGGAATTCCTGGTGTAGTGGTGAAATACGTAGATATCAGGAGGAACATCGGTGGCGAAGGCGGCTAGCTGGCCCAACACTGACGCTGAGATCCGAAAGCGTGGGGATCAAACAGG
## 343                                                          ACGTAGGTGACAAGCGTTATCCGGATTTACTGGGTGTAAAGGGCGTGTAGGCGGTTTTGCAAGTCAGATGTGAAATTCCCAGGCTTAACCTGGGCGCTGCATCTGAAACTGCAAGACTTGAGTACTGGAGAGGATAGTGGAATTCCTAGTGTAGCGGTAAAATGCGCAGATATTAGGAGGAACACCGGTGGCGAAGGCGACTATCTGGACAGTAACTGACGCTGAGGCGCGAAAGCGTGGGGAGCAAACAGG
## 344                                                          ACAGAGACTGCAAGCGTTATTCGGATTCACTGGGCGTAAAGGGTGCGCAGGCGGCCAAGTGTGTGAGGCGTGAAAGCCCGAGGCTCAACCTCGGAATTGCACCTCAAACTACATGGCTAGAGCATTGGAGAGGGTAGCAGAATTCATGGTGTAGCAGTGAAATGCGTAGATATCATGAGGAATACCAGAGGCGAAGGCGGCTACCTGGACAATTGCTGACGCTCAGGCACGAAAGCGTGGGGAGCAAAAGGG
## 345                                                          ACGAAGGGTGCTAGCGTTACTCGGAATTATTGGGCGTAAAGCGTGCGCAGGCGGTATTGTAAGTTAAAAGTGAAAGCCTCAGGCTCAACCTGAGAATGGCTTTTAATACTGCAGTACTAGAGTATCAGAAAAGATGGCGGAATTCCTAGTGTAGAGGTGAAATTCGTAGATATTAGGAGGAACACCAGTGGCGAAAGCGGCTATCTAGCTGAATACTGACGCTGATGCACGAAAGCGTGGGGAGCAAACAGG
## 346                                                          ACAGAGGGTGCAAGCGTTAATCGGAATTACTGGGCGTAAAGCGCGCGTAGGTGGTTTTGTAAGTTGGAGGTGAAATCCCCGGGCTCAACCTGGGAACTGCCTCCAAAACTGCATAGCTAGAGTACGGTAGAGGGTGGTGGAATTTCCTGTGTAGCGGTGAAATGCGTAGATATAGGAAGGAACACCAGTGGCGAAGGCGACCACCTGGACTGATACTGACACTGAGGTGCGAAAGCGTGGGGAGCAAACAGG
## 347                                                          ACGAGTGCCTCAAGCGTTATCCGGAATCATTGGGCGTAAAGGTTGTGTAGGTGGTTATATTAGTCTTTTGTTAAATTCTTCGGCTTAACCGGGGGCATGCAAAGGAAACGGTATGACTAGAGGATGCGAGGGGTCTGCGGAACTCATAGTGTAGCGGTAAAATGCGTTGATATTATGGGGAACACCAAAAGCGAAGGCAGCAGACTGGAGCACTCCTGACACTGAAACAAGAAAGCGTGGGTCGCGAATGGG
## 348                                                          ACGGAGGGAGCTAGCGTTGTTCGGAATTACTGGGCGTAAAGAGTACGTAGGCGGTTATTCAAGTCAGAGGTGAAAGCCTGGAGCTCAACTCCAGAACTGCCTTTGAAACTAGATAGCTAGAATCTTGGAGAGGTGAGTGGAATTCCGAGTGTAGAGGTGAAATTCGTAGATATTCGGAAGAACACCAGTGGCGAAGGCGACTCACTGGACAAGTATTGACGCTGAGGTACGAAAGCGTGGGGAGCAAACAGG
## 349                                                          ACGAAGGGTGCTAGCGTTGTTCGGAATCATTGGGCGTAAAGCGTACGTAGGCGGTTTAGTAAGTCGATTGTGAAATACCCGAGCTCAACTCAGGAATTGCAATCGAAACTGCTTTGCTAGAGTTTAGTGGGGGATAGTGGAATTCCTAGTGTAGGGGTGAAATCCGTAGAGATTAGGAGGAACATCAGTGGCGAAGGCGACTATCTACGCTAATACTGACGCTAAGGTACGAAAGCGTGGGGAGCAAACAGG
## 350                                                          ACGAGTGCCTCAAGCGTTATCCGGAATCATTGGGCGTAAAGGTTGTGTAGGTGGTCATATTAGTCTTTTGTTAAATTCTTCGGCTTAACCGGGGGCATGCAAGAGAAACGGTATGACTAGAGGATGCGAGGGGTCTGCGGAATTCATAGTGTAGCGGTAAAATGCGTTGATATTATGGAGAACACCAAAAGCGAAGGCAGCAGACTGGAGCACTCCTGACACTGAAACAAGAAAGCGTGGGTCGCGAATGGG
## 351                                                          ACGTAGGGTGCAAGCGTTAATCGGAATTACTGGGCGTAAAGCGTGCGCAGGCGGTGATGTAAGACAGGCGTGAAATCCCCGGGCTTAACCTGGGAACTGCGCTTGTGACTGCATCGCTGGAGTGCGGCAGAGGGGGATGGAATTCCGCGTGTAGCAGTGAAATGCGTAGATATGCGGAGGAACACCGATGGCGAAGGCAATCCCCTGGGCCTGCACTGACGCTCATGCACGAAAGCGTGGGGAGCAAACAGG
## 352                                                          ACGTAGGGTGCGAGCGTTAATCGGAATTACTGGGCGTAAAGCGTGCGCAGGCGGTTATGTAAGACAGATGTGAAATCCCCGGGCTCAACCTGGGAACTGCGTTTGTGACTGCATAACTAGAGTACGGCAGAGGGAGGTGGAATTCCACGTGTAGCAGTGAAATGCGTAGAGATGTGGAGGAACACCGATGGCGAAGGCAGCCTCCTGGGCCAGTACTGACGCTCATGCACGAAAGCGTGGGGAGCAAACAGG
## 353                                                          ACGTGAGCCCCAAGCGTTATCCGGAATTACTGGGCGTAAAGAGTGCGTAGGCGGTTATGTTAGTCTTCTGTTTAATCCATCGGCTCAACCGGTGAACCGCAGAGGAAACGGCATGACTGGAGTGTGTGAGAGGTTTGCGGAACTCATGGTGTAGGGGTGAAATCCGTTGATATCATGGGGAACACCAAATGCGAAGGCAGCAAACTGGCACATTACTGACGCTGAGGCACGAAAGCGTGGGTAGCGAATGGG
## 354                                                          ACGAAGGATTCAAGCGTTGTCCGGATTTACTGGGTTTAAAGGGTGCGTAGGCGGAAAGCTAAGTCAGTGGTGAAAGCCCGCAGCTCAACTGCGGAACTGCCATTGAAACTGGCCTTCTTGAATACAGTGGAGGTAGATGGAATATAACATGTAGCGGTGAAATGCTTAGATATGTTATAGAACACCGATTGCGAAGGCAGTCTGCTACACTGTCATTGACGCTGAGGCACGAAAGCGTGGGGAGCGAACAGG
## 355                                                          ACGTAGGGTGCGAGCGTTAATCGGAATTACTGGGCGTAAAGGGTGCGCAGGCGGATATATAAGTTAGATGTGAAATACCTGGGCTTAACCTAGGAACGGCATTTAATACTGTATATCTAGAGTTTGTCAGAGGGTGGTAGAATTCCAAGTGTAGCAGTGAAATGCGTAGAGATTTGGAGGAATACCGGTGGCGAAGGCGGCCACCTGGGATAAAACTGACGCTCATGCACGAAAGCGTGGGGAGCAAACAGG
## 356                                                          ACGTAGGGTGCAAGCGTTAATCGGAATTACTGGGCGTAAAGCGTGCGCAGGCGGTTGTGCAAGACAGGTGTGAAATCCCCGGGCTTAACCTGGGAACTGCACTTGTGACTGCACAGCTGGAGTACGGCAGAGGGGGATGGAATTCCGCGTGTAGCAGTGAAATGCGTAGATATGCGGAGGAACACCGATGGCGAAGGCAATCCCCTGGGCCTGTACTGACGCTCATGCACGAAAGCGTGGGGAGCAAACAGG
## 357                                                          ACGGAGGGTCCGAGCGTTATCCGGAATTATTGGGTTTAAAGGGTCCGTAGGCGGGCTATTAAGTCAGGGGTGAAAGTTTGCAGCTCAACTGTAAAATTGCCTTTGATACTGGTAGTCTTGAGTTGTATTGAAGTGGCTGGAATATGTAGTGTAGCGGTGAAATGCATAGATATTACATAGAACACCGATTGCGAAGGCAGGTCACTAAGTACACACTGACGCTGATGGACGAAAGCGTGGGGAGCGAACAGG
## 358                                                          ACGTAGGGTGCAAGCGTTAATCGGAATTATTGGGCGTAAAGGGTGCGCAGGCTGTGCTGTAAGTCAGATGTGAAATCCCCGGGCTTAACCTGGGAATTGCGTTTGAAACTGCAGTGCTAGAGTGTGATAGAGGAGAGTGGAATTCCATGTGTAGCAGTGAAATGCGTAGAGATGTGGAAGAACACCAATGGCGAAGGCAGCTCTCTGGATTAACACTGACGCTCATGCACGAAAGCGTGGGGAGCAAACAGG
## 359                                                          ACGAAGGTGGCGAGCGTTACTCGGAATTACTAGGCGTAAAGCGTGGGCAGGCGGCTTGATAAGTCTTTTGTGAAAGCCTTGGGCTTAACCCAGGGAGGCCAGAAGATACTGTCAGGCTTGGATGTGGGAGAGGAGACTGGAATTCCCGGTGTAGCGGTGAAATGCGTTGATATCGGGAGGAACACCAATGGCGAAAGCAAGTCTCTGGACCACCATCGACGCTCATCCACGAAAGCTGGGGGATCAAACAGG
## 360                                                          ACGTAGGGTGCGAGCGTTAATCGGAATTACTGGGCGTAAAGCGTGCGCAGGCGGTTGGGTAAGTCAGATGTGAAATCCCCGGGCTCAACCTGGGAACTGCATTTGAGACTGCCTAGCTGGAGTTTGGCAGAGGGGGGTGGAATTCCACGTGTAGCAGTGAAATGCGTAGAGATGTGGAGGAACACCGATGGCGAAGGCAGCCCCCTGGGCCAATACTGACGCTCATGCACGAAAGCGTGGGGAGCAAACAGG
## 361                                                          ACGGAGGGTGCGAGCGTTAATCGGAATTACTGGGCGTAAAGCGCGCGTAGGCGGTCGGCTAAGTCTGCTGTGAAAGCCCTGGGCTTAACCTGGGAACTGCAGTGGATACTGGTCGGCTAGAGTATGGTAGAGGAGAGTGGAATTTCCGGTGTAGCAGTGAAATGCGTAGAGATCGGAAGGAACACCAGTGGCGAAGGCGGCTCTCTGGACCAATACTGACGCTGAGGTGCGAAAGCGTGGGGAGCAAACAGG
## 362                                                          ACGGAGGGTGCAAGCGTTAATCGGAATAACTGGGCGTAAAGCGCACGCAGGCGGTTATATAAGTCAGATGTGAAAGCCCCGGGCTCAACCTGGGAACTGCATTTGAAACTGTGTAACTAGAGTCTTGTAGAGGGGGGTAGAATTCCAGGTGTAGCGGTGAAATGCGTAGAGATCTGGAGGAATACCGGTGGCGAAGGCGGCCCCCTGGACAAAGACTGACGCTCAGGTGCGAAAGCGTGGGGAGCAAACAGG
## 363                                                          ACGGAAGGTGCAAGCGTTAATCGGAATTACTGGGCGTAAAGCGCGCGTAGGCGGCTTGGTAAGTCGGATGTGAAAGCCCCGGGCTTAACCTGGGAACTGCATTCGATACTGCTGGGCTGGAGTACGAGAGAGGGGGGTGGAATTCCAGGTGTAGCGGTGAAATGCGTAGAGATCTGGAGGAACATCGGTGGCGAAGGCGGCCCCCTGGCTCGATACTGACGCTGAGGTGCGAAAGCGTGGGGAGCAAACAGG
## 364                                                          ACGGAGGGTGCAAGCGTTACTCGGAATCACTGGGCGTAAAGGACGCGTAGGCTGTTTAGCAAGTCAGATGTGAAATCCAATGGCTCAACCATTGAACTGCATTTGAAACTGCTAGACTAGAGTATGGGAGGGGGAGATGGAATTAGTGGTGTAGGGGTAAAATCCGTAGATATCACTAGGAATATCTAAAGCGAAGGCGATCTCCTGGAACATTACTGACGCTAAGGCGTGAAAGCGTGGGGAGCAAACAGG
## 365                                                          ACGGAGGGTGCAAGCGTTATCCGGATTTACTGGGTTTAAAGGGTGCGTAGGCGGGCAGGTAAGTCAGTGGTGAAATCCCCGAGCTTAACTCGGGAACTGCCGTTGATACTATCTGTCTTGAATATCGTGGAGGTAAGCGGAATATGTCATGTAGCGGTGAAATGCTTAGATATGACATAGAACACCAATTGCGAAGGCAGCTTGCTACACGATTATTGACGCTGAGGCACGAAAGCGTGGGGATCAAACAGG
## 366                                                          ACAGAGGATGCAAGCGTTATCCGGAATCACTGGGCATAAAGCGTCTGTAGGTGGTTTGATAAGTCTGCTGTTAAAGACTAGGGCTTAACCCTAGGACAGCAGTGGAAACTATTAGACTTGAGTATGGTAGAGGTAGAGGGAATTCCTAGTGTAGCGGTGAAATGCGTAGATATTAGGAAGAACACCAATGGCGAAAGCACTCTACTGGGCCACAACTGACACTGAGAGACGACAGCTAGGGGAGCAAATGGG
## 367                                                          ACGTAGGGTGCGAGCGTTAATCGGAATTACTGGGCGTAAAGCGTGCGCAGGCGGTTTGGTAAGACAGGCGTGAAATCCCCGGGCTCAACCTGGGAACTGCGCTTGTGACTGCCTCACTAGAGTACGGCAGAGGGGGGTGGAATTCCACGTGTAGCAGTGAAATGCGTAGAGATGTGGAGGAACACCGATGGCGAAGGCAGCCCCCTGGGCCGATACTGACGCTCATGCACGAAAGCGTGGGTAGCAAACAGG
## 368                                                          ACAGAGGGTGCAAGCGTTAATCGGAATTACTGGGCGTAAAGCGCGCGTAGGCGGTTTGTTAAGTCGGATGTGAAATCCCCGGGCTCAACCTGGGCACTGCATCCGATACTGGCTGACTAGAGTGTGGGAGAGGAAGGTAGAATTCCAGGTGTAGCGGTGAAATGCGTAGAGATCTGGAGGAATACCGATGGCGAAGGCAGCCTTCTGGCCTAACACTGACGCTGAGGTGCGAAAGCATGGGGAGCAAACAGG
## 369                                                          ACGTAGGGTGCGAGCGTTAATCGGAATTACTGGGCGTAAAGCGTGCGCAGGCGGTTTAGTAAGACAGGCGTGAAATCCCCGGGCTCAACCTGGGAACTGCGCTTGTGACTGCTAAGCTAGAGTACGGCAGAGGGGGGTGGAATTCCACGTGTAGCAGTGAAATGCGTAGAGATGTGGAGGAACACCGATGGCGAAGGCAGCCCCCTGGGCCGATACTGACGCTCATGCACGAAAGCGTGGGTAGCAAACAGG
## 370                                                          ACGTAGGGAGCGAGCGTTGTCCGGAATTACTGGGTGTAAAGGGAGTGTAGGCGGGACGGTAAGTCAGGTGTGAAATTTATGGGCTCAACTCATAAACTGCACTTGAAACTGCTGTTCTTGAGTGAAGTAGAGGTTGGCGGAATTCCTAGTGTAGCGGTGAAATGCGTAGATATTAGGAGGAACATCAGTGGCGAAGGCGGCCAACTGGGCTTTTACTGACGCTGAGGCTCGAAAGCGTGGGGAGCAAACAGG
## 371                                                           ACGTAGGGGGCAAGCGTTATCCGGAATTACTGGGCGTAAAGGGTGCGTAGGCGGTTTATCAAGTCAGGGGTGAAAGGCTACGGCTCAACCGTAGTGAGCCTCTGAAACTGGTGAACTTGAGTGTAGGAGAGGAAAGTGGAATTCCCAGTGTAGCGGTGAAATGCGTAGATATTGGGAGGAACACCAGTGGCGAAGGCGACTTTCTGGACTACAACTGACGCTGAGGCACGAAAGCGTGGGGAGCAAACAGG
## 373                                                          ACGAAGGTGGCAAGCGTTGTTCGGATTTACTGGGCGTAAAGAGTGCGTAGGCGGTTTAACAAGTCAGGAGTGAAATCCCGAGGCTCAACCTTGGAATTGCTTTTGAAACTGTTTTACTTGAGTCCGGGAGAGGTTGGCGGAATTCCGAGTGTAGAGGTGAAATTCGCAGATATTCGGAGGAACACCAGTGGCGTAAGCGGCCAACTGGACCGGTACTGACGCTGAGGCACGAAAGCGTGGGGAGCAAACAGG
## 374                                                          ACGTAGGGTGCAAGCGTTAATCGGAATTACTGGGCGTAAAGCGTGCGCAGGCGGTTTTGTAAGACTGTCGTGAAATCCCCGGGCTCAACCTGGGAATGGCGATGGTGACTGCAAGGCTAGAGTTTGGCAGAGGGGGGTAGAATTCCACGTGTAGCAGTGAAATGCGTAGATATGTGGAGGAACACCGATGGCGAAGGCAGCCCCCTGGGTCAAAACTGACGCTCATGCACGAAAGCGTGGGGAGCAAACAGG
## 375                                                          ACAGAGGGTGCGAGCGTTAATCGGAATTACTGGGCGTAAAGGGTGCGTAGGTGGTTTTCTAAGTTGTTTGTGAAATATCCGGGCTTAACCTGGGTGCTGCGAACAATACTGGGAGACTAGAGTAGGGTAGAGGGAAGCGGAATTTCCGGTGTAGCAGTGAAATGCGTAGATATCGGAAGGAACACCAGTGGCGAAGGCGGCTTCCTGGACTCATACTGACACTGAGGCACGAAAGCGTGGGGATCAAACAGG
## 376                                                          ACATAGGGTGCAAGCGTTGTCCGGAATTATTGGGCGTAAAGAGCTCGTAGGTGGTTTGTTACGTCGGATGTGAAATTCAGGGGCTCAACCCCTGACTTGCATCCGATACGGGCAAGCTAGAGTTTGGTAGGGGAGACTGGAATTCCTGGTGTAGCGGTGGAATGCGCAGATATCAGGAGGAACACCGATGGCGAAGGCAGGTCTCTGGGCCAATACTGACACTGAGGAGCGAAAGCGTGGGGAGCGAACAGG
## 377                                                          TCGAGGAAGGCAAGCGTTATCCGGAATCATTGGGCGTAAAGCGTGCGCAAGCGGTTATATAAGTGAGGTGTCAAATTCCAACGCTCAACGTTGGACTCGCGCTTCATACTGTATGACTTGAGAATAGTAGAGGCAAACGGAATTCCCGGTGTAGTAGTGAAATGCGTTAATATCGGGAGGAACGCCAAAAGCGAAAGCAGTTTGCTGGGCTATTTCTGACGCCGTTGCACGACAGCGTGGGGAGCAAACAGG
## 378                                                           ACGTAGGGGGCTAGCGTTATCCGGAATTACTGGGCGTAAAGGGTGCGTAGGTGGTTTCTTAAGTCAGAGGTGAAAGGCTACGGCTCAACCGTAGTAAGCCTTTGAAACTGGGAAACTTGAGTGCAGGAGAGGAGAGTGGAATTCCTAGTGTAGCGGTGAAATGCGTAGATATTAGGAGGAACACCAGTTGCGAAGGCGGCTCTCTGGACTGTAACTGACACTGAGGCACGAAAGCGTGGGGAGCAAACAGG
## 379                                                          ACGGGGGATGCAAGCGTTATCCGGAATGATTGGGCGTAAAGCGCCTGTAGGTTGTTTATTAAGTCCGTTGTTAAAGCCTAGGGCTCAACCCTAGAAAAGCAATGGAAACTAGTAGACTAGAGTATGGCAGGGGTAGAGGGAATTTCTAGTGTAGCGGTGAAATGCGTAGATATTAGAAAGAACACCGGTGGCGAAAGCGCTCTACTGGACCATTACTGACACTCAGAGGCGAAAGCTAGGGTAGCAAAAGGG
## 380                                                          ACGTAGGGTGCAAGCGTTAATCGGAATTACTGGGCGTAAAGCGTGCGCAGGCGGTCATGCAAGACAGATGTGAAATCCCCGGGCTCAACCTGGGAACTGCATTTGTGACTGCATGGCTTGAGTGCGGCAGAGGGGGATGGAATTCCGCGTGTAGCAGTGAAATGCGTAGATATGCGGAGGAACACCGATGGCGAAGGCAATCCCCTGGGCCTGCACTGACGCTCATGCACGAAAGCGTGGGGAGCAAACAGG
## 381                                                          ACGAAGGATGCAAGCGTTATCCGGATTCATTGGGTTTAAAGGGAGCGTAGGTGGACTGATAAGTCAGTGGTGAAATCTTCGAGCTTAACTCGGAAATTGCCATTGATACTGTCGGTCTTGAGTACAGTTGCTGTGGGCGGAATATGACATGTAGTGGTGAAATACATAGAGATGTCATAGAACACCGATTGCGAAGGCAGCTCACAAAGCTGTAACTGACACTGAGGCTCGAAAGTGTGGGGATCAAACAGG
## 382                                                          ACAGAGGGTGCGAGCGTTAATCGGATTTACTGGGCGTAAAGCGCGCGTAGGTGGTTAATTAAGTCAAATGTGAAATCCCCGAGCTTAACTTGGGAATTGCATTCGATACTGGTTAGCTAGAGTATGGGAGAGGATGGTAGAATTCCAGGTGTAGCGGTGAAATGCGTAGAGATCTGGAGGAATACCGATGGCGAAGGCAGCCATCTGGCCTAATACTGACACTGAGGTGCGAAAGCATGGGGAGCAAACAGG
## 383                                                          ACGGAGGATCCAAGCGTTATCCGGATTTATTGGGTTTAAAGGGTGCGTAGGTGGATTAATAAGTCAGTGGTGAAAGCCTGCAGCTTAACTGTAGAACTGCCATTGATACTGTTAGTCTTGAATGTAGTTGAGGTGGGCGGAATATGACATGTAGCGGTGAAATGCTTAGATATGTCATAGAACACCGATTGCGAAGGCAGCTCACTAAGCTATAATTGACACTGAGGCACGAAAGCGTGGGGAGCAAACAGG
## 384                                                          ACGGGAGTGGCAAGCGTTATCCGGAATTATTGGGCGTAAAGCGTCCGCAGGCGGTCTTGTAAGTCTGTCGTTAAAGCGTGGAGCTTAACTCCATTTGAGCGATGGAAACTGCAAGACTAGAGTGTGGTAGGGGCAGAGGGAATTCCCGGTGTAGCGGTGAAATGCGTAGATATCGGGAAGAACACCAGTGGCGAAGGCGCTCTGCTGGGCCATAACTGACGCTCATGGACGAAAGCCAGGGGAGCGAAAGGG
## 385                                                          ACGTAGGGTGCAAGCGTTAATCGGAATTACTGGGCGTAAAGCGTGCGCAGGCGGTTCTATAAGACAGAGGTGAAATCCCCGGGCTCAACCTGGGAACTGCCTTTGTGACTGTAGGGCTAGAGTGCGGCAGAGGGGGATGGAATTCCGCGTGTAGCAGTGAAATGCGTAGATATGCGGAGGAACACCGATGGCGAAGGCAATCCCCTGGGCCTGCACTGACGCTCATGCACGAAAGCGTGGGGAGCAAACAGG
## 386                                                          ACGGAGGGTGCAAGCGTTATCCGGAATCATTGGGTTTAAAGGGTCCGCAGGCGGATTTATAAGTCAGTGGTGAAATCTCACAGCTCAACTGTGAAACTGCCATTGATACTGTAAGTCTTGAATTCGGTCGAAGTGGGCGGAATATGACATGTAGCGGTGAAATGCTTAGATATGTCATAGAACACCGATAGCGAAGGCAGCTCACTAGGCCTGGATTGACGCTCAGGGACGAAAGCGTGGGGAGCAAACAGG
## 387                                                          ACGTAGGGTGCAAGCGTTAATCGGAATTACTGGGCGTAAAGCGTGCGCAGGCGGTTTTGTAAGACAGGCGTGAAATCCCCGGGCTCAACCTGGGAATGGCGCTTGTGACTGCAAGGCTAGAGTGCGGCAGAGGGGGATGGAATTCCGCGTGTAGCAGTGAAATGCGTAGATATGCGGAGGAACACCGATGGCGAAGGCAATCCCCTGGGCCTGCACTGACGCTCATGCACGAAAGCGTGGGGAGCAAACAGG
## 388                                                          ACAGAGGATGCAAGCGTTATCCGGAATCACTGGGCATAAAGCGTCTGTAGGTGGTTTGGTAAGTCTGCTGTTAAAGACTGGGGCTCAACCCCAGAAAAGCAGTGGAAACTGCTAGACTTGAGTGTGGTAGAGGTAGAGGGAATTCCTAGTGTAGCGGTGAAATGCGTAGATATTAGGAAGAACACCAATGGCGAAGGCACTCTACTGGACCATAACTGACACTGAGAGACGACAGCTAGGGGAGCAAACAGG
## 389                                                          ACGAAGGGTGCAAGCGTTGTTCGGAATCATTGGGCGTAAAGCGCGCGCAGGCGGATCAGCAAGTCAGATGTGAAATCTCGAAGCTCAACTTCGAAACTGCGTCTGAAACTGCTAGTCTAGAATGTCGGAGGGGGTAGGGGAATTTCACGTGTAGGGGTAAAATCCGTAGAGATGTGAAGGAACACCGGAGGCGAAGGCGCCTGCCTGGACGACTATTGACGCTGAGGCGCGAAAGCGTGGGGAGCAAACAGG
## 390                                                          ACGGAGGATCCGAGCGTTATCCGGATTTATTGGGTTTAAAGGGTGCGTAGGCGGAATAATAAGTCAGTTGTGAAAGTTTGCGGCTCAACCGTAAAATTGCAGTTGATACTGTTATTCTTGAGTGTACATAAGGTAGGCGGAATTCGTGGTGTAGCGGTGAAATGCTTAGATATCACGAAGAACTCCAATTGCGAAGGCAGCTTACCGGGGTACAACTGACGCTGAGGCACGAAAGTGTGGGTATCAAACAGG
## 392                                                          ACATAGGGTGCAAGCGTTGTCCGGATTTATTGGGCGTAAAGAGTTCGTAGGCGGTTTTTCGCGTCGGATGTGAAAACTCAGGGCTCAACCCTGAGCCTGCATTCGATACGGGAAGACTCGAGGACGGCAGGGGAGACTGGAACTTCTGGTGTAGCGGTGGAATGCGCAGATATCAGAAAGAACACCAATGGCGAAGGCAGGTCTCTGGGCCGATCCTGACGCTGAGGAACGAAAGCGTGGGGAGCAAACAGG
## 393                                                          ACGGAGGGTGCAAGCGTTGTCCGGAATCATTGGGCGTAAAGAGTTCGTAGGTGGCATGTTAAGTCTGGTGTTAAAGCCCGAAGCTCAACTTCGGTTCGGCACTGGATACTGGCAAGCTTGAATGCGGTAGAGGTAAAGGGAATTCCTGGTGTAGCGGTGAAATGCGTAGATATCAGGAGGAACATCGGTGGCGTAAGCGCTTTACTGGGCCGTAATTGACACTGAGGAACGAAAGCCGGGGTAGCAAATGGG
## 394                                                          ACGAAGGATGCAAGCGTTATCCGGATTCATTGGGTTTAAAGGGAGCGTAGGCGGTCTGATAAGTCAGTGGTGAAATCTCTCAGCTTAACTGAGAAACTGCCATTGATACTGTTAGACTTGAGTACGGTTGCTGTGGGCGGAATATGACATGTAGTGGTGAAATACATAGAGATGTCATAGAACACCGATTGCGAAGGCAGCTCACAAAGCCGTAACTGACGCTGAGGCTCGAAAGTGCGGGGATCAAACAGG
## 395                                                          ACAGAGACCTCAAGCGTTATCCGGATTCATTGGGCGTAAAGCGTCCGCAGGTGGTTTTTCAAGTTGGGAGTCAAATCTTTGGGCTTAACCTAGAGACTGCTTTCAATACTGGGAAACTTGAGACTGGGAGAGGTATACGGAACTGTTGGTGTAGTAGTAAAATGCGTTGATATCAACAGGAACACCAAAGGCGAAAGCAGTATACTGGAACAGTTCTGACACTCATGGACGAAAGCGTGGGGAGCGAATGGG
## 396                                                          ACGGAGGGTGCAAGCGTTATCCGGATTTATTGGGTTTAAAGGGTCCGTAGGCGGGCCGATAAGTCAGTGGTGAAAGCCCATAGCTCAACTATGGAACTGCCATTGATACTGTCGGTCTTGAGTATATGTGATGTTGGCGGAATGTGTAGTGTAGCGGTGAAATGCTTAGATATTACACAGAACACCGATTGCGAAGGCAGCTGACAAATATATAACTGACGCTGAGGGACGAAAGTGTGGGGATCAAACAGG
## 397                                                          ACAGAGGGTGCGAGCGTTAATCGGATTTACTGGGCGTAAAGCGTGCGTAGGCGGCTGATTAAGTCGGATGTGAAATCCCTGAGCTTAACTTAGGAATTGCATTCGATACTGGTCAGCTAGAGTATGGGAGAGGATGGTAGAATTCCAGGTGTAGCGGTGAAATGCGTAGAGATCTGGAGGAATACCGATGGCGAAGGCAGCCATCTGGCCTAATACTGACGCTGAGGTACGAAAGCATGGGGAGCAAACAGG
## 398                                                          ACGGAGGGTGCAAGCGTTATCCGGATTTACTGGGTTTAAAGGGTGCGTAGGCGGGCAGGTAAGTCAGTGGTGAAATCCCCGAGCTTAACTCGGGAACTGCCGTTGATACTATCTGTCTTGAATATAGTGGAGGTAAGCGGAATATGTCATGTAGCGGTGAAATGCTTAGATATGACATAGAACACCAATTGCGAAGGCAGCTTGCTACACTATTATTGACGCTGAGGCACGAAAGCGTGGGGATCAAACAGG
## 399                                                          ACGGAGGATGCAAGTGTTATCCGGAATCACTGGGCGTAAAGCGTCTGTAGGTGGTTTAATAAGTCAACTGTTAAATCTTGAGGCTCAACTTCAAAATCGCAGTCGAAACTATTAGACTAGAGTATAGTAGAGGTAAAGGGAATTTCCAGTGGAGCGGTGAAATGCGTAGAGATTGGAAAGAACACCGATGGCGAAAGCACTTTACTGGGCTATTACTAACACTCAGAGACGAAAGCTCGGGTAGCAAATGGG
## 400                                                          ACGTAGGGTGCAAGCGTTAATCGGAATTACTGGGCGTAAAGCGTGCGCAGGCGGTTTTGTAAGACAGATGTGAAAGCCCCGGGCTCAACCTGGGAAGTGCATTTGTGACTGCAAGACTAGAGTGTGTCAGAGGGGGGTGGAATTCCACGTGTAGCAGTGAAATGCGTAGAGATGTGGAGGAACACCGATGGCGAAGGCAGCCCCCTGGGATAACACTGACGCTCATGCACGAAAGCGTGGGGAGCAAACAGG
## 401                                                           ACGGAGGGTGCAAGCGTTATTCGGATTTATTGGGTTTAAAGGGTGTGTAGGCGGTCTTGTAAGTCAGTGGTGAAATCGGGAGGCTCAACTTCGCGATTGCCATTGATACTGTTAGGCTAGAGTATAGCCGGCTTGAGAGGAATGCGACAAGTAGCGGTGAAATGCTTAGATATGTCGCAGAACACCGATAGCGAAGGCATCTCAAGAGGTTATACTGACGCTGAGGCACGAAAGCGTGGGGATCAAACAGG
## 402                                                          ACGAAGGGTGCAAGCGTTGTTCGGAATCATTGGGCGTAAAGCGCGCGCAGGCGGATCAGCAAGTCAGATGTGAAATCTCGAAGCTCAACTTCGAAACTGCGTCTGAAACTGCTAGTCTAGAATGTCGGAGGGGGCAGGGGAATTTCACGTGTAGGGGTAAAATCCGTAGAGATGTGAAGGAACACCGGGGGCGAAGGCGCCTGCCTGGACGACTATTGACGCTGAGGCGCGAAAGCGTGGGGAGCAAACAGG
## 403                                                          ACGTAGGGTGCAAGCGTTAATCGGAATTACTGGGCGTAAAGCGTGCGCAGGCGGTTATGTAAGACAGAGGTGAAATCCCCGGGCTCAACCTGGGAACTGCCTTTGTGACTGCATAGCTGGAGTGCGGCAGAGGGGGATGGAATTCCGCGTGTAGCAGTGAAATGCGTAGATATGCGGAGGAACACCGATGGCGAAGGCAATCCCCTGGACCTGTACTGACGCTCATGCACGAAAGCGTGGGGAGCAAACAGG
## 404                                                          ACGAAGGGTGCAAGCGTTACTCGGAATTACTGGGCGTAAAGCGTGCGTAGGTGGTTCGTTAAGTCTGATGTGAAAGCCCTGGGCTCAACCTGGGAATGGCATTGGATACTGGCGGGCTAGAGTGCGGTAGAGGGTAGTGGAATTCCCGGTGTAGCAGTGAAATGCGTAGAGATCGGGAGGAACATCCGTGGCGAAGGCGACTACCTGGACCAGCACTGACACTGAGGCACGAAAGCGTGGGGAGCAAACAGG
## 405                                                          ACAGAGGATGCAAGCGTTATCCGGAATGATTGGGCGTAAAGCGTCTGTAGGTGGCTCTCCAAGTCTCCTGTCAAATCCCAGAGCTTAACTTTGGATCGGCACGAGAAACTCGAGAGCTTGAGTACGGTAGGGGCAGAGGGAATTCCCGGTGTAGCGGTGAAATGCGTAGAGATCGGGAAGAACACCGATGGCGAAAGCACTCTGCTGGGCCGTTACTGACACTGAGAGACGAAAGCTAAGGGAGCGAATGGG
## 407                                                          ACGAGTGCCCCAAGCGTTATCCGGAATTATTGGGTGTAAAGGGTGCGTAGGTGGTTTTATTAGTCTTGTGTTAAAGCCTGGAGCTTAACTCCAGAAATGCATAGGAAACGGTAAAACTAGAGGATGTGAGAGGTCTGTGGAACTCATAGTGTAGGGGTGAAATCCGTTGATATTATGGGGAACACCAAAAGCGAAGGCAGCAGACTGGCACATTCCTGACATTGAGGCACGAAAGCGTAGGTAGCGAATGGG
## 408                                                          ACAGAGGGGGCAAGCGTTGTCCGGAGTTACTGGGCGTAAAGCGCACGTAGGCGGTTCTGCAAGTGGTGTGTGAAACCAATCAGCTTAACTGATTGACGCCATGCCAAACTGCAGGACTCGAGTCATGGAGAGGTAGTTCGAATTGCTGGTGTAGTGGTGAAATGCGTAGATATCAGCAGGAAGACCAAGGGAGAAATCAGACTACTGGCCATGAACTGACGCTCAGGTGCGAAAGCGTGGGGAGCGAACTGG
## 409                                                          ACGGAGGGTGCAAGCGTTATCCGGATTCACTGGGTTTAAAGGGTGCGTAGGTGGGCAGTTAAGTCAGTGGTGAAATCTCCGGGCTTAACCCGGAAACTGCCATTGATACTATCTGTCTTGAATATCCTGGAGGTGAGCGGAATATGTCATGTAGCGGTGAAATGCTTAGATATGACATAGAACACCGATTGCGAAGGCAGCTCGCTACGGGATTATTGACACTGAGGCACGAAAGCGTGGGGATCAAACAGG
## 410                                                          ACGGAGGGTGCAAGCGTTATCCGGAATCATTGGGTTTAAAGGGTCCGCAGGCGGATTTATAAGTCAGTGGTGAAAGCCTATCGCTTAACGATAGAACTGCCATTGATACTGTAAGTCTTGAATTCGGTCGAAGTGGGCGGAATGTGTAGTGTAGCGGTGAAATGCATAGATATTACACAGAACACCGATAGCGAAGGCAGCTCACTAGGCCTGGATTGACGCTCAGGGACGAAAGCGTGGGGATCAAACAGG
## 411                                                          ACGGAGGGTGCAAGCGTTAATCGGAATTACTGGGCGTAAAGCGCACGCAGGCGGTCTGTCAAGTCGGATGTGAAATCCCCGGGCTCAACCTGGGAACTGCATCCGAAACTGGCAGGCTAGAGTCTTGTAGAGGGGGGTAGAATTCCAGGTGTAGCGGTGAAATGCGTAGAGATCTGGAGGAATACCGGTGGCGAAGGCGGCCCCCTGGACAAAGACTGACGCTCAGGTGCGAAAGCGTGGGGAGCAAACAGG
## 412                                                          ACATAGGGTGCAAGCGTTGTCCGGAATTATTGGGCGTAAAGAGCTCGTAGGTGGTCGATCACGTCGGATGTGAAATTCTGAGGCTTAACCTCGGACCTGCATTCGATACGGGTTGACTAGAGTGTGGTAGGGGAGACTGGAATTCCTGGTGTAGCGGTGGAATGCGCAGATATCAGGAGGAACACCGATGGCGAAGGCAGGTCTCTGGGCCATTACTGACACTGAGGAGCGAAAGCGTGGGGAGCGAACAGG
## 413                                                          ACGGAGGGTGCAAGCGTTAATCGGAATTACTGGGCGTAAAGCGCACGCAGGCGGTCAGATAAGTCAGATGTGAAAGCCCCGGGCTCAACCTGGGAACTGCATTTGAAACTGTCTGACTAGAGTCTTGTAGAGGGGGGTAGAATTCCAGGTGTAGCGGTGAAATGCGTAGAGATCTGGAGGAATACCGGTGGCGAAGGCGGCCCCCTGGACAAAGACTGACGCTCAGGTGCGAAAGCGTGGGGAGCAAACAGG
## 414                                                          ACAGAGACCCCGAGCGTTGACCGGATTTATTGGGCGTAAAGCGTACGCAGGCTGTCTTGTAAGTCATTTGTTAAACCTTCAGGCTCAACCTGGAGACTGCGGGTGATACTGCAAGACTAGAGTATGGGAGAGGTCGGCGGAATGCCTAGTGTAGGGGTAAAATCCGTTAATATTAGGTAGAACACCAAATGTGAAAACAGCCGACTGGAACATTACTGACGCTGATGTACGAAAGCATGGGTAGCGAATGGG
## 415                                                          ACAGAGGGTGCAAGCGTTAATCGGAATTACTGGGCGTAAAGCGCGCGTAGGCGGTTTGTTAAGTCGGATGTGAAATCCCTGGGCTCAACCTAGGCACTGCATCCGATACTGGCTGACTAGAGTGTGGGAGAGGAAGGTAGAATTCCAGGTGTAGCGGTGAAATGCGTAGAGATCTGGAGGAATACCGATGGCGAAGGCAGCCTTCTGGCCTAACACTGACGCTGAGGTGCGAAAGCATGGGGAGCAAACAGG
## 416                                                          ACGTAGGGTGCAAGCGTTGTCCGGAATTATTGGGCGTAAAGAGCTCGTAGGCGGTTTGTCGCGTCTGCTGTGAAATCCCGAGGCTCAACCTCGGGCCTGCAGTGGGTACGGGCAAACTAGAGTGTGGTAGGGGAGATTGGAACTCCTGGTGTAGCGGTGGAATGCGCAGATATCAGGAAGAACACCGATGGCGAAGGCAGATCTCTGGGCCATTACTGACGCTGAGGAGCGAAAGCGTGGGGAGCGAACAGG
## 417                                                           ACGGGAGGGGCAAGCGTTATCCGAAATTACTGGGCGTAAAGTGTCCGTAGATTGTAATATAAGTTATATGTTAAAATTTAAAGCTTAACTTTAAAAAAATATACAATACTGTTTTACTTGAGTTTTATACGGAAGAGTAGAATTTTACGTTAAGGAGTAAATTCCAAGAATACGTAGAGGAATATCATTAGCGAAGGCGACTCTTTAGTACAAACTGACATTGAGGGACGAAAGTGTAGGGAGCAAACAGG
## 418                                                          ACGTAGGGTGCAAGCGTTAATCGGAATTACTGGGCGTAAAGCGTGCGCAGGCGGTTATGCAAGACAGAGGTGAAATCCCCGGGCTCAACCTGGGAACTGCCTTTGTGACTGCATAGCTAGAGTACGGCAGAGGGGGATGGAATTCCGCGTGTAGCAGTGAAATGCGTAGATATGCGGAGGAACACCGATGGCGAAGGCAATCCCCTGGACCTGTACTGACGCTCATGCACGAAAGCGTGGGGAGCAAACAGG
## 419                                                          ACGTAGGGTGCAAGCGTTGTCCGGAATTATTGGGCGTAAAGAGCTCGTAGGCGGTTTGTCACGTCTGCTGTGAAATTTCGAGGCTCAACCTCGAACTTGCAGTGGGTACGGGCAGGCTAGAGTGCGGTAGGGGAGATGGGAATTCCTGGTGTAGCGGTGGAATGCGCAGATATCAGGAGGAACACCAATGGCGAAGGCACATCTCTGGGCCGTAACTGACGCTGAGGAGCGAAAGCGTGGGGAGCGAACAGG
## 420                                                          ACATAGGGTGCAAGCGTTGTCCGGAATTATTGGGCGTAAAGAGCTCGTAGGTGGTTTGATACGTCGGGTGTGAAATTCAGGGGCTCAACCCCTGACCTGCATTCGATACGGTCAAGCTAGAGTTTGGTAGGGGAGACTGGAATTCCTGGTGTAGCGGTGGAATGCGCAGATATCAGGAGGAACACCGATGGCGAAGGCAGGTCTCTGGGCCAATACTGACACTGAGGAGCGAAAGCGTGGGGAGCGAACAGG
## 421                                                          ACGAGTGCCCCAAGCGTTATCCGGAATCATTGGGCGTAAAGGGTGTGTAGGTGGTTTTGTTAGTCTTATGTTAAAACCCGAGGCTTAACTTCGGACATGCATTTGATACGGCAAAACTAGAGGATGACAGAGGTGTGCGGAACTCATGGAGTAGGGGTGAAATCCGTTGATATCATGGGGAACACCAAAAGCGAAGGCAGCACACTGGGTCATTCCTGACACTGAAACACGAAAGCGTGGGGAGCAAAAAGG
## 422                                                          ACGTAGGGTGCGAGCGTTAATCGGAATTACTGGGCGTAAAGCGTGCGCAGGCGGTTTCGTAAGACAGACGTGAAATCCCCGGGCTCAACCTGGGAACTGCCTTTGTGACTGCATAGCTAGAGTACGGCAGAGGGGGATGGAATTCCGCGTGTAGCAGTGAAATGCGTAGATATGCGGAGGAACACCGATGGCGAAGGCAATCCCCTGGGCCTGTACTGACGCTCATGCACGAAAGCGTGGGGAGCAAACAGG
## 423                                                          ACGTAGGGTGCAAGCGTTAATCGGAATTACTGGGCGTAAAGCGTGCGCAGGTGGTTGTATAAGACAGATGTGAAATCCCCGGGCTCAACCTGGGAACTGCATTTGTGACTGTACGGCTAGAGTGTGTCAGAGGGAGGTGGAATTCCACGTGTAGCAGTGAAATGCGTAGATATGTGGAGGAACACCAATGGCGAAGGCAGCCTCCTGGGATAACACTGACGCTCATGCACGAAAGCGTGGGGAGCAAACAGG
## 424                                                          ACGTAGGGTGCAAGCGTTAATCGGAATTACTGGGCGTAAAGCGTGCGCAGGCGGACTTTTAAGCCAGATGTGAAAGCCCCGAGCTTAACTTGGGAATTGCGTTTGGAACTGGGAGTCTAGAGTCTGTCAGAGGGGGATGGAATTCCACGTGTAGCAGTGAAATGCGTAGAGATGTGGAGGAACACCGATGGCGAAGGCAATCCCCTGGACCTGTACTGACGCTCATGCACGAAAGCGTGGGGAGCAAACAGG
## 425                                                          ACATAGGGTGCAAGCGTTGTCCGGAATTATTGGGCGTAAAGAGCTCGTAGGTGGTTTGTTACGTCGGATGTGAAATGCAGGGGCTCAACCCCTGACTTGCATCCGATACGGGCAAGCTAGAGTTTGGTAGGGGAGACTGGAATTCCTGGTGTAGCGGTGGAATGCGCAGATATCAGGAGGAACACCGATGGCGAAGGCAGGTCTCTGGGCCAATACTGACACTGAGGAGCGAAAGCGTGGGGAGCGAACAGG
## 426                                                          ACGTGAGGTGCTAACATTACCCGGATTTATTGGGCGTAAAGAGTTCGTAGGCGGCAAGGTAAGTCAGATATTAAATCCTGAGGCTCAACTTCAGGTCTGTATCTGAAACTGTCTAGCTAGAGTCTTGGAGAGGTGAGTGGAATTCCTCATGTAGGGGTAAAATCCGTAGATATGAGGAGGAACACCAAAAGCGAAGGCAGCTCACTGGCCAAGTACTGACGCTAAAGAACGAAAGCATGGGTAGCGAACGGG
## 427                                                          ACGTAGGGTGCAAGCGTTAATCGGAATTACTGGGCGTAAAGCGCGCGTAGGCGGTTAATTAAGCGAGATGTGAAATCCCTGGGCTCAACCTAGGAATTGCGTTTCGAACTGATTAGCTAGAGTGCAGTAGAGGGTGGTGGAATTTCCGGTGTAGCGGTGAAATGCGTAGAGATCGGAAGGAACATCAGTGGCGAAGGCGACTGCCTGGACTGACACTGACGCTGAGGTGCGAAAGCGTGGGGAGCAAACAGG
## 428                                                          ACGTAGGGTGCGAGCGTTAATCGGAATTACTGGGCGTAAAGCGTGCGCAGGCGGTTGTGTAAGACAGATGTGAAATCCCCGGGCTCAACCTGGGAACTGCGTTTGTGACTGCACAACTAGAGTACGGCAGAGGGAGGTGGAATTCCGCGTGTAGCAGTGAAATGCGTAGAGATGCGGAGGAACACCGATGGCGAAGGCAGCCTCCTGGGCCAGTACTGACGCTCATGCACGAAAGCGTGGGGAGCAAACAGG
## 429                                                          ACGTAGGGTGCAAGCGTTAATCGGAATTACTGGGCGTAAAGCGTGCGCAGGCGGTTCGGAAAGAAAGGTGTGAAATCCCAGGGCTTAACCTTGGAACTGCACTTTTAACTACCGAGCTAGAGTATGTCAGAGGGGGGTGGAATTCCGCGTGTAGCAGTGAAATGCGTAGATATGCGGAGGAACACCGATGGCGAAGGCAATCCCCTGGACCTGTACTGACGCTCATGCACGAAAGCGTGGGGAGCAAACAGG
## 430                                                          ACGAAGGATGCAAGCGTTATCCGGATTCATTGGGTTTAAAGGGAGCGTAGGCGGTCTTATAAGTCAGTGGTGAAAGCTCTTCGCTTAACGAAGAAATTGCCATTGATACTGTAGGACTTGAGTACAGTTGCTGTGGGCGGAATATGACATGTAGTGGTGAAATACATAGAGATGTCATAGAACACCGATTGCGAAGGCAGCTCACAAAACTGTAACTGACGCTGAGGCTCGAAAGTGCGGGGATCAAACAGG
## 431                                                          ACGGAGGGTGCAAGCATTAATCGGAATTATTGGGCGTAAAGGGCGCGTAGGCGGATAGGATAGTCAGATGTGAAATTTCGGAGCTCAACTTCGAAGCTGCATTTGAAACTACTTATCTGGAGGGTAGGCGGAGAAAACGGAATTCCACATGTAGCGGTGAAATGCGTAGAGATGTGGAAGAACACCTGTGGCGAAAGCGGTTTTCTAGCTTATTCCTGACGCTGAGGCGCGAAAGCAAGGGGAGCAAACAGG
## 432                                                          ACGGAGGATCCAAGCGTTATCCGGATTTATTGGGTTTAAAGGGTGCGTAGGCGGGCCCTTAAGTCAGTGGTGAAAGTTTGCAGCTTAACTGTAAAATTGCCATTGAAACTGAGGGTCTTGAGTGTAAATAAGGTAGGCGGAATGTGTTGTGTAGCGGTGAAATGCTTAGATATAACACAGAACACCAATTGCGAAGGCAGCTTACTGGGATACAACTGACGCTGAGGCACGAAAGCGTGGGGATCAAACAGG
## 433                                                          ACGGAGGGTGCAAGCGTTAATCGGAATAACTGGGCGTAAAGCGCACGCAGGCGGTCGGATAAGTCAGATGTGAAAGCCCCGGGCTCAACCTGGGAACTGCATTTGAAACTGTTCGACTAGAGTCTTGTAGAGGGGGGTAGAATTCCAGGTGTAGCGGTGAAATGCGTAGAGATCTGGAGGAATACCGGTGGCGAAGGCGGCCCCCTGGACAAAGACTGACGCTCAGGTGCGAAAGCGTGGGGAGCAAACAGG
## 434                                                          ACGAAGGGTGTAAGCGTTGTTCGGATTTATTGGGCGTAAAGCGCGCGCAGGTGGATTTTTAAGTCAGATGTGAAATCTCGGGGCTCAACCCCGAACGTGCGTCTGAAACTGGAGATCTAGAATATTGGAGGGGGTAGAGGAATTTCACATGTAGGGGTAAAATCCGTAGAGATGTGAAGGAACACCAGAGGCGAAGGCGTCTACCTGGCCAAACATTGACACTGAGGCGCGAAAGCGTGGGGAGCAAACAGG
## 435                                                          ACGTAGGGTGCGAGCGTTAATCGGAATTACTGGGCGTAAAGCGTGCGCAGGCGGCTTTGCAAGACAGAGGTGAAATCCCCGGGCTCAACCTGGGAACTGCCTTTGTGACTGCAAGGCTAGAGTACGGCAGAGGGGGATGGAATTCCGCGTGTAGCAGTGAAATGCGTAGATATGCGGAGGAACACCGATGGCGAAGGCAATCCCCTGGGCCTGTACTGACGCTCATGCACGAAAGCGTGGGGAGCAAACAGG
## 436                                                          ACGGAGGATCCAAGCGTTATCCGGATTTATTGGGTTTAAAGGGTCCGTAGGCGGGTCTTTAAGTCAGTGGTGAAAGCCTGCAGCTTAACTGTGGAAATGCCATTGATACTGGAGGCCTTGAGTGTAGTAGAAGTAGGCGGAATAGGGCATGTAGCGGTGAAATGCATAGATATGCCCTAGAACACCGATTGCGAAGGCAGCTTACTATGTTACAACTGACGCTGAGGGACGAAAGCGTGGGGAGCAAACAGG
## 437                                                          ACGAAGGGTGCAAGCGTTATTCGGAATGACTGGGCGTAAAGAGCGTGAAGGCGGCTTCTTAAGTCAACTGTTAAAGGGCTCGGCTTAACTGAGCAACTGCGGGTGAAACTGAGAAGCTAGAGTGCAGAAGAGAAAGGTAGAATTCTCGGAGTAGCGGTAAAATGCGTAGATCTCGAGAGGAATACCGGTTGCGAAGGCGGCCTTTTGGTCTGTAACTGACGCTCAAGCGCGAAAGCGTGGGGAGCAAACAGG
## 439                                                          ACGGAGGGTGCAAGCGTTACTCGGAATCACTGGGCGTAAAGGATGCGTAGGCTGGAAATCAAGTCGAGAGTGAAATCCAACGGCTCAACCGTTGAACTGCTCTCGAAACTGATTACCTAGAATATGGGAGAGGTAGATGGAATTGGTGGTGTAGGGGTAAAATCCGTAGATATCACCAGGAATACCGATTGCGAAGGCGATCTACTGGAACATTATTGACGCTGAGGCATGAAAGCGTGGGGAGCAAACAGG
## 440                                                          ACGGAGGATGCAAGTGTTATCCGGAATCACTGGGCGTAAAGCGTCTGTAGGTGGTCAAATAAGTCAACTGTTAAATCTTGAGGCTCAACCTCAAAATCGCAGTCGAAACTATTAGACTAGAGTATAGTAGGGGTAAAGGGAATTTCCAGTGGAGCGGTGAAATGCGTAGAGATTGGAAAGAACACCGATGGCGAAGGCACTTTACTGGGCTATTACTAACACTGAGAGACGAAAGCTAGGGTAGCAAATGGG
## 441                                                          ACGTAGGGTGCAAGCGTTAATCGGAATTACTGGGCGTAAAGCGTGCGCAGGCGGTTGTGCAAGACAGATGTGAAATCCCCGGGCTTAACCTGGGAATGGCATTTGTGACTGCACGGCTAGAGTGTGTCAGAGGGGGGTAGAATTCCACGTGTAGCAGTGAAATGCGTAGATATGTGGAGGAATACCGATGGCGAAGGCAGCCCCCTGGGATAACACTGACGCTCATGCACGAAAGCGTGGGGAGCAAACAGG
## 442                                                          ACAGAGACTGCAAGCGTTATTCGGATTCACTGGGCGTAAAGGGTGCGCAGGCGGTTGCGTGTGTTGGATGTGAAAGCCCGGGGCTCAACCCCGGAATTGCGTCCAAAACTACGCGACTAGAGTACCGGAGAGGATAGCGGAATTCACGGTGTAGCAGTGAAATGCGTAGATATCGTGAAGAACACCAGCGGCGAAGGCGGCTATCTGGAAGGTAACTGACGCTCAGGCACGAAAGCGTGGGGAGCAAAAGGG
## 443                                                          ACAGAGGGTGCAAACGTTGCTCGGAATTACTGGGCGTAAAGCGCGTGTAGGTGGACCAGCAAGTCAGACGTGAAAGCCCTGGGCTCAACCCAGGAACTGCATTTGAAACTGCTGGTCTGGAGTGGCGGAGAGGATCGTGGAATTCCTGGTGTAGAGGTGAAATTCGTAGATATCAGGAGGAACACCGGTGGCGAAGGCGGCGATCTGGACGCTTACTGACACTGAGACGCGAAAGCGTGGGGAGCAAACAGG
## 444                                                          ACAGAGGTGGCAAGCGTTGTTCGGATTTATTGGGTGTAAAGGGCAGGTAGGCGGCTAAGTAAGTCTGTTGTGAAATCCCGGGGCTCAACCCCGGAACTGCAACGGAAACTACTTAGCTGGAACATAGTAGGGGTGAGGGGAATTCTCGGTGTAAGGGTGAAATCTGTAGATATCGAGAGGAACACCAATGGCGAAGGCACCTCACTGGACTATTGTTGACGCTGAGCTGCGAAAGTAGGGGGAGCAAACAGG
## 445                                                         ACGAGGGCCCCGAGCGTTATCCGGAATTATTGGGCGTAAAGGGTGTGTAGGTGGTCATATTAGTCTTGTGTAAAAGCCTGTCGCCTTACGACAGATCCGCACGGGAAACGGTATGACTGAGAGGGTGCAAGAGGTATATGGAACTCACGGTGTAGGGGTGAAATCCGTTGATATCGTGGGGAACGCCAAAAGCGAAAGCAATATACTGGTGCATACCTGACACTGAAACACGAAAGCCAGGGTAGCGAATGGG
## 447                                                          ACGTAGGTGGCGAGCGTTGTCCGGATTTATTGGGTTTAAAGGGTGCGTAGGCGGCTTATTAAGTCAGTGGTGAAAGACGGCAGCTTAACTGTCGAGGTGCCATTGATACTGATGAGCTAGAGTACTGATGAGGTAGGCGGAATGGACGGTGTAGCGGTGAAATGCATAGATATCGTCCAGAACACCGATAGCGAAGGCAGCTTACTAAGGAGTAACTGACGCTGAGGCACGAAAGTGTGGGGATCAAACAGG
## 448                                                          ACGAGTGCCCCGAGCGTTATCCGGATTTATTGGGCGTAAAGCGTCCGCAGGCGGTCTGTAACATCTTTTGTTAAATCACAAGGCTTAACTTTGTGACCGCGAGAGATATGGACAGACTAGAGACTGGGAGAGGTAAGCGGAATTGCTGGTGTAGGGGTTAAATCCGTTAATATCAGCAGGAACACCAAATGCGAAGGCAGCTTACTGGAACAGTTCTGACGCTCATGGACGAAAGCGTGGGTAGCGAATGGG
## 449                                                          ACGGAGGGGGCTAGCGTTGTTCGGAATTACTGGGCGTAAAGCGCACGTAGGCGGACTGGAAAGTCAGGGGTGAAATCCCGGGGCTCAACCCCGGAACTGCCTTTGAAACTCCCAGTCTTGAGGTCGAGAGAGGTGAGTGGAATTCCGAGTGTAGAGGTGAAATTCGTAGATATTCGGAGGAACACCAGTGGCGAAGGCGGCTCACTGGCTCGATACTGACGCTGAGGTGCGAAAGCGTGGGGAGCAAACAGG
## 450                                                          ACAGAGGGTGCGAGCGTTGTCCGGAATCACTGGGCGTAAAGGGCGCGTAGGTGGCTTGGTAAGCGTGTGGTGAAAGTCCGGGGCTCAACCCCGGATCTGCCGTGCGAACTGCTGAGCTCGAGCGATGTAGAGGCAGGTGGAATTTCGGGTGTAGCGGTGGAATGCGTAGATATCCGAAAGAACACCAGTGGCGAAGGCGGCCTGCTGGGCATCAGCTGACACTGAGGCGCGACAGCGTGGGGAGCAAACAGG
## 451                                                          ACAGAGACCTCAAACATTGTCCGGATTCATTGGGCGTAAAGCGTCCGCAGGTGGCTTAATAAGTTAGGGGTTAAAGCTTCCGACCTAATCGGAAAACTGCTTCTAATACTATTAAGCTCGAGGTTGGGAGAGGCATATGGAACTGCCGGTGTAGTAGTAAAATGCGTTGATATCGGCAGGAACACCAAAGGCGAAGGCAATATGCTGGAACAACTCTGACACTCAGGGACGAAAGCGTGGGGAGCGAATGGG
## 452                                                          ACGTAGGGTGCGAGCGTTAATCGGAATTACTGGGCGTAAAGCGTGCGCAGGCGGTTTGTTAAGACAGTTGTGAAATCCCCGGGCTTAACCTGGGAACTGCAATTGTGACTGGCAGGCTAGAGTTTGGCAGAGGGGGGTGGAATTCCTGGTGTAGCAGTGAAATGCGTAGATATCAGGAGGAACACCGATGGCGAAGGCAGCCCCCTGGGCCATGACTGACGCTCATGCACGAAAGCGTGGGGAGCAAACAGG
## 453                                                           ACGTAGGTGGCGAGCGTTGTCCGGAATTATTGGGCGTAAAGGGAGCGCAGGTGGGCTGTCAAGTCTGTCTTAAAAGTGCGGGGCTTAACCCCGTGATGGGATAGAAACTGGCAGTCTTGAGTGCAGGAGAGGAAAGCGGAATTCCTAGTGTAGCGGTGAAATGCGTAGATATTAGGAGGAACACCAGTGGCGAAGGCGGCTTTCTGGACTGTAACTGACACTGAGGCTCGAAAGCCAGGGGAGCGAACGGG
## 454                                                          ACAGAGGTCCCAAGCGTTGTTCGGATTCACTGGGCGTAAAGGGTGCGTAGGCGGTGGGGTAAGTTTGAGGTGAAATCTCCGGGCTCAACCCGGAAAATGCCTTGAAGACTATCCTGCTAGAGGATCGGAGGGGAGACTGGAATTCTCGGTGTAGCAGTGAAATGCGTAGATATCGAGAGGAACACCAGTGGCGAAGGCGAGTCTCTGGACGATTCCTGACGCTGAGGCACGAAAGCCAGGGGAGCAAACGGG
## 455                                                          ACGGAGGGTGCAAGCGTTGTCCGGATTTATTGGGTTTAAAGGGTGCGTAGGCGGCTGAATAAGTCAGCGGTGAAAGACTCCGGCTTAACCGGAGCAGTGCCGTTGATACTGTTTAGCTTGAGTTCTGCAGGGGTACATGGAATTGATGATGTAGCGGTGAAATGCATAGATATCATCAGGAACACCGATAGCGAAGGCATTGTACTGGGCAGCGACTGACGCTGAGGCACGAAAGTGTGGGGATCGAACAGG
## 456                                                          ACATAGGGTGCAAGCGTTGTCCGGAATTATTGGGCGTAAAGAGCTCGTAGGTGGTTCGTTACGTCGGATGTGAAAACCTGAGGCTCAACCTCAGGCCTGCATTCGATACGGGCGAACTGGAGTGTGGTAGGGGAGACTGGAATTCCTGGTGTAGCGGTGGAATGCGCAGATATCAGGAGGAACACCAATGGCGAAGGCAGGTCTCTGGGCCACTACTGACACTGAGGAGCGAAAGCGTGGGGAGCAAACAGG
## 457                                                          ACGTAGGGGGCGAGAGTTGTCCGGATTTACTGGGTGTAAAGGGTGCTCAGGTGGATTTGTAAGTCAGAGGTGAAATCCCAAAGCTCAACTTTGGTGCTGCCTTTGATACTGCAAGTCTTGAGTTTGGAAGAGGGTAATGGAATATCTGGTGTAGCAGTGAAATGCGTAGATATCAGATAGAACACCAATGGCGAAGGCAGTTACCTGGTCCAAAACTGACACTAAAGCACGAAAGTGTGGGGAGCAAACAGG
## 458                                                          ACAGAGGGTGCAAGCGTTAATCGGAATTACTGGGCGTAAAGCGCACGTAGGCGGCTACGTAAGTCAGGTGTGAAATCCCCGGGCTCAACCTGGGAACTGCACTTGAGACTGCGTGGCTAGAGTATGGGAGAGGAAGGCGGAATTCCCGGTGTAGCGGTGAAATGCGTAGATATCGGGAGGAACATCAGTGGCGAAGGCGGCCTTCTGGACCAATACTGACGCTCAGGTGCGAAAGCGTGGGGAGCAAACAGG
## 459                                                          ACGAAGGTGGCAAGCGTTGTTCGGATTTACTGGGCGTAAAGAGTGCGTAGGCGGTTTAATAAGTCAGAAGTGAAAGCCCAGGGCTCAACCTTGGAATTGCTTTTGAAACTGTTTTACTTGAATCCGGTAGAGGTTGGCGGAATTCTCAGTGTAGAGGTGAAATTCGCAGATATTGAGAGGAACACCAGTGGCGTAAGCGGCCAACTGGACCGGTATTGACGCTGAGGCACGAAAGCATGGGGAGCAAACAGG
## 460                                                          ACGTAGGGTGCGAGCGTTAATCGGAATTACTGGGCGTAAAGGGTGCGCAGGCGGTTTTGTAAGTCAGATGTGAAATCCCCGGGCTTAACCTGGGAATTGCGTTTGAAACTACAAATCTAGAGTGTGGCAGAGGGAGGTGGAATTCCATGTGTAGCAGTGAAATGCGTAGAGATATGGAAGAACATCGATGGCGAAGGCAGCCTCCTGGGTTAACACTGACGCTCATGCACGAAAGCGTGGGGAGCAAACAGG
## 461                                                          ACGTAGGGTGCAAGCGTTAATCGGAATTACTGGGCGTAAAGCGTGCGCAGGCGGTTATGTAAGACAGAGGTGAAATCCCCGGGCTCAACCTGGGAACTGCCTTTGTGACTGCATAGCTAGAGTACGGTAGAGGGGGATGGAATTCCGCGTGTAGCAGTGAAATGCGTAGATATGCGGAGGAACACCGATGGCGAAGGCAATCCCCTGGGCCTGTACTGACGCTCATGCACGAAAGCGTGGGGAGCAAACAGG
## 462                                                          ACGGAGGGTGCAAGCGTTATCCGGAATCATTGGGTTTAAAGGGTCCGCAGGCGGACTTATAAGTCAGTGGTGAAAGCCTACAGCTCAACTGTAGAACTGCCATTGATACTGTAAGTCTTGAATATGTTCGGAGTGGGCGGAATGTGACATGTAGCGGTGAAATGCTTAGATATGTCACAGAACACCGATAGCGAAGGCAGCTCACTAGACCATTATTGACGCTCATGGACGAAAGCGTGGGGAGCAAACAGG
## 463                                                          ACGTAGGGTGCAAGCGTTAATCGGAATTACTGGGCGTAAAGCGTGCGCAGGCGGTGATGTAAGACAGAGGTGAAATCCCCGGGCTCAACCTGGGAACTGCCTTTGTGACTGCATCGCTGGAGTACGGCAGAGGGGGATGGAATTCCGCGTGTAGCAGTGAAATGCGTAGATATGCGGAGGAACACCGATGGCGAAGGCAATCCCCTGGGCCTGTACTGACGCTCATGCACGAAAGCGTGGGGAGCAAACAGG
## 464                                                          ACGGAGGGTGCAAGCGTTATCCGGATTCACTGGGTTTAAAGGGTGTGTAGGCGGGCTTGTAAGTCCGAGGTGAAATCTCCGAGCTCAACTTGGAAACTGCCTTGGATACTATAGGTCTTGAATATCGTTGAGGTAGGCGGAATATGTCATGTAGCGGTGAAATGCTTAGATATGACATAGAACACCGATTGCGAAGGCAGCTTGCTAAACGATTATTGACGCTGAGGCACGAAAGCGTGGGGATCAAACAGG
## 466                                                          ACGAAGGATCCAAGCGTTGTCCGGATTTACTGGGTTTAAAGGGTGCGTAGGCGGGAAATTAAGTCAGTGGTGAAAGCTGGTAGCTCAACTATCAAATTGCCATTGAAACTGATTTTCTCGAGTACGGTTGAGGTAGCTGGAATGTAACATGTAGCGGTGAAATGCTTAGATATGTTACAGAACACCAATTGCGAAGGCAGGCTACTAAACCGTAACTGACGCTGAGGCACGAAAGCGTGGGGATCAAACAGG
## 467                                                          ACGGAGGGTGCAAGCGTTATCCGGATTCACTGGGTTTAAAGGGTGCGTAGGCGGGCAGGTAAGTCAGTGGTGAAATCTTCGAGCTTAACTCGGAAACTGCCGTTGATACTATCTGTCTTGAATTTAGTGGAGGTAAGCGGAATATGTCATGTAGCGGTGAAATGCTTAGATATGACATAGAACACCAATTGCGAAGGCAGCTTGCTACACTAATATTGACGCTGAGGCACGAAAGCGTGGGGATCAAACAGG
## 468                                                          ACGGAGGGTGCAAGCGTTACTCGGAATCACTGGGCGTAAAGAGCGTGTAGGCGGATATATAAGTCAGAAGTGAAATCCAATAGCTTAACTATTGAACTGCTTTTGAAACTGTATATCTAGAATGTGGGAGAGGTAGATGGAATTTCTGGTGTAGGGGTAAAATCCGTAGAGATCAGAAGGAATACCGATTGCGAAGGCGATCTACTGGAACATTATTGACGCTGAGACGCGAAAGCGTGGGGAGCAAACAGG
## 469                                                          ACGGAGGGTGCTAGCGTTGTTCGGAATTACTGGGCGTAAAGCGCGCGTAGGCGGTCTAGTAAGTTGGAAGTGAAATCCCAGAGCTTAACTCTGGAATTGCTTTCAAAACTGTTAGACTTGAGAATGGTAGGGGAAAGTGGAATTCCTAGTGTAGAGGTGAAATTCGTAGATATTAGGAGGAACACCAGAGGCGAAAGCGGCTTTCTGGACCATTTCTGACGCTAAGGCGCGAAAGCGTGGGGAGCAAACAGG
## 470                                                          ACGGAGGATCCGAGCGTTATCCGGATTTATTGGGTTTAAAGGGAGCGTAGGCGGACGCTTAAGTCAGTTGTGAAAGTTTGCGGCTCAACCGTAAAATTGCAGTTGATACTGGGTGTCTTGAGTACAGTAGAGGCAGGCGGAATTCGTGGTGTAGCGGTGAAATGCTTAGATATCACGAAGAACTCCGATTGCGAAGGCAGCTTGCTGGACTGTAACTGACGCTGATGCTCGAAAGTGTGGGTATCAAACAGG
## 471                                                          ACAGAGGATGCAAGCGTTATCCGGAATCACTGGGCATAAAGCGTCTGTAGGTTGCTTGCCAAGTCTGCTGTTAAAGATCAGGGCTTAACCCTGGGAAAGCAGTGGAAACTAGTAGGCTTGAGTGTGGTAGAGGTAGAGGGAATTCCTGGTGTAGCGGTGAAATGCGTAGATATTAGGAAGAACACCAATGGCGAAAGCACTCTACTGGGCCACAACTGACACTGAGAGACGACAGCTAGGGGAGCAAATGGG
## 472                                                          ACGAAGGATCCAAGCGTTGTCCGGATTTACTGGGTTTAAAGGGTGCGTAGGCGGACTTTTAAGTCAGTGGTGAAAGCCGGTAGCTTAACTATCGAATTGCCATTGAAACTGAAAGTCTCGAGTATGGTTGAGGTGGCCGGAATGTATCATGTAGCGGTGAAATGCTTAGATATGATACAGAACACCAATTGCGAAGGCAGGTCACTAAGCCATAACTGACGCTGAGGCACGAAAGCGTGGGGAGCAAACAGG
## 473                                                          ACGTAGGGTGCGAGCGTTAATCGGAATTACTGGGCGTAAAGCGTGCGCAGGCGGTTATGCAAGACAGAGGTGAAATCCCCGGGCTCAACCTGGGAACTGCCTTTGTGACTGCATGACTAGAGTACGGCAGAGGGAGATGGAATTCCGCGTGTAGCAGTGAAATGCGTAGATATGCGGAGGAACACCGATGGCGAAGGCAATCTCCTGGGCCTGTACTGACGCTCATGCACGAAAGCGTGGGGAGCAAACAGG
## 474                                                           ACGTATGTCGCAAGCGTTATCCGGAATTATTGGGCATAAAGGGCGTCTAGGCGGTTTAGAAAGTCTGATGTGAAAATGCGGGGCTCAACCCCGTATTGCGTTGGAAACTACTAGACTAGAGTATTGGAGAGGTAAGCGGAACTACAAGTGTAGAGGTGAAATTCGTAGATATTTGTAGGAATGCCAATTGGGAAGCCAGCTTACTGGACAAATACTGACGCTGAAGCGCGAAAGCTAGGGGAGCAAACAGG
## 475                                                          ACGGAGAGTGCAAACGTTGTTCGGAATTATTGGGCGTAAAGGGCGCGTAGGCGGCTCGGTAAGTCAGATGTGAAAGCCCGGGGCTCAACCCTGGAAGTGCGTTTGAAACTGCTGAGCTTGAGTGCAGGAGGGAGAGGTGGAATTCCAGGTGTAGGAGTGAAATCCGTAGATATCTGGAGGAACACCGGTGGCGAAGGCGACCTCTTGGCCTGAAACTGACGCTGAGGCGCGAAGGCGTGGGGAGCAAACAGG
## 476                                                          ACAGAGGATGCAAGCGTTATCCGGAATCACTGGGCATAAAGCGTCTGTAGGTTGCTTAGCAAGTCTGCTGTTAAAGATCAGGGCCTAACCCTGGGAAAGCAGTGGAAACTAGTAGGCTTGAGTGTGGTAGAGGTAGAGGGAATTCCTGGTGTAGCGGTGAAATGCGTAGATATTAGGAAGAACACCAATGGCGAAAGCACTCTACTGGGCCACAACTGACACTAAGAGACGACAGCTAGGGGAGCAAATGGG
## 478                                                          ACGTAGGGTGCAAGCGTTAATCGGAATTACTGGGCGTAAAGCGTGCGCAGGCGGTTGTGCAAGACAGGTGTGAAATCCCCGGGCTTAACCTGGGAACTGCACTTGTGACTGCACAGCTTGAGTGCGGCAGAGGGGGATGGAATTCCGCGTGTAGCAGTGAAATGCGTAGATATGCGGAGGAACACCGATGGCGAAGGCAATCCCCTGGGCCTGCACTGACGCTCATGCACGAAAGCGTGGGGAGCAAACAGG
## 480                                                          ACGTGAGTGGCAAGCGTTATCCGGAATCATTGGGCGTAAAGCGTCAGCAGGGGGATGTTCAAGTGAAATGTTAAAGCCTTCGACCCAATCGAAGATCTGTGTTTCATACTGTTCATCTAGAAGATAGGAGAGGTAAATAGAATTTCCGGAGTAGGGGTAATATCCGTAGATACCGGAAGGAACACCAATAGCGAAGGCAATTTACTGGCCTATTCTTGACCCTCAGTGACGAAAGCGTGGGTAGCAAACAGG
## 481                                                          ACAGAGGGTGCAAGCGTTAATCGGAATTACTGGGCGTAAAGCGCGCGTAGGCGGCTGCCTAAGTCGGATGTGAAATCCCCGGGCTTAACCTGGGAACTGCATTCGATACTGGGCGGCTAGAGTATGAGAGAGGGAGGTAGAATTCCACGTGTAGCGGTGAAATGCGTAGATATGTGGAGGAATACCGGTGGCGAAGGCGGCCTCCTGGCTTAATACTGACGCTGAGGTGCGAAAGCGTGGGGAGCAAACAGG
## 482                                                          ACGAAGGGACCTAGCGTAGTTCGGAATTACTGGGCGTAAAGCGCGCGTAGGCCGTTGAGTTAGTTAATTGTGAAATCCCAAAGCTTAACTTTGGAACTGCAATTAAAACTGCTCGACTAGAGTTTGATAGAGGAAAGCGGAATACATAGTGTAGAGGTGAAATTCGTAGATATTATGTAGAACACCAGTTGCGAAGGCGGCTTTCTGGATCAACACTGACGCTGAGGCGCGAAAGTATGGGTAGCAAAGAGG
## 483                                                          ACGAGTGCCCCAAGCGTTATCCGGAATTATTGGGCGTAAAGGGTGCGTAGGTGGTTTGATTAGTCGTTTGTTAAAAGTCCCGGCTTAACCGGGAAGATGCAGGCGAAACGGTCAGACTAGAGAGAGTGAGAGGCCGGTGGAACTCATGGTGTAGGGGTGAAATCCGTTGATATCATGGGGAACGCCAAAAGCGAAGGCAGCCGGCTGGCGCTTATCTGACACTGAGGCACGAAAGCGTGGGTAGCGAACGGG
## 484                                                          ACGGGAGTGGCAAGCGTTATCCGGAATTATTGGGCGTAAAGCGTCCGCAGGCGGTTTTACAAGTCTGTCGTTAAAGCGTGGAGCTTAACTCCATTTCAGCGATGGAAACTGTAAGACTAGAGTGTGGTAGGGGCAGAGGGAATTCCCGGTGTAGCGGTGAAATGCGTAGATATCGGGAAGAACACCAGTGGCGAAGGCGCTCTGCTGGGCCATAACTGACGCTCATGGACGAAAGCCAGGGGAGCGAAAGGG
## 485                                                          ACGGAGGGTGCAAGCGTTATCCGGATTCACTGGGTTTAAAGGGTGCGTAGGTGGGTTGATAAGTCAGTGGTGAAAGCTCCGAGCTTAACTTGGAAACTGCCATTGATACTGTCAGTCTTGAATGTCGTAGAGGTAAGCGGAATGTTGCATGTAGCGGTGAAATGCTTAGATATGTTACAGAACACCAATTGCGAAGGCAGCTTACTATGCGATGATTGACGCTGAGGCACGAAAGCGTGGGGAGCAAACAGG
## 486                                                          ACGTAGGGTGCAAGCGTTAATCGGAATTACTGGGCGTAAAGCGTGCGCAGGCGGTCTTGTAAGACAGAGGTGAAATCCCCGGGCTCAACCTGGGAACTGCCTTTGTGACTGCAAGGCTTGAGTGCGGCAGAGGGGGATGGAATTCCGCGTGTAGCAGTGAAATGCGTAGATATGCGGAGGAACACCGATGGCGAAGGCAATCCCCTGGGCCTGCACTGACGCTCATGCACGAAAGCGTGGGGAGCAAACAGG
## 487                                                          ACGTAGGGTGCGAGCGTTAATCGGAATTACTGGGCGTAAAGCGTGCGCAGGCGGTTTCGTAAGACAGAGGTGAAATCCCCGGGCTCAACCTGGGAACTGCCTTTGTGACTGCATAGCTAGAGTACGGCAGAGGGGGATGGAATTCCGCGTGTAGCAGTGAAATGCGTAGATATGCGGAGGAACACCGATGGCGAAGGCAATCCCCTGGGCCTGTACTGACGCTCATGCACGAAAGCGTGGGGAGCAAACAGG
## 488                                                          ACGTAGGGTGCGAGCGTTAATCGGAATTACTGGGCGTAAAGGGTGCGCAGGTGGTTTTGCAAGACTGACGTGAAAGCCCCGGGCTCAACCTGGGAACGGCGTTAGTGACTGCAAGACTAGAGTGTGGCAGAGGGGGGTGGAATTCCACGTGTAGCAGTGAAATGCGTAGAGATGTGGAGGAACACCGATGGCGAAGGCAGCCCCCTGGGCTAACACTGACACTCATGCACGAAAGCGTGGGGAGCAAACAGG
## 490                                                          ACGAAGGGTGCAAGCGTTGTTCGGAATCATTGGGCGTAAAGCGCGCGCAGGCGGATTACTAAGTCAGATGTGAAATCTCGGGGCTCAACCCCGAAACTGCGTCTGAAACTGGTAGTCTAGAATGTCGGAGGGGGCAGGGGAATTTCACGTGTAGGGGTAAAATCCGTAGAGATGTGAAGGAACACCGGGGGCGAAGGCGCCTGCCTGGACGACTATTGACGCTGAGGCGCGAAAGCGTGGGGAGCAAACAGG
## 491                                                          ACGAAGGGTGCAAGCGTTAATCGGAATTACTGGGCGTAAAGCGTGCGTAGATGGTTCGCTAAGTCGGATGTGAAAGCCCCGGGCTCAACCTGGGAACTGCATCCGATACTGGCGGACTAGAGTGTGATAGAGGATGGCGGAATTCCCGGTGTAGCGGTGAAATGCGTAGAGATCGGGAGGAACATCCGTGGCGAAGGCGGCCATCTGGATCAACACTGACGTTGAGGCACGAAAGCGTGGGGAGCAAACAGG
## 492                                                          ACAGAGGATGCAAGCGTTATCCGGAATCACTGGGCATAAAGCGTCTGTAGGTTGCCTACCAAGTCTGCTGTTAAAGATCAGGGCCTAACCCTGGGAAAGCAGTGGAAACTAGTAGGCTTGAGTGTGGTAGAGGTAGAGGGAATTCCTGGTGTAGCGGTGAAATGCGTAGATATTAGGAAGAACACCAATGGCGAAAGCACTCTACTGGGCCATAACTGACACTGAGAGACGACAGCTAGGGGAGCAAATGGG
## 493                                                          ACGTAGGGTGCAAGCGTTAATCGGAATTACTGGGCGTAAAGCGTGCGCAGGCGGTTATGCAAGACAGAGGTGAAATCCCCGGGCTCAACCTGGGAACTGCCTTTGTGACTGCATAGCTAGAGTACGGCAGAGGGGGATGGAATTCCGCGTGTAGCAGTGAAATGCGTAGATATGCGGAGGAACACCGATGGCGAAGGCAATCCCCTGGGCCTGCACTGACGCTCATGCACGAAAGCGTGGGGAGCAAACAGG
## 494                                                          ACGTAGGGTGCGAGCGTTAATCGGAATTACTGGGCGTAAAGCGTGCGCAGGCGGTTGCGTAAGACAGATGTGAAATCCCCGGGCTCAACCTGGGAACTGCGTTTGTGACTGCGCGACTAGAGTACGGCAGAGGGAGGTGGAATTCCACGTGTAGCAGTGAAATGCGTAGAGATGTGGAGGAACACCGATGGCGAAGGCAGCCTCCTGGGCCAGTACTGACGCTCATGCACGAAAGCGTGGGGAGCAAACAGG
## 495                                                          ACGAGTGCCTCAAGCGTTATCCGGAATCATTGGGCGTAAAGGGTGTGTAGGTGGTCATATTAGTCTTCTGTTAAATTCTTCGGCTTAACCGGGGGCATGCAGGGGAAACGGTATGACTTGAGGATGCGAGGGGTCTGTGGAACTCTAGGTGTAGCGGTGAAATGCGTTGATATCTAGGGGAACACCAAAAGCGAAGGCAGCAGACTGGAGCACTCCTGACACTGAAACACGAAAGCGTGGGTAGCGAATGGG
## 496                                                          ACGTAGGGTGCGAGCGTTAATCGGAATTACTGGGCGTAAAGCGTGCGCAGGCGGTTTCTTAAGCCAGACGTGAAATCCCCGGGCTTAACCTGGGAACTGCGTTTGGAACTGGGAGACTAGAGTGTGTCAGAGGGAGGTGGAATTCCGCGTGTAGCAGTGAAATGCGTAGATATGCGGAGGAACACCGATGGCGAAGGCAGCCTCCTGGGATGACACTGACGCTCATGCACGAAAGCGTGGGGAGCAAACAGG
## 497                                                          ACGGGAGTGGCAAGCGTTATCCGGAATTATTGGGCGTAAAGCGTCCGCAGGCGGTTTTACAAGTCTGTCGTTAAAACGTGGAGCTTAACTCCATTTCAGCGATGGAAACTGTAGAACTAGAGTGTGGTAGGGGCAGAGGGAATTCCCGGTGTAGCGGTGAAATGCGTAGATATCGGGAAGAACACCAGTGGCGAAGGCGCTCTGCTGGGCCATAACTGACGCTCATGGACGAAAGCCAGGGGAGCGAAAGGG
## 498                                                          ACAGAGGGTGCAAGCGTTGTTCGGAATTACTGGGCGTAAAGAGCGCGTAGGCGGTTCTATAAGTCAGACGTGAAAGCCCTGGGCTTAACCCAGGAGGTGCGTTTGAAACTGTAGAGCTAGAGGACGAGAGAGGTGAGTGGAATTCCTAGTGTAGAAGTGAAATTCGTAGATATTAGGAGGAACATCAGTGGCGAAGGCGACTCACTGGCTCGTAACTGACGCTGAGGCGCGAAAGCGTGGGGAGCAAACAGG
## 499                                                          ACAGAGACCTCAAGTGTTATCCGGATTTATTGGGCGTAAAGCGTCCGCAGATTGTTTGACGGGTCAGGGGTTAAAATCCAGTGCTTAACACTGGAATCGCCTTTGAAACTATCAAGCTAGAGGGTGGAAGAGGTAAGCGGAATTGCAGGTGTAGTCGTAATAAGCGTTGATATCTGCAAGAACACCAAAGGCGAAGGCAGCTTACTGGTACACATCTGACGTTCATGGACGAAAGCGTGGGGAGCAAACAGG
## 500                                                          ACGTAGGGTGCAAGCGTTAATCGGAATTACTGGGCGTAAAGCGTGCGCAGGCGGACTTTTAAGCCAGATGTGAAAGCCCCGAGCTTAACTTGGGAATTGCGTTTGGAACTGGGAGTCTAGAGTCTGTCAGAGGGGGATGGAATTCCACGTGTAGCAGTGAAATGCGTAGAGATGTGGAGGAACACCGATGGCGAAGGCAGTCCCCTGGGCCTGCACTGACGCTCATGCACGAAAGCGTGGGGAGCAAACAGG
## 501                                                          ACGGAGGGTGCAAGCGTTGTCCGGAATCATTGGGCGTAAAGAGTTCGTAGGTGGTATGTTAAGTCTGGTGTTAAAGCCCGAAGCTCAACTTCGGTTCGGCACTGGATACTGGCAAACTTGAATGTGGTAGAGGTAAAGGGAATTCCTGGTGTAGCGGTGAAATGCGTAGATATCAGGAGGAACATCGGTGGCGAAAGCGCTTTACTGGGCCATTATTGACACTGAGGAACGAAAGCCGGGGTAGCAAATGGG
## 502                                                          ACGTAGGGTGCAAGCGTTAATCGGAATTACTGGGCGTAAAGCGTGCGCAGGCGGCTTTGCAAGACAGATGTGAAATCCCCGGGCTCAACCTGGGAACTGCATTTGTGACTGCAAGGCTAGAGTACGGTAGAGGGGGATGGAATTCCGCGTGTAGCAGTGAAATGCGTAGATATGCGGAGGAACACCGATGGCGAAGGCAATCCCCTGGACCTGTACTGACGCTCATGCACGAAAGCGTGGGGAGCAAACAGG
## 503                                                          ACGAAGGGGGCTAGCGTTGCTCGGAATGACTGGGCGTAAAGGGCGCGTAGGCGGTTTGATCAGTTAGGTGTGAAATTCCTGGGCTTAACCTGGGGGCTGCACTTAATACGGTCAGGCTAGAGTGTGAAAGAGGGTCGTGGAATTCCCAGTGTAGAGGTGAAATTCGTAGATATTGGGAAGAACACCGGTGGCGAAGGCGGCGACCTGGTTCATTACTGACGCTGAGGCGCGAAAGCGTGGGGAGCAAACAGG
## 504                                                          ACGAAGGGTGCAAGCGTTACTCGGAATTACTGGGCGTAAAGCGTGCGTAGGCGGTTTGTTAAGTCTGCTGTGAAAGCCCTGGGCTCAACCTGGGAACTGCAGTGGATACTGGCGAGCTAGAGTGTGTCAGAGGATGGTGGAATTCCCGGTGTAGCGGTGAAATGCGTAGAGATCGGGAGGAACACCAGTGGCGAAGGCGGCCATCTGGGACAACACTGACGCTGAGGCACGAAAGCGTGGGGAGCAAACAGG
## 505                                                          ACAGAGACCTCAAACGTTGTCCGGATTTATTGGGCGTAAAGGGTCCGCAGGCGGTTGTGTGCGTCACGGGTTAAATTTCAGGGCTCAACCTTGGAACCGCTCGTGATACGACATGACTAGAGACTGGGAGAGGTAAGCGGAATTCACGGTGTAGTAGTAAAATGCGTTAATATCGTGAAGAACACCAAATGCGAAGGCAGCTTACTGGAACAGTTCTGACGCTCAGGGACGAAAGCGTGGGGAGCGAAAGGG
## 506                                                          ACGAAGGGTGCAAGCGTTGCTCGGAATTATTGGGCGTAAAGGGTAGGTAGGTGGTTACGTATGTCGAGAGTGAAATCCCTGGGCTTAACCCAGGAAGTGCTTTGGAAACGGCGTAACTAGAGTTCTGGAGAGGTTCGTGGAATTCCCAGTGTAGCGGTGAAATGCGTAGAGATTGGGAGGAACATCAGAGGCGAAGGCGACGAACTGGACAGATACTGACACTGAACTACGAAAGCGTGGGTAGCAAACAGG
## 508                                                          ACGAAGGTGGCAAGCGTTGTTCGGATTTACTGGGCGTACAGGGAGCGTAGGCGGTTGGGTAAGCCCTCCGTGAAATCTCCGGGCCTAACCCGGAAAGTGCGGAGGGGACTGCTCGGCTAGAGGATGGGAGAGGAGCGCGGAATTCCCGGTGTAGCGGTGAAATGCGTAGAGATCGGGAGGAAGGCCGGTGGCGAAGGCGGCGCTCTGGAACATTTCTGACGCTGAGGCTCGAAAGCGTGGGGAGCAAACAGG
## 510                                                          ACATAGGATGCAAGCGTTATCCGGAGTCACTGGGCGTAAAGGGTTCGCAGCCGGATCTATAAGTCTGTTTTCAAATTCTTTCGCTCAACGAAAGGCAGGGAATAGATACTGTAGATCTAGAGGTTTTTGGAGGGTAATGGAATTCCAGGTGGAGCGGTGAAATGTGTTGATATCTGGAAGAATGCCGAAAGCGAAAGCAGTTACCTACAATTTACCTGACGGTCAGGAACGAAAGCTTGGGGAGCAAATGGG
## 511                                                          ACGGAGGATCCAAGCGTTATCCGGATTTATTGGGTTTAAAGGGTGCGTAGGCGGCGTTGTAAGTCAGTGGTGAAAGTTTGCAGCTTAACTGTAAAATTGCCATTGATACTGCAGTGCTTGAGTACAGATGAGGTGGGCGGAATGTGTCATGTAGCGGTGAAATGCATAGATATGACACAGAACACCGATTGCGAAGGCAGCTCACTAAACTGTAACTGACGCTGAGGCACGAAAGCGTGGGGATCAAACAGG
## 512                                                          ACGTAGGGTGCGAGCGTTAATCGGAATTACTGGGCGTAAAGCGTGCGCAGGCGGTTTGTTAAGACAGTCGTGAAATCCCCGGGCTTAACCTGGGAACTGCGATTGTGACTGGCAAGCTAGAGTACGGCAGAGGGGGGTAGAATTCCTGGTGTAGCAGTGAAATGCGTAGAGATCAGGAGGAATACCGATGGCGAAGGCAGCCCCCTGGGCTAGTACTGACGCTCATGCACGAAAGCGTGGGGAGCAAACAGG
## 513                                                          ACAGAGGGTGCAAGCGTTAATCGGAATTACTGGGCGTAAAGCGCACGCAGGCGGTTGGTTAAGTCAGATGTGAAAGCCCCGGGCTCAACCTGGGAATTGCATTTGAAACTGGCCAACTAGAGTACGTGAGAGGGGGGTAGAATTCCAAGTGTAGCGGTGAAATGCGTAGAGATTTGGAGGAATACCAGTGGCGAAGGCGGCCCCCTGGCACGATACTGACGCTCAGGTGCGAAAGCGTGGGGAGCAAACAGG
## 514                                                          ACAGAGGGTGCAAGCGTTATTCGGAATTACTGGGCATAAAGAGCGCGTAGGCGGTCTAATAAGTCTGATGTGAAATCCCTGGACTCAATTCAGGAAGTGCATCGGATACTGCTAGACTAGAGTGCAGGAGAGGAGAGCGGAATTCCTGGTGTAGAGGTGAAATTCGTAGATATCAGGAGGAACAACAGTGGCGAAGGCGGCTCTCTGGACTGTAACTGACGCTGAGGCGCGAAAGCGTGGGGAGCAAACAGG
## 515                                                          ACGAGTGGTCCAAGCGTTATCCGGAATTATTGGGCGTAAAGAGTGCGTAGGTGGATATACTAGTCGGTTGTTTAACCCACGGGCTCAACCTGTGGATTGCGAGCGAAACGGTATATCTTGAGTATGTTAGGGGTAAGCGGAACTCATAGTGTAGGGGTGAAATCCGTTGATATTATGGGGAACACCAAAAGCGAAGGCAGCTTACTGGAACATTACTGACACTGAGGCACGAAAGCGTGGGTAGCGAATGGG
## 516                                                          ACGTAGGGGGCAAGCGTTATCCGGATTTACTGGGTGTAAAGGGAGCGCAGACGGTTATGCAAGTCTGGAGTGAAACCCCACAGCTCAACTGTGGGCTTGCTTTGGAAACTGTGTAACTAGAGTACTGGAGAGGTAAGCGGAATTCCTAGTGTAGCGGTGAAATGCGTAGATATTAGGAGGAACACCGGTGGCGAAGGCGGCTTACTGGACAGCAACTGACGTTGAGGCTCGAAGGCGTGGGGAGCAAACAGG
## 517                                                          ACAGAGGGTGCGAGCGTTAATCGGAATTACTGGGCGTAAAGGGTGCGTAGGTGGTTATGTAAGTTAGATGTGAAATTTCCGGGCTTAACCTGGGAACTGCGTCTGATACTGCATAGCTAGAGTCCTGTAGAGGAAAGCGGAATTTCCGGTGTAGCAGTGAAATGCGTAGATATCGGAAGGAACATCAGTGGCGAAGGCGGCTTTCTGGACAGAGACTGACACTGAGGCACGAAAGCGTGGGGAGCAAACAGG
## 518                                                          ACGAAGGTGGCGAGCGTTACTCGGATTTACTAGGCGTAAAGCGTGGGCAGGCGGCTCGATAAGTCTCTTGTGAAAGCCTTGGGCTTAACCCAGGGAGGCCAAGAGATACTATCGGGCTTGGATGTGGGAGAGGAGACTGGAATTCCCGGTGTAGCGGTGAAATGCGTTGATATCGGGAGGAACACCAATGGCGAAAGCAAGTCTCTGGACCACCATCGACGCTCATCCACGAAAGCTGGGGGATCAAACAGG
## 519                                                          ACGGGGGATGCAAGTGTTATCCGGATTTACTGGGCGTAAAGCGTCTGCAGGTGGTTTCTTAAGTCTACTGTTAAATCTTGAGGCTCAACCTCAAATCTGCAGTAGAAACTAGGAGACTTGAGTATAGTAGGGGTAGAGGGAATTTCCAGTGGAGCGGTGAAATGCGTAGATATTGGAAAGAACACCGATGGCGAAGGCACTCTACTGGGCTATTACTGACACTCAGAGACGAAAGCTAGGGGAGCAAATGGG
## 520                                                          ACGGAGGGGGCTAGCGTTATTCGGAATTACTGGGCGTAAAGCGCACGTAGGCGGACTGAAAAGTCAGAGGTGAAATCCCAGGGCTCAACCTTGGAACTGCCTTTGAAACTCTCAGTCTTGAGGTCGTGAGAGGTGAGTGGAATTCCGAGTGTAGAGGTGAAATTCGTAGATATTCGGAGGAACACCAGTGGCGAAGGCGGCTCACTGGCACGATACTGACGCTGAGGTGCGAAAGCGTGGGGAGCAAACAGG
## 521                                                          ACGAAGGGTGCGAGCGTTAATCGGAATTACTGGGCGTAAAGCGCGCGTAGGTGGTTTTGTAAGCGGAATGTGAAATCCCCGGGCTCAACCTGGGAACTGCATGCCGAACTGCAAGGCTAGAGTATAGTAGAGGGGTGTGGAATTCCCGGTGTAGCGGTGAAATGCGTAGATATCGGGAGGAACATCAGTGGCGAAGGCGGCACCCTGGACTGATACTGACACTGAGGCGCGAAAGCGTGGGTAGCGAACAGG
## 522                                                          ACATGTGGGGCAAGCGTTGTTCGGAATTACTGGGCATAAAGGGTGCGTAGGCGGCGGTATAAGTCAGCGGTGAAATCCTTCGGCCCAACCGGAGAACTGCCGTTGATACTGTACTGCTTGAGTGCGTGAGGGGTGGCTGGAACGAGTGGTGTAGCGGTGAAATGCGTAGATATCACTCGGAACACCCAAGGCGAAGGCAGGCCACTGGCACGCAACTGACGCTGAGGCACGAAAGCGTGGGGAGCAAACAGG
## 524                                                          ACGTAGGGTGCGAGCGTTAATCGGAATTACTGGGCGTAAAGCGTGCGCAGGCGGTTTGGTAAGTCAGATGTGAAATCCCCGGGCTCAACCTGGGAACTGCATTTGAGACTGCCAAGCTGGAGTTTGGCAGAGGGGGGTGGAATTCCACGTGTAGCAGTGAAATGCGTAGAGATGTGGAGGAACACCGATGGCGAAGGCAGCCCCCTGGGCCAATACTGACGCTCATGCACGAAAGCGTGGGGAGCAAACAGG
## 525                                                          ACGTAGGGTGCGAGCGTTAATCGGAATTACTGGGCGTAAAGCGTGCGCAGGCGGTTTTGTAAGACAGACGTGAAATCCCCGGGCTCAACCTGGGAATGGCGTTTGTGACTGCAAAGCTGGAGTGCGGCAGAGGGGGATGGAATTCCGCGTGTAGCAGTGAAATGCGTAGATATGCGGAGGAACACCGATGGCGAAGGCAATCCCCTGGGCCTGCACTGACGCTCATGCACGAAAGCGTGGGGAGCAAACAGG
## 526                                                          ACGAGGGGTGCAAGCGTTGCTCGGAATTATTGGGCGTAAAGGGTAGGTAGGTGGTCCTGTTTGTCAGAGGTGAAATCCTTGGGCTTAACCCAAGAAGTGCCTCTGAAACGGCAGGACTCGAGTTCTGGAGAGGGTCGTGGAATTCCCGGTGTAGCGGTGAAATGCGTAGAGATCGGGAGGAACACCAGAGGCGAAGGCGGCGACCTGGACAGATACTGACACTCAACTACGAAAGCGTGGGTAGCAAACAGG
## 527                                                          ACGTAGGGTGCAAGCGTTAATCGGAATTACTGGGCGTAAAGCGTGCGCAGGCGGTTATGTAAGACAGAGGTGAAATCCCCGGGCTCAACCTGGGAACTGCCTTTGTGACTGCATAGCTAGAGTACGGTAGAGGGGGATGGAATTCCGCGTGTAGCAGTGAAATGCGTAGATATGCGGAGGAACACCGATGGCGAAGGCAATCCCCTGGGCCTGCACTGACGCTCATGCACGAAAGCGTGGGGAGCAAACAGG
## 528                                                          ACGTAGGGTGCAAGCGTTGTCCGGATTTATTGGGCGTAAAGAGCTCGTAGGCGGCCTGACACGTCGGATGTGAAAACACGGTGCTTAACACCGTGCCTGCATTCGATACGGTCAAGCTAGAGGATGTCAGGGGAAACTGGAATTCCTGGTGTAGCGGTGAAATGCGCAGATATCAGGAGGAACACCGGTGGCGAAGGCGGGTTTCTGGGACATTACTGACGCTGAGGAGCGAAAGCGTGGGGAGCAAACAGG
## 529                                                          ACGGAGGGTGCAAGCGTTACTCGGAATCACTGGGCGTAAAGAGCGTGTAGGCGGGTATATAAGTCAGAAGTGAAATCCAATAGCTTAACTATTGAACTGCTTTTGAAACTGTATACCTAGAATGTGGGAGAGGTAGATGGAATTTCTGGTGTAGGGGTAAAATCCGTAGAGATCAGAAGGAATACCGATTGCGAAGGCGATCTACTGGAACATTATTGACGCTGAGACGCGAAAGCGTGGGGAGCAAACAGG
## 530                                                          ACGGAGGGAGCTAGCGTTGTTCGGAATTACTGGGCGTAAAGCGCACGTAGGCGGCTACTCAAGTCAGAGGTGAAAGCCCAGGGCTCAACCCTGGAACTGCCTTTGAAACTAGGTAGCTAGAATCTTGGAGAGGTCAGTGGAATTCCGAGTGTAGAGGTGAAATTCGTAGATATTCGGAAGAACACCAGTGGCGAAGGCGACTGACTGGACAAGTATTGACGCTGAGGTGCGAAAGCGTGGGGAGCAAACAGG
## 531                                                          ACGAGAGGTGCAAGCGTTATTCGGAATTATTGGGCGTAAAGGGCGCGTAGGCGGCTTTATAAGTTTCTTGTTTAAGACTTCGGCCTAACTGAAGAAAAGCAAGGAATACTGTATAGCTAGAGTATAATAGAGAGAAGTGGAATTTTCGGAGTAGCGGTTAAATGCGTAGATCTCGAAAGGAACGCCAATGGCGAAGGCAGCTTCTTGGGTTATTACTGACGCTGAGGCGCGAAGGCATGGGGAGCAAACAGG
## 532                                                          ACGGAGGGGGCTAGCGTTGTTCGGAATGACTGGGCGTAAAGGGCGCGTAGGCGGTTTTTTAAGTGAGGCGTGAAAGCCCTGGGCTTAACCCAGGAGGTGCGTTTCATACTGGAAGACTTGAGTACGAGAGAGGAAAGCGGAATTCCTAGTGTAGAGGTGAAATTCGTAGATATTAGGAAGAACACCAGTGGCGAAGGCGGCTTTCTGGCTCGTAACTGACGCTGAGGCGCGAAAGCGTGGGGAGCAAACAGG
## 533                                                          ACGAAGGTCCCGAGCGTTGTTCGGAATCACTGGGCGTAAAGGGAGCGTAGGCGGCGTGGTAAGTCAGATGTGAAATCCCGGGGCTCAACCCCGGAACTGCATCCGATACTGCCGTGCTAGAGGATTGGAGAGGTAGCTGGAATTCACGGTGTAGCAGTGAAATGCGTAGATATCGTGAGGAACACTCGTGGCGAAAGCGAGCTACTGGACAACTCCTGACGCTGAGGCTCGAAGGCCAGGGTAGCGAAAGGG
## 534                                                          ACGAAGGGTGCAAGCGTTGCTCGGAATTATTGGGCGTAAAGGGTAGGTAGGTTGTTACGTATGTCTGGGGTGAAATCCCTGAGCTTAACTCAGGAAGTGCCTTGGAAACGGCGTAACTAGAGTGCTAGAGAGGTTCGTGGAATTCCCAGTGTAGCGGTGAAATGCGTAGAGATTGGGAGGAACACCAGAGGCGAAGGCGGCGAACTGGATAGCAACTGACACTGAACTACGAAAGCGTGGGTAGCAAACAGG
## 535                                                          ACGAAGGGGGCTAGCGTTGCTCGGAATTACTGGGCGTAAAGGGCGCGTAGGCGGCTTAGTCAGTCAGGCGTGAAATTCCTGGGCTTAACCTGGGGGCTGCGCTTGATACGGCTAGGCTAGAGGGTAGGAGAGGGTCGCGGAATTCCCAGTGTAGAGGTGAAATTCGTAGATATTGGGAAGAACACCGGTGGCGAAGGCGGCGACCTGGCCTATTACTGACGCTGAGGCGCGACAGCGTGGGGAGCAAACAGG
## 536                                                          ACAGAGGGTGCGAGCGTTGTCCGGAATCACTGGGCGTAAAGGGCGCGTAGGTGGCTTGGTAAGCGTGTGGTGAAAGTCCGGGGCTCAACCCCGGATCTGCCGTGCGAACTGCCGAGCTTGAGCATTGTAGAGGCAGGTGGAATTCCGGGTGTAGCGGTGGAATGCGTAGAGATCCGGAAGAACACCAGTGGCGAAGGCGGCCTGCTGGGCAATTGCTGACACTGAGGCGCGACAGCGTGGGGAGCAAACAGG
## 537                                                          ACAGAGACCTCAAGCATTATCCGGAATTATTGGGCGTAAAGGGTCCGCAGGTGGCTCGGTAAGTCATGCGTCAAATCTTTGGGCTCAACCCAGAGGCTGCGTGTGATACTGCTGAGCTTGAGGCCGGGAGAGGTGCATGGAATTACTGGTGTAGCGGTAAAATGCGTTAAGATCAGTAAGAACACCAAAGGCGAAGGCAATGCACTGGAACGGTCCTGACACTCAGGGACGAAAGCGTGGGGAGCGAAAGGG
## 539                                                          ACGTAGGTCTCGAGCGTTATCCGGAATTACTGGGCGTAAAGCGTCCGCAGCCGGATGTATAAGTCTGTCTTTAAATATCTCGATTCAATCGAGAAAAAGGGATAGATACTGTATGTCTAGAGGACTTTAGAGGTTAGTGGAATTTCCGGTGGAGCGGTGAAATGCGTTGATATCGGAAGGAATGCCAAAAGCGAAAGCAGCTATCTATAAAGTATCTGACGGTCAGGGACGACAGCTTGGGGAGCAAACGGG
## 540                                                          ACAGAGACTGCAAGCGTTATTCGGATTCACTGGGCGTAAAGGGTGCGCAGGCGGTTGCATGTGTTGGGTGTGAAAGCCCGGGGCTCAACCCCGGAATTGCGCCCAAAACTATGCGACTAGAGTACCGGAGAGGATAGCGGAATTCACGGTGTAGCAGTGAAATGCGTAGATATCGTGAAGAACACCAGCGGCGAAGGCGGCTATCTGGAAGGTAACTGACGCTCAGGCACGAAAGCGTGGGGAGCAAAAGGG
## 541                                                          ACGTAGGGTGCGAGCGTTAATCGGAATTACTGGGCGTAAAGCGTGCGCAGGCGGTTTTGTAAGACAGGTGTGAAATCCCCGGGCTTAACCTGGGAACTGCGCTTGTGACTGCAAGACTTGAGTGCGGCAGAGGGGGGTGGAATTCCACGTGTAGCAGTGAAATGCGTAGAGATGTGGAGGAACACCGATGGCGAAGGCAGCCCCCTGGGTCGACACTGACGCTCATGCACGAAAGCGTGGGGAGCAAACAGG
## 542                                                          ACAGGGGATGCAAGTGTTATCCGGAATTATTGGGCGTAAAGCGTCTGCAGGTGGATTTATAAGTCTTTTGTTAAATCTTTGAGCTTAACTCAAAATCGGCAAAAGAAACTATTTATCTTGAGTATGGTAGAGGTAAAGGGAATTTCCAGTGGAGCGGTGAAATGCGTAGATATTGGAAAGAACACCAACAGCGAAGGCACTTTACTGGGCCATTACTGACACTCAGAGACGAAAGCTAGGGGAGCAAACGGG
## 543                                                          ACGGAGGGTGCAAGCGTTATCCGGATTCACTGGGTTTAAAGGGTGCGTAGGCGGGCAGGTAAGTCAGTGGTGAAATCTTCGAGCTTAACTCGGAAACTGCCGTTGATACTATCTGTCTTGAATATAGTGGAGGTAAGCGGAATATGTCATGTAGCGGTGAAATGCTTAGAGATGACATAGAACACCAATTGCGAAGGCAGCTTGCTACACTATTATTGACGCTGAGGCACGAAAGCGTGGGGATCAAACAGG
## 544                                                          ACGTAGGGTGCGAGCGTTAATCGGAATTACTGGGCGTAAAGCGTGCGCAGGCGGTTGTGTAAGCCAGATGTGAAATCCCCGGGCTTAACCTGGGAATGGCATTTGGGACTGCACGGCTGGAGTATGGCAGAGGGGACTGGAATTCCTGGTGTAGCAGTGAAATGCGTAGATATCAGGAGGAATACCGATGGCGAAGGCAGGTCCCTGGGCCAATACTGACGCTCATGCACGAAAGCGTGGGGAGCAAACAGG
## 545                                                          ACAGGGGATGCAAGTGTTATCCGGATTCATTGGGCGTAAAGCGTCTGCAGGTGGATATATAAGTCTGTTGTTAAATCTTTGGGCTTAACCCAAAATCTGCAAAAGAAACTATATATCTTGAGTATGGTAGAGGTAAAGGGAATTTCCGGTGGAGCGGTGAAATGCGTAGATATCGGAAAGAACACCAACTGCGAAGGCACTTTACTGGGCCAGTACTGACACTCAGAGACGAAAGCTAAGGGAGCAAACAGG
## 546                                                          ACGGAGGGGGCTAGCGTTATTCGGAATTACTGGGCGTAAAGCGCACGTAGGCGGACTGAAAAGTCAGAGGTGAAATCCCAGGGCTCAACCTTGGAACTGCCTTTGAAACTCTCAGTCTTGAGGTCGAGAGAGGTGAGTGGAATTCCGAGTGTAGAGGTGAAATTCGTAGATATTCGGAGGAACACCAGTGGCGAAGGCGGCTCACTGGCTCGATACTGACGCTGAGGTGCGAAAGCGTGGGGAGCAAACAGG
## 547                                                          ACGAAGGATCCAAGCGTTGTCCGGATTTACTGGGTTTAAAGGGTGCGTAGGCGGGAGATTAAGTCAGTGGTGAAAGCCCTCAGCTCAACTGAGGAACTGCCATTGAAACTGATTTTCTTGAATATGGTTGAGGTAGATGGAATATAACATGTAGCGGTGAAATGCTTAGATATGTTATAGAACACCGATTGCGAAGGCAGTCTGCTAAGCCATTATTGACGCTGAGGCACGAAAGCGTGGGGAGCAAACAGG
## 548                                                           ACGTAGGTGGCAAGCGTTGTCCGGAATTATTGGGCGTAAAGCGCGCGCAGGCGGGTTTTTAAGTCTGTCTTAAAAGTTCGGGGCTTAACCCCGTGAGGGGATGGAAACTGGAAACCTAGAGTATCGGAGAGGAAAGCGGAATTCCTAGTGTAGCGGTGAAATGCGTAGATATTAGGAAGAACACCAGTGGCGAAGGCGGCTTTCTGGACGAAAACTGACGCTGAGGCGCGAAAGCCAGGGGAGCAAACGGG
## 549                                                         ACGTAGGTGGCGAGCGTTATCCGGATTTATTGGGTTTAAAGGGAGCGTAGGCGGCTGATTAAGTCAGCGGTGAAAGGTAGCAGCTTAACTGTTTTACATGCCGTTGATACTGGTTAGCTTGAGTTGACAGAAGGCAGATAGAATTCCTGGTGTAGCGGTGAAATGCTTAGATACCAGGAGGAATACCGATTGCGAAGGCAGTCTGCTGCAGTCACACTGACGCTGAGGCTCGAAGGTGCGGGGATCAAACAGG
## 550                                                          ACGAAGGTCCCGAGCGTTATTCGGAATCACTGGGCGTAAAGGGAGCGTAGGCGGCGTGGAAAGTCAGATGTGAAATCCCGGGGCTCAACCCCGGAACTGCATCCGATACTACCATGCTAGAGTATTGGAGGGGTAACTGGAATTCTCGGTGTAGCAGTGAAATGCGTGGATATCGAGAGGAACACCTGTGGCGAAAGCGAGTTACTGGACAATTACTGACGCTGAGGCTCGAAGGCTAGGGGAGCAAAAGGG
## 551                                                        ACGTAATCCCTAAGTGGTATCCACGAATATTGGGCCTAAAGCGTTCGTAGCCGGCTGTATAAGTCCTTGGTGAAAGGGGCTTGCTTAACTTGTCCAATGGCTAAGGATACTGTACGGCTGAGAGACCAGTAGAGGTGCATGGAATTACGGGGGTAACGGTGAAATGTTGTGATCCTCGTAGGACCACCGGTAGCGAAGGCGATGCACCAGGATGGTTCTGACGGTGAGGAACGAAAGCGGGGGGAGCGACCCGG
## 552                                                          ACGTAGGTGGCAAGCGTTGTCCGGAATTATTGGGCGTAAAGGGCGTGTAGGTGGATTCTTAAGTCTTGTGTCTAAGTGCGGGGCTCAACCCCGTATGGGCGCAGGAAACTGAGAATCTTGAGTGCAGGAGAGGAAAGTGGAATTCCCAGTGTAGCGGTGAAATGCGTAGATATTGGGAGGAACACCAGTGGCGAAGGCGACTTTCTGGACTGTGTCTGACACTGAGGCGCGAAAGCCAGGGGAGCGAACGGG
## 553                                                          ACGGAGGGTGCGAACGTTATCCGGAATCACTGGGTTTAAAGGGTGCGTAGGCTGTTTAATAAGTCAGTGGTGAAAGGCCGTCGCTTAACGATGGGACTGCCATTGATACTGTTAGACTTGAATCAGGTTGAGGTTGGCGGAATGTGGCATGTAGCGGTGAAATGCATAGATATGCCATGGAATACCGATTGCGAAGGCAGCTGACTGGACCTTGATTGACGCTGAGGCACGAAAGCGTGGGTAGCGAACAGG
## 554                                                          ACGTAGGGTGCAAGCGTTAATCGGAATTACTGGGCGTAAAGCGTGCGCAGGCGGTTTTGTAAGACAGGCGTGAAATCCCCGGGCTTAACCTGGGAATGGCGTTTGTGACTGCAAGACTAGAGTGTGTCAGAGGGGGGTAGAATTCCACGTGTAGCAGTGAAATGCGTAGAGATGTGGAGGAACACCGATGGCGAAGGCAGCCCCCTGGGATAACACTGACGCTCATGCACGAAAGCGTGGGGAGCAAACAGG
## 555                                                         ACGTAGGGGGCGAGCGTTGTCCGGATTTATTGGGCGTAAAGAGCTCGTAGGCGGTTCGGTAAGTCGGGTGTGAAACCTCCAGGCTCAACCTGGAGACGCCACCTGATACTGCTGTGACTAGAGTCCGGTAGGGGAGTGTGGAACTCCTGGTGTAGCGGTGAAATGCGCAGATATCAGGAAGAACACCAGCGGCGAAGGCGGCACTCTGGGCCGGTACTGACGCTGAGGAGCGAAAGCGTGGGTAGCAAACAGG
## 556                                                           ACGAAGGCCCCAAGCGTTATCCGGAATTACTGGGCGTAAAGCGTCTGTAGGTGGTCTGGCAAGTCTCGTGTGAAACTTCAAAGCTCAACTTTGAATTGCACGAGAAACTATCAGACTTGAGTGTGGGAGAGGCAAGCAGAACGGTATGAGTAGGGGTGCAATCCGTTGATACATACCAGAATACCAAAAGCGAAGGCAGCTTGCTGGAACACAACTGACACTGAGAGACGAAAGCGTGGGGAGCGAAAGGG
## 557                                                          ACAGAGGATGCAAGCGTTATCCGGAATGATTGGGCGTAAAGCGTCTGTAGGTGGCTTTCCAAGTCTTTTGTCAAATCCCAGAGCTTAACTTTGGATCGGCACGAGAAACTCGAGAGCTTGAGTACGGTAGGGGCAGAAGGAATTCCCCGTGTAGCGGTGAAATGCGTAGAGATGGGGAAGAACACCGATGGCGAAAGCATTCTGCTGGGCCGTTACTGACACTGAGAGACGAAAGCTAAGGGAGCGAATAGG
## 559                                                          ACGAAGGGTGCTAGCGTTGTTCGGAATCATTGGGCGTAAAGCGTGCGTAGGCGGCTTAGTAAGTCGTTTGTGAAATCCCAAGGCTTAACCTTGGAATTGCAATCGAAACTGCTTTGCTAGAGTATGGTGGGGGATAGTGGAATTCCTAGTGTAGGGGTGAAATCCGTAGAGATTAGGAGGAACATCAGTGGCGAAAGCGACTATCTACGCCAATACTGACGCTGAGGTACGAAAGCGTGGGGAGCAAACAGG
## 560                                                          ACGTAGGGTGCAAGCGTTAATCGGAATTACTGGGCGTAAAGCGTGCGCAGGTGGTTGTGCAAGACAGATGTGAAATCCCCGGGCTTAACCTGGGAACTGCATTTGTGACTGCACAGCTAGAGTGCGGCAGAGGGGGATGGAATTCCGCGTGTAGCAGTGAAATGCGTAGATATGCGGAGGAACACCGATGGCGAAGGCAATCCCCTGGGCCTGCACTGACACTCATGCACGAAAGCGTGGGGAGCAAACAGG
## 561                                                          ACAGAGGGTGCGAGCGTTAATCGGAATGACTGGGCGTAAAGGGTGCGTAGGTGGTTTGTTAAGTTAGCTGTGAAAGCCCCGGGCTTAACCTGGGAATTGCAGCTAATACTGGTGAACTTGAGTAGTTCAGAGGAGTGTGGAATTTCCGGTGTAGCGGTGAAATGCGTAGAGATCGGAAAGAACACCAGTGGCGAAGGCGGCACTCTGGGAACATACTGACACTGAGGCACGAAGGCGTGGGGAGCAAACAGG
## 562                                                          ACGTAGGGTGCGAGCGTTAATCGGAATTACTGGGCGTAAAGCGTGCGCAGGCGGTTATGTAAGACAGATGTGAAATCCCCGGGCTCAACCTGGGACCTGCATTTGTGACTGCATGGCTGGAGTACGGCAGAGGGGGATGGAATTCCGCGTGTAGCAGTGAAATGCGTAGATATGCGGAGGAACACCGATGGCGAAGGCAATCCCCTGGGCCTGTACTGACGCTCATGCACGAAAGCGTGGGGAGCAAACAGG
## 563                                                          ACGAAGGATCCAAGCGTTGTCCGGATTTACTGGGTTTAAAGGGTGCGTAGGCGGACCGATAAGTCAGTGGTGAAAGCCCATAGCTCAACTATGGAACTGCCATTGAAACTGCCGGTCTTGAATTCGGTTGAGGCAGATGGAATATAACATGTAGCGGTGAAATGCTTAGATATGTTATAGAACACCGATTGCGAAGGCAGTCTGCTAAGTCGACATTGACGCTGAGGCACGAAAGCGTGGGGAGCGAACAGG
## 564                                                          ACGTAGGGTGCGAGCGTTAATCGGAATTACTGGGCGTAAAGGGTGCGCAGGCTGGAATACAAGTTAGATGTGAAATACCCGAGCTTAACTTGGGAGGTGCATTTAAGACTGTATACCTAGAGTTAGTCAGAGGATGGGGGAATTCCGAGTGTAGCAGTGAAATGCGCAGATATTCGGAGGAACACCGGTGGCGAAGGCGCCCATCTGGGATATAACTGACGCTCATGCACGAAAGCGTGGGGAGCAAACAGG
## 566                                                          ACGTAGGGTGCGAGCGTTAATCGGAATTACTGGGCGTAAAGCGTGCGCAGGCGGTTTTTTAAGACAGGCGTGAAATCCCCGGGCTTAACCTGGGAACTGCGCTTGTGACTGGAAGGCTAGAGTACGGCAGAGGGGGGTGGAATTCCACGTGTAGCAGTGAAATGCGTAGAGATGTGGAGGAACACCGATGGCGAAGGCAGCCCCCTGGGCCGATACTGACGCTCATGCACGAAAGCGTGGGGAGCAAACAGG
## 567                                                          ACGAAGGGGGCTAGCGTTGTTCGGAATCACTGGGCGTAAAGCGCACGTAGGCGGATCTTTAAGTCAGAGGTGAAATCCCAAGGCTCAACCTTGGAACTGCCTTTGATACTGGGGATCTCGAGTCCGGGAGAGGTGAGTGGAACTGCGAGTGTAGAGGTGAAATTCGTAGATATTCGCAAGAACACCAGTGGCGAAGGCGGCTCACTGGCCCGGTACTGACGCTGAGGTGCGAAAGCGTGGGGAGCAAACAGG
## 569                                                          ACGAGGGGTCCTAGCGTTGTTCGGAATCACTGGGCGTAAAGCGCATGTAGGTGGCTTTTTAAGTCAGGTGTGAAATCCCACGGCTCAACCGTGGAGGTGCACTTGATACTGGGAAGCTTGAGTACGAGAGAGGATGGTAGAATTCCTGGTGTAGTGGTGAAATACGTAGATATCAGGAGGAACACCGGTGGCGAAGGCGGCCATCTGGCTCGATACTGACACTGAGATGCGAAAGCGTGGGGAGCAAACAGG
## 571                                                          ACGAAGGGGGCTAGCGTTGTTCGGAATTACTGGGCGTAAAGCGCACGTAGGCGGGTCGTTAAGTCAGGGGTGAAATCCCGGAGCTCAACTCCGGAACTGCCTTTGATACTGGCGATCTTGAGATCGGAAGAGGTGAGTGGAATTCCCAGTGTAGAGGTGAAATTCGTAGATATTGGGAAGAACACCAGTGGCGAAGGCGGCTCACTGGTCCGATACTGACGCTGAGGTGCGAAAGCGTGGGGAGCAAACAGG
## 572                                                                                                               ACAGACACTGCAAGCATCTTACAACATAACTAGGCAAAGAGCGTTGTTTTGCAAAGTCGAAGCGAAATTATTGCTCAGGAATTTTCAAAGAATCAGTTAAATGAGAGGATATTGAAAGGAACGCCGAAGGTGAAGACAAGAGCATAAATCTTCTAAAGCAACTCAACGAAAGTTCAAGGAGCAAATCGGATTAGATACC
## 574                                                          ACGTAGGGTGCGAGCGTTAATCGGAATTACTGGGCGTAAAGCGTGCGCAGGCGGTTTTGTAAGACAGATGTGAAATCCCCGGGCTTAACCTGGGAACTGCGTTTGTGACTGCAAGGCTAGAGTACGGCAGAGGGGGGTGGAATTCCTGGTGTAGCAGTGAAATGCGTAGAGATCAGGAGGAACACCGATGGCGAAGGCAGCCCCCTGGGCCTGTACTGACGCTCATGCACGAAAGCGTGGGGAGCAAACAGG
## 575                                                          ACGGAGGGTGCGAGCGTTAATCGGAATTACTGGGCGTAAAGCGCGCGTATGTGGTCTGATAAGTCGGATGTGAAAGCCCCGAGCTTAACTTGGGAACGGCATTCGATACTGTCTGACTAGAGTATGGGAGAGGAGAGCGGAATTCCCGGTGTAGCGGTGAAATGCGTAGATATCGGGAGGAACATCAGTGGCGAAGGCGGCTCTCTGGACTAATACTGACACTGAGGCGCGAAAGCGTGGGTAGCAAACAGG
## 576                                                          ACATAGGGTGCAAGCGTTGTCCGGAATTATTGGGCGTAAAGAGCTCGTAGGTCGTTTGTTACGTCGGATGTGAAAACCTGAGGCTCAACCTCAGGCCTGCATTCGATACGGGCAAACTAGAGTTTGGTAGGGGAGACTGGAATTCCTGGTGTAGCGGTGGAATGCGCAGATATCAGGAGGAACACCAATGGCGAAGGCAGGTCTCTGGGCCAATACTGACACTGAGGAGCGAAAGCGTGGGGAGCGAACAGG
## 577                                                          ACGAAGGGTGCTAGCGTTGTTCGGAATCACTGGGCGTAAAGCGCGCGTAGGCGGTTTAACAAGTCAGGGGTGAAAGCCTGGAGCTTAACTCCAGAACTGCCTTTGAAACTGTTAAACTAGAGTGTCGGAGGGGATAGCGGAATTGCTAATGTAGAGGTGAAATTCGTAGATATTAGCAGGAACACCGGTGGCGAAGGCGGCTATCTGGACGACAACTGACGCTGAGGCGCGAAAGCGTGGGGATCAAACAGG
## 578                                                          ACGTAGGGTGCGAGCGTTAATCGGAATTACTGGGCGTAAAGCGTGCGCAGGCGGTTGTGTAAGTCAGATGTGAAATCCCCGGGCTCAACCTGGGAACTGCATTTGAGACTGCACGGCTAGAGTGTGGCAGAGGGGGGTGGAATTCCACGTGTAGCAGTGAAATGCGTAGAGATGTGGAGGAACACCGATGGCGAAGGCAGCCCCCTGGGCCAATACTGACGCTCATGCACGAAAGCGTGGGGAGCAAACAGG
## 579                                                          ACGTAGGGTCCAAGCGTTAATCGGAATTACTGGGCGTAAAGCGTGCGCAGGTGGTTTGTTAAGCACGATGTGAAATCCCCGAGCTCAACTTGGGAATTGCATTGTGAACTGGCTAACTAGAGTACGGCAGAGGGGGGTGGAATTCCACGTGTAGCAGTGAAATGCGTAGAGATGTGGAGGAACACCGATGGCGAAGGCAACCCCCTGGGCTGATACTGACACTCATGCACGAAAGCGTGGGGAGCAAACAGG
## 580                                                          ACGGAGGGTGCAAACGTTGCTCGGAATTATTGGGCGTAAAGCGCATGTAGGCGGTCCGCTAAGTCGGATGTGAAAGCCCTCGGCTTAACCGAGGAAGTGCATCCGAAACTGGCGGGCTAGAGTACGGAAGAGGGTTTCGGAATTCCCGGTGTAGAGGTGAAATTCGTAGATATCGGGAGGAACACCAGTGGCGAAGGCGGAAACCTGGGCCGATACTGACGCTGAGATGCGAAAGCGTGGGGAGCAAACAGG
## 581                                                         ACGAGTGCCTCGAGCGTTATCCGGAATTATTGGGCGTAAAGGGTGTGTAGGTGGTAGTGTTAGTCTTGCGTTAAATCTATTGGCTCAACTGATAGGCTGCGCGGGAAACGGCACAACTGAGAGGATGGTAGGGGTCTGCGGAACTCATAGTGTAGCGGTGAAATGCGTTGATATTATGGGGAACACCAAAAGCGAAGGCAGCAGACTGGACCACTCCTGACACTGAAACACGAAAGCGTGGGTCGCGAATGGG
## 582                                                          ACGTAGGGTGCGAGCGTTAATCGGAATTACTGGGCGTAAAGCGTGCGCAGGCGGTTCTATAAGACAGATGTGAAATCCCCGGGCTTAACCTGGGAACTGCGTTTGTGACTGTAGGACTGGAGTGCGGCAGAGGGGGGTGGAATTCCACGTGTAGCAGTGAAATGCGTAGAGATGTGGAGGAACACCGATGGCGAAGGCAGCCCCCTGGGTCGACACTGACGCTCATGCACGAAAGCGTGGGGAGCAAACAGG
## 583                                                         ACGTAGGGTGCAAGCGTTACCCGGTCTTACTGGGCGTAAAGAGTTCTGTAGGCTGTTGTGTAAGTCAGGGGTTAAATACCTCGGCCCAACCGAGGAAAAGCTTTTGATACTGCATGGCTAGAGGAGGTTAGGGGGCAACGGAACGTATGGTGTAGCAGTGAAATGCGTTGATATCATACGGAACACCAAGGGGGAAGCCAGTTGCCTGGGACCTACCTGACGCTGAGAGACGAAAGCGTGGGGAGCGAAACGG
## 584                                                          ACATAGGTCCCAAGCGTTATCCGGAATTACTGGGCGTAAAGCGTCTGCAGGTGGAAAAGTGTGTGAGATGTGAAAGACCGGGGCTTAACCCCGTGTTTGTGTCTCAAACTGCTTTTCTTGAGTGAGCAAGAGGTATGCGGAATTTATGGAGTAGGAGTGCAATCCGTTGACACCATAAAGAACACCAAAAGCGAAGGCAGCATACTGGGGCTCTACTGACACTCAGAGACGAAAGCGTGGGGAGCGAAAGGG
## 585                                                          ACGGAGGGTGCAAGCGTTATCCGGAATCACTGGGTTTAAAGGGTACGTAGGCGGTTTGATAAGTCAGGTGTGAAATACCGCGGCCTAACTGCGGAACTGCACTTGATACTGTCAGACTTGAGTAGGGTGGAGGTACACGGAATGCATCATGTAGCGGTGAAATGCTTAGATATGATGTAGAACACCGATAGCGAAGGCAGTGTGCCATACCTTAACTGACGCTGAGGTACGAAAGCGTGGGGAGCAAACAGG
## 586                                                          ACGGAGGGTGCAAGCGTTGTTCGGATTTACTGGGCGTAAAGCGTGTCTAGGTGGATTTGCAAGTCAGATGTGAAAGCCCCCGGCTCAACCGGGGAGGGTCATTTGATACTACAGATCTTGAGTTTCGGGGAGGTGAGTGGAATTCCCAGTGTAGAGGTGAAATTCGTAGAGATTGGGAGGAACACCGGAGGCGAAAGCGGCTCACTAACCGAATACTGACACTCAAGCACGAAAGCGTGGGGAGCAAACAGG
## 587                                                          ACGTAGGGTGCAAGCGTTAATCGGAATTACTGGGCGTAAAGCGTGCGCAGGCGGTTATGTAAGACAGAGGTGAAATCCCCGGGCTCAACCTGGGAACTGCGTTCGAAACTGCAAGGCTAGAGTGTGTCAGAGGGGGGTAGAATTCCACGTGTAGCAGTGAAATGCGTAGAGATGTGGAGGAATACCAATGGCGAAGGCAGCCCCCTGGGATAACACTGACGCTCATGCACGAAAGCGTGGGGAGCAAACAGG
## 588                                                          ACGAAGGGTGCTAGCGTTGTTCGGAATGACTGGGCGTAAAGGGCGCGTAGGCGGCCAGATGCGTCTGATGTGAAATCCCTGGGCTTAACCTAGGAACTGCATTGGATACGGTCTGGCTAGAGTCCGCAAGAGGAAGATGGAATTGTGCGTGTAGAGGTGAAATTCGTAGATATGCACAAGAACACCGGTGGCGAAGGCGATCTTCTGGAGCGGTACTGACGCTAAGGCGCGAAAGCGTGGGGAGCAAACAGG
## 589                                                          ACGGGAGTGGCAAGCGTTATCCGGAATTATTGGGCGTAAAGCGTCCGCAGGCGGCCTCGTAAGTCTGTTGTTAAAGCGTGGAGCTTAACTCCATTTCGGCAATGGAAACTGTGAGGCTTGAGTGTGGTAGGGGCAGAGGGAATTCCCGGTGTAGCGGTGAAATGCGTAGATATCGGGAAGAACACCAGTGGCGAAGGCGCTCTGCTGGGCCATAACTGACGCTCATGGACGAAAGCCAGGGGAGCGAAAGGG
## 590                                                          ACGTAGGGTGCGAGCGTTAATCGGAATTACTGGGCGTAAAGCGTGCGCAGGCGGTTTGATAAGACAGGCGTGAAATCCCCGGGCTTAACCTGGGAACTGCGCTTGTGACTGTCTCACTAGAGTACGGCAGAGGGGGGTGGAATTCCACGTGTAGCAGTGAAATGCGTAGAGATGTGGAGGAACACCGATGGCGAAGGCAGCCCCCTGGGCCGATACTGACGCTCATGCACGAAAGCGTGGGGAGCAAACAGG
## 592                                                         ACGTGAGTGGCAAGCGTTATCCGGAATCATTGGGCGTAAAGCGTCCGTAGGGGGGTGTTCAAGTGAAATGTTAAATTCTTCGACCCAATCGAAGGCCTGCGTTTCATACTGTTCACCTAGAAGATGGGAGAGGTAAATAGAATTTCTGGAGTAGGGGTAATATCCGTAGAGACCAGAAGGAATACCAATAGCGAAGGCAATTTACTGGCCCATTCTTGACCCTCAAGTGACGAAAGCGTGGGGAGCAAACAGG
## 594                                                          ACGTAGGGTGCAAGCGTTGTCCGGATTTATTGGGCGTAAAGAGCTCGTAGGCGGTTAGACACGTCGGATGTGAAAACTGGTGGCTCAACCACCAGCCTGCATTCGATACGGTCTGACTTGAATGTGGTAGGGGTAACTGGAATTCCTGGTGTAGCGGTGAAATGCGCAGATATCAGGAGGAACACCGATGGCGAAGGCAGGTTACTGGGCCAATATTGACGCTGAGGAGCGAAAGCGTGGGGAGCAAACAGG
## 595                                                          ACGTAGGGTGCAAGCGTTAATCGGAATTACTGGGCGTAAAGCGTGCGCAGGCGGTTTTGTAAGACAGATGTGAAATCCTCGGGCTCAACCTGAGAACTGCATTTGTGACTGCAAAGCTAGAGTGCGGCAGAGGGGGATGGAATTCCGCGTGTAGCAGTGAAATGCGTAGATATGCGGAGGAACACCGATGGCGAAGGCAATCCCCTGGGCCTGCACTGACGCTCATGCACGAAAGCGTGGGGAGCAAACAGG
## 596                                                          ACGGAGGGTGCAAGCGTTATCCGGATTTATTGGGTTTAAAGGGTCCGTAGGCGGATGATTAAGTCAGTGGTGAAAGCCCACAGCTCAACTGTGGAACTGCCATTGATACTGGTTATCTTGAGTGCGGTTGAAGTTGGCGGAATATGTCATGTAGCGGTGAAATGCTTAGATATGACATAGAACACCTATTGCGAAGGCAGCCAACTAAGCCGTAACTGACGTTGAGGGACGAAAGCGTGGGGAGCGAACAGG
## 597                                                          ACGTAGGTGGCGAGCGTTGTCCGGATTTATTGGGTTTAAAGGGTGCGTAGGCGGCTCATTAAGTCAGTTGTGAAATACGACAGCTTAACTGTCGAGGTGCAATTGATACTGGTGAGCTTGAGTACTGATGAGGTAGGCGGAATGGACGGTGTAGCGGTGAAATGCTTAGAGATCGTCCAGAACACCGATAGCGAAGGCAGCTTACTAAGGAGTAACTGACGCTGAGGCACGAAAGTGTGGGGATCAAACAGG
## 598                                                          ACAGAGGGTGCGAGCGTTAATCGGAATTACTGGGCGTAAAGGGCGCGTAGGTGGTTTATTAAGATTGTTGTGAAATCCCTGGGCTTAACCTAGGAACTGCAACGATGACTGATAAGCTTGAGTATTGTAGAGGGTAGTGGAATTTCCGGTGTAGCGGTGAAATGCGTAGAGATCGGAAGGAACACCAGTGGCGAAGGCGGCTACCTGGACAAATACTGACACTGAGGCGCGAGAGCGTGGGGAGCAAACAGG
## 599                                                          ACGAACCGTGCGAACGTTGTTCGGAATCATTGGGCTTAAAGGGCGCGTAGGCGGGTTGACAAGTCCGGGGTGAAAGCCTCCAGCTCAACTGGAGAAGAGCCCCGGATACTGTCAGTCTGGAGAGGGATAGGGGCACATGGAACTTCCGGTGGAGCGGTGAAATGCGTAGAGATCGGAAGGAACGCCGGTGGCGAAAGCGATGTGCTGGATCTTTTCTGACGCTGAGGCGCGAAAGCTAGGGGAGCAAACGGG
## 600                                                          ACGGAGGGAGCTAGCGTTGTTCGGAATTACTGGGCGTAAAGCGCACGTAGGCGGCTACTCAAGTCAGAGGTGAAAGCCCGGGGCTCAACCCCGGAACTGCCTTTGAAACTAGGTAGCTAGAATCTTGGAGAGGTCAGTGGAATTCCGAGTGTAGAGGTGAAATTCGTAGATATTCGGAAGAACACCAGTGGCGAAGGCGACTGACTGGACAAGTATTGACGCTGAGGTGCGAAAGCGTGGGGAGCAAACAGG
## 601                                                          ACGTAGGGTGCAAGCGTTAATCGGAATTACTGGGCGTAAAGCGTGCGCAGGCGGTTATGTAAGACAGAGGTGAAATCCCCGGGCTCAACCTGGGAACTGCCTTTGTGACTGCATAGCTAGAGTACGGCAGAGGGGGATGGAATTCCGCGTGTAGCAGTGAAATGCGTAGATATGCGGAGGAACACCGATGGCGAAGGCAATCCCCTGGACCTGTACTGACGCTCATGCACGAAAGCGTGGGGAGCAAACAGG
## 602                                                          ACAGAGACTGCAAGCGTTATTCGGATTCACTGGGCGTAAAGGGTGCGCAGGCGGCCAAGTGTGTGAGGTGTGAAAGCCCGGAGCTTAACTCCGGAATTGCACCTCAAACTACTTGGCTAGAGCATTGGAGAGGGTAGCAGAATTCATGGTGTAGCAGTGAAATGCGTAGATATCATGAGGAATACCAGAGGCGAAGGCGGCTACCTGGACAATTGCTGACGCTCAGGCACGAAAGCGTGGGGAGCAAAAGGG
## 603                                                          ACGAAGGGTGCTAGCGTTGTTCGGAATCATTGGGCGTAAAGCGTATGTAGGCTGTTTGGTAAGTCAGTTGTGAAAGCCCCGAGCTTAACTTGGGAATTGCAGCTGAAACTGCCTTGCTTGAATTTGGTGGGGGATAGTGGAATTCTTAGTGTAGGGGTGAAATCCGTAGATATTAAGAGGAACACCGGTGGCGAAGGCGACTATCTACACCAATATTGACGCTGAGATACGAAAGCGTGGGGAGCAAACAGG
## 604                                                          ACGTAGGGTGCAAGCGTTAATCGGAATTACTGGGCGTAAAGCGTGCGCAGGCGGTTTGATAAGCCAGATGTGAAATCCCCGAGCTCAACTTGGGAACTGCGTTTGGAACTGTCAGACTAGAGTGCGTCAGAGGGGGGTGGAATTCCGCGTGTAGCAGTGAAATGCGTAGAGATGCGGAGGAACACCGATGGCGAAGGCAGCCCCCTGGGATGACACTGACGCTCATGCACGAAAGCGTGGGGAGCAAACAGG
## 605                                                          ACGTAGGGGGCAAGCGTTGTCCGGAATTACTGGGCGTAAAGCGCACGCAGGCGGATTAATAAGTCAGTTGTAAAATGCACGGGCTCAACTCGTGTTTGTCGACTGATACTGTTAGTCTAGAGTATGTGAGAGGGAAGTGGAATTCCCGGTGTAGCGGTGAAATGCGTAGATATCGGGAGGAACACCAGTGGCGAAGGCGGCTTCCTGGCACAAAACTGACGCTCATGTGCGAAAGCTAGGGCAGCGAACGGG
## 606                                                          ACGTAGGGTGCGAGCGTTAATCGGAATTACTGGGCGTAAAGCGTGCGCAGGCGGTTGTGCAAGACAGATGTGAAATCCCCGGGCTCAACCTGGGAACTGCATTTGTGACTGCACGACTAGAGTACGGCAGAGGGGGGTAGAATTCCACGTGTAGCAGTGAAATGCGTAGAGATGTGGAGGAATACCGATGGCGAAGGCAGCCCCCTGGGCCGATACTGACGCTCATGCACGAAAGCGTGGGGAGCAAACAGG
## 607                                                          ACGAAGGGGGCTAGCGTTGTTCGGAATCACTGGGCGTAAAGCGCACGTAGGCGGATCTTTAAGTCAGGGGTGAAATCCCGGGGCTCAACCCCGGAACTGCCCTTGATACTGGAGATCTCGAGTCCGGGAGAGGTGAGTGGAACTGCGAGTGTAGAGGTGAAATTCGTAGATATTCGCAAGAACACCAGTGGCGAAGGCGGCTCACTGGCCCGGAACTGACGCTGAGGTGCGAAAGCGTGGGGAGCAAACAGG
## 608                                                                                                               ACAGACACTGCAAGCATCTTACAACATAACTAGGCAAAGAGCGTTGTTTTGCAAAGTCGAAGCGAAATTATTGCTCAGGAATTTTCAAAGAATCAGTTAAATGAGAGGATATTGAAAGGAACGCCGAAGGTGAAGACAAGAGCATAAATCTTCTAAAGCAACTCAACGAAAGTTCAAGGAGCAAATCGGATTAGAAACC
## 609                                                          ACGTAGGGTGCGAGCGTTAATCGGAATTACTGGGCGTAAAGCGTGCGCAGGCGGCGACACAAGACAGATGTGAAATCCCCGGGCTCAACCTGGGAACTGCGTTTGTGACTGTGTTGCTAGAGTGCGGCAGAGGGGGGTGGAATTCCACGTGTAGCAGTGAAATGCGTAGAGATGTGGAGGAACACCGATGGCGAAGGCAGCCCCCTGGGTCGACACTGACGCTCATGCACGAAAGCGTGGGGAGCAAACAGG
## 610                                                          ACGGAGGGTGCTAGCGTTGTTCGGAATTACTGGGCGTAAAGCGTACGTAGGCGGTTTGATAAGTTGGAAGTGAAATCCCAAGGCTTAACCTTGGAATTGCTTTCAAAACTGTCAGACTAGAGAATGGTAGGGGAAAGTGGAATTCCTAGTGTAGAGGTGAAATTCGTAGATATTAGGAGGAACACCAGAGGCGCAAGCGGCTTTCTGGACCATTTCTGACGCTAAGGTACGAAAGCGTGGGGAGCGAACAGG
## 613                                                          ACGAGTGGCCCAAGCGTTATCCGGAATTATTGGGCGTAAAGGATGTGTAGGTGGTTGTATTAGTCGAATGTCTAACCTCTGAGCTCAACTTGGAAACAGCATTCGAAACGGTACGACTAGAGTATGTTAGGGGTAAGCGGAACTAATAGTGTAGGGGTGAAATCCGTTGATATTATTGGGAACACCAAAAGCGAAGGCAGCTTACTGGAACATTACTGACACTGAAACATGAAAGCGTGGGTAGCGAATGGG
## 614                                                          ACGTAGGATCCAAGCGTTGTCCGGATTTACTGGGTATAAAGGGTGCGTAGGCGGCTTTTTGCGTCAGAGGTGAAATCCACGGGCTTAACCCGTGGGGTGCCTTTGAAACGGGAAGGCTTGAGTATGACAGAGGCGGATGGAATTCCTGGTGTAGCAGTGAAATGCGTAGATATCAGGAGGAACACCGGTGGCGAAGGCGGTCCGCTGGGTCATAACTGACGCTGAGGCACGAAAGCGTGGGGATCAAACAGG
## 615                                                          ACAGAGGTGGCAAGCGTTGTTCGGATTTATTGGGTGTAAAGGGCAGGTAGGCTGCTAAGTAAGTCTGTTGTGAAATCCCGGGGCTCAACCCCGGAACTGCAACGGAAACTACTTGGCTGGAGTATTGTAGGGGTGAGGGGAATTCTCGGTGTAAGGGTGAAATCTGTAGATATCGAGAGGAACACCAATGGCGAAGGCACCTCACTGGACAATTACTGACGCTGAGCTGCGAAAGCTGGGGGAGCAAACAGG
## 616                                                          ACGTAGGGTGCAAGCGTTAATCGGAATTACTGGGCGTAAAGCGTGCGCAGGCGGTTTGATAAGACAGAGGTGAAATCCCCGGGCTCAACCTGGGAACTGCCTTTGTGACTGTCAGGCTAGAGTGTGTCAGAGGGGGGTAGAATTCCACGTGTAGCAGTGAAATGCGTAGAGATGTGGAGGAATACCGATGGCGAAGGCAGCCCCCTGGGATAACACTGACGCTCATGCACGAAAGCGTGGGGAGCAAACAGG
## 617                                                          ACGGAGGGTGCAAGCGTTAATCGGAATAACTGGGCGTAAAGCGCACGCAGGCGGTCGGATAAGTCAGATGTGAAAGCCCCGGGCTCAACCTGGGAACTGCATTTGAAACTGTCTGACTAGAGTCTTGTAGAGGGGGGTAGAATTCCAGGTGTAGCGGTGAAATGCGTAGAGATCTGGAGGAATACCGGTGGCGAAGGCGGCCCCCTGGACAAAGACTGACGCTCAGGTGCGAAAGCGTGGGGAGCAAACAGG
## 618                                                          ACGGAGGGTGCAAGCATTGATCGGAATTACTGGGCGTAAAGGGCGCGTAGGCGGCTAGGAAAGTCAGATGTGAAATTCCGGGGCTCAACCCCGGAGCTGCATTTGAAACTTCCTGACTAGAGGTCAGTTAGGGAAAACGGAATTCCACGTGTAGCGGTGAAATGCGTAGATATGTGGAAGAACACCGGTGGTGAAGACGGTTTTCTGGGCTGATACTGACGCTGAGGCGCGAAAGCAAGGGGAGCAAACAGG
## 620                                                          ACAGAGGTGGCAAGCGTTGTTCGGATTCATTGGGTGTAAAGGGCAGGTAGGCTGTTTTGCAAGTTTGGCGTGAAATCCCAGGGCTCAACCCTGGAACTGCGCTGAAAACTGCATGACTTGAGTACTGTAGAGGTGAGGGGAATTCTCGGTGTAAGGGTGAAATCTGTAGATATCGAGAGGAACACCAGTGGCGAAGGCGCCTCACTGGACAGTTACTGACGCTGAGCTGCGAAAGCAAGGGGAGCAAACAGG
## 621                                                          ACATAGGGTGCAAGCGTTGTCCGGAATTATTGGGCGTAAAGAGCTCGTAGGTGGTTCGTCACGTCGGATGTGAAACTCTGGGGCTTAACCCCAGACCTGCATTCGATACGGGCGAGCTTGAGTATGGTAGGGGAGTCTGGAATTCCTGGTGTAGCGGTGGAATGCGCAGATATCAGGAGGAACACCAATGGCGAAGGCAGGTCTCTGGGCCAATACTGACACTGAGGAGCGAAAGTCTGGGGAGCGAACAGG
## 622                                                          ACGAGTGGTCCAAGCGTTATCCGGAATTATTGGGCGTAAAGAGTGCGTAGGTGGATTTATTAGTCAGTTGTTTAACCCATGGGCTCAACCTGTGGATTGCAATTGAAACGGTAAATCTTGAGTATGTTAGGGGTGTGCGGAACTCATAGTGTAGGGGTGAAATCCGTTGATATTATGGGGAACACCAAAAGCGAAGGCAGCACACTGGAACATTACTGACACTGAGGCACGAAAGCGTGGGTAGCGAATGGG
## 623                                                          ACGGAGGATCCAAGCGTTATCCGGATTTATTGGGTTTAAAGGGTGCGTAGGCGGCGTTGTAAGTCAGTGGTGAAAGTTTTCAGCTTAACTGGAAAATTGCCATTGATACTGCAGTGCTTGAGTATAGATGAGGTGGGCGGAATGTGTCATGTAGCGGTGAAATGCATAGATATGACACAGAACACCGATTGCGAAGGCAGCTCACTAAACTATAACTGACGCTGAGGCACGAAAGCGTGGGGATCAAACAGG
## 624                                                          ACGAAGGATCCAAGCGTTGTCCGGATTTACTGGGTTTAAAGGGTGCGTAGGCGGGGAATTAAGTCAGTGGTGAAAGCTGGTAGCTCAACTATCAAATTGCCATTGAAACTGATTCTCTCGAGTACGGTTGAGGTAGCTGGAATGTAACATGTAGCGGTGAAATGCTTAGATATGTTACAGAACACCGATTGCGAAGGCAGGCTACTAAACCGTAACTGACGCTGAGGCACGAAAGCGTGGGGATCAAACAGG
## 625                                                          ACGTAGGGTGCAAGCGTTAATCGGAATTACTGGGCGTAAAGCGTGCGCAGGCGGTTATGTAAGACAGTTGTGAAATCCCCGGGCTCAACCTGGGAACTGCATCTGTGACTGCATAGCTAGAGTACGGCAGAGGGGGATGGAATTCCGCGTGTAGCAGTGAAATGCGTAGATATGCGGAGGAACACCGATGGCGAAGGCAATCCCCTGGGCCTGTACTGACGCTCATGCACGAAAGCGTGGGGAGCAAACAGG
## 626                                                          ACGTAGGGTGCGAGCGTTAATCGGAATTATTGGGCGTAAAGGGTGAGTAGGCGGTTTAGTAAGTTAATAGTGAAAGCATTGGGCTTAACCTGATAAGTGCTATTGATACTGCTAGACTAGAGTTAGTTAGAGGATTGTGGAATTCCAAGTGTAGTAGTGAAATGCGTAGATATTTGGAGGAACCTTAATGGCGAAGGCAGCAATCTGGGATGAGACTGACGCTGAGTCACGAAAGCGTGGGGAGCAAACAGG
## 627                                                          ACGAAGGTGGCGAGCGTTACTCGGAATTACTAGGCGTAAAGCGTGGGTAGGTGGTTCGGCAAGTCTGTTGTGGAAGCTCCTGGCTCAACCGGGAGAGGCCAATGGATACTACCGAGCTTGAATGTGGGAGAGGTCACTGGAATTCCTGGTGTAGCGGTGAAATGCGTAGAGATCAGGAGGAACACCGATGGCGAAAGCAGGTGACTGGACCACTATTGACACTGAGCCACGAAAGCTAGGGGAGCAAACAGG
## 628                                                          ACAGGGGATGCAAGTGTTATCCGGAATTATTGGGCGTAAAGCGTCTGCAGGTTGATATTTAAGTCTTTTGTTAAATTTCTAGGCTTAACCTGGAAGTCGCAAAAGAAACTATTTATCTTGAGTATGGTAGAGGTAAAGGGAATTTCAAGTGGAGCGGTGAAATGCGTAGATATTTGAAAGAACACCAACAGCGAAGGCACTTTACTGGGCCATTACTGACACTCAGAGACGAAAGCTAGGGGAGCAAACAGG
## 629                                                          ACGTAGGTGGCGAGCGTTGTCCGGATTTACTGGGCGTAAAGGGAGCGTAGGCGGACTTTTAAGTGAGATGTGAAATACCCGGGCTCAACTTGGGTGCTGCATTTCAAACTGGAAGTCTAGAGTGCAGGAGAGGAGAATGGAATTCCTAGTGTAGCGGTGAAATGCGTAGAGATTAGGAAGAACACCAGTGGCGAAGGCGATTCTCTGGACTGTAACTGACGCTGAGGCTCGAAAGCGTGGGGAGCAAACAGG
## 630                                                          ACGAAGGTGGCAAGCGTTACTCGGAATTACTAGGCGTAAAGGGCAGGTAGGTGGTTTGATAAGTCTGTTGTGAAAGCTCCTGGCTTAACTGGGAGAGGTCAACAGAAACTATCAGGCTTGAGTATGGGAGAGGGTACTGGAATTCCCGGTGTAGCGGTGAAATGCGCAGAGATCGGGAGGAACACCTATGGCGAAAGCAGGTACCTGGACCATTACTGACACTCAGCTGCGAAAGCTAGGGGAGCAAACAGG
## 631                                                         ACGAGTGCCTCGAGCGTTATCCGGAATTATTGGGCGTAAAGGGTGTGTAGGCGGTAGTGTTAGTCTTGTGTTAAATCTATTGGCTCAACCGATAGGCTGCATGGGAAACGGCACAACTAAGAGGATGGAAGGGGTCTGCGGAACTCATGGTGTAGCGGTGAAATGCGTTGATATCATGGGGAACACCGAAAGCGAAGGCAGCAGACTGGTCCACTCCTGACGCTGAAACACGAAAGCGTGGGTCGCGAATGGG
## 632                                                          ACGGAGGATCCGAGCGTTATCCGGATTTATTGGGTTTAAAGGGAGCGTAGGTGGATTGTTAAGTCAGTTGTGAAAGTTTGCGGCTCAACCGTAAAATTGCAGTTGAAACTGGCAGTCTTGAGTACAGTAGAGGTGGGCGGAATTCGTGGTGTAGCGGTGAAATGCTTAGATATCACGAAGAACTCCGATTGCGAAGGCAGCTCACTAGACTGTCACTGACACTGATGCTCGAAAGTGTGGGTATCAAACAGG
## 633                                                          ACAGAGACCTCGATCGTTGTCCGGATTCATTGGGCGTAAAGGGTCCGTAGGCGGTGGTGCAAGTCATTTGTCAAATCTCAGGGCTTAACTCTGAGGCTGCGAGTGAAACTGCATTACTAGAGGCCGGGAGAGGCAAGCGGAATTACCGGTGTAGTCGTAATAAGCGTTGATATCGGTAAGAACACCAAAGGCGAAGGCAGCTTGCTGGAACGGTTCTGACGCTCAGGGACGAAAGCGTGGGTAGCGAATGGG
## 634                                                          ACGAAGGTGGCGAGCGTTACTCGGAATTACTAGGCGTAAAGCGTGGGCAGGCGGCTCGATAAGTCTTTTGTGAAAGCCCCGGGCTTAACCCGGGGAGGTCAAAGGATACTGTCGGGCTTGGATGTGGGAGAGGTCACTGGAATTCCTGGTGTAGCGGTGAAATGCGTAGATATCAGGAGGAACACCAATGGCGAAGGCAGGTGACTGGACCACTATCGACGCTCATCCACGAAAGCTGGGGGATCAAACAGG
## 635                                                          ACGGAGGGTGCAAGCGTTGTTCGGAATCACTGGGCGTAAAGGGCGCGTAGGCGGTTTGATAAGTCAGATGTGAAAGCCCACGGCTTAACCGTGGAAGTGCATTTGAAACTGTCAGACTTGAGTACCAGAGGGGAAAGTGGAATTCCCGGTGTAGAGGTGAAATTCGTAGATATCGGGAGGAATACCGGTGGCGAAGGCGACTTTCTGGCTGGATACTGACGCTGAGGCGCGAAAGCGTGGGGAGCAAACAGG
## 636                                                          ACGGAGGGTGCGAGCGTTAATCGGAATTACTGGGCGTAAAGCGCGCGTAGGCGGTTTGTTAAGTCAGCTGTGAAAGCCCCGGGCTCAACCTGGGAACTGCAGTTGATACTGGCCGACTAGAGTACGAGAGAGGGAGGTAGAATTCCATGTGTAGCGGTGAAATGCGTAGATATATGGAGGAATACCGGTGGCGAAGGCGGCCTCCTGGCTCGATACTGACGCTGAGGTGCGAAAGCGTGGGGATCAAACAGG
## 637                                                          ACGTAGGGTGCAAGCGTTAATCGGAATTACTGGGCGTAAAGCGTGCGCAGGCGGACTTTTAAGCCAGATGTGAAAGCCCCGAGCTTAACTTGGGAATTGCGTTTGGAACTGAGCATCTAGAGTCTGTCAGAGGGGGATGGAATTCCACGTGTAGCAGTGAAATGCGTAGAGATGTGGAGGAACACCGATGGCGAAGGCAGTCCCCTGGGATAAGACTGACGCTCATGCACGAAAGCGTGGGGAGCAAACAGG
## 639                                                          ACGTAGGGTGCGAGCGTTAATCGGAATTACTGGGCGTAAAGCGTGCGCAGGCGGTTATGTAAGACAGATGTGAAATCCCCGGGCTCAACCTGGGAACTGCGTTTGTGACTGCATAACTAGAGTACGGCAGAGGGAGGTGGAATTCCGCGTGTAGCAGTGAAATGCGTAGATATGCGGAGGAACACCGATGGCGAAGGCAATCCCCTGGGCCTGCACTGACGCTCATGCACGAAAGCGTGGGGAGCAAACAGG
## 640                                                          ACGAAGGATGCAAGCGTTATCCGGATTCATTGGGTTTAAAGGGTGCGTAGGCGGACTTGTAAGTCAGTGGTGAAATCTCTTCGCTTAACGAAGAAACTGCCATTGATACTGCAGGTCTAGAGTATAGATGACGTTGGCGGAATATGACATGTAGTGGTGAAATACTTAGATATGTCATAGAACACCGATTGCGAAGGCAGCTAACGAAACTATAACTGACGCTGAGGCACGAAAGTGCGGGGATCAAACAGG
## 641                                                          ACAGAGACCTCAAGCGTTATCCGGATTTATTGGGCGTAAAGGGTCCGCAGGTGGCTTTGTGCGTCGATGGTTAAATTTTCGGGCTCAACTCGAAAACTGCTATCGATACGGCATGGCTAGAGGCCGGAAGAGGTAAGCGGAATTGCCGGTGTAGTAGTAATATGCGTTAATATCGGCAAGAACACCAAATGCGAAGGCAGCTTACTGGGACGCGCCTGACACTCAGGGACGAAAGCGTGGGGAGCGAAAGGG
## 642                                                          ACGGAAGGTGCGAGCGTTAATCGGAATTACTGGGCGTAAAGCGCACGTAGGTGGCTTGGTAAGTCGGATGTGAAAGCCCCGGGCTCAACCTGGGAACGGCATTCGATACTGCTGAGCTAGAGTACGAAAGAGGGGGGTGGAATTCCAGGTGTAGCGGTGAAATGCGTAGAGATCTGGAGGAACACCGGTGGCGAAGGCGGCCCCCTGGTTCGATACTGACGCTGAGGTGCGAAAGCGTGGGGAGCAAACAGG
## 643                                                          ACGAGGGGAGCTAGCGTTGTTCGGAATTACTGGGCGTAAAGGGCGTGTAGGCGGTACCATAAGTCAGATGTGAAAGCCCTGGGCTTAACCTAGGATGTGCATTTGATACTGTGGAACTAGAGATCGAGAGAGGAAAGTGGAATTACGAGTGTAGAGGTGAAATTCGTAGATATTCGTAAGAACACCAGTGGCGAAGGCGACTTTCTGGCTCGATACTGACGCTAAGGCGCGAAAGCGTGGGGAGCAAACAGG
## 644                                                          ACGTAGGGTGCAAGCGTTGTCCGGAATTATTGGGCGTAAAGAGCTCGTAGGCGGTTTGTCACGTCTGCTGTGAAAATCCAAGGCTCAACCTTGGACTTGCAGTGGGTACGGGCAGGCTAGAGTGCGGTAGGGGAGATGGGAATTCCTGGTGTAGCGGTGGAATGCGCAGATATCAGGAGGAACACCAATGGCGAAGGCACATCTCTGGGCCGTAACTGACGCTGAGGAGCGAAAGCGTGGGGAGCGAACAGG
## 646                                                          ACGGAGGGTGCAAGCGTTATCCGGAATCATTGGGTTTAAAGGGTCCGCAGGCGGGAATGTAAGTCAGAGGTGAAATCCTACAGCTCAACTGTAGAACTGCCTTTGATACTGCGTTTCTTGAATTCGATCGAAGTGGGCGGAATATGACATGTAGCGGTGAAATGCATAGATATGTCATAGAACACCGATAGCGAAGGCAGCTCACTAGGTCTGAATTGACGCTCATGGACGAAAGCGTGGGGAGCAAACAGG
## 647                                                          ACGAAGGGGGCTAGCGTTGTTCGGAATTACTGGGCGTAAAGCGCACGTAGGCGGGTTTGTAAGTAGGGGGTGAAATCCCAGGGCTCAACCCTGGAACTGCCTTCTAAACTGCAAGCCTGGAGGTCAGGAGAGGCGAGTGGAATACCGAGTGTAGAGGTGAAATTCGTAGATATTCGGTGGAACACCAGTGGCGAAGGCGACTCGCTGGACTGATACTGACGCTGAGGTGCGAAAGCGTGGGGAGCAAACAGG
## 648                                                          ACGTAGGGTGCAAGCGTTGTCCGGAATTACTGGGTGTAAAGGGAGCGCAGGCGGACCGGCAAGTTGGAAGTGAAAACTATGGGCTCAACCCATAAATTGCTTTCAAAACTGCTGGCCTTGAGTAGTGCAGAGGTAGGTGGAATTCCCGGTGTAGCGGTGGAATGCGTAGATATCGGGAGGAACACCAGTGGCGAAGGCGACCTACTGGGCACCAACTGACGCTGAGGCTCGAAAGCATGGGTAGCAAACAGG
## 649                                                          ACGTAGGGTGCGAGCGTTAATCGGAATTACTGGGCGTAAAGCGTGCGCAGGCGGTTGTGTAAGACAGGCGTGAAATCCCCGGGCTCAACCTGGGAACTGCGCTTGTGACTGCACAGCTAGAGTACGGCAGAGGGGGGTGGAATTCCACGTGTAGCAGTGAAATGCGTAGAGATGTGGAGGAACACCGATGGCGAAGGCAGCCCCCTGGGCCGATACTGACGCTCATGCACGAAAGCGTGGGTAGCAAACAGG
## 650                                                          ACGGAGGATTCGAGCGTTGTCCGGATTTATTGGGTTTAAAGGGTGCGTAGGCGGATTTTTAAGTCAGTGGTGAAAGCCTGCAGCTCAACTGTAGAATTGCCATTGAAACTGAAAATCTTGAATTTGGTTAAAGTAGGCGGAATGTATCATGTAGCGGTGAAATGCTTAGATATGATACAGAACACCGATAGCGAAGGCAGCTTGCTGAACCAATATTGACGCTGAGGCACGAAAGCGTGGGGAGCAAACAGG
## 651                                                          ACGAAGGGGGCTAGCGTTGTTCGGAATCACTGGGCGTAAAGCGCACGTAGGCGGATCTTTAAGTCAGAGGTGAAAGCCTGGAGCTCAACTCCAGAACTGCCTTTGATACTGGGGATCTCGAGTCCGGGAGAGGTGAGTGGAACTGCGAGTGTAGAGGTGAAATTCGTAGATATTCGCAAGAACACCAGTGGCGAAGGCGGCTCACTGGCCCGGTACTGACGCTGAGGTGCGAAAGCGTGGGGAGCAAACAGG
## 652                                                          ACGTAGGGTGCGAGCGTTAATCGGAATTACTGGGCGTAAAGCGTGCGCAGGCGGTTTCGTAAGACAGACGTGAAATCCCCGGGCTCAACCTGGGAACTGCGTTTGTGACTGCGAGGCTAGAGTATGGCAGAGGGGGGTGGAATTCCACGTGTAGCAGTGAAATGCGTAGATATGCGGAGGAACACCGATGGCGAAGGCAATCCCCTGGGCCTGTACTGACGCTCATGCACGAAAGCGTGGGGAGCAAACAGG
## 653                                                          ACGTAGGGTGCGAGCGTTAATCGGAATTACTGGGCGTAAAGCGTGCGCAGGCGGTTTTGTAAGACAGATGTGAAATCCCCGGGCTCAACCTGGGACCTGCATTTGTGACTGCAAGGCTAGAGTACGGTAGAGGGGGATGGAATTCCGCGTGTAGCAGTGAAATGCGTAGATATGCGGAGGAACACCGATGGCGAAGGCAATCCCCTGGACCTGTACTGACGCTCATGCACGAAAGCGTGGGGAGCAAACAGG
## 655                                                          ACGGAGGGTGCAAACGTTAATCGGAATTACTGGGCGTAAAGCGCGCGTAGGCGGTCCGGCAAGTGGGATGTGAAAGCCCTGGGCTTAACCTGGGAACTGCATTCCAAACTACCGGGCTAGAGTATGGTAGAGGGAGGTAGAATTTCCTGTGTAGCGGTGAAATGCGTAGATATAGGAAGGAATACCAGTGGCGAAGGCGGCCTCCTGGACCAATACTGACGCTGAGGTGCGAAAGCGTGGGGAGCAAACAGG
## 656                                                          ACGGAGGATCCGAGCGTTATCCGGATTTATTGGGTTTAAAGGGAGCGTAGGTGGATTGTTAAGTCAGTTGTGAAAGTTTGCGGCTCAACCGTAAAATTGCAGTTGAAACTGGCAGTCTTGAGTACAGTAGAGGTGGGCGGAATTCGTGGTGTAGCGGTGAAATGCTTAGATATCACGAAGAACTCCGATTGCGAAGGCAGCTCACTAGACTGCAACTGACACTGATGCTCGAAAGTGTGGGTATCAAACAGG
## 658                                                          ACAGAGACTGCAAGCGTTATTCGGATTCACTGGGCGTAAAGGGTGCGCAGGCGGCCGTGTGTGTGAGATGTGAAATCCCGGAGCTTAACTCCGGAACTGCGTCTCAAACTACACGGCTAGAGCATTGGAGAGGGAAGTGGAATTCACGGTGTAGCAGTGAAATGCGTAGATATCGTGAGGAACACCAGAGGCGAAGGCGACTTCCTGGACAATTGCTGACGCTCAGGCACGAAAGCGTGGGGAGCAAAAGGG
## 659                                                          ACGTAGGGTGCGAGCGTTAATCGGAATTACTGGGCGTAAAGCGTGCGCAGGCGGTGATGTAAGACAGATGTGAAATCCCCGGGCTCAACCTGGGAACTGCGTTTGTGACTGCATCACTCGAGTACGGCAGAGGGAGGTGGAATTCCGCGTGTAGCAGTGAAATGCGTAGATATGCGGAGGAACACCGATGGCGAAGGCAGCCTCCTGGGCCAGTACTGACGCTCATGCACGAAAGCGTGGGGAGCAAACAGG
## 662                                                          ACGAAGGCCCCAAGCGTTATCCGGAATTACTGGGCGTAAAGCGTCTGTAGGTGGTTTTTTAAGTCTTTCCGTGAAACTTCGGGGCTCAACCCTGAATTGCGGGAGAAACTGGAAAACTTGAGTGTGGGAGAGGCTAGCAGAACGGTAAGAGTAGGGGTGCAATCCGTTGATACTTACCAGAATACCAAAAGCGAAGGCAGCTAGCTGGAACACTACTGACACTGAGAGACGAAAGCGTGGGGAGCGAAAGGG
## 663                                                          ACGGAGGGTGCAAGCGTTGTCCGGAATCATTGGGCGTAAAGCGTTCGTAGGCGGCATGTCAAGTCTGGTGTTAAAGCCCGGGGCTCAACTCCGGTTCGGCACTGGATACTGGCAAGCTAGAATGTGGTAGAGGTAAAGGGAATTCCTGGTGTAGCGGTGAAATGCGTAGATATCAGGAGGAACACCGGTGGCGTAAGCGCTTTACTGGGCCATAATTGACGCTGAGGAACGAAAGCCGGGGGAGCAAATGGG
## 664                                                          ACGGAGGGTGCAAGCGTTAATCGGAATGACTGGGCGTAAAGCGCACGCAGGCGGGATGTTAAGTCAGATGTGAAATCCCCGGGCTTAACCTGGGAACTGCATTTGAAACTGGCATTCTTGAGTTTCGTAGAGGGGGGTAGAATTCCAGGTGTAGCGGTGAAATGCGTAGATATCTGGAGGAATACCGGTGGCGAAGGCGGCCCCCTGGACGGAGACTGACGCTCAGGTGCGAAAGCGTGGGGATCAAACAGG
## 665                                                          ACATAGGGTGCAAGCGTTGTCCGGAATTATTGGGCGTAAAGAGCTCGTAGGTGGTTAGTTACGTCGGATGTGAAAATCTAGGGCTCAACCCTAGACCTGCATCCGATACGGGCTAGCTTGAGTTTGGTAGGGGAGACTGGAATTCCTGGTGTAGCGGTGGAATGCGCAGATATCAGGAGGAACACCAATGGCGAAGGCAGGTCTCTGGGCCAATACTGACACTGAGGAGCGAAAGTGTGGGGAGCGAACAGG
## 666                                                          ACGGAGGGTGCAAGCGTTGTTCGGATTTATTGGGCGTAAAGCGCGCGCAGGCGGATTTGTAAGTCAGGTGTGAAATCTCGAGGCTCAACCTCGAAACTGCGCCTGAAACTGTGAATCTAGAATATGGTAGGGGGAAGGGGAATTTCACGTGTAGGGGTAAAATCCGTAGAGATGTGAAGGAACACCGGAGGCGAAAGCGCCTTCCTGGGCCATTATTGACGCTGAGGCGCGAAAGCGTGGGGAGCAAACAGG
## 667                                                          ACGAAGGGTGCAAGCGTTGTTCGGAATCATTGGGCGTAAAGCGCGCGCAGGCGGATTGATAAGTCAGATGTGAAATCTCGGGGCTCAACCTCGAAACTGCGTCTGAAACTGTTAGTCTAGAATGTCGGAGGGGGCAGGGGAATTTCACGTGTAGGGGTAAAATCCGTAGAGATGTGAAGGAACACCGGGGGCGAAGGCGCCTGCCTGGACGACTATTGACGCTGAGGCGCGAAAGCGTGGGGAGCAAACAGG
## 668                                                          ACGTAGGGTGCAAGCGTTGTCCGGAATTATTGGGCGTAAAGAGCTCGTAGGCGGTTTGTCACGTCTGCTGTGAAAATCCAAGGCTCAACCTTGGACTTGCAGTGGGTACGGGCAGGCTAGAGTGCGGTAGGGGAGATGGGAATTCCTGGTGTAGCGGTGGAATGCGCAGATATCAGGAGGAACACCAATGGCGAAGGCACATCTCTGGGCCGTAACTGACGCTGAGGAGCGAAAGCGTGGGGAGCAAACAGG
## 669                                                          ACGAGTGCCCCGAGCGTTATCCGGAATTATTGGGCGTAAAGGGTGTGTAGGTGGTCAGGTTAGTCTCACGTCAAAGCCTTCGGCTCAACCGAAGAACTGCGTGGGAAACGGCTTGACTTGAGGGTGCGAGGGGTGAATGGAACTCATGGTGTAGGGGTGAAATCCGTTGATATCATGGGGAACACCAAAAGCGAAGGCAATTCACTGGCGCACATCTGACACTGAAACACGAAAGCGTGGGTAGCGAATGGG
## 670                                                          ACGTAGGGTGCGAGCGTTAATCGGAATTACTGGGCGTAAAGCGTGCGCAGGCGGTTACATAAGCCAGATGTGAAATCCCCGGGCTTAACCTGGGAACTGCATTTGGGACTGTGTGGCTTGAGTATGGCAGAGGGAGGTGGAATTCCACGTGTAGCAGTGAAATGCGTAGAGATGTGGAGGAACACCGATGGCGAAGGCAGCCTCCTGGGCCAATACTGACGCTCATGCACGAAAGCGTGGGGAGCAAACAGG
## 671                                                          ACGAGGGGTCCAAGCGTTGTTCGGAATCATTGGGCGTAAAGCGGGTGTAGGTGGCTCTATAAGTCAGGAGTGAAATCCCCGAGCTTAACTTGGGAAGTGCTTTTGATACTGCAGAGCTTGAATGTGGGAGAGGATCGTAGAATTCCAGGTGTAGTGGTGAAATACGTAGATATCTGGAGGAATACCGGTGGCGAAGGCGGCGATCTGGCCCAACATTGACACTGAGACCCGAAAGCGTGGGGATCAAACAGG
## 672                                                          ACGGAGGGTGCAAGCGTTAATCGGAATTACTGGGCGTAAAGCGCGCGTAGGCGGTTGACTAAGTCTGCTGTGAAAGCCCCGGGCTTAACCTGGGAACTGCAGTGGATACTGGTTGGCTAGAGTATGGTAGAGGGTAGTGGAATTTCCGGTGTAGCAGTGAAATGCGTAGAGATCGGAAGGAACACCAGTGGCGAAGGCGGCTATCTGGACCAATACTGACGCTGAGGTGCGAAAGCGTGGGGAGCAAACAGG
## 674                                                          ACGGAGGGTGCAAGCGTTGCTCGGATTTACTGGGCGTAAAGCGCACGCAGGCGGTCTGGAAGGTCGGGTGTGAAAGCCCGGGGCTCAACCCCGGAACTGCACTCGAAACCGCCAGACTTGAGGTCGAGAGAGGAGGGTAGAATTCCTGGTGTAGAGGTGAAATTCGTTGATATCAGGAGGAATACCGGTGGCGAAGGCGGCCCTCTGGCTCGATTCTGACGCTCATGTGCGAAAGCGTGGGGAGCAAACAGG
## 675                                                          ACGTAGGGTGCAAGCGTTAATCGGAATTACTGGGCGTAAAGCGTGCGCAGGCGGATGTTTAAGCCAGATGTGAAAGCCCCGAGCTTAACTTGGGAATTGCGTTTGGAACTGAGCATCTAGAGTCTGTCAGAGGGGGATGGAATTCCACGTGTAGCAGTGAAATGCGTAGAGATGTGGAGGAACACCGATGGCGAAGGCAGCCCCCTGGGCCAATACTGACGCTCATGCACGAAAGCGTGGGGAGCAAACAGG
## 676                                                          ACGGAGGGTGCGAGCGTTATCCGGATTCACTGGGTTTAAAGGGTGCGTAGGTGGGTTGGTAAGTCAGTGGTGAAATCCTCGAGCTTAACTCGAGAACTGCCGTTGATACTACCAGTCTTGAATTCTGTGGAGGTAAGCGGAATATGTCATGTAGCGGTGAAATGCTTAGATATGACATAGAACACCGATAGCGAAGGCAGCTTACTACGCAGATATTGACACTGAGGCACGAAAGCGTGGGGATCAAACAGG
## 677                                                          ACGGAGGGTGCAAGCGTTATCCGGAATCACTGGGTTTAAAGGGTACGTAGGCGGTTTAATAAGTCAGGTGTGAAATACTGCAGCTTAACTGCGGAACTGCACATGATACTGTTAGACTTGAGTAGGGAGGAGGGACACGGAATGCATCATGTAGCGGTGAAATGCTTAGATATGATGTAGAACACCGATAGCGAAGGCAGTGTTCCATACCTATACTGACGCTGAGGTACGAAAGCGTGGGGAGCGAACAGG
## 678                                                          ACGGAGGGTGCAAGCGTTATCCGGATTCACTGGGTTTAAAGGGTGCGTAGGTGGGCATGTAAGTCAGAGGTGAAATCCCCGAGCTTAACTTGGGAACTGCCTTTGATACTATATGTCTTGAATATCGTGGAGGTAAGCGGAATATGTCATGTAGCGGTGAAATGCATAGAGATGACATAGAACACCAATTGCGAAGGCAGCTTGCTACACGATGATTGACACTGAGGCACGAAAGCGTGGGGAGCAAACAGG
## 679                                                          ACGTAGGTTGCGAGCGTTATCCGGAATTATTGGGCGTAAAGAGTTCGTAGGTGGTATGTTAAGTTGGATGTTAAACCTTTGAGCTCAACTCAAAGAATGTGTCCAAAACTGGCAAACTAGAGACAGGTAGAGGTAAGCGGAATTCTGTATGTAGGGGTAATATCCGTAGATATACAGAGGAACACCAAAAGCGAAGGCAGCTTACTGGACCTGTTCTGACACTCAAGAACGAAAGCGTGGGGAGCAAACGGG
## 680                                                          ACGAAGGGTGCAAGCGTTATTCGGATTGATTGGGCGTAAAGGGTGTGTAGGTGGTGTGCTAAGTCAACTGTTAAATTCCCTGGCTTAACCAGGGGTCAGCAGTAGATACTGGCGCGCTAGAGGATGGAAGAGAGAAGTGGAATTCTCGGAGTAGCGGTAAAATGCGTAGATCTCGAGAAGAACACCGATGGCGAAGGCAGCTTCTTGGTCCATTCCTGACACTGAGGCACGAAAGCGTGGGGAGCAAACAGG
## 681                                                          ACAGGGGGTGCAAGCGTTGCTCGGAATTACTGGGCGTAAAGCGTGCGCAGGCGGTTCAGTAAGTCGGAGGTGAAAGCCCGAGGCTCAACCTCGGAAATGCCTTCGAAACTACTGATCTTGAGTACGGGAGAGGGTCGCGGAATTCTTGGTGTAGAGGTGAAATTCGTAGATATCAGGAAGAACACCAGTGGCGAAAGCGGCGACCTGGTCCGAGACTGACGCTCATGCACGAAAGCGTGGGGAGCAAACAGG
## 682                                                          ACAGAGGGTGCAAGCGTTGTTCGGAATTACTGGGCGTAAAGCGTGCGTAGTCGGTATTGAGAGTCGCGGGTGAAATCCCAGGGCTTAACCCTGGAACTGCCTGCGAGACCTCAGTACTAGAGTGTGAGAGGGGATAGTGGAATACCCAGTGTAGCGGTGAAATGCGTAGAGATTGGGTGGAACACCGGTGGCGAAGGCGGCTATCTGGCTCACAACTGACGATCAGGCACGAAAGCGTGGGGAGCAAACAGG
## 684                                                          ACGGAGGGTGCAAGCGTTAATCGGAATTACTGGGCGTAAAGCGTGCGTAGGCGGCTCGCTAAGTCAGATGTGAAAGCCCCGGGCTCAACCTGGGAACGGCATTTGAAACTGGCAAGCTAGAGTTTAGGAGAGGAGAGTGGAATTTCAGGTGTAGCGGTGAAATGCGTAGAGATCTGAAGGAACACCAGTGGCGAAGGCGACTCTCTGGCCTAAAACTGACGCTGAGGTACGAAAGCGTGGGTAGCAAACAGG
## 685                                                          ACGGAGGGTGCAAGCGTTATCCGGAATCATTGGGTTTAAAGGGTCCGCAGGCGGGCATATAAGTCAGTGGTGAAAGCCTACAGCTTAACTGTAGAACTGCCATTGATACTGTATGCCTTGAATTCGATCGAAGTGGGCGGAATGTGTAGTGTAGCGGTGAAATGCATAGATATTACACAGAACACCGATAGCGAAGGCAGCTCACTAGGTCTGAATTGACGCTCATGGACGAAAGCGTGGGGAGCAAACAGG
## 686                                                          ACGTAGGGAACAAGCGTTGTCCGGATTTACTGGGCGTAAAGGGCACGCAGGCGGTTTTTTAAGTCAGATGTGAAAGGTACCGGCTCAACCGGTGACATGCATTTGAAACTGAAAGACTTGAGTATTGGAGAGGCAAGTGGAATTCCTAGTGTAGCGGTGAAATGCGTAGAGATTAGGAGGAACACCAGTGGCGAAGGCGGCTTGCTGGACAAATACTGACGCTGAGGTGCGAAAGCGTGGGGAGCAAACAGG
## 687                                                          ACGAGGGATCCTAGCGTTGTTCGGAATCATTGGGCGTAAAGCGGGTGTAGGTGGCTCTATAAGTCAGATGTGAAAGCCCTGGGCTTAACCCAGGAAGTGCATTTGATACTGCTGAGCTTGAGTGTGGGAGAGGCTAGTGGAATTCCTGGTGTAGTGGTGAAATACGTAGATATCAGGAGGAACATCGGTGGCGAAGGCGGCTAGCTGGCCCAACACTGACACTGAGACCCGAAAGCGTGGGGATCAAACAGG
## 688                                                         ACAGAGACCACAAGCGTTATCCGGAATTATTGGGCGTAAAGGGTGAGTAGGTGGTATTGTTAGTCGGGTTTGAAAGATCGTGGGCTTAACCCATGATAGGGACCCGAAACGGCAAAACTAGAATATGTAAGAGGTTAGTGGAACTATATGTGTAGGGGTAAAATCCGTTGATATATATGGGAACACCTAAAGCGAAGGCAGCTAACTGGTACATTATTGACACTGAATCACGAAAGCGTGGGTAGCGATACGG
## 689                                                          ACGGAGGATCCAAGCGTTATCCGGAATCATTGGGTTTAAAGGGTCCGTAGGCGGTTTTATAAGTCAGTGGTGAAATCTGGTCGCTCAACGATCAAACGGCCATTGATACTGTAGAACTTGAATTACTTGGAAGTAACTAGAATATGTAGTGTAGCGGTGAAATGCTTAGAGATTACATGGAATACCAATTGCGAAGGCAGGTTACTACAAGTGGATTGACGCTGATGGACGAAAGCGTGGGGAGCGAACAGG
## 690                                                          ACGTAGGGGGCAAGCGTTGTCCGGAATTACTGGGCGTAAAGCGCACGCAGGCGGATTAAAAAGTCAGCCGTAAAATGCACGGGCTCAACCTGTGTTAGTCAGCTGATACTATTAGTCTAGAGTATGTGAGAGGGAAGTGGAATTCCCGGTGTAGCGGTGAAATGCGTAGATATCGGGAGGAACACCAGTGGCGAAGGCGGCTTCCTGGCACAAAACTGACGCTCATGTGCGAAAGCTAGGGCAGCGAACGGG
## 691                                                          ACGTAGGGTGCGAGCGTTAATCGGAATTACTGGGCGTAAAGCGTGCGCAGGCGGTTTTGTAAGACAGATGTGAAATCCCCGGGCTCAACCTGGGACCTGCATTTGTGACTGCAAAGCTGGAGTGCGGCAGAGGGGGATGGAATTCCGCGTGTAGCAGTGAAATGCGTAGATATGCGGAGGAACACCGATGGCGAAGGCAATCCCCTGGGCCTGCACTGACGCTCATGCACGAAAGCGTGGGGAGCAAACAGG
## 692                                                           ACGTAGGTGGCAAGCGTTGTCCGGAATTATTGGGCGTAAAGCGCGCGCAGGCGGGATATCAAGTCTGTCTTAAAAGTGCGAGGCTCAACCTCGTGAGGGGACAGAAACTGGTATTCTTGAGTGTCGGAGAGGAAAGTGGAATTCCAAGTGTAGCGGTGAAATGCGTAGAGATTTGGAAGAACACCAGTGGCGAAGGCGACTTTCTGGACGATGTCTGACGCTGAGGCGCGAAAGCTAGGGGAGCGAACGGG
## 693                                                          ACGGAGGGTGCGAGCGTTAATCGGAATTACTGGGCGTAAAGCGCGCGTAGGCGGTTCGTTGTGTCCGCTGTGAAAGCCCTGGGCTTAACCTGGGAACTGCAGTGGAAACTGGCGGGCTAGAGTATGGTAGAGGAGAGTGGAATTTCCGGTGTAGCAGTGAAATGCGTAGAGATCGGAAGGAACACCAGTGGCGAAGGCGGCTCTCTGGACCAATACTGACGCTGAGGTGCGAAAGCGTGGGGAGCAAACAGG
## 696                                                          ACAGAGGGTGCAAGCGTTGTTCGGAATTATTGGGCGTAAAGCGAGTGTAGGCGGTTATCTAAGTCTGGTGTGAAAGCCCGGGGCTCAACCTCGGAAGTGCACTGGATACTGGGTAACTAGAGGGCGATAGAGGGGAACGGAATTAATGGTGTAGGGGTGAAATCCGTAGATATCATTAGGAACACCGAAAGCGAAGGCTGTTCCCTGGGTCGCAACTGACGCTAAGACTCGAAAGCATGGGTAGCAAACAGG
## 697                                                          ACGAAGGCCCCAAGCGTTATCCGGAATTACTGGGCGTAAAGCGTCTGTAGGTGGTTTGGAAAGTTTCAAGTGAAACGTCAGGGCTCAACCCTGTACTTGCTTGGAAAACTATCAAACTAGAGTGTGGGAGAGGCAAGCAGAACGGTATGAGTAGGGGTGCAATCCGTTGATACATACCAGAATACCAAAAGCGAAGGCAGCTTGCTGGAACACAACTGACACTGAGAGACGAAAGCGTGGGGAGCAAAAGGG
## 698                                                                                                               ACAGATGGAGCGAGCTTCCTGCTATATGATTAGGTAGATAGCGTGTGAAGATGATTCTAGTTTTATTTTAAAGTGGGGGAATTTTCTATGGATTAGTAAAATGATTGTATATAGGAAGGAACGCCAACAGCGAAGGCACTCATTTAAATATAACTCAAATTCAAACACGGAAGTCCGAGGAGCAAAACGGATTAGATAC
## 699                                                          ACGTAGGGTGCAAGCGTTGTCCGGATTTATTGGGCGTAAAGAGCTCGTAGGCGGTTTGTCGCGTCGGATGTGAAAACCTATGGCTTAACCATGGGCCTGCATTCGATACGGGCAAACTAGAGTTCGGTAGGGGAGACTGGAACTCCTGGTGTAGCGGTGAAATGCGCAGATATCAGGAAGAACACCGGTGGCGAAGGCGGGTCTCTGGGCCGATACTGACGCTGAGGAGCGAAAGCGTGGGGAGCAAACAGG
## 700                                                          ACGGAGGGTGCAAGCGTTATCCGGATTCACTGGGTTTAAAGGGTGTGTAGGCGGGCTATTAAGTCAGTGGTGAAATCTCCGGGCTCAACCCGGAAACTGCCATTGATACTATTAGTCTTGAATGTTGTTGAGGTGGGCGGAATATATCATGTAGCGGTGAAATGCATAGATATGATATAGAACACCGATTGCGAAGGCAGCTCACTAAACAACGATTGACGCTGAGGCACGAAAGCGTGGGGATCAAACAGG
## 701                                                          ACGAAGGACCCGAGCGTTATCCGGATTTATTGGGTTTAAAGGGTGCGTAGGCGGATTAGTAAGTCAGTGGTGAAAGCCTCTCGCTCAACGGGAGAACTGCCATTGATACTGCTAGTCTTGAGTATGGTTGAGGTGGGCGGAATGTGTCATGTAGCGGTGAAATGCTTAGATATGACACAGAACACCGATTGCGAAGGCAGCTCGCTAAGCCATAACTGACGCTGAGGCACGAAAGCGTGGGGAGCAAACAGG
## 702                                                          ACAGAGGGGGCAAGCGTTATTCGGAATTATTGGGCGTAAAGGGCGCGTAGGCGGTGATTTAAGTGAGATGTGTAATCCCCGGGCTTAACCTGGGAACTGCATCTCAGACTGGATCGCTAGAGTGCTGGAGAGGGTGGTAGAATTCCACGTGTAGCGGTGAAATGCGTAGAGATGTGGAGGAATACCAGTGGCGAAGGCGGCCACCTGGACAGTAACTGACGCTGAGGCGCGAAAGTGTGGGTAGCAAACAGG
## 704                                                          ACGGAGGGTGCAAGCGTTATCCGGATTTATTGGGTTTAAAGGGTCCGTAGGCGGACCTATAAGTCAGTGGTGAAAGCCCACCGCTCAACGGTGGAACTGCCATTGATACTGTAGGTCTTGAGTATGTATGAAGTTGGCGGAATGTGTGGTGTAGCGGTGAAATGCTTAGATATCACACAGAACACCGATTGCGAAGGCAGCTGACTAATACATAACTGACGCTGAGGGACGAAAGTGTGGGGATCAAACAGG
## 705                                                          ACAGAGGGTGCGAGCGTTAATCGGAATCACTGGGCTTAAAGGGTCCGTAGGCGGAACTGCAGGCGTCGAGTGAAATACCACGGCTCAACCGTGGAACTGCTCGGCGAACCGCAGTTCTTGAGGCGAGTAGAGGCGATCAGAACGATAGGTGGAGCGGTGAAATGCGTAGATATCTATCGGAATGCCGAAGGTGAAGACAGGTCGCTGGGCTCGTCCTGACGCTGAGGGACGAAAGCGTGGGGAGCAAACGGG
## 706                                                          ACGGAGGGGGCTAGCGTTGTTCGGAATTACTGGGCGTAAAGCGCACGTAGGCGGCTTGGTAAGTTAGAGGTGAAAGCCTGGAGCTCAACTCCAGAATTGCCTTTAAGACTGCTTGGCTTGAACACGGGAGAGGTGAGTGGAATTCCGAGTGTAGAGGTGAAATTCGTAGATATTCGGAAGAACACCAGTGGCGAAGGCGGCTCACTGGACCGTTGTTGACGCTGAGGTGCGAAAGCGTGGGGAGCAAACAGG
## 707                                                          ACGTAGGGTGCGAGCGTTAATCGGAATTACTGGGCGTAAAGAGTGCGTAGGCGGTTTTGTAAGACAGATGTGAAATCCCCGGGCTTAACCTGGGAACTGCATTTGTGACTGCAAGACTAGAGTGTGTCAGAGGGAGGTGGAATTCCGCGTGTAGCAGTGAAATGCGTAGAGATGCGGAAGAACACCGATGGCGAAGGCAGCCTCCTGGGATAACACTGACGCTCAGGCACGAAAGCGTGGGGAGCAAACAGG
## 708                                                          ACGAACTGTGCGAACGTTACTCGGAATCACTGGGCTTACAGGGTGCGTAGGTGGCTTGTTAAGCAGGGTGTGAAATCCCTCGGCTCAACCGAGGAACTGCGCTCTGAACTGGCGAGCTGGAGTATGCTAGAGGTGAATGGAACATCCAGTGGAGCGGTGAAATGTGTTGATATTGGATGGAACACCAGAGGCGAAGGCGATTCACTGGGGCATAACTGACACTGAGGCACGAAAGCCAGGGGAGCAAACGGG
## 709                                                          ACGTAGGGTGCAAGCGTTAATCGGAATTACTGGGCGTAAAGCGTGCGCAGGCGGTTATATAAGTCAGATGTGAAATCCCCGGGCTCAACCTGGGAACTGCATTTGAGACTGTATAGCTAGAGTACGGCAGAGGGGGATGGAATTCCGCGTGTAGCAGTGAAATGCGTAGATATGCGGAGGAACACCGATGGCGAAGGCAATCCCCTGGGCCTGCACTGACGCTCATGCACGAAAGCGTGGGGAGCAAACAGG
## 710                                                          ACAGAGGATGCAAGCGTTATCCGGAATCACTGGGCATAAAGCGTCTGTAGGTTGCTTATCAAGTCTGCTGTTAAAGATCAGGGCCTAACCCTGAAAAAGCAGTGGAAACTAGTAGGCTTGAGTGTGGTAGAGGTAGAGGGAATTCCTGGTGTAGCGGTGAAATGCGTAGATATTAGGAAGAACACCAATGGCGAAAGCACTCTACTGGGCCATAACTGACACTGAGAGACGACAGCTAGGGGAGCAAATGGG
## 711                                                          ACGTAGGTGGCAAGCGTTGTCCGGATTTATTGGGTTTAAAGGGTGCGTAGGTGGTTCCATAAGTCAGTGGTGAAATACGGCAGCTTAACTGTCGAGGTGCCATTGATACTGCGGAACTTGAGTACAGACGAGGTAGGCGGAATTGACGGTGTAGCGGTGAAATGCTTAGATATCGTCAAGAACACCGATAGCGAAGGCAGCTTACTAGACTGTAACTGACACTGAGGCACGAAAGTGTGGGGATCAAACAGG
## 712                                                          ACGTATGGTGCAAGCGTTATCCGGATTTACTGGGTGTAAAGGGAGCGCAGGCGGTGCGGCAAGTCTGATGTGAAAGCCCGGGGCTCAACCCCGGTACTGCATTGGAAACTGTCGTACTAGAGTGTCGGAGGGGTAAGCGGAATTCCTAGTGTAGCGGTGAAATGCGTAGATATTAGGAGGAACACCAGTGGCGAAGGCGGCTTACTGGACGATAACTGACGCTGAGGCTCGAAAGCGTGGGGAGCAAACAGG
## 713                                                          ACAGAGGGTGCAAGCGTTGCTCGGAATTATTGGGCGTAAAGGGCAAGTAGGTGGTCTCGTTTGTCTTGGGTGAAATCCCTGGGCTTAACCCAGGAACTGCCCCAGAAACGGCGAGACTAGAGTTCTGGAGAGGGTCGTGGAATTCCCAGTGTAGCGGTGAAATGCGTAGAGATTGGGAGGAACACCAGAGGCGAAAGCGGCGGCCTGGACAGATACTGACACTCAACTGCGAAAGCGTGGGGAGCAAACAGG
## 714                                                          ACGGAGGATCCGAGCGTTATCCGGATTTATTGGGTTTAAAGGGAGCGTAGATGGGTTGTTAAGTCAGTTGTGAAAGTTTGCGGCTCAACCGTAAAATTGCAATTGATACTGGCAGTCTTGAGTACAGTTGAGGTAGGCGGAATTCGTGGTGTAGCGGTGAAATGCTTAGATATCACGAAGAACTCCGATTGCGAAGGCAGCTTACTAACCTGTAACTGACATTGATGCTCGAAAGTGTGGGTATCAAACAGG
## 715                                                          ACGTAGGGGGCGAGCGTTGTTCGGAATTATTGGGCGTAAAGGGCATGTAGGCGGTTTTGTAAGCCTGGTGTGAAATCCTGCAGCTTAACTGTAGAATTGCATTGGGTACTGCGAGACTTGAATCACAGAGGGGAAACTAGAATTCCAGGTGTAGGGGTGGAATCTGTAGATATCTGGAAGAATACCGGTGGCGAAGGCGGGTTTCTAGCTGATGATTGACGCTGAGGTGCGAAAGTGCGGGGAGCAAACAGG
## 716                                                          ACGTAGGGACCAAGCGTTGTTCGGATTTACTGGGCGTAAAGGGCGCGTAGGCGGTGCGGTAAGTCACTTGTGAAATCTCTGAGCTTAACTCAGAACGGCCAAGTGATACTGCAGTACTAGAGTGCAGAAGGGGCAATCAGAATTCTTGGTGTAGCGGTGAAATGCGTAGATATCAAGAGGAATACCTGAGGTGAAGACGGGTTGCTAGGCTGACACTGACGCTGAGGCGCGAAAGCTAGGGGAGCAAACGGG
## 717                                                          ACAGAGGGTGCAAGCGTTGTTCGGAATTACTGGGCGTAAAGCGTGCGTAGTCGGTATTGAGAGTCATAGGTGAAATCCCAGGGCTTAACCCTGGAACTGCCCGTGAGACCTCAGTACTAGAGTGTGAGAGGGGATAGTGGAATACCAAGTGTAGCGGTGAAATGCGTAGAGATTTGGTAGAACACCGGTGGCGAAGGCGGCTATCTGGCTCACAACTGACGATCAGGCACGAAAGCGTGGGGAGCAAACAGG
## 718                                                          ACGGAGGATCCGAGCGTTATCCGGATTTATTGGGTTTAAAGGGAGCGTAGGCGGATTGTTAAGTCAGTTGTGAAAGTTTGCGGCTCAACCGTAAAATTGCAGTTGATACTGGCAGTCTTGAGTGCAGTAGAGGTGGGCGGAATTCGTGGTGTAGCGGTGAAATGCTTAGATATCACGAAGAACTCCGATTGCGAAGGCAGCTCACTGGAGTGTAACTGACGCTGATGCTCGAAAGTGTGGGTATCAAACAGG
## 719                                                          ACGAAGGGGGCTAGCGTTGTTCGGAATCACTGGGCGTAAAGCGCACGTAGGCGGATCTTTAAGTCAGGGGTGAAATCCCGAGGCTCAACCTCGGAACTGCCTTTGATACTGGAGGTCTCGAGTCCGGGAGAGGTGAGTGGAACTGCGAGTGTAGAGGTGAAATTCGTAGATATTCGCAAGAACACCAGTGGCGAAGGCGGCTCACTGGCCCGGTACTGACGCTGAGGTGCGAAAGCGTGGGGAGCAAACAGG
## 720                                                          ACGGAGGGTGCGAGCGTTAATCGGAATTACTGGGCGTAAAGGGTGCGTAGGTGGTTTATATAGTCATGCGTAGAAGCCCCGGGCTCAACCTGGGAATGTCGTGTGATACTGATAGACTGGAGTACAGGAGAGGGCAGTGGAATTTCCGGTGTAGCGGTGAAATGCGTAGATATCGGAAGGAACACCGGTGGCGAAGGCGGCTGCCTGGCCTGATACTGACACTGAGGCACGAAAGCGTGGGGAGCAAACAGG
## 721                                                          ACGGAGGGTGCGAGCGTTATCCGGAATTACTGGGTTTAAAGGGTGCGTAGGCGGCTTTGTAAGTCAGGAGTGAAAGTTTGCGGCTCAACCGTAAAATTGCTTCTGATACTGCATAGCTAGAATTAGGATGAGGTCAGCGGAATGTGGCATGTAGCGGTGAAATGCATAGATATGCCATAGAACACCAATTGCGAAGGCAGCTGGCTAGACCTGGATTGACGCTGAGGCACGAAAGCGTGGGGAGCGAACAGG
## 722                                                          ACGAAGGGGGCTAGCGTTGTTCGGAATTACTGGGCGTAAAGCGCGCGTAGGCGGTGATCTTTGTCAGAGGTGAAAGCCTGGGGCTCAACCCCAGAATTGCCTTTGAAACGGGATCGCTAGAGTCCGAGAGAGGATGGCGGAATTCCTAGTGTAGAGGTGAAATTCGTAGATATTAGGAAGAACACCGGTGGCGAAGGCGGCCATCTGGCTCGGTACTGACGCTCAGGCGCGAAAGCGTGGGGAGCAAACAGG
## 723                                                          ACAGGGGGTGCAAGCGTTGTTCGGAATCATTGGGCGTAAAGCGCGTGTAGGCGGACCGTTAAGTCTGGTGTGAAAGCCCTCGGCTCAACTGAGGAAGTGCATTGGATACTGGCGGCCTTGAGTCCTGGAGAGGGTAGCGGAATTCCCAGTGTAGAGGTGAAATTCGTAGATATTGGGAGGAACACCGGTGGCGAAGGCGGCTACCTGGACAGTGACTGACGCTGAGACGCGAAAGCATGGGTAGCAAACAGG
## 724                                                          ACGTAGGGTGCGAGCGTTAATCGGAATTACTGGGCGTAAAGCGTGCGCAGGCGGTTATGTAAGACAGTTGTGAAATCCCCGGGCTCAACCTGGGAATTGCATCTGTGACTGCATAGCTAGAGTACGGTAGAGGGGGATGGAATTCCGCGTGTAGCAGTGAAATGCGTAGATATGCGGAGGAACACCGATGGCGAAGGCAATCCCCTGGACCTGTACTGACGCTCATGCACGAAAGCGTGGGGAGCAAACAGG
## 725                                                         ACGAAGGGTGCAAGCGTTGTCCGGATTTATTGGGCGTAAAGCGTTCCGCAGGCGCGTGTGTAAGTCATGCTTCAAAGACCGGGGCTCAACCTCGGAAAGGGGTGTGATACTGCACATGTAGAATAATGTAGGGGCTACTGGAACTTTGCATGTAGGAGTGAAATCCGTTGATATGCAAAGGAACACCGAGGGCGTAGGCAGGTAGCTGGACATTTATTGACGCTCAGGGACGACAGCTAGGGTAGCGAAAGGG
## 726                                                          ACGTAGGGTGCAAGCGTTAATCGGAATTACTGGGCGTAAAGCGTGCGCAGGCGGATGTTTAAGCCAGATGTGAAAGCCCCGAGCTTAACTTGGGAATTGCGTTTGGAACTGAGCATCTAGAGTCTGTCAGAGGGGGATGGAATTCCACGTGTAGCAGTGAAATGCGTAGAGATGTGGAGGAACACCGATGGCGAAGGCAATCCCCTGGGCCTGCACTGACGCTCATGCACGAAAGCGTGGGGAGCAAACAGG
## 727                                                          ACAGAGGGTGCAAGCGTTGTTCGGAATTATTGGGCGTAAAGGGCAGGTAGGTTGTCTTTTAAGTCTGCTGTGAAATCCCTGGGCTCAACCTGGGAAGTGCGGTGGATACTGGAAGACTAGAGTACTATAGGGGTATGCGGAATTCCCGGTGTAGCGGTGAAATGCGTAGATATCGGGAGGAACACCAGAGGCGAAGGCGGCATACTGGAGAGTAACTGACACTAAACTGCGAAAGCGTGGGGAGCAAACAGG
## 728                                                         ACGTAGGCACCAAGCGTTGTCCGGATTTATTGGGCGTAAAGAGCTCGTAGGCGGTTCAGCAAGTCGGGTGTGAAAACTATGGGCTTAACCCATAGCCTGCATCCGAAACTGCTGTGACTAGAGTTCGGTAGGGGAGAGGGGAATTCCTAGTGTAGCGGTGAAATGCGCAGATATTAGGAGGAACACCGATGGCGAAGGCACCTCTCTGGGCCGATACTGACGCTGAGGAGCGAAAGCGTGGGTAGCAAACAGG
## 729                                                          ACGAAGGGGGCTAGCGTTGTTCGGAATTACTGGGCGTAAAGGGCGCGTAGGCGGCCCGATAAGTCAGATGTGAAAGCCCCGAGCTTAACTTGGGAACTGCATTTGATACTGTCGGGCTAGAGAGTGAGAGAGGAAAGTGGAATTGCGAGTGTAGAGGTGAAATTCGTAGATATTCGCAAGAACACCAGTGGCGAAGGCGGCTTTCTGGCTCACAACTGACGCTAAGGCGCGAAAGCGTGGGGAGCAAACAGG
## 731                                                          ACGGAGGGTGCAAGCGTTAATCGGAATAACTGGGCGTAAAGCGCACGCAGGCGGTTGGATAAGCGAGATGTGAAAGCCCCGGGCTCAACCTGGGAACTGCATTTCGAACTGTCCGACTAGAGTCTTGTAGAGGGGGGTAGAATTCCAGGTGTAGCGGTGAAATGCGTAGAGATCTGGAGGAATACCGGTGGCGAAGGCGGCCCCCTGGACAAAGACTGACGCTCAGGTGCGAAAGCGTGGGGAGCAAACAGG
## 732                                                          ACAGAGGTCCCGAGCGTTGTTCGGATTCACTGGGCGTAAAGGGTGCGTAGGTGGTGGGGTAAGTCGGATGTGAAATCTCCAAGCTCAACTTGGAAATGGCATTGGAAACTGCCCTGCTAGAGGATCGGAGGGGGGACTGGAATACTTGGTGTAGCAGTGAAATGCGTAGATATCAAGTGGAACACCAGTGGCGAAGGCGAGTCCCTGGACGATACCTGACACTGAGGCACGAAAGCTAGGGGAGCAAACAGG
## 733                                                          ACGAAGGGGGCGAGCGTTATTCGGAATCACTGGGCGTAAAGCGTGCGTAGGCTGCTTGGAAAGTTGGAAGTGAAAGCCCAGGGCTCAACCCTGGAATTGCTTTCAAAACTACCAGGCTCGGATTCGGGAGAGGATAGCGGAATTGTCAGTGTAGCAGTGAAATGCGTAGATATTGACAGGAACACCAGTGGCGCAAGCGGCTATCTGGACCGACATCGACGCTGAGGCACGAAAGCGTGGGGATCAAACAGG
## 734                                                          ACGTAGGGTGCAAGCGTTAATCGGAATTACTGGGCGTAAAGCGTGCGCAGGCGGTTATATAAGTCAGATGTGAAATCCCCGGGCTCAACCTGGGAACTGCATTTGAGACTGTATAGCTAGAGTACGGTAGAGGGGGATGGAATTCCGCGTGTAGCAGTGAAATGCGTAGATATGCGGAGGAACACCGATGGCGAAGGCAGTCCCCTGGGATAAGACTGACGCTCATGCACGAAAGCGTGGGGAGCAAACAGG
## 735                                                          ACGTAGGGTGCAAGCGTTAATCGGAATTACTGGGCGTAAAGCGTGCGCAGGCGGTTATATAAGACAGATGTGAAATCCCCGAGCTTAACTTGGGAACTGCATTTGTGACTGTATAGCTAGAGTACGGCAGAGGGGGATGGAATTCCGCGTGTAGCAGTGAAATGCGTAGATATGCGGAGGAACACCGATGGCGAAGGCAATCCCCTGGGCCTGTACTGACGCTCATGCACGAAAGCGTGGGGAGCAAACAGG
## 736                                                          ACAGAGGGTGCGAGCGTTATCCGGAATTATTGGGCGTAAAGCGTCCGCAGGTGGTTTGTTAAGTCGAGTATTAAAGACAACAGCTCAACTGTTGGAATGTATTCGATACTGGCAAGCTTGAGCCTTGGAGAGGTGAGCAGAATTCTGCATGTAGGGGTAAAATCCGTAGATATGCAGAGGAATACCAAAAGCGAAGGCAGCTCACTGGCCAAGTGCTGACACTCATGGACGAAAGCGTGGGGAGCAAACAGG
## 737                                                          ACAGAGGATGCAAGCGTTATCCGGAATCACTGGGCATAAAGCGTCTGTAGGTGGTTTGGTAAGTCTGCTGTTAAAGACTAGGGCTTAACCCTAGGAAAGCAGTGGAAACTGCTAGACTTGAGTGTGGTAGAGGTAGAGGGAATTCCTAGTGTAGCGGTGAAATGCGTAGATATTAGGAAGAACACCAATGGCGAAAGCACTCTACTGGGCCATAACTGACACTGAGAGACGACAGCTAGGGGAGCAAATGGG
## 740                                                          ACGTAGGGTGCGAGCGTTAATCGGAATTACTGGGCGTAAAGCGTGCGCAGGCGGTTTCGTAAGACAGAGGTGAAATCCCCGGGCTCAACCTGGGAACTGCCTTTGTGACTGCGAGGCTAGAGTATGGCAGAGGGGGGTGGAATTCCACGTGTAGCAGTGAAATGCGTAGAGATGTGGAGGAACACCGATGGCGAAGGCAGTCCCCTGGGATAAGACTGACGCTCATGCACGAAAGCGTGGGGAGCAAACAGG
## 741                                                          ACATAGGGTGCAAGCGTTGTCCGGAATTATTGGGCGTAAAGAGCTCGTAGGTGGTTCGTCACGTCGGATGTGAAACTCTGGGGCTTAACCCCAGACCTGCATTCGATACGGGCGAGCTTGAGTATGGTAGGGGAGTCTGGAATTCCTGGTGTAGCGGTGGAATGCGCAGATATCAGGAGGAACACCAATGGCGAAGGCAGGACTCTGGGCTAACACTGACGCTCATGCACGAAAGCGTGGGGAGCAAACAGG
## 742                                                          ACGGAGGGTGCAAGCGTTATCCGGAATCACTGGGTTTAAAGGGTACGTAGGCGGTTTAATAAGTCAGGTGTGAAATACCGCAGCCTAACTGCGGAACTGCACTTGATACTGTTAGACTTGAGAGGAGTGGAGGTATACGGAATGCATCATGTAGCGGTGAAATGCTTAGATATGATGTAGAACACCGATAGCGAAGGCAGTGTGCCACACTTTATCTGACGCTGAGGTACGAAAGCGTGGGTAGCGAACAGG
## 744                                                          ACGGAGGGTGCGAGCGTTAATCGGAATTACTGGGCGTAAAGCGCACGTAGGCGGTCATGTAAGTGGGATGTGAAATCCCCGGGCTTAACCTGGGACTTGCATCTCAGACTGCATGACTAGAGTATGGGAGAGGGTGGTGGAATTTCCGGTGTAGCGGTGAAATGCGTAGAGATCGGAAGGAACATCAGTGGCGAAGGCGGCCACCTGGCCTAATACTGACGCTGAGGTGCGAAAGCGTGGGGATCAAACAGG
## 745                                                          ACGGGGGGTGCAAGCGTTACTCGGAATCACTGGGCGTAAAGGGAGCGTAGGCGTTCTATTAAGTCTTGAGTGAAAGCCCGCAGCCTAACTGCGGAACTGCTTGGGAAACTGATAGAATTGAGTATGGAAGAGGTCAGTGGAATTTCCGGTGTAGGGGTAAAATCCGTAGATATCGGAAGGAATACCAGAAGCGAAGGCGACTGACTGGGACATTACTGACGCTGAGGCTCGAAAGCGTGGGGAGCAAACAGG
## 747                                                          ACAGAGGCCTCAAGCGTTGTTCGGAATTACTGGGCGTAAAGGGAGTGTAGGTGGTGATGCAAGTCAGATGTGAAAGCCCTGGGCTTAACCCGGGAATTGCATTTGATACTGCGTTGCTGGAGTGCAGGAGAGGTGAGAGGAATTCCTGGTGTAGGGGTGAAATCCGTAGATATCAGGAGGAATACCAATGGCGTAGGCATCTCGCTGGCCTGTAACTGACACTGAGACTCGAAAGCATGGGTAGCAAACAGG
## 748                                                          ACGTAGGGTGCAAGCGTTAATCGGAATTACTGGGCGTAAAGCGTGCGCAGGCGGTTATGTAAGACAGAGGTGAAATCCCCGGGCTCAACCTGGGAACTGCCTTTGTGACTGCATAGCTAGAGTACGGCAGAGGGGGATGGAATTCCGCGTGTAGCAGTGAAATGCGTAGATATGCGGAGGAACACCGATGGCGAAGGCAATCCCCTGGGCCTGCACTGACGCTCATGCACGAAAGCGTGGGGAGCAAACAGG
## 749                                                          ACGGAGGATCCGAGCGTTATCCGGATTTATTGGGTTTAAAGGGAGCGTAGGTGGACAGTTAAGTCAGTTGTGAAAGTTTGCGGCTCAACCGTAAAATTGCAGTTGATACTGGCTGTCTTGAGTACAGTAGAGGTGGGCGGAATTCGTGGTGTAGCGGTGAAATGCTTAGATATCACGAAGAACTCCGATTGCGAAGGCAGCTCACTGGACTGCAACTGACACTGATGCTCGAAAGTGTGGGTATCAAACAGG
## 750                                                          ACGGAGGGTGCAAGCGTTATCCGGATTTATTGGGTTTAAAGGGTCCGTAGGCGGATCGATAAGTCAGTGGTGAAAGCCCGCAGCTCAACTGCGGAACTGCCATTGATACTGTCGATCTTGAGTATAATTGAAGTGGGCGGAATGTGTGGTGTAGCGGTGAAATGCTTAGATATCACACAGAACACCGATTGCGAAGGCAGCTCACTAATTTGTAACTGACGCTGAGGGACGAAAGTGTGGGGATCAAACAGG
## 751                                                          ACAGAGACTGCAAGCGTTATTCGGATTCACTGGGCGTAAAGGGTGCGCAGGCGGCCAAGTGTGTGAGGCGTGAAAGCCCGGGGCTTAACCCCGGAATTGCGCCTCAAACTACATGGCTAGAGCATTGGAGAGGGTAGCAGAATTCATGGTGTAGCAGTGAAATGCGTAGATATCATGAGGAATACCAGAGGCGAAGGCGGCTACCTGGACAATTGCTGACGCTCAGGCACGAAAGCGTGGGGAGCAAAAGGG
## 752                                                          ACGTATGGGGCAAGCGTTGTTCGGAATTATTGGGCGTAAAGGGCTCGCAGGTGGTTTGTTAAGTTGGTGGTTTAATCTCTGGGCTCAACCCAGAGTCAGCCATCAAAACTGGCGAACTTGAGTACGATAGGGGATAGCGGAATTCTCGGTGTAGCGGTGGAATGCGTAGATATCGAGAGGAACACCAATGGCGAAGGCAGCTATCTGGATCGTAACTGACACTCATGAGCGAAAGTGCGGGGAGCAAACAGG
## 753                                                          ACGAAGGGGGCTAGCGTTGCTCGGAATCACTGGGCGTAAAGCGCACGTAGGCGGACTTTTAAGTCAGGGGTGAAATCCTGGAGCTCAACTCCAGAACTGCCTTTGATACTGGAAGTCTCGAGTTCGGGAGAGGTGAGTGGAACTGCGAGTGTAGAGGTGAAATTCGTAGATATTCGCAAGAACACCAGTGGCGAAGGCGGCTCACTGGCCCGATACTGACGCTGAGGTGCGAAAGCGTGGGGAGCAAACAGG
## 755                                                          ACGGAGGATCCAAGCGTTATCCGGAATCATTGGGTTTAAAGGGTCCGTAGGCGGCCTTATAAGTCAGTGGTGAAATCTCCTAGCTCAACTAGGAAACTGCCATTGATACTGTAGGGCTTGAATTTTTGTGAAGTAACTAGAATATGTAGTGTAGCGGTGAAATGCTTAGATATTACATGGAATACCAATTGCGAAGGCAGGTTACTAACAAACGATTGACGCTGATGGACGAAAGCGTGGGGAGCGAACAGG
## 756                                                          ACGTAGGGTGCGAGCGTTAATCGGAATTACTGGGCGTAAAGCGTGCGCAGGCGGTTTCGTAAGACAGAGGTGAAATCCCCGGGCTCAACCTGGGAACTGCCTTTGTGACTGCGAGGCTAGAGTATGGCAGAGGGGGGTGGAATTCCACGTGTAGCAGTGAAATGCGTAGAGATGTGGAGGAACACCGATGGCGAAGGCAATCCCCTGGACCTGTACTGACGCTCATGCACGAAAGCGTGGGGAGCAAACAGG
## 757                                                          ACAGAGGGTGCGAGCGTTAATCGGATTTACTGGGCGTAAAGCGTGCGTAGGCGGCTTTTTAAGTCGGATGTGAAATCCCCGAGCTTAACTTGGGAATTGCATTCGATACTGGGAAGCTAGAGTATGGGAGAGGATGGTAGAATTCCAGGTGTAGCGGTGAAATGCGTAGAGATCTGGAGGAATACCGATGGCGAAGGCAGCCATCTGGCCTAATACTGACGCTGAGGTACGAAAGCATGGGGAGCAAACAGG
## 759                                                          ACGGAGGGTGCAAGCGTTATCCGGAATCATTGGGTTTAAAGGGTCCGCAGGCGGATTTATAAGTCAGTGGTGAAAGCCTACAGCTTAACTGTAGAACTGCCATTGATACTGTAAGTCTTGAATTCGGTCGAAGTGGGCGGAATGTGTAGTGTAGCGGTGAAATGCTTAGATATTACACAGAACACCGATAGCGAAGGCAGCTCACTAGGCCTGGATTGACGCTCAGGGACGAAAGCGTGGGGAGCAAACAGG
## 761                                                          ACGGAGGGTCCGAGCGTTATCCGGATTTATTGGGTTTAAAGGGTGCGTAGGCGGTTTTATAAGTCAGCGGTGAAATTTTCCGGCTCAACCGGGATACTGCCGTTGATACTGTAAGGCTAGGATATGGTTGCTGTGGGTGGAATGTGTGGTGTAGCGGTGAAATGCTTAGAGATCACACAGAATATCGATTGCGAAGGCAGCTCACAAAGCCATTATTGACGCTGAGGCACGAAAGTGTGGGGATCAAACAGG
## 762                                                          ACAGAGGGTGCAAGCGTTAATCGGAATTACTGGGCGTAAAGCGTGCGTAGACGGTTATCTAAGTCGGATGTGAAATCCCCGGGCTCAACCTGGGAACTGCCTTTGTGACTGCGAGGCTAGAGTATGGCAGAGGGGGGTGGAATTCCACGTGTAGCAGTGAAATGCGTAGAGATGTGGAGGAACACCGATGGCGAAGGCAGCCCCCTGGGCCAATACTGACGCTCATGCACGAAAGCGTGGGGAGCAAACAGG
## 763                                                          ACGGAGGATCCAAGCGTTATCCGGATTCATTGGGTTTAAAGGGTGCGTAGGCGGAATGATAAGTCAGTGGTGAAATCTCGCAGCTTAACTGCGAAACTGCCATTGATACTGTCAATCTTGAGTACATTTGATGAGGGCGGAATGTGTCATGTAGCGGTGAAATGCTTAGATATGACACAGAACACCGATTGCGAAGGCAGCTCTCAAAACTGTAACTGACGCTGAGGCACGAAAGCGTGGGTATCAAACAGG
## 764                                                          ACGGAGGGTGCAAGCGTTAATCGGAATTACTGGGCGTAAAGCGCGCGTAGGCGGCTAGATAAGCTAGATGTGAAATCCCCGGGCTTAACCTGGGAACTGCATTTAGAACTGTCTAGCTAGAGTGCAGAAGAGGAGTGTGGAATTTCAGGTGTAGCGGTGAAATGCGTAGAGATCTGAAGGAACATCAGTGGCGAAGGCGACACTCTGGTCTGACACTGACGCTGAGGTGCGAAAGCGTGGGTAGCAAACAGG
## 766                                                          ACGTAGGGTGCAAGCGTTAATCGGAATTACTGGGCGTAAAGCGTGCGCAGGCGGTTTTGTAAGACAGTGGTGAAATCCCCGGGCTCAACCTGGGAACTGCCATTGTGACTGCAAGGCTGGAGTGCGGCAGAGGGGGATGGAATTCCGCGTGTAGCAGTGAAATGCGTAGATATGCGGAGGAACACCGATGGCGAAGGCAATCCCCTGGGCCTGCACTGACGCTCATGCACGAAAGCGTGGGGAGCAAACAGG
## 769                                                          ACGTAGGGTGCAAGCGTTGTCCGGAATTATTGGGCGTAAAGAGCTCGTAGGCGGTTTGTCGCGTCTGCTGTGAAAATCCGAGGCTCAACCTCGGACCTGCAGTGGGTACGGGCAAACTAGAGTGCGGTAGGGGAGAAGGGAATTCCTGGTGTAGCGGTGGAATGCGCAGATATCAGGAGGAACACCGATGGCGAAGGCACTTCTCTGGGCCGTAACTGACGCTGAGGAGCGAAAGCGTGGGGAGCGAACAGG
## 770                                                          ACGAAGGATGCAAGCGTTATCCGGAATTATTGGGTTTAAAGGGTGCGTAGGCGGACTGATAAGTCAGTGGTGAAAGCCTGCAGCTTAACTGCAGAATTGCCATTGATACTGTCGGTCTTGAGTACAGTTGAGGTAGGCGGAATGTGTCATGTAGCGGTGAAATGCTTAGATATGACACAGAACACCGATTGCGAAGGCAGCTTGCTAAGCTGTAACTGACGCTGAGGCACGAAAGCGTGGGGAGCAAACAGG
## 771                                                          ACGTAGGTGGCAAGCGTTGTCCGGATTTATTGGGTTTAAAGGGTGCGTAGGTGGTTTAATAAGTCAGTGGTGAAAGCTGGTTGCTCAACAATCAAGTTGCCATTGATACTGTTAGACTTGAGAGAAGTGGAGGCTGGTGGAATGGATGGTGTAGCGGTGAAATGCATAGATATCATCCAGAACGTCAATTGCGAAGGCAGCTGGCTGTACTTTTTCTGACACTGAGGCACGAAAGTGTGGGGATCAAACAGG
## 772                                                          ACGGAGGATGCAAGTGTTATCCGGAATCACTGGGCGTAAAGCGTCTGTAGGTGGTTTCATAAGTCAACTGTTAAATCTTGAGGCTCAACTTCAAAATCGCAGTCGAAACTATGAGACTGGAGTATAGTAGGGGTAAAGGGAATTTCCAGTGGAGCGGTGAAATGCGTAGAGATTGGAAAGAACACCAATGGCGAAGGCACTTTACTGGGCTATTACTGACACTGAGAGACGAAAGCTAGGGTAGCAAATGGG
## 773                                                          ACGTAGGGTGCGAGCGTTAATCGGAATTACTGGGCGTAAAGCGTGCGCAGGCGGTTTTGTAAGACAGGCGTGAAATCCCCGGGCTCAACCTGGGAACTGCGTTTGTGACTGCAAGGCTAGAGTATGGCAGAGGGGGGTGGAATTCCACGTGTAGCAGTGAAATGCGTAGAGATGTGGAGGAACACCGATGGCGAAGGCAGCCCCCTGGGCCAATACTGACGCTCATGCACGAAAGCGTGGGTAGCAAACAGG
## 775                                                          ACGTAGGTGGCGAGCGTTACTCGGAATTACTAGGCGTAAAGCGTACGTAGGCGGAAGTTTAAGTCTGTTGTGTAATCTCCAAGCTCAACTTGGAAACTGCAACAGAAACTGGGCTTCTTGAGTGAGGCAGAGGAAAACGGAATTCCTGGTGTAGCAGTGAAATGCGTAGATATCAGGAGGAACACCGGTGGCGAAGGCGGTTTTCTGGGCCTTTACTGACGCTAAAGTACGAAAGCTAGGGGAGCAAACGGG
## 778                                                          ACGGAGGGTGCAAGCGTTAATCGGAATAACTGGGCGTAAAGCGCACGCAGGCGGTTAGATAAGTCAGATGTGAAAGCCCCGGGCTCAACCTGGGAACTGCATTTGAAACTGTCTGACTAGAGTCTTGTAGAGGGGGGTAGAATTCCAGGTGTAGCGGTGAAATGCGTAGAGATCTGGAGGAATACCGGTGGCGAAGGCGGCCCCCTGGACAAAGACTGACGCTCATGCACGAAAGCGTGGGGAGCAAACAGG
## 780                                                           ACGAAGGCCCCAAGCGTTATCCGGAATTACTGGGCGTAAAGCGTCTGTAGGTGGTTTCAAAGGTTTCGTGTGAAACTTCGGGGCTCAACCCTGAATTGCACGGAAAACCATGGAACTTGAGTGTGGGAGAGGCTAGCAGAACGGTAAGAGTAGGGGTGCAATCCGTTGATACTTACCAGAATACCAAAAGCGAAGGCAGCTAGCTGGAACATTACTGACACTGAGAGACGAAAGCGTGGGGAGCGAAAGGG
## 781                                                          ACGAGAGCCCCAAGTGTTATCCGGAATTATTGGGCGTAAAGGGTGCGTAGGTGGCTGTATTAGTCTTTCGTTAAATCCTGGGGCTTAACCCCAGAACCGCGAAGGAAACGGTACGGCTAGAGGTTGTGTTGGGTGTATGGAACTCATGGAGTAGGGGTGAAATCCGTTGATATCATGGGGAACACCAAAAGCGAAGGCAGTACACTGGCACAAATCTGACACTGAGGCACGAAACCCTGGGTAGCGAATGGG
## 783                                                          ACGGAGGGTGCAAGCGTTATCCGGAATCATTGGGTTTAAAGGGTCCGCAGGCGGATTTATAAGTCAGTGGTGAAAGCCTACAGCTCAACTGTAGAACTGCCATTGATACTGTAAGTCTTGAATTCGGTCGAAGTGGGCGGAATGTGTAGTGTAGCGGTGAAATGCTTAGATATTACACAGAACACCGATAGCGAAGGCAGCTCACTAGGCCTGAATTGACGCTCATGGACGAAAGCGTGGGGAGCAAACAGG
## 784                                                          ACGTAGGGAGCAAGCGTTATCCGGATTTACTGGGTGTAAAGGGCGCGTAGGCGGGAATGCAAGTCAGATGTGAAATCTGGGGGCTCAACCCTCAAACTGCATTTGAAACTGTATTTCTTGAGTGATGGAGAGGCAAGTGGAATTCCTAGTGTAGCGGTGAAATGCGTAGATATTAGGAGGAACACCAGTGGCGAAGGCGACTTGCTGGACATTAACTGACGCTGAGGCGCGAAAGCGTGGGGAGCAAACAGG
## 785                                                         ACGTAGGATCCAAGCGTTATCCGGAATTACTGGGTGTAAAGAGTTGCGTAGGTGGCAGAGTAAGTAGGGAGTGAAAGCGTTCGGCTCAACCGAATATACATTTCCTAAACTGCTCAGCTAGAGAACGAGAGAGGTAAATGGAATTCCCAGTGTAGGAGTGAAATCCGTAGATATTGGGAGGAACACCGATGGCGTAGGCAGTTTACTGGCTCGTTTCTGACACTAAGGCACGAAAGCGTGGGGAGCAAACAGG
## 786                                                          ACGGAGGGTGCAAGCGTTGTTCGGAATTACTGGGCGTAAAGCGCGTGCAGGCGGTTTTATAAGTTTCGTGTGAAATCCCATGGCTTAACCATGGAACGGCACGGAATACTGCAAGACTTGAATACCGGAGAAGAGGGCGGAATTCCCAGTGTAGAGGTGAAATTCGTAGATATTGGGAGGAACACCGGCGGCGAAAGCGGCTCTCTAGACGGATATTGACGCTCATACGCGAGAGCGTGGGTAGCAAACAGG
## 787                                                          ACGGAGGATCCGAGCGTTATCCGGATTTATTGGGTTTAAAGGGTGCGTAGGCGGAAGAATAAGTCAGTGGTGAAAGTTTGCAGCTCAACTGTAAAATTGCCGTTGAAACTGTTTTTCTTGAGTGTAAATGAGGTAGGCGGAATGTGTTGTGTAGCGGTGAAATGCTTAGATATGACACAGAACACCGATTGCGAAGGCAGCTTACTAAGATACAACTGACGCTGAGGCACGAAAGCGTGGGGATCGAACAGG
## 788                                                          ACGAAGGGTGCTAGCGTTGTTCGGAATCATTGGGCGTAAAGCGTGCGTAGGCGGCTAAGTAAGTCGTTTGTGAAATCCCGAGGCTTAACTTCGGAATTGCAATCGAAACTGCTTAGCTTGAGTATGGTGGGGGATAGTGGAATTCCTAGTGTAGGGGTGAAATCCGTAGAGATTAGGAGGAACATCAGTGGCGAAAGCGACTATCTACGCCAATACTGACGCTGAGGCACGAAAGCGTGGGGAGCAAACAGG
## 789                                                          ACAGAGGATGCAAGCGTTATCCGGAATCACTGGGCATAAAGCGTCTGTAGGTGGTTTGATAAGTCTGCTGTTAAAGACTAGGGCTTAACCCTAGGAAAGCAGTGGAAACTGTCTGACTTGAGTATGGTAGAGGTACAGGGAATTCCTAGTGTAGCGGTGAAATGCGTAGATATTAGGAAGAACACCAATGGCGAAAGCACTGTACTGGGCCACAACTGACACTGAGAGACGACAGCTAGGGGAGCAAATGGG
## 791                                                          ACGAAGGGTGCAAGCGTTAATCGGAATTACTGGGCGTAAAGCGCGCGTAGGTGGTTTGTTAAGTTGGATGTGAAAGCCCCGGGCTCAACCTGGGAACTGCATCCAAAACTGGCAAGCTAGAGTATGGCAGAGGGTGGTGGAATTTCCTGTGTAGCGGTGAAATGCGTAGATATAGGAAGGAACACCAGTGGCGAAGGCGACCACCTGGGCTAATACTGACACTGAGGTGCGAAAGCGTGGGGAGCAAACAGG
## 793                                                          ACGTGGAGTGCGAGCGTTATCCGGATTTACTGGGCGTAAAGAGTTCGTAGGCGTTTGGTAAAGTTTCGTTTGAAAGACCGAGGCTCAACTTCGGAAACGGACGAAATACTTATCAGATTGAGATATCTAGGGGGTACTGGAACTGATAGTGTAGCAGTGAAATGCGTTGATATTATCAAGAACACCAAGGGCGAAGGCAGGTACCTGGGGATATTCTGACGCTGAGGAACGAAAGCTAGGGGAGCGAAAGGG
## 794                                                          ACGTAGGGTGCGAGCGTTAATCGGAATTACTGGGCGTAAAGCGTGCGCAGGCGGCCTTGAAAGTCAGATGTGAAATCCCCGAGCTCAACTTGGGAACTGCGTTTGAAACTCCAAGGCTAGAATATGTCAGAGGGGGGTAGAATTCCACGTGTAGCAGTGAAATGCGTAGAGATGTGGAGGAATACCAATGGCGAAGGCAGCCCCCTGGGATAATATTGACGCTCATGCACGAAAGCGTGGGGAGCAAACAGG
## 795                                                          ACGGAGGGTGCAAGCGTTATCCGGATTCACTGGGTTTAAAGGGTGCGTAGGTGGGTTGGTAAGTCAGTGGTGAAATCTCCGAGCTTAACTTGGAAACTGCCATTGATACTATCAATCTTGAATATTGTGGAGGTTAGCGGAATATGTCATGTAGCGGTGAAATGCTTAGATATGACATAGAACACCAATTGCGAAGGCAGCTGGCTACACATATATTGACACTGAGGCACGAAAGCGTGGGGATCAAACAGG
## 796                                                          ACGAAGGGGGCTAGCGTTGTTCGGAATTACTGGGCGTAAAGCGCACGTAGGCGGGTTTGTAAGTAGAGGGTGAAATCCCAGAGCTCAACTCTGGAACTGCCTTCTAGACTGCAAGCCTGGAGGTCAGGAGAGGCGAGTGGAATACCGAGTGTAGAGGTGAAATTCGTAGATATTCGGTGGAACACCAGTGGCGAAGGCGACTCGCTGGACTGATACTGACGCTGAGGTGCGAAAGCGTGGGGAGCAAACAGG
## 797                                                         ACAGAGGGTGCAAGCGTTATCCGGATTTATTGGGCGTAAAGCGTTTCGTAGGCGGTTTGGAAAGTTATCCTTCAAAGACCACGGCTTAACCGGGGGAAGGGGGGTAATACTGCCAGACTTGAAATATGGTGGGGTATCTGGAACTGATGGTGTAGTAGTGAAATACGTTGATATCATCAGGAACTCCGAGGGCGAAGGCAGGATACTAACCATTTTTTGACGCTGAGGAACGACAGCTAGGGGAGCGAAAGGG
## 798                                                          ACGGAGGGCGCGAGCGTTAATCGGAATCACTGGGCGTAAAGCGCGCGTAGGCGGCCGATCAAGTCAGAGGTGAAAGCCCTCGGCTCAACCGAGGAATTGCCTTTGATACTGGTCGGCTGGAGTGCGGGAGAGGGTGGCGGAATTCCCGGTGTAGGGGTGAAATCCGTAGAGATCGGGAGGAACACCAGTGGCGAAGGCGGCCACCTGGACCGCAACTGACGCTGAGGCGCGAAAGCATGGGGAGCAAACAGG
## 799                                                          ACGAGGGGTGCAAGCGTTATTCGGAATAACTGGGCGTAAAGAGCGCGTAGGCGGCTTGTTAAGTCTGTTGTTAAATTACCTAGCTTAACTGGGAAGAGGCAATAGAAACTGGCGAGCTAGAGGACAAGAGAGAGAAGTGGAATTCTCGGAGTAGCGGTAAAATGCGTAGATCTCGAGAGGAACACCGATGGCGAAGGCAGCTTCTTGGCTTGTATCTGACGCTCAAGTGCGAAAGCGTGGGGAGCAAACAGG
## 800                                                          ACGTAGGGTGCAAGCGTTAATCGGAATTACTGGGCGTAAAGCGTGCGCAGGCGGCTTTGCAAGACAGATGTGAAATCCCCGGGCTCAACCTGGGAACTGCATTTGTGACTGCAAGGCTAGAGTACGGTAGAGGGGGATGGAATTCCGCGTGTAGCAGTGAAATGCGTAGATATGCGGAGGAACACCGATGGCGAAGGCAATCCCCTGGGCCTGCACTGACGCTCATGCACGAAAGCGTGGGGAGCAAACAGG
## 801                                                          ACGGAGGGTGCAAGCGTTGTCCGGAATCATTGGGCGTAAAGAGTTCGTAGGTGGTATGTTAAGTCTGGTGTTAAAGGCAGAAGCTCAACTTCTGTAAGGCACTGGATACTGGCAAACTTGAATGTGGTAGAGGTAAAGGGAATTCCTGGTGTAGCGGTGAAATGCGTAGATATCAGGAGGAACATCGGTGGCGAAAGCGCTTTACTGGGCCATTATTGACACTGAGGAACGAAAGCCGGGGTAGCAAATGGG
## 802                                                          ACGAAGGGGGCTAGCGTTGTTCGGATTTACTGGGCGTAAAGCGCGTGTAGGCGGACTGGAAAGTTGGGGGTGAAATCCCGAGGCTCAACCTCGGAACTGCCTCCAAAACTTCCAGTCTGGACTCAAGCAGAGGCAAGTGGAATTGCGAGTGTAGAGGTGAAATTCGTAGATATTCGCAGGAACACCAGTGGCGAAGGCGACTTGCTGGGCTTGTAGTGACGCTGAGACGCGAAAGCGTGGGGAGCAAACAGG
## 803                                                           ACGGAGAGGGCGAGTGTTACTCATAATGACTGGGCGTAAAGGGTACGTAGGTTGTTAGGTAAGCGGTTAGTGAAATGAGAGGTTAATGACCTTTAAACGCTTTCTGAACTACCTAGCTTGAGTGCTGTTGAGGATACTGGTACTTTTAAAGTAGAGGTGATATTCGATAATATTAAAAGGACAACTTAAGGCGAAGGCAAGTATCTAGGAAGCACTGACACTGAGGTACTGAAGCATGGGTAGCAAATCGG
## 805                                                          ACGGAGGGTGCAAGCGTTACTCGGAATCACTGGGCGTAAAGGATGCGTAGGCTGTAATATAAGTCAGAAGTGAAATCCAACGGCTTAACCGTTGAACTGCTTTTGAAACTGTTTTACTAGAATATGGGAGAGGTAGATGGAATTGGTGGTGTAGGGGTAAAATCCGTAGATATCACCAGGAATACCGATTGCGAAGGCGATCTACTGGAACATTATTGACGCTGAGGCATGAAAGCGTGGGGAGCAAACAGG
## 806                                                          ACATAGGGTGCAAGCGTTGTCCGGAATTATTGGGCGTAAAGAGCTCGTAGGTCGTTTGTTACGTCGGATGTGAAAACCTGAGGCTCAACCTCAGGCCTGCATTCGATACGGGCAAACTAGAGTTTGGTAGGGGAGACTGGAATTCCTGGTGTAGCGGTGGAATGCGCAGATATCAGGAGGAACACCAATGGCGAAGGCAGGACTCTGGGCCATTACTGACACTGAGGAGCGAAAGCGTGGGGAGCGAACAGG
## 807                                                          ACGAAGGGTGCAAGCGTTATCCGGATTCATTGGGTTTAAAGGGTGCGTAGGCGGAGCGTTAAGTCAGTGGTGAAATCCTGCAGCTCAACTGTAGACTTGCCATTGATACTGGCGCTCTTGAGTGCGCTTGAAGTGGGCGGAATGTGCCGTGTAGCGGTGAAATGCTTAGATATGGCACAGAACACCAATTGCGAAGGCAGCTCACTAAGGCGATACTGACGCTGAGGCACGAAAGCGTGGGGATCGAACAGG
## 808                                                          ACGGGAGTGGCAAGCGTTATCCGGAATTATTGGGCGTAAAGCGTCCGCAGGCGGCCTTGAAAGTCTGTCGTTAAAGCGTGGAGCTTAACTCCATTTAAGCGATGGAAACTACAAGGCTAGAGTGTGGTAGGGGCAGAGGGAATTCCCGGTGTAGCGGTGAAATGCGTAGATATCGGGAAGAACACCAGTGGCGAAGGCGCTCTGCTGGGCCATAACTGACGCTCATGGACGAAAGCCAGGGGAGCGAAAGGG
## 809                                                          ACGTAGGGTGCGAGCGTTAATCGGAATTACTGGGCGTAAAGCGTGCGCAGGCGGTTTTGTAAGTCAGATGTGAAAGCCCCGGGCTCAACCTGGGAACTGCGTTTGAAACTACAAGGCTAGAGTGTGTCAGAGGGGGGTAGAATTCCACGTGTAGCAGTGAAATGCGTAGAGATGTGGAGGAATACCGATGGCGAAGGCAGCCCCCTGGGATAACACTGACGCTCATGCACGAAAGCGTGGGGAGCAAACAGG
## 810                                                          ACGGAGGGGGCTAGCGTTGTTCGGAATCACTGGGCGTAAAGCGTGCGTAGGCGGCCATTCAAGTCAGAGGTGAAAGCCCAGGGCTCAACCTTGGAACTGCCTTTGAAACTAGATGGCTCGAACACGGGAGAGGTGAGTGGAATTCCGAGTGTAGAGGTGAAATTCGTAGATATTCGGAAGAACACCAGTGGCGAAGGCGGCTCACTGGACCGTTGTTGACGCTGAGGCACGAAAGCGTGGGGAGCAAACAGG
## 811                                                          ACGGAGGGGGCTAGCGTTATTCGGAATTACTGGGCGTAAAGCGCACGTAGGCGGATTGGAAAGTCAGAGGTGAAATCCCAGAGCTCAACTTTGGAACTGCCTTTGAAACTCCCAGTCTTGAGGTCGAGAGAGGTGAGTGGAATTCCGAGTGTAGAGGTGAAATTCGTAGATATTCGGAGGAACACCAGTGGCGAAGGCGGCTCACTGGCTCGATACTGACGCTGAGGTGCGAAAGCGTGGGGAGCAAACAGG
## 812                                                          ACGGAGGGTGCAAGCGTTAATCGGAATGACTGGGCGTAAAGCGCACGCAGGCGGTCTGTTAAGTTGGATGTGAAATCCCCGGGCTTAACCTGGGAACTGCATTCAAAACTGACAGGCTAGAGTCTCGTAGAGGGGGGTAGAATTCCAGGTGTAGCGGTGAAATGCGTAGAGATCTGGAGGAATACCGGTGGCGAAGGCGGCCCCCTGGACGAAGACTGACGCTCAGGTGCGAAAGCGTGGGGAGCAAACAGG
## 813                                                          ACGGAAGGTCCGGGCGTTATCCGGATTTATTGGGTTTAAAGGGAGCGTAGGCCGGAGATTAAGCGTGTTGTGAAATGTAGATGCTCAACATCTGCACTGCAGCGCGAACTGGTTTCCTTGAGTACGCACAAAGTGGGCGGAATTCGTGGTGTAGCGGTGAAATGCTTAGATATCACGAAGAACTCCGATTGCGAAGGCAGCTCACTGGAGCGCAACTGACGCTGAAGCTCGAAAGTGCGGGTATCGAACAGG
## 814                                                          ACGGAGGGTGCAAGCGTTAATCGGAATTACTGGGCGTAAAGGGTGCGTAGGTGGTTTGCTAAGTTATCTGTGAAATTCCTGGGCTTAACCTGGGCAGGTCAGATAAAACTGGTGAACTCGAGTATGGGAGAGGGTAGTGGAATTTCCGGTGTAGCGGTGAAATGCGTAGAGATCGGAAGGAACACCAGTGGCGAAGGCGGCTACCTGGCCTAATACTGACACTGAGGCACGAAAGCGTGGGGAGCAAACAGG
## 815                                                          ACGTAGGTGGCGAGCGTTACTCGGAGTTACTGGGCGTAAAGGGTCCGTAGGTGGCCATCTAAGTCTGAGGTGAAAGCCTGCAGCTCAACTGCAGAACGGCCTTGGGTACTGGGTGGCTAGAGTGCAGGAGAGGTAAGTGGAATTCGTGGTGTAGCGGTGGAATGCGTAGATATCACGAGGAACACCAGAGGCGAAAGCGGCTTACTGGACTGCAACTGACACTGAGGGACGAAAGCCAGGGGAGCAAAAAGG
## 816                                                          ACGTAGGGTGCAAGCGTTAATCGGAATTACTGGGCGTAAAGCGTGCGCAGGCGGTCTTGCAAGACAGATGTGAAATCCCCGGGCTTAACCTGGGAACTGCATTTGTGACTGCAAGGCTGGAGTGCGGCAGAGGGGGATGGAATTCCGCGTGTAGCAGTGAAATGCGTAGATATGCGGAGGAACACCGATGGCGAAGGCAATCCCCTGGGCCTGCACTGACGCTCATGCACGAAAGCGTGGGGAGCAAACAGG
## 817                                                          ACGAAGGGACCTAGCGTAGTTCGGAATTACTGGGCTTAAAGAGTTCGTAGGTGGTTAAAAAAGTTGATGGTGAAATCCCAAGGCTCAACCTTGGAACTGCCATCAAAACTTTTTAGCTAGAGTGTGATAGAGGAAAGTGGAATTTCTAGTGTAGAGGTGAAATTCGTAGATATTAGAAAGAACATCAAAAGCGAAGGCAACTTTCTGGATCATTACTGACACTGAGGAACGAAAGCATGGGTAGCGAAGAGG
## 818                                                          ACAGAGGGTGCAAACGTTGCTCGGAATTATTGGGCGTAAAGCGCGTGTAGGCGGTCTCGTAAGTCGACTGTGAAAGCCCTTGGCTCAACCAAGGAAGTGCAGTCGAAACTGCGAGACTAGAGTGCTGAAGAGGATAGCGGAATGGCCAGTGTAGAGGTGAAATTCGTAGATATTGGCTAGAACACCGGTGGCGAAGGCGGCTATCTGGGAAGTTACTGACGCTGAGACGCGAAAGCGTGGGGAGCAAACAGG
## 821                                                          ACGGGAGTGGCAAGCGTTATCCGGAATTATTGGGCGTAAAGCGTCCGCAGGCGGTCTTTTAAGTCTGCTGTTAAAGCGTGGAGCTTAACTCCATTTCGGCAGTGGAAACTGGAAGACTAGAGTGTGGTAGGGGCAGAGGGAATTCCCGGTGTAGCGGTGAAATGCGTAGATATCGGGAAGAACACCAGTGGCGAAGGCGCTCTGCTGGGCCATAACTGACGCTCATGGACGAAAGCCAGGGGAGCGAAAGGG
## 822                                                          ACGGAGGGTGCAAGCGTTATCCGGATTCACTGGGTTTAAAGGGTGCGTAGGTGGGCAGGTAAGTCAGTGGTGAAATCTTCGAGCTTAACTCGGAAACTGCCATTGATACTATCTGTCTTGAATATCCTGGAGGTGAGCGGAATATGTCATGTAGCGGTGAAATGCTTAGATATGACATAGAACACCAATTGCGAAGGCAGCTCACTACGGGATGATTGACACTGAGGCACGAAAGCGTGGGGATCAAACAGG
## 823                                                          ACGAGGGGTCCAAGCGTTGTTCGGAATTATTGGGCGTAAAGCGGGTGTAGGTGGATCTATAAGTCAGGAGTGAAAGCCCCGAGCTTAACTTGGGAAGTGCTTTTGATACTGTAGATCTTGAATGTGGGAGAGGATCGTGGAATTCCAGGTGTAGTGGTGAAATACGTAGATATCTGGAGGAACACCGGTGGCGAAGGCGGCGATCTGGCCCAACATTGACACTGAGACCCGAAAGCATGGGGATCAAACAGG
## 824                                                          ACGGAGGGTGCAAGCGTTATCCGGATTCACTGGGTTTAAAGGGTGCGTAGGTGGGCAGGTAAGTCAGTGGTGAAATCCCCGAGCTCAACTTGGGAACTGCCATTGATACTATCTGTCTTGAATACCGTGGAGGTTAGCGGAATATGTCATGTAGCGGTGAAATGCTTAGATATGACATAGAACACCGATTGCGAAGGCAGCTGGCTACACGAATATTGACACTGAGGCACGAAAGCGTGGGGATCAAACAGG
## 825                                                          ACGTAGGTGGCAAGCGTTGTCCGGATTTACTGGGCGTAAAGGGAGCGTAGGTGGATATTTAAGTGGGATGTGAAATACTCGGGCTTAACCTGGGTGCTGCATTCCAAACTGGATATCTAGAGTGCAGGAGAGGAAAGTAGAATTCCTAGTGTAGCGGTGAAATGCGTAGAGATTAGGAAGAATACCAGTGGCGAAGGCGACTTTCTGGACTGTAACTGACACTGAGGCTCGAAAGCGTGGGGAGCAAACAGG
## 827                                                          ACGGAGGGTGCAAGCGTTAATCGGAATTACTGGGCGTAAAGCGCACGCAGGCGGTTGGATAAGTTAGATGTGAAAGCCCCGGGCTCAACCTGGGAATTGCATTTAAAACTGTCCAGCTAGAGTCTTGTAGAGGGGGGTAGAATTCCAGGTGTAGCGGTGAAATGCGTAGAGATCTGGAGGAATACCGGTGGCGAAGGCGGCCCCCTGGACCTGTACTGACGCTCATGCACGAAAGCGTGGGGAGCAAACAGG
## 828                                                          ACGTAGGGTGCAAGCGTTAATCGGAATTACTGGGCGTAAAGCGTGCGCAGGCGGTTATATAAGACAGATGTGAAATCCCCGGGCTCAACCTGGGAACTGCATTTGTGACTGTATAGCTAGAGTACGGTAGAGGGGGATGGAATTCCGCGTGTAGCAGTGAAATGCGTAGATATGCGGAGGAACACCGATGGCGAAGGCAATCCCCTGGGCCTGTACTGACGCTCATGCACGAAAGCGTGGGGAGCAAACAGG
## 830                                                      ACAGAGACCTCAAGCGTTATCCGGAATCATTGGGCGTAAAGCGTACCGATAGGTGGTTTAGAAAGTCAGAAGTGAAATCTCCAAGCTCAACTTGGAGTCTGTCTTTTGAAACTTCTAAACTAGAGGGGCAAAGAGGAAGCTGGAACGAACGGTGTAGTAGTGAAATGCGTTGATATCGTTCGGAACACCAATAGCGAAGGCAAGCTTCTGGGTGCCACCTGACACTGCTAGGACGAGAGCGTGGGGAGCGAATGGG
## 831                                                          ACGTAGGGTGCAAGCGTTGTCCGGATTTATTGGGCGTAAAGAGCTCGTAGGCGGTTTGTTACGTCGGATGTGAAATCCTGGAGCTTAACTCCGGGCCTGCATTCGATACGGGCAGACTAGAGTGTTGTAGGGGAGACTGGAACTCCTGGTGTAGCGGTGAAATGCGCAGATATCAGGAAGAACACCGATGGCGAAGGCAGGTCTCTGGGCAACTACTGACGCTGAGGAGCGAAAGCGTGGGGAGCAAACAGG
## 832                                                          ACGGAGGGTGCAAGCGTTGTTCGGAATTACTGGGCGTAAAGCGCGTGCAGGTGGTTTTTTAAGTCTCGTGTGAAATCCCTCGGCTTAACCGAGGAACTGCGCGAGATACTGGAGAACTTGAATACGGGAGAAGAGGGCGGAATTCCCAGTGTAGAGGTGAAATTCGTAGATATTGGGAGGAACACCGGCGGCGAAAGCGGCTCTCTAGACCGATATTGACGCTCATACGCGAGAGCGTGGGTAGCAAACAGG
## 833                                                          ACGGAGGGTGCAAGCGTTATCCGGAATCATTGGGTTTAAAGGGTCCGCAGGCGGATTTATAAGTCAGTGGTGAAATCCTATCGCTTAACGATAGAACTGCCATTGATACTGTAAGTCTTGAATTCGGTCGGAGTGGGCGGAATGTGTAGTGTAGCGGTGAAATGCATAGATATTACACAGAACACCGATAGCGAAGGCAGCTCACTAGGCCTGAATTGACGCTCATGGACGAAAGCGTGGGGAGCAAACAGG
## 834                                                          ACAGAGGGTGCAAGCGTTGTTCGGATTTACTGGGCGTAAAGCGTGCGTAGGTTGTATTGAGAGTCGCGGGTGAAATCCCAGGGCTTAACCCTGGAACTGCCTGCGAGACCTCAGTACTGGAGTATGAAAGAGGATAGTGGAATTCCTGGTGTAGCGGTGAAATGCGTAGAGATCAGGAGGAACACCTGTGGCGAAGGCGGCTATCTGGTTCATTACTGACACTGAGGCACGAAAGCGTGGGTAGCAAACAGG
## 835                                                          ACGAAGGGGGCTAGCGTTGCTCGGAATCACTGGGCGTAAAGCGCACGTAGGCGGATCTTTAAGTCAGAGGTGAAATCCCAAGGCTCAACCTTGGAACTGCCTTTGATACTGGGGATCTCGAGTCCGGAAGAGGTGAGTGGAACTCCGAGTGTAGAGGTGAAATTCGTAGATATTCGGAAGAACACCAGTGGCGAAGGCGGCTCACTGGTCCGGTACTGACGCTGAGGTGCGAAAGCGTGGGGAGCAAACAGG
## 836                                                          ACGGAGGGTGCAAGCGTTACTCGGAATCACTGGGCGTAAAGAGCGTGTAGGCGGATTGATAAGTTTGAAGTGAAATCCTATAGCTTAACTATAGAACTGCTTTGAAAACTGTTAATCTAGAATGTGGGAGAGGTAGATGGAATTTCTGGTGTAGGGGTAAAATCCGTAGAGATCAGAAGGAATACCGATTGCGAAGGCGATCTACTGGAACATTATTGACGCTGAGATGCGAAAGCGTGGGGAGCAAACAGG
## 837                                                          ACGTAGGTGGCAAGCGTTGTCCGGATTTACTGGGCGTAAAGGATGCGTAGGCGGATTTTTAAGTCAGATGTGAAATACCCGAGCTTAACTTGGGTGCTGCATTTGAAACTGGAAGTCTAGAGTGCGGGAGAGGAGAGTGGAATTCCTAGTGTAGCGGTGAAATGCGTAGAGATTAGGAAGAACACCAGTGGCGAAGGCGACTCTCTGGACCGTAACTGACGCTGAGGCATGAAAGCGTGGGTAGCAAACAGG
## 839                                                          ACATAGGGTGCAAGCGTTGTCCGGAATTATTGGGCGTAAAGAGCTCGTAGGTGGTCGATCACGTCGGATGTGAAAATCTGAGGCTTAACCTCAGACCTGCATTCGATACGGGTTGACTAGAGTGTGGTAGGGGAGACTGGAATTCCTGGTGTAGCGGTGGAATGCGCAGATATCAGGAGGAACACCGATGGCGAAGGCAGGTCTCTGGGCCATTACTGACACTGAGGAGCGAAAGCGTGGGGAGCGAACAGG
## 840                                                          ACGTAGGGTGCGAGCGTTAATCGGAATTACTGGGCGTAAAGGGTGCGCAGGCGGCTATGTAAGTCAGATGTGAAATCCCCGGGCTTAACCTGGGAATTGCGTTTGAAACTACATGACTAGAGTGTAACAGAGGGAAGTGGAATTCCATGTGTAGCAGTGAAATGCGTAGAGATATGGAGGAACATCGATGGCGAAGGCAGCTTCCTGGGTTAACACTGACGCTCATGCACGAAAGCGTGGGGAGCAAACAGG
## 843                                                           ACAGAGGGTGCAAGCGTTATTCGGAATTACTGGGCGTAAAGCGCGCGCGGGCTGTCTAGCAAGTCTGATGTGAAATCCCCTGCAAAAACAGGGAAGTGCATTAGATACTACTAGACTAGAGTATAGGAGGGGAGAGTGGAATTCCTGGTGTAGAGGTGAAATTCGCAGATATCAGGAGGAACAACGGTGGCGAAGGCGACTCTCTGGACTATTACTGACGCTGAGGCGCGAAAGCGTGGGGAGCAAACAGG
## 844                                                          ACGTAGGGTGCGAGCGTTAATCGGAATTACTGGGCGTAAAGCGTGCGCAGGCGGTTTTGTAAGTCAGATGTGAAATCCCCGGGCTCAACCTGGGAACTGCATTTGAGACTGCAAGGCTAGAGTGTAGCAGAGGGGGGTAGAATTCCACGTGTAGCAGTGAAATGCGTAGAGATGTGGAGGAATACCGATGGCGAAGGCAGCCCCCTGGGTTACTACTGACGCTCATGCACGAAAGCGTGGGGAGCAAACAGG
## 845                                                          ACGAAGGTGGCAAGCGTTACTCGGAATTACTAGGTGTAAAGCGCAGGTAGGTGGTTAGATTAGTCTGTAATGAAATCTCCCGGCTTAACTGGGAGAGGTTTATGGATACTGTCTAGCTCGAGTGTGGGAGAGGGTGCTGGAATTCCCGGTGTAGCGGTGAAATGCGTAGATATCGGGAGGAACACCAATGGCGAAAGCAGGCACCTGGCCCAATACTGACACTGAGCTGCGAAAGCTAGGGGAGCAAACAGG
## 846                                                          ACGAGGGGTCCAAGCGTTGTTCGGAATTATTGGGCGTAAAGCGGGTGTAGGTGGCTCTGTAAGTCAGGAGTGAAAGCCCCGAGCTTAACTTGGGAAGTGCTTTTGATACTGCAGAGCTTGAGTGTGGGAGAGGATCGTGGAATTCCAGGTGTAGTGGTGAAATACGTAGATATCTGGAGGAACACCGGTGGCGAAGGCGGCGATCTGGCCCAACACTGACACTGAGACCCGAAAGCGTGGGGATCAAACAGG
## 847                                                          ACAGAGACCTCAAACGTTATCCGGATTTATTGGGCGTAAAGGGTCCGCAGGTGGTTTGAAAAGTCCGTGGTTAAACCCCGATGCTTAACATCGGGACTGCTGTGGATACTGTCAAACTTGAGGCTGGGAGAGGCAAGCGGAACTATCGGTGTAGTCGTAATAAGCGCTGATATCGATGGGAACACCAAAGGCGAAGGCAGCTTGCTGGAACATGCCTGACACTCAGGGACGAAAGCGTGGGGAGCGAAGGGG
## 848                                                          ACGAGGGGTGCAAGCGTTGTTCGGAATTACTGGGCGTAAAGCGCGTGTAGGTGGTCAAGCAAGTCGGATGTGAAAGCCCTCGGCTTAACCGAGGAAGTGCGTCCGAAACTATTTGGCTTGAGTGTGGGAGAGGAGGGTGGAATTCCCAGTGTAGAGGTGAAATTCGTAGATATTGGGAGGAACACCGGTGACGAAGGTGACTCTCTGGACCACAACTGACACTAAGACGCGAAAGCGTGGGTAGCAAACAGG
## 849                                                          ACAGAGACTGCAAGCGTTATTCGGATTCACTGGGCGTAAAGGGTGCGCAGGCGGTTGGGTGTGTCAGATGTGAAATCCCGAGGCTTAACCTCGGAACTGCGTCTGAAACTACTCGACTAGAGTACTGGAGAGGGAAACGGAATTCACGGTGTAGCAGTGAAATGCGTAGATATCGTGAGGAACACCAGAGGCGAAGGCGGTTTCCTGGACAGTTACTGACGCTCAGGCACGAAAGCATGGGGAGCAAAAGGG
## 850                                                          ACGGGGGGTGCAAGCGTTACTCGGAATCACTGGGCGTAAAGAGCATGTAGGCGGATTAATAAGTTTGAAGTGAAATCCTATAGCTTAACTATAGAACTGCTTTGAAAACTGTTAATCTAGAATGTGGGAGAGGTAGATGGAATTTCTGGTGTAGGGGTAAAATCCGTAGAGATCAGAAGGAATACCGATTGCGAAGGCGATCTACTGGAACATTATTGACGCTGAGGCATGAAAGCGTGGGGAGCAAACAGG
## 851                                                          ACGTAGGGTGCAAGCGTTGTCCGGAATTATTGGGCGTAAAGAGCTCGTAGGCGGTTGGTCGCGTCTGCTGTGAAAACCCGAGGCTCAACCTCGGGCCTGCAGTGGGTACGGGCCAACTAGAGTGCGGTAGGGGAGATCGGAATTCCTGGTGTAGCGGTGGAATGCGCAGATATCAGGAGGAACACCAATGGCGAAGGCAGATCTCTGGGCCGTAACTGACGCTGAGGAGCGAAAGCGTGGGGAGCGAACAGG
## 852                                                          ACGGAGGGTGCAAGCGTTATCCGGAATCACTGGGTTTAAAGGGTGCGTAGGCGGCTTAGTAAGTCAGTGGTGAAAGGCTGTGGCTTAACCATGGAATTGCCATTGATACTGCAGAGCTTGAATGAGGTTGAGGTTGGCGGAATGTGACATGTAGCGGTGAAATGCTTAGATATGTCATGGAACACCGATTGCGAAGGCAGCTGACTGGACCTATATTGACGCTGAGGCACGAAAGCGTGGGGAGCGAACAGG
## 853                                                          ACAGGGGATGCAAGTGTTATCCGGATTCATTGGGCGTAAAGCGTCTGCAGGTTGATATTTAAGTCTTTTGTTAAAACTTCGGGCTTAACCCGAAATCTGCAAAAGAAACTATGTATCTTGAGTATGGTAGAGGTAAAGGGAATTTCCAGTGGAGCGGTGAAATGCGTAGAGATTGGAAAGAACACCAACAGCGAAGGCACTTTACTGGGCCAGTACTGACACTGAGAGACGAAAGCTAGGGGAGCAAACGGG
## 854                                                          ACGAAGGATCCAAGCGTTGTCCGGATTTACTGGGTTTAAAGGGTGCGTAGGCGGGCTATTAAGTCAGTGGTGAAAGCTTGCAGCTTAACTGTAAAATTGCCATTGAAACTGATAGCCTCGAATATGGTTGAGGTGGTTGGAATGTATCATGTAGCGGTGAAATGCTTAGATATGATACAGAACACCGATTGCGAAGGCAGATCACTAAGCCATTATTGACGCTGAGGCACGAAAGCGTGGGGATCAAACAGG
## 857                                                          ACAGAGGTGGCGAGCGTTGTTCGGATTTACTGGGCGTAAAGGGCGCGTAGGCGGCGCGGTGTGTCGGGTGTGAAATCCATGGGCTCAACCCATGAAGTGCGCCCGAAACTGCCGCGCTCGAGTTCGGGAGGGGGGATCGGAATGCAGGGTGTAGCGGTGAAATGCGTTGATATCCTGCAGAACACCGGAGGCGAAGGCGGATCCCTGGAACGACACTGACGCTGAGGCGCGAAAGCAGGGGGAGCAAACAGG
## 858                                                          ACGTAGGGTGCAAGCGTTAATCGGAATTACTGGGCGTAAAGCGTGCGCAGGCGGTTCCATAAGACAGATGTGAAATCCCCGGGCTCAACCTGGGAACTGCATTTGTGACTGTGGGGCTAGAGTACGGTAGAGGGGGATGGAATTCCGCGTGTAGCAGTGAAATGCGTAGATATGCGGAGGAACACCGATGGCGAAGGCAATCCCCTGGACCTGTACTGACGCTCATGCACGAAAGCGTGGGGAGCAAACAGG
## 859                                                          ACGTAGGATGCAAGCGTTGTCCGGATTTATTGGGCGTAAAGAGTTCGTAGGCGGTTATTTAAGTCTGGTGTTAAATACTGTGGCCCAACCACAGAACTGCATTGGATACTGGATGACTTGAGTGCGGTAGAGGCGAGTGGAATTCCCAGTGTAGCGGTGAAATGCGTAGATATTGGGAAGAACACCAGTGGCGTAAGCGACTCGCTGGCCCGTAACTGACGCTGAGGAACGAAAGCCAGGGGAGCGAATGGG
## 860                                                          ACGTAGGTGGCAAGCGTTGTCCGGATTTATTGGGTTTAAAGGGTGCGTAGGCGGTCCATTAAGTCAGTGGTGAAATACGGCAGCTTAACTGTCGAGGTGCCATTGATACTGGAGGACTTGAGTACAGACGAGGTAGGCGGAATTGACGGTGTAGCGGTGAAATGCTTAGATATCGTCAAGAACACCGATAGCGAAGGCAGCTTACTAGACTGTAACTGACGCTGAGGCACGAAAGTGTGGGGATCAAACAGG
## 864                                                          ACAGAGGGTGCAAGCGTTGCTCGGAATTATTGGGCGTAAAGGGCAGGTAGGCGGTCTTATTTGTCAGAGGTGAAAGCCTTGGGCTTAACCCAAGAAGTGCCTCTGAAACGGTAAGACTAGAGTACTAGATAGGGTCGCGGAATTCCTGGTGTAGCGGTGAAATGCGTAGAGATCAGGAGGAACATCGGAGGCGAAGGCGGCGACCTGGAGAGTGACTGACGCTCAACTGCGAAAGCGTGGGTAGCAAACAGG
## 865                                                          ACGAAGGTGGCTAGCGTTGTTCGGATTTACTGGGCGTAAAGGGAGCGTAGGCGGTGAAACAAGTCAGATGTGAAAGCCCAAGGCTTAACCTTGGAATAGCATTTGAAACTGTTTTGCTAGAGTATGGAAGAGGATAGCGGAATTCCTAGTGTAGAGGTGAAATTCGTAGATATTAGGAAGAACACCAGTGGCGAAGGCGGCTATCTGGTCCATTACTGACGCTGAAGTTCGAAAGTGTGGGGATCAAACAGG
## 866                                                          ACGAAGGGGGCTAGCGTTGTTCGGAATTACTGGGCGTAAAGCGCGCGCAGGCGGCTATCCAAGTCAGTGGTGAAAGCCCGGAGCTCAACTCCGGAACTGCCATTGAAACTGTTTAGCTTGAGGACGAGAGAGGTGAGTGGAATTCCCAGTGTAGAGGTGAAATTCGTAGATATTGGGAAGAACACCGGTGGCGAAGGCGGCTCACTGGCTCGTATCTGACGCTCAGGCGCGATAGCGTGGGGATCAAACAGG
## 868                                                           ACGTAGGTGGCAAGCGTTGTCCGGAATTATTGGGCGTAAAGGGAGCGCAGGCGGGAAGGTAAGTCGGTCTTAAAAGTGCGGGGCTCAACCCCGTGATGGGATCGAAACTATCTTTCTTGAGTGCAGGAGAGGAAAGTGGAATTCCTAGTGTAGCGGTGAAATGCGTAGATATTAGGAGGAACACCAGTGGCGAAGGCGACTTTCTGGACTGTAACTGACGCTGAGGCTCGAAAGCCAGGGGAGCGAACGGG
## 869                                                          ACGAGGGACCCTAGCGTTGTTCGGAATCATTGGGCGTAAAGCGGGTGCAGGTGGCTTTGTAAGTCAGGTGTGAAAGCCCAGGGCTCAACCCTGGAAGTGCATTTGATACTGCGAAGCTTGAGTGCTGGAGAGGTTACTAGAATTCCAGGTGTAGTGGTGAAATACGTAGATATCTGGAGGAATACCGGAGGCGAAGGCGGGTAACTGGCCAGACACTGACACTCAGACCCGAAAGCGTGGGGATCAAACAGG
## 871                                                           ACGTAGGTGGCAAGCGTTGTCCGGAATTATTGGGCGTAAAGGGCGTGCAGGCGGCCAGGCAAGTCTGTCTTAAAAGTGCGGGGCTTAACCCCGTGATGGGATGGAAACTGTCAGGCTAGAGTGTCGGAGAGGAAAGCGGAATTCCTAGTGTAGCGGTGAAATGCGTAGATATTAGGAGGAACACCAGTGGCGAAAGCGGCTTTCTGGACGACAACTGACGCTGAGGCGCGAAAGCCAGGGGAGCAAACGGG
## 872                                                          ACGGAGGATCCAAGCGTTATCCGGAATCATTGGGTTTAAAGGGTCCGTAGGCGGTTTTATAAGTCAGTGGTGAAATCCGGCAGCTCAACTGTCGAACTGCCATTGATACTGTAGAACTTGAATTACTTGGAAGTAACTAGAATATGTAGTGTAGCGGTGAAATGCTTAGAGATTACATGGAATACCAATTGCGAAGGCAGGTTACTACGAGTATATTGACGCTGATGGACGAAAGCGTGGGGAGCGAACAGG
## 875                                                          ACGTAGGATGCAAGCGTTGTCCGGAATCATTGGGCGTAAAGCGTTCGTAGGCGGCATATTAAGTCTGATGTTAAAGCCCGGGGCTCAACTCCGGTTCGGCATTGGATACTGATAAGCTAGAGTGCAATAGAGGTAGGGGGAATTCCTAGTGTAGCGGTGAAATGCGTAGATATTAGGAGGAACACCGGTGGCGAAAGCGCCCTACTGGGTTGTAACTGACGCTGAGGAACGAAAGCCAGGGTAGCAAATGGG
## 876                                                          ACGGAGGATGCAAGTGTTATCCGGAATCACTGGGCGTAAAGCGTCTGTAGGTGGTCTAATAAGTCAACTGTTAAATCTTGAGGCTCAACTTCAAAACCGCAGTCGAAACTATTAGACTAGAGTATAGTAGAGGTAAAGGGAATTTCCAGTGGAGCGGTGAAATGCGTAGATATTGGAAAGAACACCGATGGCGAAAGCACTTTACTGGGCTATTACTAACACTCAGAGACGAAAGCTAGGGTAGCAAATGGG
## 877                                                          ACAGAGACTGCAAGCGTTACTCGGATTCACTGGGCGTAAAGGGAGCGCAGGCGGACTGGTGTGTCAGGCGTGAAATCCCGGGGCTCAACCCCGGGGCTGCGTTTGAAACTACCAGTCTAGAGATTCGGAGGGGTAAGCGGAATTCGTGGTGGAGCAGTGAAATGCGTAGATATCACGAGGAACACCGACGGCGAAGGCAGCTTACTGGACGAAATCTGACGCTCAGGCTCGAAAGCATGGGGAGCAAAAGGG
## 881                                                          ACGTAGGACCCGAGCGTTGTCCGGATTTACTGGGTATAAAGGGTGCGTAGGCGGCCTTGTGCGTCAGAGGTGAAATATCCGGGCTTAACCCGGAGGGTGCCTTTGATACGGCGAGGCTTGAGTGCGAGAGAGGATGATGGAATTCCTGGTGTAGCGGTGAAATGCGTAGATATCAGGAGGAACACCGGTGGCGAAGGCGGTCATCTGGCTCGTAACTGACGCTGAGGCACGAAAGCGCGGGGATCAAACAGG
## 882                                                          ACGTAGGGTGCAAGCGTTAATCGGAATTACTGGGCGTAAAGCGTGCGCAGGCGGTTATATAAGACAGATGTGAAATCCCCGGGCTCAACCTGGGAACTGCATTTGTGACTGTATAGCTAGAGTACGGTAGAGGGGGATGGAATTCCGCGTGTAGCAGTGAAATGCGTAGATATGCGGAGGAACACCGATGGCGAAGGCAATCCCCTGGGCCTGCACTGACGCTCATGCACGAAAGCGTGGGGAGCAAACAGG
## 883                                                          ACGGAGGGTGCAAGCGTTAATCGGAATTACTGGGCGTAAAGGGTTCGTAGGTGGTTAATTAAGTTATCTGTGAAATCCCCGGGCTCAACCTGGGCAGGTCAGATAATACTGGTTAACTCGAGTATGGGAGAGGGTAGTGGAATTTCCGGTGTAGCGGTGAAATGCGTAGAGATCGGAAGGAACACCAGTGGCGAAGGCGGCTACCTGGCCTAATACTGACACTGAGGAACGAAAGCGTGGGGAGCAAACAGG
## 884                                                          ACAGAGGGTGCAAGCGTTGCTCGGAATTATTGGGCGTAAAGGGCAAGTAGGTGGTCTGATTTGTCAGGGGTGAAAGCCTTGGGCTTAACCTGAGAAGTGCCCCTGAAACGGTCAGACTTGAGTCCTGGAGAGGGTCGTGGAATTCCCGGTGTAGCGGTGAAATGCGTAGAGATCGGGAGGAACACCTGAGGCGAAGGCGGCGACCTGGACAGGAACTGACACTCAACTGCGAAAGCATGGGTAGCAAACAGG
## 885                                                          ACAGAGGGTGCAAGCGTTAATCGGAATTACTGGGCGTAAAGCGCGCGTAGGCGGCTGATTAAGTCGGATGTGAAATCCCCGGGCTTAACCTGGGAACTGCGTTCGATACTGATCGGCTAGAGTATGAGAGAGGGAGGTAGAATTCCACGTGTAGCGGTGAAATGCGTAGATATGTGGAGGAATACCGGTGGCGAAGGCGGCCTCCTGGCTTAATACTGACGCTGAGGTGCGAAAGCGTGGGGAGCAAACAGG
## 886                                                          ACGTAGGGGGCAAGCGTTATCCGGATTTACTGGGTGTAAAGGGAGTGTAGACGGTCTGGTAAGTCAGATGTGAAAGCCCGGGGCTCAACCCCGGGACTGCATTTGAAACTATCAGACTAGAGTGCAGGAGAGGTAAGTGGAATTCCTAGTGTAGCGGTGAAATGCGTAGATATTAGGAGGAACACCAGTGGCGAAGGCGGCTTACTGGACTGTAACTGACGTTGAGGCTCGAAAGCGTGGGGAGCAAACAGG
## 887                                                          ACGAATGTGGCAAGCGTTGTTCGGAATCACTGGGCGTAAAGAATGCGTAGGCGGCTTGGCAAGTCAGAAGTGAAATCCCAGGGCTTAACCCTGGAACTGCTTTTGAAACTGTTAGGCTAGTGTCCCGGAGGGGTTGATGGAATTCCGAGTGTAGAGGTGAAATTCGCAGATATTCGGAGGAACACCAGTGGCGAAAGCGATCAACTGGACGGGTACAGACGCTGAGGCATGAAAGTGTGGGGATCAAACAGG
## 888                                                          ACGTAGGGTGCGAGCGTTAATCGGAATTACTGGGCGTAAAGCGTGCGCAGGCGGCTTTGTAAGACAGGTGTGAAATCCCCGGGCTTAACCTGGGAACTGCGCTTGTGACTGCAAGGCTCGAGTGCGGCAGAGGGGGGTGGAATTCCACGTGTAGCAGTGAAATGCGTAGAGATGTGGAGGAACACCGATGGCGAAGGCAGCCCCCTGGGTCGACACTGACGCTCATGCACGAAAGCGTGGGTAGCAAACAGG
## 890                                                          ACGGAAAGTGCGAGCGTTGTTCGGATTGACTGGGCGTAAAGAGCACGTAGGCGGGTCTGTAAGTCGATTGTGAAATCCCCGGGCTCAACCCGGGAACTGCAGTCGATACTGTGGATCTTGAATGTTCGAGGGGTTGGTGGAATTCCAGGTGTAGAAGTGAAATTCGTAGATATCTGGAGGAACACCGGAGGCGAAGGCGGCCGACTGGCGAAACATTGACGCTGAGGTGCGAAAGCGTGGGGAGCAAACAGG
## 891                                                          ACGTGAGGTGCAAACATTACCCGGAATCATTGGGCGTAAAGCGTCCGCAGGCGGCGCAGTAAGTCAGGTATTAAATCTTAAGGCTCAACTTTAAGGCTGTACTTGAAACTACTGTGCTAGAGTTTTGGAGAGGTGAGCGGAATTCTACATGTAGGGGTAAAATCCGTAGATATGTAGAGGAACACCAAAAGCGAAGGCAGCTCACTGGCCAAATACTGACGCTCATGGACGAAAGCGTGGGTAGCAAACGGG
## 892                                                          ACGAAGGGTGCAAGCGTTATTCGGAATGACTGGGTGTAAAGGGCGTGTAGGCGGTTGATTAAGTTAATGGTAAAAGGCCATAGCCTAACTGTGGTATAGCCGTTAAAACTAATCAGCTAGAGTTAGGAAGAGAGAAGTGGAATTCTCGGAGTAGCGGTAAAATGCGTAGATCTCGAGAGGAACACCGATGGCGAAGGCAGCTTCTTGGTCCTATACTGACGCTGAGGCGCGAAAGCGTGGGGAGCAAACAGG
## 895                                                         ACGTAGGCTCCAAGCGTTGTCCGGATTTATTGGGCGTAAAGAGCTCGTAGGCGGTTCAGCAAGTCGGGTGTGAAAACTATGGGCTTAACCCATAGCCTGCACCCGAAACTGCTGTGACTAGAGTTTGGTAGGGGAGCGGGGAATTCCTGGTGTAGCGGTGAAATGCGCAGATATCAGGAGGAACACCGGTGGCGAAGGCGCCGCTCTGGGCCAATACTGACGCTGAGGAGCGAAAGCGTGGGTAGCAAACAGG
## 896                                                           ACGTATGTCGCAAGCGTTATCCGGATTTATTGGGCGTAAAGCGCGTCTAGGCGGAAAAATAAGTCTGATGTTAAAATGCGGGGCTCAACTCCGTATTGCGTTGGAAACTGTTTTTCTAGAGTACTGGAGAGGTGGGCGGAACTACAAGTGTAGAGGTGAAATTCGTAGATATTTGTAGGAATGCCGATGGAGAAGTCAGCTCACTGGACAGATACTGACGCTAAAGCGCGAAAGCGTGGGGAGCAAACAGG
## 897                                                          ACGAAGGTCCCGAGCGTTGTTCGGAATCACTGGGCGTAAAGGGAGCGTAGGCGGCGCGGTAAGTCAGATGTGAAATCCCGGGGCTCAACCCCGGAACTGCATCCGATACTGCCGTGCTAGAGTAATGGAGAGGTAGCTGGAATTATCGGTGTAGCAGTGAAATGCGTAGATATCGATAGGAACACTCGTGGCGAAAGCGAGCTACTGGACATTTACTGACGCTGAGGCTCGAAGGCTAGGGGAGCGAAAGGG
## 898                                                          ACAGAGGGTGCAAGCGTTAATCGGAATTACTGGGCGTAAAGCGCGCGTAGGCGGTTCGTTAAGTCCGTTGTGAAATCCCTGGGCTCAACCTGGGAATGGCGATGGATACTGGCGAGCTAGAGTATGAGAGAGGTGGGTAGAATTCCAGGTGTAGCGGTGAAATGCGTAGATATCTGGAGGAATACCGGTGGCGAAGGCGGCCCACTGGCTTAATACTGACGCTGAGGTGCGAAAGCGTGGGGAGCAAACAGG
## 899                                                          ACGGAGGGGGCGAGCGTTGTTCGGAATCACTGGGCGTAAAGCGTGCGTAGGCGGTTATACAAGTCAGGAGTGAAATCCCAGGGCTCAACCCTGGAATTGCTTTTGAGACTGTATGGCTAGAGATCTGGAGAGGTTGGGGGAATTCCGAGTGTAGCAGTGAAATGCGCAGATATTCGGAGGAACACCAGTGGCGTAGGCGCCCAACTGGCCAGATACTGACGCTGAGGCACGAAAGCGTGGGGAGCAAACAGG
## 900                                                          ACAGAGGGTGCAAGCGTTGCTCGGAATTATTGGGCGTAAAGGGCAGGTAGGTGGTCTCGTTTGTCTGTGGTGAAATCCCTGGGCTCAACCCAGGAAGTGCCTCAGAAACGGCGGGACTAGAGTGCTAGAGAGGGTCGTGGAATTCCCGGTGTAGCGGTGAAATGCGTAGAGATCGGGAGGAACACCAGAGGCGAAGGCGGCGACCTGGATAGCAACTGACACTCAACTGCGAAAGCGTGGGGAGCAAACAGG
## 902                                                          ACGGGGGATGCAAGTGTTATCCGGAATCACTGGGCGTAAAGCGTCTGTAGGTGGTTAAATAAGTCAACTGTTAAATCTTGAGGCTCAACCTCAAAATCGCAGTCGAAACTGTTTGACTAGAGTATAGTAGGGGTAAAGGGAATTTCCAGTGGAGCGGTGAAATGCGTAGATATTGGAAAGAACACCGATGGCGAAGGCACTTTACTGGGCTATTACTAACACTGAGAGACGAAAGCTAGGGTAGCAAATGGG
## 903                                                         ACGTAGGATCCAAGCGTTATCCGGAATTACTGGGCGTAAAGCGTCTGTAGGCGGCTTTTTAAGTCGGGTGCGAAATCTTGCGGCTCAACCGTATAGACTGTGCTCGAAACTATGAAGCTAGAGGTAGGTAGAGGCAAGTGGAATTTCTGGTGTAGGAGTGACATCCGTAGATATCAGAAGGAACACCAATGGCGAAGGCAGCTTGCTGGGCCTTACCTGACGCTCAGAGACGAAAGCGTGGGGAGCGAACGGG
## 904                                                           ACGTAGGTGGCAAGCGTTGTCCGGAATTATTGGGCGTAAAGCGCGCGCAGGTGGGATCTTAAGTCCATTTTGAAAGTTCGGAGCTCAACTCCGTGATGGGATGGAAACTGGGATTCTTGAGTACCGGAGAGGAAAGCGGAATTCCCCGTGTAGCGGTGAAATGCGTAGATATGGGGAGGAACACCAGTGGCGAAGGCGGCTTTCTGGACGGTGTCTGACACTGAGGCGCGAAAGCCAGGGGAGCGAACGGG
## 905                                                          ACGTAGGTGGCAAGCGTTGTCCGGAATTACTGGGTGTAAAGGGCGTGTAGGCGGGAATGCAAGTCAGATGTGAAATTCCGGGGCTCAACCCCGGCGCTGCATCTGAAACTGTGTTTCTTGAGTGCTGGAGAGGAAAGCGGAATTCCTAGTGTAGCGGTGAAATGCGTAGATATTAGGAGGAACACCAGTGGCGAAGGCGGCTTTCTGGACAGTAACTGACGCTGAGGCGCGAAAGCGTGGGGAGCAAACAGG
## 906                                                          ACGAACCGTCCAAACGTTATTCGGAATCACTGGGCTTAAAGGGTGCGTAGGCGGCGCGGAAAGTTGGGTGTGAAATCCCTCGGCTCAACCGAGGAACTGCGCCCAAAACTACCGTGCTCGAGGGAGATAGAGGTGAGCGGAACTTAGGGTGGAGCGGTGAAATGCGTTGATATCCTAAGGAACACCGGTGGCGAAAGCGGCTCACTGGATCTCTTCTGACGCTGAGGCACGAAAGCTAGGGTAGCGAACGGG
## 908                                                          ACAGAGACTGCAAGCGTTATTCGGATTCACTGGGCGTAAAGGGTGCGCAGGCGGCAATGTGTGTGAGACGTGAAAGCCCGGAGCTTAACTCCGGAATTGCGCCTCAAACTACATTGCTAGAGCATTGGAGAGGGTAGCAGAATTCACGGTGTAGCAGTGAAATGCGTAGATATCGTGAGGAATACCAGAGGCGAAGGCGGCTACCTGGACAATTGCTGACGCTCAGGCACGAAAGCGTGGGGAGCAAAAGGG
## 909                                                          ACAGAGGGTGCGAGCGTTAATCGGAATTACTGGGCGTAAAGCGCGCGTAGGCGGTTTGTTAAGTCGGATGTGAAATCCCCGGGCTCAACCTGGGAACTGCATTCGATACTGGCAGACTAGAGTATGGGAGAGGATGGTAGAATTCCAGGTGTAGCGGTGAAATGCGTAGAGATCTGGAGGAATACCGATGGCGAAGGCAGCCATCTGGCCTAATACTGACGCTGAGGTGCGAAAGCATGGGGAGCAAACAGG
## 910                                                          ACGGAGGGTGCAAGCGTTGTTCGGAATCATTGGGCGTAAAGGGCGTGTAGGTGGTCTGCCAAGTCAGGTGTGAAATCCCGCGGCTCAACCGCGGAACTGCACTTGAAACTGGTGGACTCGAGTGTCGGAGGGGGCAGCGGAATTCCCGGTGTAGAGGTGAAATTCGTAGATATCGGGAGGAACACCAGTGGCGAAGGCGGCTGCCTGGCCGATGACTGACACTGAGACGCGAAAGCGTGGGGAGCAAACAGG
## 913                                                          ACAGGGGGTGCTAGCGTTGTTCGGAATTACTGGGCGTAAAGGGCGCGTAGGCGGTCTTGCAAGTCAGAGGTGAAATCCCGGAGCTTAACTCCGGAATTGCTTTTGATACTGCAAGGCTAGAGTATGTTAGAGGAAATCGGAATTCCTAGTGTAGAAGTGAAATTCGTAGATATTAGGAAGAACACCGGTGGCGAAGGCGGATTTCTGGGACATAACTGACGCTGAGGCGCGAAAGCGTGGGGATCAAACAGG
## 914                                                          ACGTAGGGTGCAAGCGTTATCCGGAATCATTGGGCGTAAAGCGTTCGTAGGTGGTTTAGAAAGTTTGTGGTTAAATCCGATCGCTCAACGATCGAATCGCTACAAAAACTCCTAAACTAGAGAATGATAGAGGCAACTGGAATTTCGCATGTAGGGGTAAAATCCGTTGATATGCGAAGGAACACCAAAAGCGAAGGCAGGTTGCTGGGTCATTTCTGACACTCAAGAACGAAAGCGTGGGGAGCAAACAGG
## 915                                                        ACGTACTCCCTGAGTGGTATCCACTGATATTGGGCCTAAAGCGTTCGTAGCCCGTGAAGAAAGTCCTTGGTGAAAGTGGCACGCTCAACGTGTCAAATGGCTAAGGATACTACTTCGCTTTGAGGTGATACGAGGTCAAGAGAATTGCAGGGGTAACGGTGAAATGTTGTGATCCTTGCAGGACTACCAGTAGCGAAGGCGCTTGACCAGGATCATCCTGACGGTGAGGAACGAAAGCTGGGGGAGCGACCCGG
## 916                                                          ACGTAGGGTGCAAGCGTTAATCGGAATTACTGGGCGTAAAGCGTGCGCAGGCGGATATGTAAGTTAGAAGTGAAATCCCCGGGCTCAACCTGGGAATGGCTTTTAAGACTGCGTATCTAGAGTTTGTCAGAGGGGGGTGGAATTCCAAGTGTAGCAGTGAAATGCGTAGAGATTTGGAGGAACACCAATGGCGAAGGCAACCCCCTGGGATAAAACTGACGCTCATGCACGAAAGCGTGGGTAGCAAACAGG
## 919                                                          ACGAGTGGTGCAAGCGTTATCCGGAATTATTGGGCGTAAAGGATGTGTAGGTGGCAATGTTAGTCGAGTGTTTAACCCATGGGCTCAACCTGTGGATCGCATTCGAAACGGCATAGCTAGAAGGTATTAGGGGTATGCGGAACTATATGTGTAGGGGTGAAATCCGTTGATATATATGGGAACACCAAAAGCGAAGGCAGCATACTGGAATATTCTTGACACTGAGACATGAAAGCGTGGGTAGCGAATGGG
## 920                                                          ACAGAGGGTGCGAGCGTTGTCCGGAATCACTGGGCGTAAAGGGCGCGTAGGTGGCTTGGTAAGCGTGTGGTGAAAGTCCGGGGCTCAACCCCGGATCTGCCGTGCGAACTGCTGAGCTTGAGCATTGTAGAGGCAGGTGGAATTCCGGGTGTAGCGGTGGAATGCGTAGAGATCCGGAAGAACACCAGTGGCGAAGGCGGCCTGCTGGGCAATAGCTGACACTGAGGCGCGACAGCGTGGGGAGCAAACAGG
## 921                                                          ACAGAGAGGGCAAGCGTTGTCCGGAATGACTGGGCGTAAAGAGCGCGTAGGCGGTTTTTTAAGTCGGATGTGAAATCCCTGGGCTTAACCTAGGAACTGCATCCGATACTGGAGAACTAGAGGACGAGAGAGGAAAGTGGAATGACGAGTGTAGAGGTGAAATTCGTAGATATTCGTCGGAACACCAGAGGCGAAGGCGGCTTTCTGGCTCGTACCTGACGCTGAGGCGCGAAAGCGTGGGGAGCAAACAGG
## 922                                                          ACGAAGGATCCAAGCGTTATCCGGATTCATTGGGTTTAAAGGGTGCGTAGGCGGGTTTGTAAGTCAGTGGTGAAAGCCGGTCGCTCAACGATCGAATTGCCATTGATACTGCGGACCTAGAATATAGATGATGTTGGCGGAATATGACATGTAGCGGTGAAATGCATAGATATGTCATAGAACACCTATTGCGAAGGCAGCTGACAAAACTATTATTGACGCTGATGCACGAAAGTGCGGGGATCAAACAGG
## 923                                                          ACGAATGGGGCAAGCGTTGTTCGGAATCACTGGGCTTAAAGCGTGCGTAGGCGGTCTTGCAAGTCAGAAGTGAAAGCCTGGGGCTCAACCCCAGAATTGCTTTTGAAACTGCATGACTAGAATACCGGAGAGGTTGGGAGAATTCCTAGTGTAGAGGTGAAATTCGCAGATATTAGGAGGAATACCAGTGGCGTAGGCGCCCAACTGGACGGTTATTGACGCTCAGGCACGAAAGCGTGGGGAGCAAACAGG
## 924                                                          ACGGAGGATGCGAGCGTTATCCGGATTTATTGGGTTTAAAGGGTGCGTAGGTGGTGATTTAAGTCAGCGGTGAAAGTTTGTGGCTCAACCATAAAATTGCCGTTGAAACTGGGTTACTTGAGTGTGTTTGAGGTAGGCGGAATGCGTGGTGTAGCGGTGAAATGCATAGATATCACGCAGAACTCCGATTGCGAAGGCAGCTTACTAAACCATAACTGACACTGAAGCACGAAAGCGTGGGGATCAAACAGG
## 925                                                          ACGAGGGATCCTAGCGTTGTTCGGAATCATTGGGCGTAAAGCGGGTGTAGGTGGCTCTGTAAGTCAAGTGTGAAAGCCCCGGGCTCAACCTGGGAAGTGCATTTGATACTGCGGAGCTTGAGTGCTAGAGAGGTCATTAGAATACCTGGTGTAGTGGTGAAATACGTAGATATCAGGTGGAATACCAGAGGCGAAGGCGGATGGCTGGCTAGACACTGACACTCAGACCCGAAAGCGTGGGGATCAAACAGG
## 927                                                          ACGGAGGATCCAAGCGTTATCCGGAATCATTGGGTTTAAAGGGTCCGTAGGCGGTTTTATAAGTCAGTGGTGAAATCTGGTCGCTCAACGATCAAACGGCCATTGATACTGTAGAACTTGAATTACTTGGAAGTAACTAGAATATGTAGTGTAGCGGTGAAATGCTTAGAGATTACATGGAATACCAATTGCGAAGGCAGGTTACTACGAGTATATTGACGCTGATGGACGAAAGCGTGGGGAGCGAACAGG
## 928                                                          ACGTAGACCACAAGCGTTATCCGGATTTATTGGGCGTAAAGGGTGTGTAGGAGGTTGTGTGCGTCTTTGGTTAAATCCCACTGCCTAACAGTGGACCTGCCAGAGATACGACACGGCTAGAGGAAGTTAGAGGTTCATGGAGCGCACGGTGTAGGGGTGAAATCCGTTGATATCGTGCGAAACACCAAAGGCGAAGGCAATGAACTGGGACTCTCCTGACTCTGAAACACGAAAGCGTGGGGAGCAAAAAGG
## 929                                                          ACGAGGGATCCTAGCGTTGTTCGGAATCATTGGGCGTAAAGCGGGTGTAGGTGGCTACATAAGTCAGATGTGAAAGCCTTGGGCTTAACCCAAGAAGTGCATTTGATACTGTGTAGCTTGAGTGTGGTAGAGGCTACTAGAATTCCTGGTGTAGTGGTGAAATACGTAGATATCAGGAGGAATACCGGAGGCGAAGGCGGGTAGCTGGGCCAACACTGACACTGAGACCCGAAAGCGTGGGGATCAAACAGG
## 930                                                          ACGGGAGTGGCAAGCGTTATCCGGAATTATTGGGCGTAAAGCGTCCGCAGGCGGCCTTTTAAGTCTGTTGTTAAAGCGTGGAGCTTAACTCCATTTCAGCAATGGAAACTGGAAGGCTTGAGTGTGGTAGGGGCAGAGGGAATTCCCGGTGTAGCGGTGAAATGCGTAGATATCGGGAAGAACACCAGTGGCGAAGGCGCTCTGCTGGGCCATAACTGACGCTCATGGACGAAAGCCAGGGGAGCGAAAGGG
## 931                                                          ACGTAGGGTGCAAGCGTTAATCGGAATTACTGGGCGTAAAGCGTGCGCAGGCGGTTATATAAGTCAGATGTGAAATCCCCGGGCTCAACCTGGGAACTGCATTTGAGACTGTATAGCTAGAGTACGGCAGAGGGGGATGGAATTCCGCGTGTAGCAGTGAAATGCGTAGATATGCGGAGGAACACCGATGGCGAAGGCAATCCCCTGGACCTGTACTGACGCTCATGCACGAAAGCGTGGGGAGCAAACAGG
## 932                                                          ACAGAGGTCTCAAGCGTTGTTCGGAATCACTGGGCGTAAAGCGTGCGTAGGCTGTTTCGTAAGTCGTGTGTGAAAGGCGCGGGCTCAACCCGCGGACGGCACATGATACTGCGAGACTAGAGTAATGGAGGGGGAACCGGAATTCTCGGTGTAGCAGTGAAATGCGTAGATATCGAGAGGAACACTCGTGGCGAAGGCGGGTTCCTGGACATTAACTGACGCTGAGGCACGAAGGCCAGGGGAGCGAAAGGG
## 933                                                          ACGGGAGTGGCAAGCGTTATCCGGAATTATTGGGCGTAAAGCGTCCGCAGGCGGTCTTACAAGTCTGTCGTTAAAGCGTGGAGCTTAACTCCATTTCGGCGATGGAAACTGTAAGACTAGAGTGTGGTAGGGGCAGAGGGAATTCCCGGTGTAGCGGTGAAATGCGTAGATATCGGGAAGAACACCAGTGGCGAAGGCGCTCTGCTGGGCCATAACTGACGCTCATGGACGAAAGCCAGGGGAGCGAAAGGG
## 934                                                          ACGAAGGGTGCAAGCGTTAATCGGAATTACTGGGCGTAAAGCGCGCGTAGGCGGTTTGATAAGTTGGATGTGAAAGCCCCGGGCTCAACCTGGGAATTGCATCCAAGACTGTCAGGCTAGAGTACGGGAGAGGGGGGTAGAATTCCACGTGTAGCGGTGAAATGCGTAGAGATGTGGAGGAATACCGGTGGCGAAGGCGGCCCCCTGGATCGATACTGACGCTGAGGTGCGAAAGCGTGGGGAGCAAACAGG
## 936                                                          ACGGAGGGTGCAAGCGTTATCCGGATTCACTGGGTTTAAAGGGTGCGTAGGCGGGCAGGTAAGTCAGTGGTGAAATCTTCGAGCTTAACTCGGAAACTGCCGTTGATACTATCTGTCTTGAATTTAGTGGAGGTAAGCGGAATATGTCATGTAGCGGTGAAATGCTTAGATATGACATAGAACACCAATTGCGAAGGCAGCTTGCTACACTATTATTGACGCTGAGGCACGAAAGCGTGGGGATCAAACAGG
## 937                                                          ACGAAGGTGGCAAGCGTTACTCGGAATTACTAGGCGTAAAGGGCAGGTAGGTGGTTTTGTAAGTCTGTTGTGAAAGCTCCTGGCTTAACTGGGAGAGGTCAACGGATACTGCAGGACTTGAGTATAGGAGAGGTTACTGGAATTCCCGGTGTAGCGGTGAAATGCGCAGATATCGGGAGGAACACCAATGGCGAAAGCAGGTAACTGGACTATTACTGACACTCAGCTGCGAAAGCTAGGGGAGCAAACAGG
## 938                                                          ACAGAGACCTCAAACGTTATCCGGATTCATTGGGCGTAAAGCGTCCGCAGGTGGTTTGATAAGTGAGTGGTTAAATCCCGGGGCTTAACTTCGGGGCTGCCGTTCATACTGTCAAGCTAGAGGATGGAAGAGGTAAGCGGAATTCTCGGTGTAGTCGTAATAAGCGCTGATATCGAGAAGAACACCAAAGGCGAAGGCAGCTTACTGGTACATTCCTGACACTCATGGACGAAAGCGTGGGGAGCGAATAGG
## 941                                                         ACATGTGGGGCGAGCGTTGTTCGGAATTACTGGGCATAAAGGGTGCGTAGGCGGTCTTGTACGTCTGGGGTGAAAACCTGCGGCCTAACCGTAGGACGGCCTTGGATACGGCTCGACTTGAGTGCGTGAGGGGTGATCGGAACGCGTGGTGTAGCGGTGAAATGCGTAGATATCACGCGGAAGGCCAACGGCGAAGGCAGATCACTGGTGCGCAACTGACGCTGAGTGCACGAAAGCGTGGGGAGCAAACAGG
## 942                                                          ACGAAGGGGGCAAGCGTTGTTCGGAATTACTGGGCGTAAAGGGCGCGTAGGCGGCTTATCAAGTCAGGCGTGAAATTCCCGGGCTCAACCTGGGGGCTGCGCTTGATACTGATGAGCTTGAATGCGGGAGAGGATAGTGGAATTCCCAGTGTAGAGGTGAAATTCGTAGATATTGGGAAGAACACCGGTGGCGAAGGCGGCTATCTGGCCCGTAATTGACGCTGAGGCGCGAAAGCGTGGGGAGCAAACAGG
## 943                                                          ACGGAGGGTGCAAGCGTTATCCGGAATCACTGGGTTTAAAGGGTGCGTAGGCGGGGGATTAAGTCAGTGGTGAAAGCCTGTAGCTTAACTACAGAATTGCCATTGATACTGATTTTCTTGAATTGGGTTGAGGTTAGCGGAATGTGACATGTAACGGTGAAATGTTTAGATATGTCATGGAACACCGATTGCGAAGGCAGCTAGCTGGGCCTTGATTGACGCTGAGGCACGAAAGCGTGGGTAGCGAACAGG
## 944                                                          ACGGAGGGTGCAAGCGTTAATCGGAATCACTGGGCGTAAAGCGCACGTAGGCGGCTTGGTAAGTCAGGGGTGAAATCCCACAGCCCAACTGTGGAACTGCCTTTGATACTGCCAGGCTTGAGTACCGGAGAGGGTGGCGGAATTCCAGGTGTAGGAGTGAAATCCGTAGATATCTGGAGGAACACCGGTGGCGAAGGCGGCCACCTGGACGGTAACTGACGCTGAGGTGCGAAAGCGTGGGTAGCAAACAGG
## 945                                                          ACGAAGGGTGCAAGCGTTGTTCGGAATTACTGGGCGTAAAGGGCGCGTAGGTTGGTTTGTAAGTCTAATGTGAAATCCCCGGGCTTAACCCGGGACGTGCATTGGAAACTGCAGATCTTGAATGCTTAAGAGGGTGGTGGAATTCCAGGAGTAGAAGTGAAATTCGTAGATATCTGGAGGAACACCTGAGGCGAAGGCGGCCACCTGGTAAGACATTGACGCTGAGGCGCGAAAGTGCGGGGAGCAAACAGG
## 946                                                         ACGGAGGATGCGAGCGTTATCCGGATTTATTGGGTTTAAAGGGTTCGTAGGCGGCTTTGTAAGTCAGCGGTGAAATACCAACGCTCAACGTTGGGGCTGCCGTTGATACTGCTGGGCTTGAGATGGATCGACGATGGCGGAATGTGACAAGTAGCGGTGAAATGCATAGATATGTCACAGAACACCGATAGCGAAGGCAGCTGTCGAGGTCATCATCTGACGCTGAGGAACGAAAGCGTGGGGATCAAACAGG
## 947                                                         ACAGAGGGTGCAAGCGTTAATCGGAATTACTGGGCGTAAAGGGCTTGTAGGTGGTTAGATAAGTTGGACGTGAAAACCCTGGGCTCAACCTAGGAATGGCGACCAATACTGTTTAGCTAGAGTATAGTAGAGGGTATAGGAATTTCCGGTGTAGCGGTGAAATGCGTAGATATCGGAAGGAACATCAGTGGCGAAGGCGTATACCTGGACTAAGAACTGACACTGAGGAGCGAAAGCGTGGGGATCAAACAGG
## 949                                                          ACGGAGGATTCAAGCGTTATCCGGATTTATTGGGTTTAAAGGGTGCGTAGGCGGTTTGATAAGTTAGAGGTGAAATTTCGGGGCTCAACCCTGAACGTGCCTCTAATACTGTTGAGCTAGAGAGTAGTTGCGGTAGGCGGAATGTATGGTGTAGCGGTGAAATGCTTAGAGATCATACAGAACACCGATTGCGAAGGCAGCTTACCAAACTATATCTGACGTTGAGGCACGAAAGCGTGGGGAGCAAACAGG
## 952                                                          ACGGAGGATGCGAGCGTTATCCGGATTCATTGGGTTTAAAGGGTGCGCAGGCGGATATATAAGTCGGAGGTGAAATTCTGATGCTCAACATCAGCCGTGCCTTCGATACTGTATATCTAGAGTCAAGTTGGAGTAGGCGGAATGTGTTGTGTAGCGGTGAAATGCTTAGATATAACACAGAACACCGATCGCGAAGGCAGCTTACTAAACTTTGACTGACGCTCATGCACGAAAGCGTGGGGATCAAACAGG
## 953                                                         ACGGAGGATGCGAGCGTTATCCGGATTTATTGGGTTTAAAGGGTTCGTAGGCGGCTGAATAAGTCAGCGGTGAAATACCAGAGCTTAACTCTGGGGCTGCCGTTGATACTGTTTAGCTAGAGTATGATCGACGTTGGCGGAATGTGACAAGTAGCGGTGAAATGCATAGATATGTCACAGAACACCGATAGCGAAGGCAGCTGACGAGGTCACATACTGACGCTGAGGAACGAAAGCGTGGGGATCAAACAGG
## 954                                                          ACGAAGGGTGCAAGCGTTGTTCGGAATCATTGGGCGTAAAGCGTGCGTAGGCGGCTTAGTAAGTCGTTTGTGAAATCCCAGGGCTTAACCTTGGAATTGCAATCGAAACTGCTTTGCTAGAGTTTAGTGGGGGATAGTGGAATTCCTAGTGTAGGGGTGAAATCCGTAGAGATTAGGGGGAACATCAGTGGCGAAAGCGACTATCTACGCTAATACTGACGCTGAGGTACGAAAGCGTGGGGAGCAAACAGG
## 955                                                          ACGAAGGTCCCAAGCGTTGTTCGGAATCATTGGGCGTAAAGCGGATGTAGGCGGTTCCATAAGTCAGAGGTGAAATCCCGGGGCTTAACCCCGGAAGTGCCTTTGAGACTGTGGAACTTGAATGTAGGAGAGGGTGCTAGAATTCCTGGTGTAGTGGTGAAATACGTAGAGATCAGGAGGAATTCCAGATGCGAAGGCGGGTGCCTGGCCTAACATTGACGCTGAGATCCGAAAGCGTGGGGATCAAACAGG
## 957                                                          ACGAAGGGTGCAAGCGTTACTCGGAATTACTGGGCGTAAAGCGTGCGTAGGCGGTTTGTTAAGTCTGATGTGAAATCCCCGGGCTCAACCTGGGACGTGCATTGGAAACTGGCAATCTAGAGTACGGTAGAGGGTGGTGGAATTCCCGGTGTAGCAGTGAAATGCGTAGAGATCGGGAGGAACACTCGTGGCGAAGGCGGCCACCTGGACCAGTACTGACGCTGAGGCACGAAAGCGTGGGGAGCAAACAGG
## 958                                                          ACGTAGGGTGCAAGCGTTAATCGGAATTACTGGGCGTAAAGCGTGCGCAGGCGGTTATATAAGTCAGATGTGAAATCCCCGGGCTCAACCTGGGAACTGCATTTGAGACTGTATAGCTAGAGTACGGTAGAGGGGGATGGAATTCCGCGTGTAGCAGTGAAATGCGTAGATATGCGGAGGAACACCGATGGCGAAGGCAGCCTCCTGGGCCAGTACTGACGCTCATGCACGAAAGCGTGGGGAGCAAACAGG
## 959                                                          ACGGAGGGTGCAAGCGTTATCCGGATTCACTGGGTTTAAAGGGTGCGTAGGCGGGCTAGTAAGTCAGTGGTGAAATCCCCGAGCTTAACTTGGGAACTGCCGTTGATACTATTAGTCTTGAATATCGTGGAGGTAAGCGGAATATGTCATGTAGCGGTGAAATGCTTAGATATGACATAGAACACCAATTGCGAAGGCAGCTTGCTACACGGTCATTGACACTGAGGCACGAAAGCGTGGGGAGCAAACAGG
## 960                                                          ACAGAGGGTGCGAGCGTTAATCGGAATTACTGGGCGTAAAGCGTGTGTAGGTGGTTAGATAAGTTAGATGTGAAATCCCTGGGCTTAACCTGGGCACTGCGTTTAAGACTGTTTAGCTAGAGTACTGTAGAGGATAGTGGAATTTCCAGTGTAGCGGTGAAATGCGTAGATATTGGAAGGAACACCAGTGGCGAAGGCGACTATCTGGACAGATACTGACACTGAGACACGAAAGCGTGGGGAGCAAACAGG
## 961                                                          ACGGAGGGTGCAAGCGTTATCCGGATTTACTGGGTTTAAAGGGTGTGTAGGCGGGCTATTAAGTCAGTGGTGAAATCTCCGAGCTTAACTTGGAAACTGCCATTGATACTATTAGTCTTGAATTTTGTTGAGGTAGGCGGAATAAGTCATGTAGCGGTGAAATGCATAGATATGACTTAGAACACCAATTGCGAAGGCAGCTTGCTAAACAAACATTGACGCTGAGGCACGAAAGCGTGGGGATCAAACAGG
## 962                                                          ACAGAGGGTGCAAGCGTTGCTCGGAATTATTGGGCGTAAAGGGCAGGTAGGTGGTCTCGTTTGTCCCGGGTGAAATCCCTGAGCTTAACTCAGGAAGTGCCCTGGAAACGGCGAGACTAGAGTTCTGGAGAAGGTCGTGGAATTCCCAGTGTAGCGGTGAAATGCGTAGAGATTGGGAGGAACACCAGAGGCGAAAGCGGCGACCTAGACAGATACTGACACTCAACTGCGAAAGCGTGGGGAGCAAACAGG
## 964                                                          ACGGAGGGAGCTAGCGTTGTTCGGAATTACTGGGCGTAAAGCGTGCGTAGGCGGTGACTCAAGTCAGAGGTGAAAGCCTGGAGCTCAACTCCAGAACTGCCTTTGAAACTAGGTCGCTAGAATCTGGGAGAGGTGAGTGGAATTCCGAGTGTAGAGGTGAAATTCGTAGATATTCGGAAGAACACCAGTGGCGAAGGCGGCTCACTGGACCAGAATTGACGCTGAGGCACGAAAGCGTGGGGAGCAAACAGG
## 966                                                          ACGAGGGGAGCTAGCGTTGTTCGGAATTACTGGGCGTAAAGCGCGCGTAGGCGGCTTTTTAAGTCAGAGGTGAAAGCCCGGGGCTCAACCCCGGAATAGCCTTTGAAACTGGAAAGCTAGAATCTTGGAGAGGTCAGTGGAATTCCGAGTGTAGAGGTGAAATTCGTAGATATTCGGAAGAACACCAGTGGCGAAGGCGACTGACTGGACAAGTATTGACGCTGAGGTGCGAAAGCGTGGGGAGCAAACAGG
## 969                                                          ACGAGTGCCCCGAGCGTTATCCGGAATTATTGGGCGTAAAGGGTGTGTAGGCGGTCGTGTTAGTCCTCCGTTAAATCTCTCGGCTCAACCGAGAACCAGCGGGGGAGACGGCACGACTCGAGGGTATGAGAGGTGCATGGAACTCACAGTGTAGGGGTGAAATCCGTTGATATTGTGGGGAACACCAAATGCGAAGGCAGTGCACTGGCATACTTCTGACGCTGAGACACGAAAGCGTGGGTCGCGAATGGG
## 972                                                          ACAGAGAGTGCAAGCGTTAATCGGAATTATTGGGCGTAAAGGGTGTGTAGGTTGTTAAAAAAGTTAGATGTGAAATCCCTGGGCGCAACCTAGGAATTGCATTTAATACTTTTTATCTAGAGTATTGTAGGGGGAAGTGGAATTTCCGGTGTAGCGGTGAAATGCGTAGATATCGGAAGGAACACCAGTGGCGAAGGCGACTTCCTGGACAAATACTGACACTGAGACACGAAAGCGTGGGGAGCAAACAGG
## 973                                                          ACGGAGGATCCAAGCGTTATCCGGAATCATTGGGTTTAAAGGGTCCGTAGGCGGTTTGGTAAGTCAGTGGTGAAAGCCCTTCGCTCAACGGAGGAACGGCCATTGATACTGCCAGACTTGAATTACTGGGAAGTAACTAGAATATGTAGTGTAGCGGTGAAATGCTTAGATATTACATGGAATACCAATTGCGAAGGCAGGTTACTACCGGTGTATTGACGCTGATGGACGAAAGCGTGGGGAGCGAACAGG
## 975                                                          ACGGGAGTGGCAAGCGTTATCCGGAATTATTGGGCGTAAAGCGTCCGCAGGCGGCCTTGTAAGTCTGTCGTCAAAGCGTGGAGCTTAACTCCATTTAAGCGATGGAAACTGCAAGGCTAGAGTGTGGTAGGGGCAGAGGGAATTCCCGGTGTAGCGGTGAAATGCGTAGATATCGGGAAGAACACCAGTGGCGAAGGCGCTCTGCTGGGCCATAACTGACGCTCATGGACGAAAGCCAGGGGAGCGAAAGGG
## 976                                                          ACGTAGGTGGCAAGCGTTATCCGGAATTATTGGGCGTAAAGCGCGCGTAGGCGGTTTTTTAAGTCTGATGTGAAAGCCCACGGCTCAACCGTGGAGGGTCATTGGAAACTGGAAAACTTGAGTGCAGAAGAGGAAAGTGGAATTCCATGTGTAGCGGTGAAATGCGCAGAGATATGGAGGAACACCAGTGGCGAAGGCGACTTTCTGGTCTGTAACTGACGCTGATGTGCGAAAGCGTGGGGATCAAACAGG
## 977                                                          ACATAGGGTGCAAGCGTTGTCCGGAATTATTGGGCGTAAAGAGCTCGTAGGTGGTTCGTCACGTCGGATGTGAAACTCTGGGGCTTAACCCCAGACCTGCATTCGATACGGGCGAGCTTGAGTATGGTAGGGGAGTCTGGAATTCCTGGTGTAGCGGTGGAATGCGCAGATATCAGGAGGAACACCAATGGCGAAGGCACTCTACTGGACCATAACTGACACTGAGAGACGACAGCTAGGGGAGCAAATGGG
## 978                                                          ACGTAGGGTGCAAGCGTTGTTCGGAATCATTGGGCGTAAAGCGCGTGTAGGCGGTTAGTTAAGTCAGGTGTGAAATCCCTCGGCTCAACCGAGGAAGTGCATTTGAAACTAGCTAGCTCGAAGATGGTAGAGGAAGGTGGAATTCCAAGTGTAGAGGTGAAATTCGTAGATATTTGGAGGAATACCTGTGGCGAAGGCGGCCTTCTGGGCCATTCTTGACGCTGAGACGCGAAAGCGTGGGTAGCAAACAGG
## 980                                                          ACGGAGGGTGCAAGCGTTATCCGGATTCACTGGGTTTAAAGGGAGCGTAGGCGGACTTGTAAGTCCGTGGTGAAAGCCCTCAGCTTAACTGAGGAACTGCCATGGATACTATAAGTCTTGAATGTCGTTGAGGTTTGCGGAATAGGTCATGTAGCGGTGAAATGCTTAGATATGACCTAGAACACCTATTGCGAAGGCAGCAGGCTAAACGAATATTGACGCTGAGGCTCGAAAGCGTGGGGATCAAACAGG
## 981                                                          ACGGAGGATGCAAGTGTTATCCGGAATCACTGGGCGTAAAGCGTCTGTAGGTGGTCAAATAAGTCAACTGTTAAATCTTGAGGCTCAACCTCAAAATCGCAGTCGAAACTATTAGACTAGAGTATAGTAGGGGTAAAGGGAATTTCCAGTGGAGCGGTGAAATGCGTAGAGATTGGAAAGAACACCGATGGCGAAGGCACTTTACTGGGCTATTACTGACACTCAGAGACGAAAGCTAGGGTAGCAAATGGG
## 982                                                         ACGGGGGCTCCAAGCGTTGTCCGGTATCATTGGGCGTAAAGAGTTATGTAGGCGGTTATAAAAGTTACGGGTTAAATATTTGGGCTCAACCCAAAATCTGCTTGTAATACTGTATGACTAGAGAAAATCAGGGGAAGGTAGAACGCATGGTGTAGCGGTAAAATGCTTTGATATCATGCGGAATACTAAAGGCGTAAGCAGCCTTCTGGGATTTTTCTGACGCTGAGATACGAAAGCGTGGGTAGCAAAACGG
## 983                                                          ACGAAGGGTGCAAGCGTTGTTCGGAATCACTGGGCGTAAAGAATGCGTAGGCGGCTTAACAAGTCGAGAGTGAAATCCCAGGGCTCAACCCTGGAACTGCTTTCGAAACTGTTTTGCTAGAGTCCCGGAGGGGTTAGCGGAATTCCGAGTGTAGAGGTGAAATTCGCAGATATTCGGAGGAACACCAGTGGCGAAGGCGGCTAACTGGACGGGAACTGACGCTGAGGCATGAAAGTGTGGGGATCAAACAGG
## 984                                                          ACGTAGGTGGCAAGCGTTGTCCGGATTTATTGGGCGTAAAGCGAGCGCAGGCGGTTCCTTAAGTCTGATGTGAAAGCCCACGGCTCAACCGTGGAAGGTCATTGGAAACTGGGGAACTTGAGTGCAGAAGAGGAGAGTGGAATTCCATGTGTAGCGGTGAAATGCGTAGATATATGGAGGAACACCAGTGGCGAAGGCGACTCTCTGGTCTGTAACTGACGCTGAGGCTCGAAAGCGTGGGGAGCAAACAGG
## 985                                                          ACGGAGGGTGCGAGCGTTAATCGGAATTACTGGGCGTAAAGCGCGCGTAGGTGGCTTGGCACGCCGGTTGTGAAAGCCCCGGGCTCAACCTGGGAACGGCATCCGGAACGGCCAGGCTAGAGTGCAGGAGAGGAAGGTAGAATTCCCGGTGTAGCGGTGAAATGCGTAGAGATCGGGAGGAATACCAGTGGCGAAGGCGGCCTTCTGGCCTGACACTGACACTGAGGTGCGAAAGCGTGGGTAGCAAACAGG
## 986                                                          ACGAAGGGTGCAAGCGTTGTTCGGATTTATTGGGCGTAAAGCGCGCGTAGGCGGACCTGCAAGTCAGATGTGAAATCTCGGGGCTCAACCCCGAAACTGCGTCTGAAACTACAGGTCTAGAATCTTGGAGGGGGAAGGGGAATTTCGCATGTAGGGGTAAAATCCGTAGAGATGCGAAGGAACACCAGAGGCGAAGGCGCCTTCCTGGACAAGTATTGACGCTGAGGCGCGAAAGCGTGGGGATCAAACAGG
## 988                                                          ACGGAGGGTGCAAGCGTTAATCGGAATTACTGGGCGTAAAGCGCACGCAGGCGGTCTGTTAAGTCAGATGTGAAATCCCCGGGCTCAACCTGGGAACTGCATTTGAAACTGGCAGGCTTGAGTCTCGTAGAGGGAGGTAGAATTCCAGGTGTAGCGGTGAAATGCGTAGAGATCTGGAGGAATACCGGTGGCGAAGGCGGCCTCCTGGACGAAGACTGACGCTCAGGTGCGAAAGCGTGGGGAGCAAACAGG
## 989                                                          ACGAAAGGTGCAAGCGTTACTCGGAATCACTGGGCGTAAAGCGCACGTAGGCGGACTCGTAGGTCAATTGTGAAATACCTGGGCTCAACCCGGGAACTGCAGTTGAAACCGCTTGTCTTGAATACCTGAGAGGGTGGGGGAATACCTGGTGGAGAAGTGAAATTCGTAGAGATCAGGTGGAACACCGGAGGCGAAGGCGCCTGCCTGGCAGTGTATTGACGCTGAGGTGCGAAAGTGCGGGGAGCAAACAGG
## 992                                                          ACGGAGGGTGCAAGCGTTATCCGGAATCATTGGGTTTAAAGGGTCCGCAGGCGGATTTATAAGTCAGTGGTGAAATCCTGCAGCTCAACTGCAGAACTGCCATTGATACTGTAAGTCTTGAATTTGATCGGAGTGGGCGGAATGTGTAGTGTAGCGGTGAAATGCATAGATATTACACAGAACACCGATAGCGAAGGCAGCTCACTAGGTTATAATTGACGCTCATGGACGAAAGCGTGGGGATCAAACAGG
## 993                                                          ACAGAGGTCCCAAGCGTTGTTCGGATTCACTGGGCGTAAAGGGTGCGTAGGCGGTCGGGTAAGTCTGACGTGAAATCTTCAAGCTCAACTTGGAAACTGCGTCGGATACTATTCGGCTAGAGGAATGGAGGGGAGACTGGAATACTTGGTGTAGCAGTGAAATGCGTAGATATCAAGTGGAACACCAGTGGCGAAGGCGAGTCTCTGGACATTTCCTGACGCTGAGGCACGAAAGCCAGGGGAGCAAACGGG
## 994                                                          ACGAACCGTACGAACGTTATTCGGAATTATTGGGCTTAAAGGGTGCGTAGGCTGTGCAGAAAGTTGGGTGTGAAAGCCCTCGGCTCAACCGAGGAATTGCGCCCAAAACTACTGTGCTCGAGGGAGACAGAGGTGAGCGGAACTCAAGGTGGAGCGGTGAAATGCGTTGATATCTTGAGGAACACCGGTGGCGAAAGCGGCTCACTGGGTCTCTACTGACGCTGAGGCACGAAAGCCAAGGTAGCAAACGGG
## 995                                                          ACGAAGGTGGCAAGCGTTACTCGGAATTACTAGGCGTAAAGGGCAGGTAGGTGGTTTGATAAGTCTGTTGTGTAAGCTCTCGGCTTAACCGGGAGAGGTCAACAGAAACTGTCAGGCTTGAGTATAGGAGAGGGTACTGGAATTCCCGGTGTAGCGGTGAAATGCGCAGAGATCGGGAGGAACACCAATGGCGAAAGCAGGTACCTGGACTATTACTGACACTAAGCTGCGAAAGCTAGGGGAGCAAACAGG
## 996                                                          ACGGAGGGTGCAAGCGTTAATCGGATTTATTGGGCGTAAAGGGCGCGTAGGCTGATGAATAAGTCGGGTGTGAAATCCTGGGGCTTAACCCCAGGGCTGCACCTGAAACTGTTTGTCTAGAGGGTAGGCGGAGAAAACGGAATTCCACAAGTAGCGGTGAAATGCGTAGATATGTGGAAGAACACCGGTGGCGAAGGCGGTTTTCTAGCTTACTCCTGACGCTGAGGCGCGAAAGTTAGGGGATCAAACAGG
## 998                                                          ACGAAGGACCCAAGCGTTATCCGGATTTATTGGGTTTAAAGGGTGCGTAGGCGGGTTGTTAAGTCAGTGGTGAAAGCCTAGAGCTTAACTCTAGAATTGCCATTGATACTGACGATCTTGAGGATAGTTGAGGTGGGCGGAATGTATCATGTAGCGGTGAAATGCTTAGAGATGATACAGAACACCGATTGCGAAGGCAGCTCACTAAGCTATTTCTGACGCTGAGGCACGAAAGCGTGGGGATCAAACAGG
## 999                                                          ACGTAGGGTGCAAGCGTTAATCGGAATTACTGGGCGTAAAGCGTGCGCAGGCGGTTATATAAGTCAGATGTGAAATCCCCAGGCTTAACCTGGGACGTGCATTTGAAACTGTGATTCTTGAGTATTGGAGAGGGTAGTGGAATTGTAGGTGTAGGAGTGACATCCGTAGAGATCTGCAGGAACATCAGAGGCGAAGGCGACTACCTGGCCGATTACTGACACTGAGGAACGAAAGCGTGGGGAGCAAACAGG
## 1000                                                         ACAGAGGGTGCAAGCGTTGTTCGGAATTACTGGGCGTAAAGGGCGTGTAGGCGGCTTAGCAAGTCAGATGTGAAATCCCGAGGCTTAACTTCGGAAGTGCATTTGAAACTGCTAGGCTAGAGTGATGTAGAGGTTGGTGGAATTCCCAGTGTAGAGGTGAAATTCGTAGATATTGGGAGGAACACCGGTGGCGAAGGCGGCCAACTGGGCATTAACTGACGCTGAGGCGCGAAAGCGTGGGGAGCAAACAGG
## 1001                                                         ACGAGTGGCCCTAGCGTTAGTCGGAATTACTGGGCTTAAAGGGTGCGTAGGCGGACCTGTAAGTGCTTTGTGAAAGCCCCTCGCTCAACGAGGGAACCGCAGCGCATACTGCAGGTCTTGAAGAAGTTAGAGGCCGGTGGAACGATAGGTGGAGCGGTGGAATGCGTAGAGATCTATCGGAACGCCAATGGTGAAGACAACCGGCTGGGACTTTCTTGACGCTGAGGCACGAAAGCGTGGGGAGCAAACAGG
## 1003                                                         ACGGAGGGTGCGAGCGTTATCCGGAATTACTGGGTTTAAAGGGTGCGTAGGCGGCTTTGTAAGTCAGGAGTGAAAGTTTGCGGCTCAACCGTAAAATTGCTTTTGATACTGCAGAGCTAGAATCAGGATGAGGTTAGCGGAATGTGGCATGTAGCGGTGAAATGCATAGATATGCCATAGAACACCAATTGCGAAGGCAGCTGGCTAGACCTGCATTGACGCTGAGGCACGAAAGCGTGGGGAGCGAACAGG
## 1005                                                         ACAGAGACCCCGAGCGTTATCCGGATTTATTGGGCGTAAAGGGCAGGTAGGCGGACATGTTAGTCGGATGTCAAATATCCCGGCTCAACCGGGAAAAGGCATTCGAAACGGCATGTCTCGAGAATGTGAGAGATCGGTGGAATTCACGGTGTAGTAGTGAAATGCGTTGATATCGTGAGGAACACCCAAGGCGAAGGCAACCGATTGGCACATTTCTGACGCTGAGCTGCGAAAGCGTGGGGAGCGAATGGG
## 1006                                                        ACGAGAGGAGCGAGCATTATTCGAAATGATTAGGCGTAAAGGGTTTGTAGGTTGTTTTTTAAGTTGAAAAAAAAATATGAAAGCTTAACTTTCTTTCTTTTTTCAAAACTGATAAACTAGAGTATAAATAGAGGATAATAGAATTCTTATTGTAAGGATAAAATCTTATAATAATAAGAGGAATTTCAAAGGCGAAGGCAATTATCTGGGTATATACTGACACTGAGGAACGAAAGCTTGGGTAGCGAACGGG
## 1007                                                         ACGTAGGGTGCTAGAGTTGTCCGGATTTACTGGGTGTAAAGGGTGCTCAGGTGGGTTTGTAAGTCAGAGGTGAAATCCCAAAGCTTAACTTTGGAGCTGCCTTTGATACTGCAAGTCTGGAGTTTGAGAGAGGGCAATGGAATATCTGGTGTAGCAGTGAAATGCGTAGATATCAGATAGAACACCAATGGCGAAGGCAGTTGCCTGGCTCAAAACTGACGCTAAAGCACGAAAGTGTGGGGAGCAAACAGG
## 1009                                                         ACGAGCTCCCCAAACGTTATTCGGTATCACTGGGCTTAAAGAGTTCGTAGGCGGCGAAGTAGGTGAGGTGTGAAAGCCCTCGGCTCAACCGAGGAACTGCGCTTCAAACCACTTTGCTAGAGGGAGATAGGGGTAAGCGGAACAGATGGTGGAGCGGTGAAATGCATTGATATCATCTGGAACACCGGTGGCGAAAGCGGCTTACTGGGTCTTTTCTGACGCTGAGGAACGAAAGCTAGGGTAGCGAACGGG
## 1010                                                         ACGAAGGTGGCGAGCGTTACTCGGAATTACTAGGCGTAAAGGGCAGGTAGGCGGTTTGATAAGTCTGTTGTGTCAGCTCCTGGCTTAACTGGGAGAGGTCAACGGAAACTATCAGGCTTGAGTGTAGGAGAGGATACTGGAATTCCTGGTGTAGCGGTGAAATGCGCAGAGATCAGGAGGAACACCAATGGCGAAAGCAAGTATCTGGACTATTACTGACGCTAAGCTGCGAAAGCTAGGGGAGCAAACAGG
## 1012                                                         ACGTAGGGTGCGAGCGTTAATCGGAATTACTGGGCGTAAAGCGTGCGCAGGCGGATTGTTAAGCAAGACGTGAAATCCCCGGGCTTAACCTGGGAATGGCGTTTTGAACTGGCAGTCTAGAGTGTGTCAGAGGGGGGTGGAATTCCACGTGTAGCAGTGAAATGCGTAGAGATGTGGAGGAACACCAATGGCGAAGGCAGCCCCCTGGGATAACACTGACGCTCATGTACGAAAGCGTGGGTAGCAAACAGG
## 1013                                                         ACAGAGGTCTCAAGCGTTGTTCGGAATCACTGGGCGTAAAGGGTGCGTAGGTGGCGTGGTAAGTCAGATGTGAAAGCCCGGGGCTCAACCCCGGAATTGCATCCGATACTACCATGCTGGAGTACTGAAGAGGTGACTAGAATTCTAGGTGTAGCAGTGAAATGCGTAGATATCTAGAGGAATACCAAAGGCGAAGGCAGGTCACTGGGCAGTTACTGACACTGAGGCACGAAGGCCAGGGTAGCGAACGGG
## 1014                                                        ACGTAGGTGGCGAGCGTTATCCGGATTTATTGGGTTTAAAGGGTGCGTAGGCGGCTTAATAAGTCGGTGGTTAAAAGTAGCAGCTTAACTGTTTTACATGCCATCGATACTGTTGAGCTTGAGATATTTGGAGGCAGCTAGAATTTCCGGTGTAGCGGTGAAATGCATAGATACCGGAAGGAATACCAATTGCGAAGGCAGGTTGCTACGAATAAACTGACGCTGATGCACGAAAGCGTGGGGATCAAACAGG
## 1015                                                         ACGTAGGGTGCGAGCGTTAATCGGAATGACTGGGCGTAAAGAGTGCGTAGGCGGTTACGCGAGCTTGATGTGAAATCCCCGGGCTTAACCTGGGAATGGCATGGAGGACAGCGTAGCTAGAGTCTGTCAGAGGTGGGTAGAATTCCCTGTGTAGCAGTGAAATGCGTAGAGATGGGGAGGAATACCGATGGCGAAGGCAGCCTGCTGGGATAAGACTGACGCTGAGGCACGAAGGCGTGGGGAGCAAACAGG
## 1016                                                         ACGGAGGGTGCAAGCGTTATCCGGAATCACTGGGTTTAAAGGGTGCGTAGGCGGTTGTATAAGTCAGTGGTGAAAGGCCGTAGCTTAACTATGGGATTGCCATTGATACTGTATGACTTGAATGAGGTTGAGGTTGGCGGAATGTGGCATGTAGCGGTGAAATGCTTAGATATGCCATGGAACATCGATTGCGAAGGCAGCTGACTGGACCTGAATTGACGCTGAGGCACGAAAGCGTGGGGAGCGAACAGG
## 1017                                                         ACGTAGGGTGCGAGCGTTAATCGGAATTACTGGGCGTAAAGCGTGCGCAGGCGGTTTCGTAAGACAGATGTGAAATCCCCGGGCTTAACCTGGGAACTGCATTTGTGACTGCGAGACTAGAGTGTAGCAGAGGGGGGTAGAATTCCACGTGTAGCAGTGAAATGCGTAGAGATGTGGAGGAATACCGATGGCGAAGGCAGCCCCCTGGGCTAACACTGACGCTCATGCACGAAAGCGTGGGGAGCAAACAGG
## 1018                                                         ACGAGTGCCCCAAGCGTTATCCGGAATTACTGGGCGTAAAGGGTGCGTAGGTGGCGTGGTTAGTCTCTTGTTAAAGCTCCCGGCTTAACCGGGAAAGTGCGAGAGATACGGCCAGGCTCGAGGTGGTGAGAGGTCTATGGAACTCATGGTGTAGGGGTGAAATCCGTTGATATCATGGGGAACACCAAATGCGAAGGCAATAGGCTGGCACCATACTGACACTGAAGCACGAAAGCGTGGGGATCAAACGGG
## 1019                                                         ACAGAGGGTGCAAGCGTTGTTCGGAATTATTGGGCGTAAAGCGCGTGTAGGCGGCTTTCTAAGTCTGGTGTGAAAGCCCGGGGCTTAACCTCGGAAGTGCACTGGATACTGGATCGCTCGAGGACGGGAGAGGGTAGTGGAATTCCTGGTGTACCGGTGAAATGGGTAGATATCAGGAAGAACATCGGTGGCGAAGGCGGCTACCTGGCCCGTATCTGACGCTGAGACGCGAAAGCGTGGGGAGCAAACAGG
## 1020                                                         ACGTAGGGTGCAAGCGTTAATCGGAATTACTGGGCGTAAAGCGTGCGCAGGCGGTTATATAAGACAGATGTGAAATCCCCGGGCTCAACCTGGGAACTGCATTTGTGACTGTATAGCTAGAGTACGGCAGAGGGGGATGGAATTCCGCGTGTAGCAGTGAAATGCGTAGATATGCGGAGGAACACCGATGGCGAAGGCAATCCCCTGGACCTGTACTGACGCTCATGCACGAAAGCGTGGGGAGCAAACAGG
## 1021                                                         ACGTAGGGTGCAAGCGTTAATCGGAATTACTGGGCGTAAAGGGTGCGCAGGCGGTTTTGTAAGTCAGATGTGAAATCCCCGGGCTTAACCTGGGAATTGCGTTTGAAACTACAAAACTGGAGTGTAGCAGAGGGAGGTGGAATTTCATGTGTAGCAGTGAAATGCGTAGAGATATGGAAGAACATCGATGGCGAAGGCAGCCTCCTGGGTTAACACTGACGCTCATGCACGAAAGCGTGGGGAGCAAACAGG
## 1022                                                         ACGAAGGGGGCTAGCGTTGCTCGGATTTACTGGGCGTAAAGGGCGCGTAGGCGGACATTTAAGTCAGGGGTGAAATCCCGGGGCTCAACCTCGGAACTGCCTTTGATACTGGGTGTCTTGAGTATGAGAGAGGTGTGTGGAACTCCGAGTGTAGAGGTGAAATTCGTAGATATTCGGAAGAACACCAGTGGCGAAGGCGACACACTGGCTCATTACTGACGCTGAGGCGCGAAAGCGTGGGGAGCAAACAGG
## 1024                                                         ACGTAGGGGGCAAGCGTTATCCGGATTTACTGGGTGTAAAGGGAGCGCAGACGGAGAGGTAAGTCAGATGTGGAAGCCCCGGGCATAACCCGGGGAGTGCATTTGAAACTATCACTCTTGAGTACTGGAGAGGTAAGCGGAATTCCTGGTGTAGCGGTGAAATGCGTAGATATCAGGAGGAACACCGGTGGCGAAGGCGGCTTACTGGACAGAAACTGACGTTGAGGCTCGAAAGCGTGGGTAGCAAACAGG
## 1026                                                         ACGGAGGGTGCAAGCGTTGTTCGGAATTACTGGGCGTAAAGTGTGTCTAGGTGGGCATGTAAGTCAGATGTGAAATTACCCGGCTCAACCGGGGAGGGTCATTTGATACTGCATGTCTTGAGTCCCGAAGAGGTGGGTGGTATTCCCAGTGTAGAGGTGAAATTCGTAGATATTGGGAGGAACACCGGAGGCGAAAGCGACTCACTGGTCGGGCACTGACACTAAAGCACGAAAGCGTGGGTAGCAAACAGG
## 1027                                                          ACGGGAGGGGCAAGCGTTATCTGAAATAACTGGGCGTAAAGAGTCCGTAGACTGTAAAGTAAGTTACTTGTTAAATTTTTAAGCTTAACTTTAAGTTAGCTTGTAATACTGCTTTACTTGAGTTTTATACAGAAAAGTAGAATTTTATACGAAAGGGTAAAATCTTAAGATATATAAAGGAATACCAGCAGCGAAGGCGACTTTTTAGTAAAAACTGACGTTGAGGGACGAAAGTGTGGGTAGCAAACAGG
## 1028                                                         ACGGAGGGGGCTAGCGTTGTTCGGAATTACTGGGCGTAAAGCGCACGTAGGCGGATCAGAAAGTCAGAGGTGAAATCCCGGGGCTCAACCCCGGAACTGCCTTTGAAACTCCTGATCTTGAGGTCGAGAGAGGTGAGTGGAATTCCGAGTGTAGAGGTGAAATTCGTAGATATTCGGAGGAACACCAGTGGCGAAGGCGGCTCACTGGCTCGATACTGACGCTGAGGTGCGAAAGCGTGGGGAGCAAACAGG
## 1029                                                         ACAGAGGCCTCAAGCGTTGTTCGGAATTACTGGGCGTAAAGGGAATGTAGGTGGCGATGTAAGTCAGATGTGAAAGCCCTGGGCTTAACCTGGGAACGGCATTTGATACTGCATTGCTCGAGTGTGGAAGAGGAGAGTGGAATTCCCGGTGTAGGAGTGAAATCCGTAGATATCGGGAGGAATTCCGATGGCGAAGGCAACTCTCTGGTCCATTACTGACACTGAGATTCGAAAGCATGGGTAGCAAACAGG
## 1030                                                         ACGAAGGTGGCTAGCGTTGTTCGGATTTATTGGGCGTAAAGGGTCCGCAGGCGGTTCGGTAAGTCAGATGTGAAATCCCACAGCTTAACTGTGGACACGCATTTGAAACTGCCAGACTAGAGTATGAGAGGGGTAAGAGGAATTTCCAGTGTAGGGGTGAAATCCGTAGATATTGGAAAGAACACCGGCCGCGAAGGCGTCTTACTGGCTCATTACTGACGCTCAGGGACGAAAGCCAGGGGAGCAAACGGG
## 1031                                                        ACGAGGACCCCGAGCGTTATCCGGATTTATTGGGCGTAAAGAGTGCGCAGGAGGTCTGATGCGTCTTGTGTTAAATGTCACTGCTTAACAGTGTCGCCGCATGAGATACGGTCAGACTAGAGCATGTTAGAGGTTACTAGAACTTATGGTGTAGGGGTGAAATCCGTTGATATCATAGGGAATACCAAAGGCGAAGGCATGTAACTGGAACATTGCTGACTCTCATTGCACGAAAGCGTGGGGAGCAAAAAGG
## 1033                                                         ACGTAGGGTGCGAGCGTTAATCGGAATTACTGGGCGTAAAGCGTGCGCAGGCGGTTTTGTAAGACAGATGTGAAATCCCCGGGCTTAACCTGGGAACTGCGTTTGTGACTGCAAGACTCGAGTGTGGCAGAGGGGGGTGGAATTCCACGTGTAGCAGTGAAATGCGTAGAGATGTGGAGGAACACCGATGGCGAAGGCAGCCCCCTGGGTCAACACTGACGCTCATGCACGAAAGCGTGGGTAGCAAACAGG
## 1034                                                         ACGGAGGATCCAAGCGTTATCCGGATTTATTGGGTTTAAAGGGTGCGTAGGCGGCCTATTAAGTCAGGGGTGAAAGACGGTGGCTCAACCATCGCAGTGCCCTTGATACTGATGGGCTTGATTACACTTGAGGTAGGCGGAATGTGACAAGTAGCGGTGAAATGCATAGATATGTCACAGAACACCAATTGCGAAGGCAGCTTACTAAGGTGTTAATGACGCTGAGGCACGAAAGCGTGGGGATCAAACAGG
## 1035                                                         ACGAGTGGCCCAAGCGTTATCCGGAATTATTGGGCGTAAAGGATGTGTAGGTGGTTATGTTAGTCTAGTGTCTAATCTCTCTGCTCAACAGGGAGCCAGCACAGGAAACGGCATGACTAGAGTATGTTAGGGGTAAGCGGAACTCATAGTGTAGGGGTGAAATCCGTTGATATTATGGGGAACACCAAAAGCGAAGGCAGCTTACTGGAACATTACTGACACTGAAACATGAAAGCGTGGGTAGCGAATGGG
## 1038                                                         ACAGGGGGTGCAAGCGTTGTTCGGAATTACTGGGCGTAAAGGGAGCGTAGGCGGGATTACAAGACAGGGGTTAAATCTCCGGGCTCAACCTGGAACTTGCCTTTGTGACTGTAGTCCTTGAATATGGTAGAGGTCAGTGGAATTTCCGGTGTAGCGGTGGAATGCGTAGAGATCGGAAAGAACACCAGAGGCGAAGGCGACTGACTGGACCAATATTGACGCTGAGGCTCGAAAGCGTGGGGAGCAAACAGG
## 1041                                                         ACGAGTGCCTCAAGCGTTATCCGGAATTATTGGGCGTAAAGGTTGTGTAGGTGGTAATGTTAGTCTCTTGTAAAATTCTTCGGCTTAACCGGGGGCTCGCAAGGGAAACGGCATTACTAGAGGATGCGAGGGGTTTGCGGAACTCATGGTGTAGCGGTGAAATGCGTTGATATCATGGGGAACACCAAAAGCGAAGGCAGCAAACTGGAGCACTCCTGACACTGAAACAAGAAAGCGTGGGTCGCGAATGGG
## 1042                                                         ACGTAGGGTGCAAGCGTTAATCGGAATTACTGGGCGTAAAGCGTGCGCAGGCGGCTTTGCAAGACAGATGTGAAATCCCCGGGCTCAACCTGGGAACTGCGTTCGAAACTGCAAGGCTAGAGTGTGTCAGAGGGGGGTAGAATTCCACGTGTAGCAGTGAAATGCGTAGAGATGTGGAGGAATACCAATGGCGAAGGCAGCCCCCTGGGATAACACTGACGCTCATGCACGAAAGCGTGGGGAGCAAACAGG
## 1043                                                         ACGGAGGGGGCTAGCGTTGTTCGGAATTACTGGGCGTAAAGCGCACGTAGGCGGACCTGAAAGTCAGAGGTGAAATCCCGGGGCTCAACCCCGGAACGGCCTTTGAAACTCCTGGTCTTGAGGTCGAGAGAGGTGAGTGGAATTCCGAGTGTAGAGGTGAAATTCGTAGATATTCGGAGGAACACCAGTGGCGAAGGCGGCTCACTGGCTCGATACTGACGCTGAGGTGCGAAAGCGTGGGGAGCAAACAGG
## 1044                                                         ACGTAGGGTGCAAGCGTTATCCGGAATTATTGGGCGTAAAGAGCTCGTAGGCGGTTTGTCGCGTCTGCTGTGAAAATCTGAGGCTCAACCTCGGACTTGCAGTGGGTACGGGCAGACTAGAGTGCGGTAGGGGAGAAGGGAATTCCTGGTGTAGCGGTGGAATGCGCAGATATCAGGAGGAACACCAATGGCGAAGGCACTTCTCTGGGCCGCTACTGACGCTGAGGAGCGAAAGCGTGGGGAGCGAACAGG
## 1045                                                         ACGAGTGCCTCAAGCGTTATCCGGAATCATTGGGCGTAAAGGTTGTGTAGGTGGTTATATTAGTCTTTTGTTAAATTCTTCGGCTTAACCGGGGGCATGCAGAGGAAACGGTATAACTAGAGGATGCGAGGGGTTTCTGGAACTCATAGTGTAGCGGTAAAATGCGTTGATATTATGGGGAACACCAAAAGCGAAGGCATGAAACTAGAGCACTCCTGACACTGAAACAAGAAAGCGTGGGTCGCGAATGGG
## 1047                                                         ACAGAGGGTGCAAGCGTTGTTCGGAATTATTGGGCGTAAAGCGCGTGTAGGCGGCTTGGCAAGTCAGATGTGAAAGCCCATGGCTTAACCATGGAAGTGCGTCTGAAACTGCTGAGCTTGAGTGCCGGAGAGGATGGCGGAATTCCGCAAGTAGAGGTGAAATTCGTAGATATGCGGAGGAACACCGGTGGCGAAGGCGGCCATCTGGACGGCAACTGACGCTGAGACGCGAAAGCGTGGGGAGCAAACAGG
## 1048                                                         ACGGAGGATGCAAGCGTTATCCGGAATCATTGGGTTTAAAGGGTCCGTAGGCGGTTTAATAAGTCAGTGGTGAAAGCCCATCGCTCAACGATGGAACGGCCATTGATACTGTTAAACTTGAATTATTAGGAAGTAACTAGAATATGTAGTGTAGCGGTGAAATGCTTAGAGATTACATGGAATACCAATTGCGAAGGCAGGTTACTACTAATATATTGACGCTGATGGACGAAAGCGTGGGGAGCGAACAGG
## 1049                                                          ACGTAGGGGGCGAGCGTTATCCGGATTTATTGGGCATAAAGAGCATGTAGGCGGTAATTTAAGTCAGGCGTGAAATATTGTGGCCTAACCATAATATGCATTTGATACTGGATTACTAGAGATCGGGAGAGGAAAGTGGAACTCATGGTGTAGCGGTGAAATGCGTAGATATCATGAGGAACACCAGTGGCGAAGGCGGCTTTCTGGACCGATTCTGACGCTGAGATGCGAAAGCGTGGGGAGCAAACAGG
## 1050                                                         ACAGAGGGTGCAAGCGTTAATCAGAATGACTGGGCGTAAAGGGCGTGTAGGTGGTTGACTAGGTTTGATGTGAAATCCCCGGGCTTAACCTGGGAATTGCGTCGAAAACGGGTCGACTCGAGTGAGATAGAGGGTTGTGGAATTTCCGGTGTAGCGGTGAAATGCGTAGATATCGGAAAGAACATCAGTGGCGAAGGCGGCAACCTGGATCTTAACTGACACTGAGGCGCGAAGGCGTGGGGAGCAAACAGG
## 1051                                                         ACGAAGGTCCCTAGCGTTGTTCGGAATCATTGGGCGTAAAGCGCACGTAGGTGGCTTTGCAAGTCAGGTGTGAAAGCCTAGGGCTTAACTCTAGAAGTGCACTTGAAACTGCTTAGCTTGAGTACGGGAGAGGTGAGTGGAATTCCTGGTGTAGTGGTGAAATACGTAGATATCAGGAGGAACACCGGTGGCGAAGGCGGCTCACTGGCCCAGTACTGACACTGAGGTGCGAAAGCATGGGGATCAAACAGG
## 1052                                                         ACAGAGGGTGCAAGCGTTGTTCGGAATTATTGGGCGTAAAGCGCGTGTAGGCGGCTTGGCAAGTCAGATGTGAAAGCCCTCGGCTTAACCGAGGAAGTGCGTCTGAAACTGCTGAGCTTGAGTCTCGGAGAGGATGGCGGAATTCCGCAAGTAGAGGTGAAATTCGTAGATATGCGGAGGAACACCGGTGGCGAAGGCGGCCATCTGGACGAGTACTGACGCTGAGACGCGAAAGCGTGGGGAGCAAACAGG
## 1053                                                         ACGGAGGGTGCAAGCGTTATCCGGATTTATTGGGTTTAAAGGGTCCGTAGGCGGACGATTAAGTCAGCGGTGAAAGCCCACAGCTCAACTGTGGAACTGCCATTGATACTGGTTGTCTTGAGTACGGTTGATGTTGGCGGAATAAGTCATGTAGCGGTGAAATGCATAGATATGACTTGGAACCCCGATTGCGAAGGCAGCCAACAAAGCCGAAACTGACGCTGAGGGACGAAAGCGTGGGTAGCGAACAGG
## 1055                                                        ACAGAGACCCCAAGCGTTATCCGGAATTATTGGGCGTAAAGGGTGAGTAGGTGGTATTATTAGTCGGGTGTTAAAGTTCTCGGGCTCAACCTGAGAAATGCATCCGAAACGGTAAAACTAGAATATTGGAGAGGTGAGTGGAACTCTATGTGTAGGGGTAAAATCCGTTGATATATAGGGGAACACCAAAAGCGAAGGCAGCTCACTGGCCAATTATTGACACTGAATCACGAAAGCGTGGGTAGCGATACGG
## 1056                                                         ACGGAGGGTGCGAGCGTTGTCCGGAATCACTGGGCGTAAAGGGCGCGTAGGTGGTCTGCTAAGCGTGCGGTGAAAGCCTGGGGCTCAACCCCAGGTCGGCCGTGCGAACTGGTGGACTGGAGCACTGTAGAGGCAGGTGGAATTCCGGGTGTAGCGGTGGAATGCGTAGAGATCCGGAAGAACACCGGTGGCGAAGGCGGCCTGCTGGGCAGTTGCTGACACTGAGGCGCGACAGCGTGGGGAGCAAACAGG
## 1057                                                         ACGTAGGGTCCAAGCGTTAATCGGAATTACTGGGCGTAAAGCGTCCGCAGGTGGCTTGATAAGATAGACGTGAAATCCCTGGGCTCAACCTAGGAATTGCGTTTATGACTGTCTCGCTAGAGTATGGGAGAGGGGGGTGGAATTCCACGTGTAGCAGTGAAATGCGTAGAGATGTGGAGGAACACCGATGGCGAAGGCAACCCCCTGGCCTAATACTGACACTCATGGACGAAAGCGTGGGGAGCAAACAGG
## 1058                                                         ACGAGGGGTGCAAGCGTTGCTCGGAATTATTGGGCGTAAAGGGCAGGTAGGTGGTCTTATTTGTCAGGGGTGAAAGCCTTGGGCTTAACCTGAGAAGTGCCCCTGAAACGGTAAGACTAGAGTCTTGGAGAGGGTTGCGGAATTCCCGGTGTAGCGGTGAAATGCGTAGAGATCGGGAGGAACACCAGAGGCGAAAGCGGCAGCCTGGACAAGCACTGACACTCAACTGCGAAAGCGTGGGGAGCAAACAGG
## 1059                                                        ACAGGGGATGCAAGCGTTATCCGGAATTATTGGGCGTAAAGCGTCCTGAGGTGGCTTACCCAGTCGACTGTGAAAGCTCAAGGCTTAACCTTGAAACGGCAGTCGAAACTTGTAAGCTTGAGTACGGTAGGGGCAGAGGGAATTCCCGGAGGAGAGGTGAAATTCGTAGATATCGGGAGGAAGGCCAAAGGCGAAAGCACTCTGCTGGGCCGATTACTGACACTGACAGACGAAAGCTAGGGGAGCGAAAGGG
## 1060                                                         ACGAAGGGTGCAAGCGTTGCTCGGAATTATTGGGCGTAAAGGGCAAGTAGGTGGTCATATAAGTCTGTGGTGAAATCCCTGAGCTCAACTCAGGACGTGCCATGGAAACTATATGACTAGAGTATCGGAGAGGTTCGTGGAATTCCCGGTGTAGCGGTGAAATGCGTAGAGATCGGGAGGAACACCAGAGGCGAAAGCGACGAACTGGACGATGACTGACACTCAATTGCGAAAGCGTGGGTAGCAAACAGG
## 1062                                                         ACGAAGGGGGCTAGCGTTGTTCGGAATTACTGGGCGTAAAGCGCACGCAGGCGGTTCGGTCAGTCAGAAGTGAAAGCCCCGGGCTTAACCTGGGAACTGCTTTTGATACTGCCGAGCTTGAATCACGGAGAGGGTAGTGGAATTCCGAGTGTAGAGGTGAAATTCGTAGATATTCGGAAGAACACCAGTGGCGAAGGCGACTACCTGGCCGTCGATTGACGCTCATGTGCGAAAGCGTGGGGAGCAAACAGG
## 1063                                                         ACGAGGGATCCTAGCGTTGTTCGGAATCATTGGGCGTAAAGCGGGTGTAGGCGGATTTCTAAGTCAGGTGTGAAATCCCAGGGCTCAACCCTGGAAGTGCATTTGATACTGGAAATCTTGAGTGTGGTAGAGGCTACTGGAATTGTTGGTGTAGTGGTGAAATACGTAGATATCAACAGGAATACCGGAGGCGAAGGCGAGTAGCTGGGCCAACACTGACGCTGAGACCCGAAAGCGTGGGGATCAAACAGG
## 1064                                                         ACGGAGGGTGCAAGCGTTGTTCGGAATTACTGGGCGTAAAGCGCGTGCAGGTGGTTTTTTAAGTCTCGTGTGAAATCCCTCGGCTTAACCGAGGAACTGCGCGAGATACTGAGAGACTTGAATACGGGAGAAGAGGGCGGAATTCCCAGTGTAGAGGTGAAATTCGTAGATATTGGGAGGAACACCGGCGGCGAAAGCGGCTCTCTAGACCGATATTGACGCTCATACGCGAGAGCGTGGGTAGCAAACAGG
## 1065                                                         ACGAGTGCTCCAAGCGTTATCCGGAATTATTGGGCGTAAAGGGTGTGTAGGCGGTTTTGTTAGTCGGTTGTTAAAACTCCTCGCTTAACGGGGAAGGTGCAATCGAAACGGCAAATCTAGAGGATGCGAAAGGTAAACAGAACTCATAGTGTAGGGGTGAAATCCGTTGATATTATGGGGAATACCAAATGCGAAGGCAGTTTACTAGCGCACTCCTGACGTTGAAACACGAAAGCGTGGGTAGCGAATGGG
## 1066                                                         ACGAAGGGGGCTAGCGTTGTTCGGAATTACTGGGCGTAAAGCGCACGTAGGCTGTGCGATAAGTCAGGGGTGAAATCCCGGAGCTCAACTCCGGAACTGCCTTTGAAACTATCGTGCTAGAATCTCAAAGGGGATAGCGGAATTCCAAATGTAGGGGTGAAATCCGTAGATATTTGGAGGAACACCGGTGGCGAAGGCGGCTATCTGGGTGAGTATTGACGCTGAGGTGCGAAAGCGTGGGGATCAAACAGG
## 1068                                                         ACGAAGGGGGCTAGCGTTGTTCGGAATTACTGGGCGTAAAGCGCACGTAGGCTGACATTTAAGTCAGGGGTGAAATCCCGAGGCTCAACCTCGGAACTGCCCTTGATACTGGGTGTCTCGAGTCCGGAAGAGGTTAGTGGAATTCCCAGTGTAGAGGTGAAATTCGTAGATATTGGGAAGAACACCAGTGGCGAAGGCGGCTAACTGGTCCGGTACTGACGCTGAGGTGCGAAAGCGTGGGGAGCAAACAGG
## 1069                                                          ACGTATGTCGCAAGCGTTATCCGGAATTATTGGGCATAAAGCGCGTCTAGGCGGTTTACTAAGTCTGATGTGAAAATGCGGAGCTCAACTCCGTATTGCGTTGGAAACTGGTAGACTAGAGTATTGGAGAGGTAGGCGGAACTACAAGTGTAGAGGTGAAATTCGTAGATATTTGTAGGAATGCCAATGACGAAGGTAGCTTACTGGACAAATACTGACGCTGAAGCGCGAAAGCTAGGGGAGCAAACAGG
## 1070                                                         ACGTAGGGTGCGAGCGTTAATCGGAATTACTGGGCGTAAAGCGTGCGCAGGCGGTTCGTTAAGTTTGCGGTGAAAGCCCCGGGCTCAACCTGGGAATGGCCGTGGAAACTGGCGGGCTGGAGTGCGGCAGAGGGGGGTGGAATTCCGCGTGTAGCAGTGAAATGCGTAGAGATGCGGAGGAACACCGATGGCGAAGGCAGCCCCCTGGGCCGACACTGACGCTCAGGCACGAAAGCGTGGGGAGCAAACAGG
## 1071                                                         ACAGAGGGAGCGAGCGTTAACCGGAATGATTGGGCGTAAAGCGTCTGTAGGTGGCTTTTTAAGTCTACTGTCAAAACCCAGAGCTTAACTTTGGATCGGCAGTGGAAACTGAAAAGCTTGAGTGCGGTAGAGGCAGAGGGAATTCCTAGTGGAGCAGTGAAATGCGTAGAGATTAGGAAGAACACCCGTGGCGAAGGCGCTCTGCTGGGCCGTAACTGACACTGAGAGACGAAAGCTAGGGGAGCAAATGGG
## 1072                                                         ACGGAGGATCCGAGCGTTATCCGGATTTATTGGGTTTAAAGGGAGCGTAGGCGGGTTGTTAAGTCAGTTGTGAAAGTTTGCGGCTCAACCGTAAAATTGCAGTTGATACTGGCGACCTTGAGTGCAACAGAGGTAGGCGGAATTCGTGGTGTAGCGGTGAAATGCTTAGATATCACGAAGAACTCCGATTGCGAAGGCAGCTTACTGGATTGTAACTGACGCTGATGCTCGAAAGTGTGGGTATCAAACAGG
## 1073                                                         ACGAGGGGTCCTAGCGTTGTTCGGAATCATTGGGCGTAAAGCGTATGTAGGTGGCCTTATAAGTCAGGTGTGAAAGCCCCGGGCTCAACCCGGGAAGTGCATTTGATACTGTTTGGCTTGAGTACCGAAGAGGATAGTGGAATTCCAGGTGTAGTGGTGAAATACGTAGATATCTGGAGGAACACCGGTGGCGAAGGCGGCTATCTGGTCGAGTACTGACACTGAGATACGAAAGCGTGGGGATCAAACAGG
## 1075                                                         ACGGAGGGTGCAAGCGTTAATCGGAATTACTGGGCGTAAAGCGCACGTAGGCGGTTTTCTAAGTCAGATGTGAAATCCCCGGGCTTAACCTGGGAACTGCACCTGATACTGGAAGACTAGAATGTGGGAGAGGAGAGTGGAATTTCCGGTGTAGCGGTGAAATGCATAGAGATCGGAAGGAACACCAGTGGCGAAGGCGGCTCTCTGGACCAACATTGACGCTGAGGTGCGAAAGCGTGGGTAGCAAACAGG
## 1076                                                         ACGGAGGGTGCAAGCGTTATCCGGATTCACTGGGTTTAAAGGGAGCGTAGGTGGGCAGGTAAGTCAGTGGTGAAATCTCCGGGCTTAACCCGGAAACTGCCATTGATACTATTTGTCTTGAATATTCTGGAGGTAAGCGGAATATGTCATGTAGCGGTGAAATGCTTAGATATGACATAGAACACCCATTGCGAAGGCAGCTTACTACGGAATTATTGACACTGAGGCTCGAAAGCGTGGGGATCAAACAGG
## 1077                                                         ACGGAGGATCCGAGCGTTATCCGGATTTATTGGGTTTAAAGGGAGCGTAGGCGGATGTTTAAGTCAGTTGTGAAAGTTTAAGGCTCAACCTTGAAATTGCAGTTGATACTGGACGTCTTGAGTGCATTAAATGTGGGCGGAATTCGTGGTGTAGCGGTGAAATGCTTAGATATCACGAAGAACTCCAATTGCGAAGGCAGCTCACAGGACTGTAACTGACGCTGATGCTCGAAAGTGTGGGTATCAAACAGG
## 1080                                                         ACGGAGGATCCAAGCGTTATCCGGATTTATTGGGTTTAAAGGGTGCGTAGGCGGGAGATTAAGTCAGTGGTGAAAGTTTGCAGCTCAACTGTAAAATTGCCATTGAAACTGATTTTCTTGAGTGCAAATGAGGTAGGCGGAATGTGTTGTGTAGCGGTGAAATGCTTAGATATAACACAGAACCCCGATTGCGAAGGCAGCTTACTGGGATGCAACTGACGCTGAGGCACGAAAGCGTGGGGATCAAACAGG
## 1082                                                        ACGAAGGGAGCTAGCGTTATCCAAAATGATTGGGCGTAAAGAGTTTGTAGGCGGTATTTGTTGTTATATGTGAAATATTAATTTTATTTTTTAATAATGCATATAATACTCTTTTACTTGAGTATAACAGAGGATAACAGAATTTCATACGGAAGAGTAAAATCTAATGATTTATGAAGGAATGTCAGAGGCGAAAGCGGTTATCTGGGTTATTTACTGACGCTGAGGAACGAAAGCATGGGGAGCAAACAGG
## 1083                                                         ACGAAGGGTGCTAGCGTTGTTCGGAATCACTGGGCGTAAAGCGTACGTAGGCGGTTTAGTAAGTTGATTGTGAAAGCCCCAGGCTTAACTTGGGAACTGCAATCAATACTGCTTTTCTAGAATATGGTGGGGGATAGTGGAATTCCTAGTGTAGGGGTGAAATCCGTAGATATTAGGAGGAATACCGGTGGCGAAAGCGACTATCTATGCCATTATTGACGCTAAGGTACGAAAGCGTGGGGAGCAAACAGG
## 1085                                                         ACAGAGGCCCCGAGCGTTGTTCGGAATTACTGGGCGTAAAGGGAATGTAGGTGGCTATATAAGTCAGATGTGAAATCCCCGGGCTTAACCCGGGAACTGCATTTGATACTGCATAGCTCGAGTGCGGAAGAGGAGAGCGGAATTCCCAGTGTAGGAGTGAAATCCGTAGATATTGGGAGGAATACCGATGGCGAAGGCAGCTCTCTGGTCCGTAACTGACACTGAGATTCGAAAGCGTGGGTAGCAAACAGG
## 1086                                                          ACGTAGGGGGCAAGCGTTGTCCGGAATTATTGGGCGTAAAGCGCGCGCAGGCGGCTTGTTAAGTCTGATGTGAAAGGCAAGGGCTCAACCCTTGTTAGCATTGGAAACTGACAGACTAGAGTGCAGCAGAGGAAAGTGGAATTCCACGTGTAGCGGTGAAATGCGTAGAGATGTGGAGGAACACCAGTGGCGAAGGCGACTTTCTGGGCTGTAACTGACGCTGAGGCGCGAAAGCGTGGGGAGCAAACAGG
## 1088                                                         ACGGAGGGTGCAAGCGTTATCCGGAATCATTGGGTTTAAAGGGTCCGCAGGCGGGCTTATAAGTCAGTGGTGAAAGCCCATCGCTTAACGATGGAACTGCCATTGATACTGTAAGTCTTGAATTCGGTCGAAGTGGGCGGAATGTGACATGTAGCGGTGAAATGCTTAGATATGTCACAGAACACCGATAGCGAAGGCAGCTCACTAGGCCTGGATTGACGCTCATGGACGAAAGCGTGGGGAGCAAACAGG
## 1089                                                         ACAGAGGTCTCAAGCGTTGTTCGGATTCATTGGGCGTAAAGGGTGCGTAGGCGGCGTGGTAAGTTGAGTGTGAAATCCTAGGGCTTAACCTTAGAACTGCACTCAATACTGCCATGCTTGAGGAATGTAGAGGAGAGTGGAATTCACGGTGTAGCAGTGAAATGCGTAGATATCGTGAGGAAGACCAGTTGCGAAGGCGACTCTCTGGGCATTTCCTGACGCTGAGGCACGAAGGCCAGGGGAGCAAACGGG
## 1091                                                         ACGTAGGGTGCAAGCGTTGTCCGGAATTACTGGGTGTAAAGGGAGCGCAGGCGGGAAGACAAGTTGGAAGTGAAAACCATGGGCTCAACCCATGAATTGCTTTCAAAACTGTTTTTCTTGAGTAGTGCAGAGGTAGATGGAATTCCCGGTGTAGCGGTGGAATGCGTAGATATCGGGAGGAACACCAGTGGCGAAGGCGGTCTACTGGGCACCAACTGACGCTGAGGCTCGAAAGCATGGGTAGCAAACAGG
## 1092                                                         ACATAGGGTGCAAGCGTTGTCCGGAATTATTGGGCGTAAAGAGCTCGTAGGTGGTTCGTCACGTCGGATGTGAAACTCTGGGGCTTAACCCCAGACCTGCATTCGATACGGGCGAGCTTGAGTATGGTAGGGGAGTCTGGAATTCCTGGTGTAGCGGTGGAATGCGCAGATATCAGGAGGAACACCAATGGCGAAGGCAGGTCTCTGGGCCATAACTGACACTGAGGAGCGAAAGTGCGGGGAGCGAACAGG
## 1093                                                         ACAGAGGATGCAAGTGTTATCCGGAATTACTGGGCGTAAAGCGTCTGTAGGTGGTTTGATAAGTCAATTGTTAAATCTTGATGCTTAACTTCAAAACTGCAATTGAAACTATTTGACTTGAGTATGGTAGAGGTAAAAGGAATTTTCAGTGGAGCGGTGAAATGCGTAGATATTGGAAAGAACACCGATGGCGAAAGCATTTTACTGGGCCATCACTGACACTCAGAGACGAAAGCTAGGGTAGCAAATGGG
## 1094                                                         ACGGAGGGTGCAAGCGTTATTCGGAGTTACTGGGCGTAAAGGGCGCGTAGGCGGTCTGTTATGTCTGATGTGAAAGCCCTGGACTTAATTCAGGAAGTGCATTGGAAACTGGCAGGCTAGAGTATAGGAGGGGAGAGCGGAATTCCTGGTGTAGAGGTGAAATTCGTAGATATCAGGAGGAACAACAGTGGCGAAGGCGGCTCTCTGGACTATAACTGACGCTGAGGCGCGAAAGCGTGGGTAGCAAACAGG
## 1096                                                         ACGAAGGGTGCAAGCGTTGTTCGGAATCATTGGGCGTAAAGCGCGCGCAGGCGGATTTGCAAGTCAGATGTGAAATCTCGAAGCTCAACTTCGAAACTGCGTCTGAAACTGCTAGTCTAGAATGTCGGAGGGGGCAGGGGAATTTCACGTGTAGGGGTAAAATCCGTAGAGATGTGAAGGAACACCGGAGGCGAAGGCGCCTGCCTGGACGACTATTGACGCTGAGGCGCGAAAGCGTGGGGAGCAAACAGG
## 1097                                                         ACAGGGGGTGCAAGCGTTGTTCGGAATCACTGGGCGTAAAGGGAGCGTAGGCGGAATTGCAAGACAGAGGTTAAATGTCCGGGCTCAACCTGGAACCTGCCTTTGTGACTGCAGTTCTTGAGTATGGCAGAGGCTAGTGGAATTACAGGTGTAGCGGTGGAATGCGTAGATATCTGTAAGAACACCAGAGGCGAAGGCGACTAGCTGGGCCAATACTGACGCTGAGGCTCGAAAGTGTGGGGAGCAAACAGG
## 1099                                                         ACAGGGGATGCAAGTGTTATCCGGAATTATTGGGCGTAAAGCGTCTGCAGGTTGATAATTAAGTCTTTTGTTAAACCTCTGGGCTTAACCCAGAATCTGCAAAAGAAACTATTTATCTTGAGTATGGTAGAGGTAAAGGGAATTTCCAGTGGAGCGGTGAAATGCGTAGAGATTGGAAAGAACACCAACAGCGAAGGCACTTTACTGGGCCAGTACTGACACTCAGAGACGAAAGCTAGGGGAGCAAACAGG
## 1100                                                         ACGAAGGGGGCAAGCGTTGTTCGGAATCACTGGGCGTAAAGCGTGCGTAGGCGGTCTAACAAGTCAGGAGTGAAATCCCCGGGCCTAACCCGGGAACTGCTTTTGAAACTGTTAGACTTGAATCCGGGAGAGGTTGGCGGAATTCTCAGTGTAGAGGTGAAATTCGCAGATATTGAGAGGAACACCAGTGGCGTAAGCGGCCAACTGGACCGGTTTTGACGCTGAGGCACGAAAGCGTGGGGATCAAACAGG
## 1101                                                        ACGAAGGTGGCAAGCGTTGTCCGGTCTTATTGGGCGTAAAGAGTTCTGTAGGCGGAATTATAAGTTATATGTTAAAGGCTACGGCCCAACCGTAGTTTCGCGTGTAATACTGTAGTTCTTGAGTAATGTAGAGGTTAGCAGAACGCACGGTGTAGTAGTGAAATGCGTTGATATCGTGCGGAATACCAAAGGCGTAGGCAGCTAACTGGACATTTACTGACGCTGAGAGACGAAAGCTTGGGGAGCAAAACGG
## 1102                                                         ACGGGGGGTGCAAGCGTTATTCGGAATTATTGGGCGTAAAGGGTGCGTAGGCGGTTTTGTAAGTCAGATGTGAAATACCGGGGCTTAACTTCGGAACTGCATTTGATACTGCGAGACTTGAGTATGGGAGAGGAGAGCGGAATTCCTGGTGTAGAGGTGAAATTCGTAGATATCAGGAGGAACACCGGTGGCGAAGGCGGCTCTCTGGACCAATACTGACGCTGAGGCACGAAGGCGTGGGGAGCAAACAGG
## 1103                                                         ACGAAGGGTGCTAGCGTTGTTCGGAATCACTGGGCGTAAAGCGCGCGTAGGCGGTTATTCAAGTCAGGGGTGAAATCCTGAGGCTCAACCTCAGAATTGCCTTTGAAACTGTATGACTGGAGTGTCGGAGGGGATAGCGGAATTGCTAATGTAGCGGTGAAATGCGTAGATATTAGCAGGAACACCGGTGGCGAAGGCGGCTATCTGGACGACAACTGACGCTGAGGCGCGAAAGCGTGGGTATCAAACAGG
## 1104                                                         ACGGAGGGTGCAAGCGTTGTTCGGAATTACTGGGCGTAAAGCGCGCGTAGGCGGTTTGCTAAGTCAGATGTGAAAGCCCTCGGCTTAACCGGGGACGTGCATTTGAAACTGGCAGACTTGAGTACTGGAGGGGGTGGTGGAATTCCCGGTGTAGAGGTGAAATTCGTAGATATCGGGAGGAATACCGGTGGCGAAGGCGACCACCTGGCCAGATACTGACGCTGAGGTGCGAAAGCGTGGGGAGCAAACAGG
## 1105                                                         ACGTAGGGTGCGAGCGTTAATCGGAATTACTGGGCGTAAAGGGTGCGCAGGCGGTTGAGTAAGACAGATGTGAAATCCCCGAGCTTAACTCGGGAATGGCATATGTGACTGCTCGACTAGAGTGTGTCAGAGGGAGGTGGAATTCCACGTGTAGCAGTGAAATGCGTAGATATGTGGAAGAACACCGATGGCGAAGGCAGCCTCCTGGGACATAACTGACGCTCAGGCACGAAAGCGTGGGGAGCAAACAGG
## 1107                                                         ACAGAGGTCTCAAGCGTTGTTCGGATTCATTGGGCGTAAAGGGTGCGTAGGTGGCGATGTAAGTCGGATGTGAAATCTCCAAGCTTAACTTGGAAACTGCATTCGATACTGCGTTGCTAGAGGACTGTAGAGGGCATTGGAATTCACGGTGTAGCAGTGAAATGCGTAGATATCGTGAGGAAGACCAGTGGCGAAGGCGAATGCCTGGGCAGTTCCTGACACTGAGGCACGAAGGCCAGGGGAGCAAACGGG
## 1109                                                         ACGAAGGGTGCTAGCGTTGTTCGGAATCATTGGGCGTAAAGCGTGCGTAGGCGGCTTAGTAAGTCGTTTGTGAAATACCTAGGCTTAACTTAGGAACTGCAATCGAAACTACTTTGCTAGAGTTTGGTGGGGGATAGTGGAACTCCTAGTGTAGGGGTGAAATCCGTAGAGATTAGGAGGAATATCAGTGGCGAAGGCGACTATCTACACCAATACTGACGCTGAGGTACGAAAGCGTGGGGAGCAAACAGG
## 1110                                                         ACATAGGGTGCAAGCGTTGTCCGGAATTATTGGGCGTAAAGAGCTCGTAGGTGGTTCGTCACGTCGGATGTGAAACTCTGGGGCTTAACCCCAGACCTGCATTCGATACGGGCGAGCTTGAGTATGGTAGGGGAGTCTGGAATTCCTGGTGTAGCGGTGGAATGCGCAGATATCAGGAGGAACACCAATGGCGAAGGCACATCTCTGGGCCGTAACTGACGCTGAGGAGCGAAAGCGTGGGGAGCGAACAGG
## 1111                                                         ACGAAGGATGCAAGCGTTATCCGGATTCATTGGGTTTAAAGGGAGCGTAGGCGGACTTATAAGTCAGTGGTGAAATCTTTGGGCTTAACCCAGAAACTGCCATTGATACTGTAAGTCTAGAGTACAGTTGCCGTTGGCGGAATATGACATGTAGTGGTGAAATACTTAGATATGTCATAGAACACCGATTGCGAAGGCAGCTAACGAAGCTGTAACTGACGCTGAGGCTCGAAAGTGCGGGGATCAAACAGG
## 1113                                                         ACAGGGGATGCAAGTGTTATCCGGAATTATTGGGCGTAAAGCGTCTGTAGGTTGGTAATTAAGTCTTTTGTTAAACCTTCGGGCTCAACCCGAAATCTGCAAAAGAAACTATTTATCTTGAGTATAGTAGGGGTAAAGGGAATTTCCAGTGGAGCGGTGAAATGCGTAGAGATTGGAAAGAACACCAAAAGCGAAGGCACTTTACTGGGCTATAACTGACACTGAGAGACGAAAGCTAGGGGAGCAAACGGG
## 1114                                                         ACGGAGGGGGCTAGCGTTGTTCGGAATCACTGGGCGTAAAGCGCGCGTAGGCGGCTTGATAAGTTAGAAGTGAAATCCCTGGGCTTAACCTAGGAATTGCTTTTAAAACTGTCGAGCTAGAGAATGGTAGAGGAGAGTGGAATTCCTAGTGTAGAGGTGAAATTCGTAGATATTAGGAGGAACACCAGAGGCGAAGGCGGCTCTCTGGGCCATTTCTGACGCTAAGGCGCGAAAGCGTGGGGAGCAAACAGG
## 1116                                                        ACGAACCAAGCGAACGTTATTCGGAATTACTGGGCTTAAAGCGCGTGTAGGCGGATGGCCACGTCGGTTGTTGAAATCCCCCGGCTTAACCGGGGAACAGGCACCGATACGAGTCGTCTAGAGGTGGGTAGGGGAGACTGGAACTTCCGGTGGAGCGGTGAAATGCGTTGAGATCGGAAGGAACGCCTGCGGCGAAAGCGAGTCTCTGGCCCCATACTGACGCTGAGACGCGAAAGCCAGGGGAGCAAACGGG
## 1117                                                         ACGTATGGGGCGAGCGTTGTTCGGAATTATTGGGCGTAAAGGGTGCGCAGGCGGTTGTACAAGTCTGGTGTTAAAGCCCGCAGCTTAACTGCGGAAAGGCATTGGAAACTGTACGACTAGAGTATTGGAGGGGTAGCTGGAATTCCAGGTGTAGGGGTGAAATCTGTAGATATCTGGAAGAACATCAATGGCGAAGGCAAGCTACTAGCCGCATACTGACGCTGAGGCACGAAAGCGTGGGGAGCAAACAGG
## 1118                                                         ACGTAGGGTGCGAGCGTTAATCGGAATTACTGGGCGTAAAGCGTGCGCAGGCGGTTATACAAGACAGGCGTGAAATCCCCGGGCTTAACCTGGGAATGGCGCCTGTGACTGTATAGCTAGAGTACGGTAGAGGGGGATGGAATTCCGCGTGTAGCAGTGAAATGCGTAGATATGCGGAGGAACACCGATGGCGAAGGCAATCCCCTGGACCTGTACTGACGCTCATGCACGAAAGCGTGGGGAGCAAACAGG
## 1121                                                         ACGTAGGATCCAAGCGTTGTCCGGATTTATTGGGTTTAAAGGGTGCGTAGGCGGAAAGATAAGTCAGTGGTGAAAGCCGGTCGCTCAACGATCGAATTGCCATTGATACTGTTTTTCTAGAATACGGTTGAGGTAGGCGGAATGTGTAGTGTAGCGGTGAAATGCATAGATATTACACAGAACTCCGATTGCGAAGGCAGCTTACTAAGCCGTTATTGACGCTGAGGCACGAAAGCGTGGGGATCAAACAGG
## 1122                                                         ACGAAGGATGCAAGCGTTATCCGGATTCATTGGGTTTAAAGGGTGCGTAGGCGGATTAGTAAGTCAGTGGTGAAATACCATCGCTTAACGATGGGACTGCCATTGATACTGCTGATCTTGAGTACATATGACGTTGGCGGAATATGACATGTAGCGGTGAAATGCATAGATATGTCATAGAACACCGATTGCGAAGGCAGCTAACGAAAATGTAACTGACGCTGATGCACGAAAGTGCGGGGATCAAACAGG
## 1123                                                         ACGGAGGATCCAAGCGTTATCCGGATTTATTGGGTTTAAAGGGTGCGTAGGCGGACTGTTAAGTCAGTGGTGAAAGGTTGACGCTCAACGTTAACATTGCCATTGAAACTGGTAGTCTTGAGTGCAAATGAGGCAGGCGGAATGTGTTGTGTAGCGGTGAAATGCTTAGATATGACACAGAACACCAATTGCGAAGGCAGCTTGCTGGAATGTAACTGACGCTGAGGCACGAAAGCGTGGGGATCAAACAGG
## 1124                                                         ACGTAGGGTGCAAGCGTTATTCGGAATTATTGGGCGTAAAGCGCGTGTAGGCGGTCTAGTTAGTCTAATGTGAAATCCCTCGACTCAATCGAGGAACTGCATCGGATACTGCTAGACTAGAGTACAGGAGGGGTGAGCGGAATTCCTGGTGTAGAGGTGAAATTCGTAGATATCAGGAGGAACAACAGCGGCGAAGGCGGCTCACTGGACTGTAACTGACGCTCAGACGCGAAAGCGTGGGGAGCAAACAGG
## 1125                                                         ACGGAGGGTGCGAGCGTTATCCGGAATTACTGGGTTTAAAGGGTGCGTAGGCGGATGATTAAGTCTAAGGTGAAAGTTTGTCGCTTAACGATAAAATTGCCTTGGATACTGGTTATCTTGAATAAGGATGAGGATGGCGGAATGTGGCATGTAGCGGTGAAATGCATAGATATGCCATAGAACACCAATTGCGAAGGCAGCTATCTGGTCTTTTATTGACGCTGAGGCACGAAAGCGTGGGGATCGAACAGG
## 1126                                                         ACGAAGGGGGCAAGCGTTGTTCGGAATTACTGGGCGTAAAGGGAGTGTAGGCGGTTATGTAAGATAGTGGTGAAATCCCAGAGCTTAACTTTGGAATTGCCATTATGACTATGTGGCTAGAATTACAGAGAGGATAGTGGAATACCCAGTGTAGAGGTGAAATTCGTAGATATTGGGTAGAACACCAGTGGCGAAGGCGACTATCTGGCTGTATATTGACGCTGAGGCTCGAAAGCATGGGGATCAAACAGG
## 1127                                                          ACGTAGGCGGCAAGCGTTGTCCGGAATTATTGGGCGTAAAGGGAGCGCAGGCGGGAAACTAAGCGGATCTTAAAAGTGCGGGGCTCAACCCCGTGATGGGGTCCGAACTGGTTTTCTTGAGTGCAGGAGAGGAAAGCGGAATTCCCAGTGTAGCGGTGAAATGCGTAGATATTGGGAAGAACACCAGTGGCGAAGGCGGCTTTCTGGACTGTAACTGACGCTGAGGCTCGAAAGCTAGGGTAGCGAACGGG
## 1128                                                         ACATAAGGTGCGAGCGTTATCCGGAATCACTGGGCGTAAAGGGCATCTAGGCGGTTTATTAAGTCAGGGGTGAAAAGCCGAAGCTCAACTTTGGTCTTGCCTTTGAAACTGATAGACTAGAATACGGGATAGGAGGGCGGAACTACAAGTGTAGAGGTGAAATTCGTAGATATTTGTAGGAATGCCGATGACGAAGGTAGCTCTCTGGACCGATATTGACGCTGAAGTGCGAAAGCTAGGGTAGCAAACAGG
## 1130                                                         ACGAGGGATCCTAGCGTTGTTCGGAATCATTGGGCGTAAAGCGGGTGTAGGTGGCTTTTTAAGTCAGGTGTGAAATCCTAGGGCTTAACCCTAGAAGTGCATTTGATACTATTAAGCTTGAGTGTGGAAGAGGCTATTAGAATTCCTGGTGTAGTGGTGAAATACGTAGATATCAGGAGGAATACCGGTTGCGAAGGCGGATAGCTGGTCCAACACTGACACTAAGACCCGAAAGCGTGGGGATCAAACAGG
## 1133                                                         ACGGAGGATGCAAGCGTTATCCGGATTCATTGGGTTTAAAGGGTGCGTAGGCGGCTTTTTAAGTCAGTGGTGAAATCCTGCCGCTTAACGGTAGAATTGCCATTGATACTGAAAAGCTTGAGTACAGTTGAGGTAGGCGGAATGTGTAGTGTAGCGGTGAAATGCTTAGATATTACACAGAACACCGATTGCGAAGGCAGCTTACTAAACTGTTACTGACGCTGATGCACGAAAGCGTGGGGAGCGAACAGG
## 1134                                                        ACGAACCAAGCGAACGTTATTCGGAATTACTGGGCTTAAAGCGCGTGTAGGCGGATTGCCACGTCGGCTGTTGAAATCCCCCGGCTCAACCGGGGAACAGGCACCGATACGAGTGATCTTGAGGTGGGTAGGGGAGACTGGAACTTCCGGTGGAGCGGTGAAATGCGTTGAGATCGGAAGGAACGCCCGCGGCGAAAGCGAGTCTCTGGCCCCATACTGACGCTGAGACGCGAAAGCCAGGGGAGCAAACGGG
## 1136                                                         ACGGAGGATCCGAGCGTTATCCGGATTTATTGGGTTTAAAGGGAGCGTAGGTGGACTGGTAAGTCAGTTGTGAAAGTTTGCGGCTCAACCGTAAAATTGCAGTTGATACTGTCAGTCTTGAGTACAGTAGAGGTGGGCGGAATTCGTGGTGTAGCGGTGAAATGCTTAGATATCACGAAGAACTCCGATTGCGAAGGCAGCTCACTAGACTGCAACTGACACTGATGCTCGAAAGTGTGGGTATCAAACAGG
## 1137                                                          ACATATGTCGCAAGCGTTATCCGGATTTATTGGGCGTAAAGCGCGTCTAGGCGGTTTATTAAGTCTGATGTGAAAATGCGGGGCTCAACTCCGTATTGCGTTGGAAACTGGTAGACTAGAGTACTGGAGAGGTGGGCGGAACTACAAGTGTAGAGGTGGAATTCGTAGATATTTGTAGGAATGCCGATGGAGAAGTCAGCTCACTGGACAGATACTGACGCTAAAGCGCGAAAGCGTGGGGAGCAAACAGG
## 1138                                                         ACGTAGGTCCCAAGCGTTATCCGGATTTACTGGGTGTAAAGAGTCTGTAGCAGGTTCTATGAGTCTATTTTTAAAGACCTCGGCTCAACTGAGGGAAGGGAATGGATACTGTAGAACTGGAGGTTATGAGGGGTTAGTAGAATTCCCGGTGGAGGGGTGAAATCCGTTGATATTGGGAGGAATGCCGAAAGCGAAAGCAGCTAACTATTATTATCCTGACTGTGAGAGACGAAAGCATGGGTAGCAAACGGG
## 1139                                                         ACAGAGACCTCAAGCATTATCCGGATTTATTGGGCGTAAAGGGTCCGCAGGTGGTTTGGTGCGTCATGGGTTAAATCTACGAGCTCAACTCGTGGACCGCTCGTGATACGACCAAACTCGAGGCCGGGAGAGGTAAGCGGAATTACGGGTGTAGCAGTAAAATGCGTTAATATCCGTAAGAACACCAAATGCGAAGGCAGCTTACTGGAACGGTTCTGACACTCAGGGACGAAAGCGTGGGTAGCGAAAGGG
## 1141                                                         ACATGTGGGGCAAGCGTTGTTCGGAATTACTGGGCATAAAGGGTGCGTAGGCGGTCTGATAAGTCGGGCGTGAAATCCGGTGGCCCAACCATCGAACTGCGCTTGATACTGTTGGACTTGAGTGCGTGAGGGGAGACTGGAACGAGTGGTGTAGCGGTGAAATGCGTAGATATCACTCGGAACGCCAACGGCGAAGGCAGGTCTCTGGTACGCAACTGACGCTGAGGCACGAAAGCGTGGGGAGCAAACAGG
## 1142                                                         ACGTAGGGGGCGAGCGTTGTTCGGATTCACTGGGTGTAAAGGGCGTGTAGGTGGTTATTTAAGTCGAATGTGAAATCCCTTGGCTCAACCAAGGAAGTGCATTCGAAACTAAGTGACTAGAGTTTGGGAGAGGAGAGCGGAATTCTCGGTGTAGCGGTGAAATGCGTAGAGATCGAGAAGAACACCAGCGGCGAAAGCGGCTCTCTGGACCAATACTGACACTGAGGCGCGAAAGCGTGGGGAGCAAACGGG
## 1143                                                         ACAGAGGGTGCGAGCGTTAATCGGAATTACTGGGCGTAAAGCGCGCGTAGGCGGCTTGTTAAGTCGGATGTGAAATCCCTGGGCTCAACCTAGGAACTGCATTCGATACTGGCAAGCTAGAGTGTGGGAGAGGAAGGTAGAATTCCAGGTGTAGCGGTGAAATGCGTAGAGATCTGGAGGAATACCGATGGCGAAGGCAGCCTTCTGGCCTAACACTGACGCTGAGGTGCGAAAGCATGGGGAGCAAACAGG
## 1144                                                         ACGAAGGGTGCAAGCGTTGTTCGGATTTATTGGGCGTAAAGCGCACGTAGGTGGACTTATAAGTCAGATGTGAAATCTCGGGGCTCAACCTCGAAACTGCGTCTGAAACTGTGAGTCTAGAATCTTGGAGGGGGTAGGGGAATTTCACATGTAGGGGTAAAATCCGTAGATATGTGAAGGAACACCAGAGGCGAAGGCGCCTGCCTGGACAAGTATTGACACTGAGGTGCGAAAGCGTGGGGATCAAACAGG
## 1145                                                         ACGGAGGGTGCAAGCGTTATCCGGAATCATTGGGTTTAAAGGGTCCGTAGGCGGATAATTAAGTCAGAGGTGAAATCCCACAGCTTAACTGTGGAACTGCCTTTGATACTGGTTGTCTTGAATTATAGTGAAGTAGATAGAATGTGTAGTGTAGCGGTGAAATGCTTAGATATTACACAGAATACCGATTGCGAAGGCAGTTTACTAACTATATATTGACGCTAATGGACGAAAGCGTGGGGAGCGAACAGG
## 1146                                                         ACGGAGGGTGCAAGCGTTACTCGGAATCACTGGGCGTAAAGGATGCGTAGGCTGTAATATAAGTCAGGAGTGAAATCCAACGGCTCAACCGTTGAACTGCTCTTGAAACTGTTTTACTAGAATATGGGAGAGGTAGATGGAATTGGTGGTGTAGGGGTAAAATCCGTAGATATCACCAGGAATACCGATTGCGAAGGCGATCTACTGGAACATTATTGACGCTGAGGCATGAAAGCGTGGGGAGCAAACAGG
## 1147                                                         ACGTAGGGGGCAAGCGTTATCCGGATTTACTGGGTGTAAAGGGAGCGTAGACGGCGATGCAAGTCCGATGTGAAAACCCATGGCTCAACCATGGGATTGCATTAGAAACTGTATTGCTGGAGTGCAGGAGAGGTAAGCGGAATTCCTGGTGTAGCGGTGAAATGCGTAGATATCAGGAGGAACACCGGTGGCGAAGGCGGCTTACTGGACTGTAACTGACGTTGAGGCTCGAAGGCGTGGGGAGCAAACAGG
## 1148                                                         ACGGAGGATCCAAGCGTTATCCGGATTTATTGGGTTTAAAGGGTCCGTAGGCGGGTCTTTAAGTCAGTGGTGAAAGCCTGCAGCTTAACTGTAGAATTGCCATTGATACTGGAGACCTTGAGTGTAGTAGAAGTAGGCGGAATAAGGCATGTAGCGGTGAAATGCATAGATATGCCTTAGAACACCGATTGCGAAGGCAGCTTACTATGTTACAACTGACGCTGAGGGACGAAAGCGTGGGGAGCGAACAGG
## 1150                                                         ACGAAGGCCCCAAGCGTTATCCGGAATTACTGGGCGTAAAGCGTCTGTAGGTGGTTTGGAAAGTCTCAAGTGAAAGGTCAGGGCTCAACCCTGTACTTGCTTGGGAAACTATCGAACTAGAGTGTGGGAGAGGCAAGCAGAACGGTATGAGTAGGGGTGCAATCCGTTGATACATACCAGAATACCAAAAGCGAAGGCAGCTTGCTGGAACATTACTGACACTGAGAGACGAAAGCGTGGGGAGCAAAAGGG
## 1151                                                         ACGGGGGGTGCAAGCGTTACTCGGAATCACTGGGCGTAAAGAGCATGTAGGCGGATTAATAAGTTTGAAGTGAAATCCTATAGCTTAACTATAGAACTGCTTTGAAAACTGTTAATCTAGAATGTGGGAGAGGTAGATGGAATTTCTGGTGTAGGGGTAAAATCCGTAGAGATCAGAAGGAATACCGATTGCGAAGGCGATCTACTGGAACAATATTGACGCTGAGACGCGAAAGCGTGGGGAGCAAACAGG
## 1152                                                         ACGGAGGGTGCAAGCGTTATCCGGAATCATTGGGTTTAAAGGGTCCGCAGGCGGGCGTATAAGTCAGTGGTGAAAGCCTATTGCTTAACAATAGAACTGCCATTGATACTGTACGTCTTGAATTCGGTCGAAGTGGGCGGAATATGACATGTAGCGGTGAAATGCTTAGATATGTCATAGAACACCGATAGCGAAGGCAGCTCACTAGGCCTGAATTGACGCTCATGGACGAAAGCGTGGGGAGCAAACAGG
## 1153                                                         ACGTAGGGTGCGAGCGTTAATCGGAATTACTGGGCGTAAAGGGTGCGTAGGCGGGAGTATAAGTTAGATGTGAAATCCCTGAGCTTAACTTAGGAATAGCATTTAAGACTGTACACCTAGAGTTTATCAGAGGGGGGTAGAATTCCAAGTGTAGCAGTGAAATGCGTAGAGATTTGGAGGAATACCGATGGCGCAGGCAGCCCCCTGGGATGAGACTGACGCTGAGGCACGAAAGCGTGGGTAGCAAACAGG
## 1154                                                         ACGGAGGGTGCAAGCGTTATTCGGAGTTACTGGGCGTAAAGGGCGCGTAGGCGGCTCGTTAAGTCTGATGTGAAAGCCCTGGACTCAATCCAGGAAGTGCATTGGATACTGGCGAGCTAGAGTGCAGGAGGGGAGAGCGGAATTCCTGGTGTAGAGGTGAAATTCGTAGATATCAGGAGGAACAACAGTAGCGAAGGCGGCTCTCTGGACTGTAACTGACGCTGAGGCGCGAAAGCGTGGGTAGCAAACAGG
## 1155                                                         ACGAGGGGTGCAAGCGTTATTCGGAATAACTGGGCGTAAAGAGCGCGTAGGCGGCTTGTTAAGTCTGTTGTTAAATTGTCTGGCCTAACCAGATGTCGGCAATAGAAACTGTCAAGCTAGAGGACAAGAGAGAGGGGTGGAATTCTCGGAGTAGCGGTAAAATGCGTAGATCTCGAGAGGAACACCAATGGCGAAGGCAGCCCCTTGGCTTGTATCTGACGCTCAAGTGCGAAAGCGTGGGGAGCAAACAGG
## 1157                                                         ACGTAGGGTGCAAGCGTTAATCGGAATTACTGGGCGTAAAGGGTGCGCAGGCGGTATTGTAAGCCAGATGTGAAATCCCCGGGCTCAACCTGGGAACTGCGTTTGGAACTGCAATGCTAGAGTGTGTCAGAGGGGGGTGGAATTCCACGTGTAGCAGTGAAATGCGTAGAGATGTGGAGGAACACCGATGGCGAAGGCAGTCCCCTGGGATAAGACTGACGCTCATGCACGAAAGCGTGGGGAGCAAACAGG
## 1158                                                         ACGGAGGGTGCAAGCGTTAATCGGAATCACTGGGCGTAAAGCGCGCGTAGGCCGCCTTCTAAGTCGGACGTGAAAGCCCTCGGCTCAACCGAGGAACTGCGTTCGAAACTGGGAGGCTTGAGTCCTGGAGAGGGTGGCGGAATTCCGGGTGTAGGAGTGAAATCCGTAGATATCCGGAGGAACACCGGTGGCGAAGGCGGCCACCTGGACAGGTACTGACGCTGAGGCGCGAAAGCGTGGGGAGCAAACAGG
## 1161                                                         ACGAAGGGTGCAAGCGTTGTTCGGAATTATTGGGCGTAAAGGGTTCGTAGGTTGGAGAGTAAGTCAAGTGTGAAATCCCCGAGCTTAACTTGGGACGTGCACCTGAAACTGCTTTTCTTGAATGTTAGAGAGGGTCGTAGAATTGCTGGTGTAGGAGTGAAATCCGTAGATATCAGCAGGAATACCAGAGGCGAAGGCGGCGACCTGGCTAAACATTGACACTGAGGAACGAAAGCGTGGGGAGCAAACAGG
## 1163                                                         ACGGAGGGTGCGAGCGTTAATCGGAATTACTGGGCGTAAAGCGCGCGTAGGCGGTTTGATCAGTCCGTCGTGAAAGCCCCGGGCTTAACCTGGGAACTGCGGTGGATACTGTCGGGCTAGAGTGTGGTAGAGGGGAGTGGAATTTCCGGTGTAGCAGTGAAATGCGTAGAGATCGGAAGGAACACCAGTGGCGAAGGCGGCTCCCTGGACTAACACTGACGCTGAGGTGCGAAAGCGTGGGGAGCAAACAGG
## 1164                                                         ACGAAGGGTGCAAGCGTTGTTCGGAATTATTGGGCGTAAAGCGCGCGCGGGCGGATAGGCAAGTCAGATGTGAAATCTCGAGGCTCAACTTCGAAACTGCGTCTGAAACTACCAATCTAGAATGTCGGAGGGGGAAGGGGAATATCGCATGTAGGGGTAAAATCCGTAGAGATGTGATGGAACACCAGAGGGGAAGCCGCCTTCCTGGACGAATATTGACGCTTAGGCGCGAAAGCGTGGGGAGCAAACAGG
## 1168                                                         ACGAAGGGGGCTAGCGTTGTTCGGAATTACTGGGCGTAAAGCGAGCGTAGGTTGCTCGATAAGTCAGTGGTGAAAGCCCAGAGCTCAACTCTGGAACTGCCATTGAAACTGTCGAGCTGGAATCTCAGAGGGGGTAGCGGAATTCCAAATGTAGGGGTGAAATCCGTAGATATTTGGAGGAACACCGGTGGCGAAGGCGGCTACCTGGATGAGTATTGACACTGAGGCTCGAAAGCGTGGGGATCAAACAGG
## 1169                                                         ACGTAGGGTGCAAGCGTTGTCCGGATTTATTGGGCGTAAAGAGCTCGTAGGCGGTTAGACACGTCGGATGTGAAAACTGGGGGCTCAACTCCCAGCCTGCATTCGATACGGTCTGACTAGAGTGTGGTAGGGGTAACTGGAATTCCTGGTGTAGCGGTGAAATGCGCAGATATCAGGAGGAACACCGATGGCGAAGGCAGGTTACTGGGCCACCACTGACGCTGAGGAGCGAAAGCGTGGGGAGCAAACAGG
## 1171                                                         ACATAGGGTGCAAGCGTTGTCCGGATTTATTGGGCGTAAAGAGTTCGTAGGCGGTTTCTCGCGTCGGATGTGAAAACTCAGGGCTCAACCCTGAGCCTGCATTCGATACGGGGAAACTTGAGGACGGCAGGGGAGACTGGAACTCCTGGTGTAGCGGTGGAATGCGCAGATATCAGGAAGAACACCAATGGCGAAGGCAGGTCTCTGGGCCGATTCTGACGCTGAGGAACGAAAGCGTGGGGAGCAAACAGG
## 1173                                                         ACAGAGGTCTCAAGCGTTGTTCGGAATCACTGGGCGTAAAGGGTGCGTAGGCGGCTTGGTAAGTCAGATGTGAAAGCTCAGGGCTCAACCCTGAAATTGCATCCGATACTGCCAGGCTAGAGGACTGGAGAGGTGTCTAGAATTCTCGGTGTAGCAGTGAAATGCGTAGATATCGAGAGGAATACCAATGGCGAAGGCAGGACACTGGACAGTATCTGACGCTGAGGCACGAAGGCTAGGGTAGCGAACGGG
## 1174                                                         ACGGGAGTGGCAAGCGTTATCCGGAATTATTGGGCGTAAAGCGTCCGCAGGCGGCCTTGTAAGTCTGCTGTCAAAGCGTGGAGCTTAACTCCATTTCGGCAGTGGAAACTACAAGGCTTGAGTGTGGTAGGGGCAGAGGGAATTCCCGGTGTAGCGGTGAAATGCGTAGATATCGGGAAGAACACCAGTGGCGAAGGCGCTCTGCTGGGCCATAACTGACGCTCATGGACGAAAGCCAGGGGAGCGAAAGGG
## 1176                                                         ACGAAGGTGGCAAGCGTTACTCGGAATTACTAGGCGTAAAGGGCAGGTAGGTGGTTTAATAAGTCTGTTGTGTCAGCTCCTGGCTTAACTGGGAGAGGTCAACGGAAACTATTAGGCTTGAGTATGGGAGAGGGTGCTGGAATTCCTGGTGTAGCGGTGAAATGCGCAGATATCAGGAGGAACACCAATGGCGAAAGCAAGCACCTGGACCATTACTGACGCTAAGCTGCGAAAGCTAGGGGAGCAAACAGG
## 1177                                                         ACGTAGGGTGCGAGCGTTAATCGGAATTACTGGGCGTAAAGCGTGCGCAGGCGGTTTCGTAAGACAGAGGTGAAATCCCCGGGCTCAACCTGGGAACTGCCTTTGTGACTGCGAGGCTAGAGTATGGCAGAGGGGGGTGGAATTCCACGTGTAGCAGTGAAATGCGTAGAGATGTGGAGGAACACCGATGGCGAAGGCAGCCCCCTGGGTCGACACTGACGCTCATGCACGAAAGCGTGGGGAGCAAACAGG
## 1178                                                        ACGTAGGCACCAAGCGTTGTCCGGATTTATTGGGCGTAAAGAGCTCGTAGGCGGTTCAGTAAGTCGGGTGTGAAAACTCTGGGCTCAACCCAGAGACGCCACTCGATACTGCTGTGACTAGAGTGCGGTAGGGGAGCGGGGAATTCCTAGTGTAGCGGTGAAATGCGCAGATATTAGGAGGAACACCAGTGGCGAAGGCGCCGCTCTGGGCCGTAACTGACGCTGAGGAGCGAAAGCGTGGGGAGCAAACAGG
## 1180                                                        ACAGAGACCCCAAGCGTTATCCGGAATTATTGGGCGTAAAGGGTGAGTAGGTGGTTTTATTAGTCTTGTTTTAAAGATTTTGGGCTTAACCTAAAATATGAATAAGAAACGGTAGAACTAGAATTTTGGAGAGGTTAGTGGAACTCTATGTGTAGGGGTAAAATCCGTTGATATATAGGGGAACACCTAAAGCGAAGGCAGCTAACTGGCCAAACATTGACACTGAATCACGAAAGCGTGGGTAGCGATACGG
## 1181                                                         ACAGAGGTCTCAAGCGTTGTTCGGAATCACTGGGCGTAAAGGGTGCGTAGGTGGCGTGGTAAGTCAGATGTGAAAGCCCGGGGCTCAACTCCGGAACTGCATCCGATACTGCCGTGCTAGAGCACTGAAGAGGTGACCGGAATTCTCGGTGTAGCAGTGAAATGCGTGGATATCGAGAGGAACACCAAAGGCGAAGGCAGGTCACTGGGCAGTTGCTGACACTGAGGCACGAAGGCCAGGGTAGCGAACGGG
## 1182                                                         ACGTAGGGTGCGAGCGTTAATCGGAATTACTGGGCGTAAAGCGTGCGCAGGCGGTTTCGTAAGACAGAGGTGAAATCCCCGGGCTCAACCTGGGAACTGCCTTTGTGACTGCGAGGCTAGAGTATGGCAGAGGGGGGTGGAATTCCACGTGTAGCAGTGAAATGCGTAGAGATGTGGAGGAACACCGATGGCGAAGGCAGCCCCCTGGGCCAATACTGACACTGAGGAGCGAAAGCGTGGGGAGCGAACAGG
## 1185                                                         ACGTAGGGTGCAAGCGTTAATCGGAATTACTGGGCGTAAAGCGTGCGTAGGCGGCTATTCAAGTCAGAGGTGAAAGCCTGGAGCTCAACTCCAGAACTGCCTTTGAAACTAGATAGCTAGAATCTTGGAGAGGTGAGTGGAATTCCGAGTGTAGAGGTGAAATTCGTAGATATTCGGAAGAACACCAGTGGCGAAGGCGACTCACTGGACAAGTATTGACGCTGAGGTACGAAAGCGTGGGGAGCAAACAGG
## 1186                                                         ACGGGGGGTGCAAGCGTTACTCGGAATCACTGGGCGTAAAGAGCATGTAGGCGGGTAAATAAGTTGGAAGTGAAATCCTATAGCTTAACTATAGAACTGCTTCCAAAACTGTTTACCTAGAATATGGGAGAGGTAGATGGAATTTCTGGTGTAGGGGTAAAATCCGTAGAGATCAGAAGGAATACCGATTGCGAAGGCGATCTACTGGAACATTATTGACGCTCAGATGCGAAAGCGTGGGGAGCAAACAGG
## 1187                                                         ACGGAAGGTCCGGGCGTTATCCGGATTTATTGGGTTTAAAGGGAGCGTAGGCCGCCTTATAAGCGAGCAGTGAAATGTAGAGGCCCAACCTCTGAATTGCTGTTCGAACTGTAGGGCTTGAGTACGCACGAGGTAGGCGGAATTTGTGGTGTAGCGGTGAAATGCTTAGATATCACAAAGAACCCCGATTGCGAAGGCAGCTTACCGGAGCGCAACTGACGCTGAAGCTCGAAAGTGCGGGTATCGAACAGG
## 1188                                                         ACGTGGGGTGCAAGCATTAATCGGATTTATTGGGCGTAAAGGGTGCGTAGGCGGATCAGAAAGTGGGATGTGAAATCCCGGGGCTTAACCCCGGAACTGCATTCCAAACTCCTGATCTTGAGGGTAGACGGGGAGAATAGAATTCCACATGTAGCGGTGAAATGCGTAGATATGTGGAAGAATACCGGTGGCGTAGGCGATTCTCTAGTTTATACCTGACGCTGAGGCACGAAAGCTGGGGGAGCAAACAGG
## 1189                                                         ACGTAGGGTGCGAGCGTTAATCGGAATTACTGGGCGTAAAGCGTGCGCAGGCGGTTTCGTAAGACAGACGTGAAATCCCCGGGCTCAACCTGGGAACTGCGTTTGTGACTGCGAGGCTAGAGTATGGCAGAGGGGGGTGGAATTCCACGTGTAGCAGTGAAATGCGTAGAGATGTGGAGGAACACCGATGGCGAAGGCAATCCCCTGGACCTGTACTGACGCTCATGCACGAAAGCGTGGGGAGCAAACAGG
## 1190                                                         ACAGAGGATGCAAGTGTTATCCGGAATTACTGGGCGTAAAGCGTCTGTAGGTGGTTTACTAAGTCGATTGTTAAAGCTTGAGGCTTAACTTCAAAACAGCAATTGAAACTAGTAGACTTGAGTATGGTAGAGGTAAAAGGAATTTTCAGTGGAGCGGTGAAATGCGTAGATATTGGAAAGAACACCGATGGCGAAAGCATTTTACTGGACCATAACTGACACTCAGAGACGAAAGCTAGGGTAGCAAATGGG
## 1192                                                         ACGAACTGTGCAAACGTTATTCGGAATCACTGGGCTTGAAGAGTGCGTAGGCGGTTTTGTAAGTAGGGTGTGAAAGCCCCCAGCTCAACTGGGGAATTGCGCCCTAAACTACAAGGCTGGAGTGAGGTAGGGGTGTGTGGAACTTCCAGTGGAGCGGTGAAATGTGTTGATATTGGAAGGAACGCCGGTGGCGAAAGCGACACACTGGGCCTTGTCTGACGCTGAGGCACGAAAGCCAGGGGAGCAAACGGG
## 1195                                                         ACGAACCGTGCGAACGTTGTTCGGAATCATTGGGCTTAAAGGGCGCGTAGGCGGGTGTTCAAGTCCGGGGTGAAAGCCTCCAGCTCAACTGGAGAAGAGCCCCGGATACTGTTCATCTGGAGAGGGATAGGGGCACATGGAACTTCCGGTGGAGCGGTGAAATGCGTAGAGATCGGAAGGAACGCCGGTGGCGAAAGCGATGTGCTGGATCTTTTCTGACGCTGAGGCGCGAAAGCTAGGGGAGCAAACGGG
## 1196                                                         ACGAAGGGTGCAAACGTTGCTCGGAATCATTGGGCGTAAAGCGCACGTAGGCGGCTTTCTAAGTCGGATGTGAAATCCCTCGGCTTAACCGAGGACGTGCATCCGAAACTGGGAGGCTTGAGTATGGAAGAGGGTCGCAGAATTCCCGGTGTAGAGGTGAAATTCGTAGATATCGGGAGGAATACCAGTGGCGAAGGCGGCGACCTGGGCCAATACTGACGCTGAGGTGCGAAAGCGTGGGGAGCAAACAGG
## 1198                                                         ACGGAAGGTCCGGGCGTTATCCGGATTTATTGGGTTTAAAGGGAGCGCAGGCCGGAAGCAAAGCGTGCCGTGAAATGTAGATGCTCAACATCTGAATTGCGGCGCGAACTGGTTTTCTTGAGTGCATACGAGGTAGGCGGAATTTGTGGTGTAGCGGTGAAATGCTTAGATATCACGAAGAACTCCGATTGCGAAGGCAGCCTACCAGGATGTAACTGACGCTAAAGCTCGAAAGTGCGGGTATCGAACAGG
## 1199                                                         ACGGAGGATCCGAGCGTTATCCGGATTTATTGGGTTTAAAGGGTGCGTAGGCGGAATAGCAAGTCAGTGGTGAAAGTTTGCAGCTTAACTGTAAAATTGCCATTGAAACTGCTGTTCTTGAGTACAGTAGAGGTAGGCGGAATGTGTAGTGTAGCGGTGAAATGCATAGATATTACACAGAACACCGATTGCGTAGGCAGCTTACTGGAGTGTAACTGACGCTGAGGCACGATAGCGTGGGGATCAAACAGG
## 1200                                                         ACAGAGGGTGCAAGCGTTGCTCGGAATCATTGGGCGTAAAGCGCGTGTAGGCGGTTTATTAAGTCTGGTGTGAAAGCCCGGGGCTCAACCCCGGAAGTGCACTGGATACTGGTAAACTAGAGGACGAGAGAGGATGGTGGAATTCCTGGTGTAGGGGTGAAATCCGTAGATATCAGGAGGAACATCGGTGGCGAAGGCGGCCATCTGGCTCGTTTCTGACGCTCAGACGCGAAAGCGTGGGTAGCAAACAGG
## 1202                                                        ACGTAGGGTGCAAGCATTATCCGGATTTACTGGGCGTAAAGAGTTCTGTAGGCGGTAATATAAGTTCAGGGTTAAATCTTCTCGCTCAACGAGAAAACTGCTTTGAATACTGTATTACTAGAGGCATTTAGGGGACAGCGGAACGTATGGTGAAGCAGTGAAATGCGTTGATATCATACGGAACATCAAGGGGGAAGCCAGCTGTCTGGGAATGATCTGACGCTGAGAGACGAAAGCGTGGGTAGCGAAAAGG
## 1206                                                         ACAGAGGGTGCAAACGTTGTTCGGAATTACTGGGCGTAAAGCGCGTGTAGGCGGCCCGACAAGTCGGATGTGAAAGCCCCGGGCTCAACCCGGGAAGTGCATTCGATACTGTCAGGCTGGAGTCCCGGAGAGGATGGTGGAACTCTCGGTGTAGAGGTGAAATTCGTAGATATCGAGAAGAACACCGGCGGCGAAGGCGGCCATCTGGACGGTGACTGACGCTGAGACGCGAAAGCGTGGGGAGCAAACAGG
## 1208                                                         ACAGAGGGTGCAAGCGTTAATCGGAATTACTGGGCGTAAAGGGCGCGTAGGCGGTCAGGTCAGTCGGATGTGAAATCCCCGGGCTCAACCTGGGAATTGCATTCGATACTGCCTGGCTAGAGTGTGGAAGAGGGAAGCGGAATTTCCGGTGTAGCGGTGAAATGCGTAGATATCGGAAGGAACACCAGTGGCGAAGGCGGCTTCCTGGTCCAACACTGACGCTGAGGCGCGAAAGCGTGGGGAGCGAACAGG
## 1209                                                         ACGAAGGATCCAAGCGTTGTCCGGATTTACTGGGTTTAAAGGGTGCGTAGGCGGGCTATTAAGTCAGTGGTGAAAGCCTGTAGCTTAACTATAGAATTGCCATTGAAACTGATAGTCTTGAGTATGTTTGAGGTTACTGGAATATAACATGTAGCGGTGAAATGCTTAGATATGTTATAGAACACCAATTGCGAAGGCAGGTAACTAAGTCATAACTGACGCTGAGGCACGAAAGCGTGGGGAGCAAACAGG
## 1210                                                         ACAGAGACTGCAAGCGTTACTCGGATTCACTGGGCGTAAAGGGAGCGCAGGCGGATTGGTGTGTCAGGCGTGAAATCCCGGGGCTCAACCCCGGGGCTGCGCCCGAAACTACCAATCTAGAGACTTGGAGGGGTAAGCGGAATTCTTGGTGGAGCAGTGAAATGCGTAGATATCAAGAGGAACACCAACGGCGAAGGCAGCTTACTGGACAAGATCTGACGCTCAGGCTCGAAAGCGTGGGGAGCAAAAGGG
## 1211                                                         ACAGAGGCCCCAAGCATTATCCGGAATCACTGGGCGTAAAGGGTGTCAAGGCGGCTATATTAGTCGTTCGTTAAATCCGTGGGCTCAACCTACGGTCTGCGAGCGAAACGGTATAGCTAGAGGTTGGAAGAGGTGCGTGGAACTCACGGTGTAGGGGTGAAATCCGTTGATATCGTGGGGAACACCAAAGGCGAAGGCAGCGCACTGGTCCAAATCTGACGCTCACACACGAAAGCCAGGGTAGCGAACGGG
## 1212                                                         ACAGAGGGTGCAAACGTTGTTCGGAATTACTGGGCGTAAAGCGTGTGTAGGCGGCTAAGTAAGTCAGATGTGAAAGCCCCGGGCTCAACCCGGGAAGTGCATTTGATACTGCCTAGCTTGAGTATCGGAGAGGTTGGTGGAATTCTTGGTGTAGAGGTGAAATTCGTAGATATCAAGAGGAACACCGGTGGCGAAGGCGGCCAACTGGACGAATACTGACGCTGAGACACGAAAGCGTGGGGAGCAAACAGG
## 1213                                                         ACATAGGGTGCAAGCGTTGTCCGGAATTATTGGGCGTAAAGAGCTCGTAGGTGGTTCGTCACGTCGGATGTGAAACTCTGGGGCTTAACCCCAGACCTGCATTCGATACGGGCGAGCTTGAGTATGGTAGGGGAGTCTGGAATTCCTGGTGTAGCGGTGGAATGCGCAGATATCAGGAGGAACACCAATGGCGAAGGCAGGACTCTGGGCCATTACTGACACTGAGGAGCGAAAGTCTGGGGAGCGAACAGG
## 1214                                                        ACGAAGGGGGCTAGCGTTGTTCGGAATTACTGGGCGTAAAGCGCGCGTAGGCGGTTTTACAAGCCAGAGGTGAAATGCCCGAGCTTAACTTGGGAATTGCCTTTGGAACTGTATGGCTTTGAGGACGAGAGAGGTGAGTGGAATTCCCAGTGTAGAGGTGAAATTCGTAGATATTGGGAAGAACACCGGTGGCGAAGGCGGCTCACTGGCTCGTTTCTGACGCTGTAGCGCGAAAGCGTGGGGATCAAACAGG
## 1217                                                         ACAGAGACCTCAAGCGTTATCCGGATTTATTGGGCGTAAAGCGTCCGCAGGTGGTTTGGCAAGTCAGGGGTTAAAACTTAACGCTTAACGTTAAGGCTGCTCTTGATACTACCAAACTAGAGATCGGAAGAGGTAAGCGGAATTCTCGGTGTAGTCGTAATAAGCGTTGATATCGAGAAGAACACCAAATGCGAAGGCAGCTTACTGGTACGTTTCTGACACTCATGGACGAAAGCGTGGGGAGCAAACAGG
## 1218                                                         ACAGAGGATGCAAGCGTTATCCGGAATCACTGGGCATAAAGCGTCTGTAGGTGGTTTGGTAAGTCTGCTGTTAAAGACTGGGGCTCAACCCCAGAAAAGCAGTGGAAACTGCTAGACTTGAGTGTGGTAGAGGTAGAGGGAATTCCTAGTGTAGCGGTGAAATGCGTAGATATTAGGAAGAACACCAATGGCGAAGGCAATCCCCTGGACCTGTACTGACGCTCATGCACGAAAGCGTGGGGAGCAAACAGG
## 1219                                                         ACGTAGGGTGTTAGCGTTGTTCGGAATCATTGGGCGTAAAGCGCGTGTAGGTGGCTGTGTAAGTCGAATGTGAAATCCCTGGGCTCAACCGAGGAAGTGCATTCGAAACTACATAGCTCGAGGACGGTAGAGGAAGGTGGAATTCCAAAAGTAGAGGTGAAATTCGTAGATATTTGGAGGAATACCGGCGGCGAAGGCGGCCTTCTGGGCCGTTCCTGACACTGAGACGCGAAAGCGTGGGTAGCAAACAGG
## 1220                                                         ACGAAGGGTGCAAGCGTTACTCGGAATTACTGGGCGTAAAGCGTGCGTAGGTGGTTCGTTAAGTCTGTCGTGAAATCCCCGGGCTCAACCTGGGAATGGCGATGGATACTGGCGGGCTAGAGTGCGGTAGAGGAGAGTGGAATTCCCGGTGTAGCAGTGAAATGCGTAGAGATCGGGAGGAACATCAGTTGCGAAGGCGGCTCTCTGGACCAGCACTGACACTGAGGCACGAAAGCGTGGGGAGCAAACAGG
## 1221                                                         ACAGAGGGTGCAAACGTTGTTCGGAATTACTGGGCGTAAAGCGCGTGTAGGCGGCTTGGCAAGTCGGGTGTGAAAGCCCTGGGCTCAACCCAGGAAGTGCACTCGAAACTGCTTCGCTAGAGTACCGGAGAGGATAGCGGAATTCTCGGTGTAGAGGTGAAATTCGTAGATATCGAGAGGAACACCGGTGGCGAAGGCGGCTATCTGGACGGTTACTGACGCTGAGACGCGAAAGCGTGGGGAGCAAACAGG
## 1222                                                         ACAGAGGATGCAAGCGTTATCCGGAATGATTGGGCGTAAAGCGTCTGTAGGTGGCTTAAAAAGTCTCCTGTCAAAGATCAGGGCTTAACCCTGGGCCGGCAGGAGAAACTCTTAGGCTAGAGTTTGGTAGGGGCAGAGGGAATTCCCGGTGGAGCGGTGAAATGCGTAGAGATCGGGAGGAACACCAAAGGCGAAAGCACTCTGCTGGGCCATAACTGACACTGAGAGACGAAAGCGAGGGGAGCAAAAGGG
## 1224                                                         ACAGAGACCTCAAGCGTTATCCGGATTAATTGGGCGTAAAGCGTAGGTAGGCGGTCTTGTTAGTCAGATGTCAAAGCCCGAGGCTCAACCTCGGAAAGGCATTTGAAACGGCAAGACTCGAGAGTGTGAGAGATCAGTGGAATTCATGGTGTAGTAGTGAAATGCGTTGATATCATGAGGAACATCCAAGGCGAAGGCAACTGATTGGCGCACTTCTGACGCTCAGCTACGAAAGCGTGGGTAGCGAATGGG
## 1225                                                          ACAGAGGTCCCGAGCGTTGTTCGGAATTACTGGGCGTAAAGGGCGCGTAGGCGGTAATACAAGTTGCGGGTAAAATTTCACGGCTCAACCGTGTCAGGCTCGCAAGACTGTGTTGCTAGAGGGCTGTAAGGGGGGTTGGAATTCTCGGTGTAGCAGTGAAATGCGTAGATATCGAGAGGAACATCAGTGGCGAAAGCGAACCCCTGGGCAGATCCAGACGCTGAGGCGCGAAAGCCAAGGTAGCAAACGGG
## 1226                                                         ACGGAGGATGCAAGCGTTATCCGGATTCATTGGGTTTAAAGGGTGCGCAGGCGGAATAATAAGTCAGCGGTGAAAGCCTACAGCTTAACTGTAGAACTGCCATTGATACTGTTATTCTTGAGTGTTGTTGAGGTAGGCGGAATGTGTCATGTAGCGGTGAAATGCTTAGATATGACACAGAACACCGATCGCGAAGGCAGCTTACCAAGCAACAACTGACGCTCATGCACGAAAGCGTGGGGATCAAACAGG
## 1228                                                         ACATAGGGTGCAAGCGTTGTCCGGAATTATTGGGCGTAAAGAGCTCGTAGGTTGTTTGTTACGTCGGATGTGAAAACCTGAGGCTCAACCTCAGGCCTGCATTCGATACGGGCAAACTAGAGTTTGGTAGGGGAGACTGGAATTCCTGGTGTAGCGGTGGAATGCGCAGATATCAGGAGGAACACCAATGGCGAAGGCAGGACTCTGGGCCATTACTGACACTGAGGAGCGAAAGCGTGGGGAGCGAACAGG
## 1230                                                         ACGGAGGGTGCAAGCGTTATTCGGAATTACTGGGCGTAAAGCGCGCGTAGGCTGCTTTGTAAGTCAGGGGTGAAATCCCACGGCTCAACCGTGGAACTGCCTTTGAAACTGCGAAGCTTGAATCCTGGAGAGGGTGGCGGAATTCCTGGTGTAGGAGTGAAATCCGTAGATATCAGGAGGAACACCGGTGGCGAAGGCGGCCACCTGGACAGGTATTGACGCTGAGGTGCGAAAGTGTGGGGAGCAAACAGG
## 1231                                                         ACGGAGGGGGCTAGCGTTGTTCGGAATTACTGGGCGTAAAGCGCACGTAGGCGGACCGGAAAGTCAGAGGTGAAATCCCAGGGCTCAACCTTGGAACTGCCTTTGAAACTCCTGGTCTTGAGGTCGAGAGAGGTGAGTGGAATTCCGAGTGTAGAGGTGAAATTCGTAGATATTCGGAGGAACACCAGTGGCGAAGGCGGCTCACTGGCTCGATACTGACGCTGAGGTGCGAAAGCGTGGGGAGCAAACAGG
## 1232                                                         ACGGAGGGTGCGAGTGTTATTCGGAATCACTGGGCGTAAAGCGAACGTAGGCGGATAGGCAAGTCAGATGTGAAATCCCGGGGCTCAACCTCGGAACTGCATTTGATACTGCTTGTCTTGAGTATGGTAGAGGTAGGTAGAATTCCCGGTGTAGAGGTGAAATTCGTAGATATCGGGAGGAATACCAGAGGCGAAGGCGACCTACTGGGCCATTACTGACGCTGAGGTTCGAAAGCGTGGGTAGCAAACAGG
## 1234                                                         ACGGAGGGTGCAAGCGTTGTTCGGAATCATTGGGCGTAAAGCGCGCGCAGGCGGATCAGCAAGTCATGTGTGAAATCTCGGGGCTCAACTCCGAAATTGCGCCTGAAACTGCTAGTCTAGAATATCATAGGGGATAGGGGTATTTCACGTGTAGGGGTAAAATCCGTAGAGATGTGAAGGAACACCGGAGGCGAAGGCGCCTATCTGGATGATTATTGACGCTGAGGCGCGAAAGCGTGGGGAGCAAACAGG
## 1235                                                         ACGTAGGATGCGAGCGTTGTCCGGAATTATTGGGCGTAAAGCGTACGTAGGCGGTTTGTTAAGTTCGATCTTAAAGACTGAGGCTCAACCTTAGGAGTGGATTGAAGACTGGCAGGCTAGAGGACATCAGAGGGAAGTGGAATTCCGAGTGTAGCGGTGAAATGCGTAGATATTCGGAGGAACACCAGAGGCGAAAGCGGCTTCCTGGGGTGGACCTGACGCTGAGGTACGAAAGCTTGGGGAGCAAACAGG
## 1236                                                         ACGGAGGGTGCAAGCGTTATCCGGATTTATTGGGTTTAAAGGGTGCGTAGGCGGGTTTGTAAGTCAGTGGTGAAAGCCTACAGCTTAACTGTAGAATTGCCATTGATACTGCAGACCTTGAGTATAGTTGAAGTAGGCGGAATGTGTCATGTAGCGGTGAAATGCTTAGATATGCCACAGAACACCGATTGCGAAGGCAGCTTGCTAAGCTATAACTGACGCTGAGGCACGAAAGCGTGGGGAGCAAACAGG
## 1239                                                         ACGAGTGGTGCAAACGTTATTCGGAATCACTGGGCTTAAAGGGTGCGTAGGCGGCTATGAAGGTCTGATGTGAAATACCACGGCTCAACCGTGGAATGGCATTGGAAACCATATGGCTGGAGTGAGACAGAGGTGAGCGGAACAGATGGTGGAGCGGTGAAATGCGTTGATATCATCTGGAACACCGGTGGCGAAAGCGGCTCACTGGGTCTTTACTGACGCTGAGGCACGAAAGCTAGGGGAGCAAACGGG
## 1240                                                         ACGTACTGTGCGAACGTTATTCGGAATCACTGGGCTTAAAGGGTGCGTAGGCGGCGATTCAAGTCAGATGTGAAATGCCTGAGCTCAACTGAGGCATTGCGTTTGAAACTGAATTGCTAGAGTATTTCAGGGGTGTGCGGAACTGCCAGTGGAACGGTGAAATGTGTTGATATTGGCAGGAACACCGGAGGCGAAAGCGGCACACTGGGGAATAACTGACGCTGAGGCACGAAAGCCAAGGGAGCGAACGGG
## 1241                                                        ACAGAGACCCCAAGCGTTATCCGGAATCATTGGGCGTAAAGGGTGAGTAGGTGGTATTATTAGTCGTGTGTTAAAGTCCAAGGGCTCAACCTTTGGAATGCATACGAAACGGTAAAACTAGAATATGTAAGAGGTGAGTGGAACTCTATGTGTAGGGGTAAAATCCGTTGATATATAGGGGAACACCAAAAGCGAAGGCAGCTCACTGGTACATTATTGACACTGAATCACGAAAGCGTGGGTAGCGATACGG
## 1242                                                        ACAGAGGGTGCAAGCGTTAATCGGAATCACTTGGGCGTAAAGGGTGCGTAGGTGGAATGATAAGTTTCGTGTGAAATCCCTGGGCTTAACCTGGGAACTGCACGGGAGACTGTTGTTCTAGAGTACAGGAGAGGTGAGTGGAATTTCCGGTGTAGCAGTGAAATGCGTAGAGATCGGAAGGAACACCAGTGGCGAAGGCGACTCACTGGCCTGATACTGACACTCAAGCACGAAAGCGTGGGGAGCAAACAGG
## 1243                                                         ACGTAGGCACCAAGCGTTGTCCGGATTTATTGGGCGTAAAGAGCTCGTAGGTGGTTCGTCACGTCGGATGTGAAACTCTGGGGCTTAACCCCAGACCTGCATTCGATACGGGCGAGCTTGAGTATGGTAGGGGAGTCTGGAATTCCTGGTGTAGCGGTGGAATGCGCAGATATCAGGAGGAACACCAATGGCGAAGGCAGGACTCTGGGCCATTACTGACACTGAGGAGCGAAAGCGTGGGGAGCGAACAGG
## 1245                                                         ACGAAGGGGGCTAGCGTTGTTCGGAATTACTGGGCGTAAAGCGCACGTAGGCGGACTTTTAAGTCAGGTGTGAAATCCCGGGGCTCAACCTCGGAACTGCATTTGAAACTGGAAGTCTTGAGACCAGGAGAGGTTAGCGGAATACCGAGTGTAGAGGTGAAATTCGTAGATATTCGGTGGAACACCAGTGGCGAAGGCGGCTAACTGGACTGGTACTGACGCTGAGGTGCGAAAGTGTGGGGAGCAAACAGG
## 1246                                                         ACGAAGGGTGCGAGCGTTAATCGGAATTACTGGGCGTAAAGCGCGCGTAGGTGGTTAAGTAAGCGATCTGTGAAAGCCCCGGGCTTAACCTGGGAACTGCAGGTCGAACTGCTTGGCTAGAGTATGGTAGAGGGGTGTGGAATTTCCTGTGTAGCGGTGAAATGCGTAGATATAGGAAGGAACATCAGTGGCGAAGGCGACACCCTGGACCGATACTGACACTGAGGCGCGAAAGCGTGGGTAGCGAACAGG
## 1247                                                         ACGAAGGGTGCAAGCGTTGTTCGGAATTATTGGGCGTAAAGGGTTCGTAGGCTGGAATTTAAGTCAAGTGTGAAATCCTCGGGCTTAACCTGAGACGTGCATTTGAAACTGAGTTTCTTGAGTACCAGAGAGGGTAGTGGAATTGCTGGTGTAGGAGTGACATCCGTAGAGATCAGCAGGAACACCGGAGGCGAAGGCGACTGCCTGGCTGGTAACTGACGCTGAGGAACGAAAGCGTGGGGAGCAAACAGG
## 1248                                                         ACGTAGGGTGCAAGCGTTATCCGGAATTACTGGGTTTAAAGGGTCTGTAGGCGGGTTGATAAGTCAGTGGTGAAATATCTGGGCTTAACTTGGAGGGTGCCATTGATACTATTAATCTTGAGAGCGGATGAAGTGGGCGGAATGTGCCGTGTAGCGGTGAAATGCTTAGATATGGCACAGAACACCGATTGCGAAGGCAGCTCACTAAACCGCGTCTGACGCTGAGGGACGAAAGCGTGGGGAGCAAACAGG
## 1249                                                         ACGGAGGGTGCAAGCGTTGTCCGGAATCATTGGGCGTAAAGAGTTCGTAGGCGGTTTATTAAGTCTGGTGTTAAAGCCCGAAGCTCAACTTCGGTTCGGCACTGGATACTGGTAGACTAGAATGTGGTAGAGGTAAAGGGAATTCCTGGTGTAGCGGTGAAATGCGTAGATATCAGGAGGAACATCGGTGGCGTAAGCGCTTTACTGGGCCATAATTGACGCTGAGGAACGAAAGCCGGGGTAGCGAATGGG
## 1250                                                         ACGAAGGGTGCTAGCGTTGTTCGGAATCACTGGGCGTAAAGCGCGCGTAGGCGGCGATACAAGTCAGAGGTGAAAGCCTGGAGCTCAACTCCAGAACTGCCTTTGAAACTGTATTGCTAGAGTGTCGGAGGGGATAGCGGAATTGCTAATGTAGAGGTGAAATTCGTAGATATTAGCAGGAACACCGGTGGCGAAGGCGGCTATCTGGACGACAACTGACGCTGAGGCGCGAAAGCGTGGGGATCAAACAGG
## 1251                                                         ACGAACCGTGCAAACGTTATTCGGAATCACTGGGCTTAAAGGGCGCGTAGGCGGGTAATCAAGTCAATGGTGAAATCCTCCAGCTTAACTGGAGAAGTGCCTTTGATACTGGTTGTCTAGAGGGAGGTAGGGGCATGTGGAACTTCAGGTGGAGCGGTGAAATGCGTAGATATCTGAAGGAACGCCAGTGGCGAAAGCGATGTGCTGGACCTCTTCTGACGCTGAGGCGCGAAAGCTAGGGGATCAAACGGG
## 1254                                                         ACGTAGGGTGCAAGCGTTAATCGGAATTACTGGGCGTAAAGCGTGCGCAGGCGGTTGTGTAAGACAGGCGTGAAATCCCCGGGCTCAACCTGGGAATGGCGCTTGTGACTGCACAGCTCGAGTGCGGCAGAGGGGGATGGAATTCCGCGTGTAGCAGTGAAATGCGTAGATATGCGGAGGAACACCAATGGCGAAGGCAATCCCCTGGGCCTGCACTGACGCTCATGCACGAAAGCGTGGGGAGCAAACAGG
## 1255                                                         ACGGAGGGTGCAAGCGTTATCCGGATTTACTGGGTTTAAAGGGTGCGTAGGCGGGCCTTTAAGTCAGTGGTGAAATCTCCCGGCTTAACCGGGAAACTGCCATTGATACTATTGGTCTTGAATTCAGTTGAGGTGGGCGGAATGTGTCGTGTAGCGGTGAAATGCTTAGATATGACACAGAACACCAATTGCGAAGGCAGCTCGCTAAACTGACATTGACGCTGAGGCACGAAAGCGTGGGGATCAAACAGG
## 1256                                                         ACAGAGGGTGCAAGCGTTAATCGGAATTACTGGGCGTAAAGAGTGCGTAGGCGGATAATTAAGTCATATGTGAAAGCCCCGGGCTTAACCTGGGAATTGCATCTGATACTGGTTATCTAGAGTATGGTAGAGGATAGTGGAATTTCTGGTGTAGCGGTGAAATGCGTAGATATCAGAAGGAACACCAGTGGCGAAGGCGACTGTCTGGGCCAATACTGACGCTGAGGTACGAAAGCGTGGGTAGCGAACAGG
## 1258                                                         ACGTATGTCGCAAGCGTTATCCGGAATTATTGGGCGTAAAGGGCATCTAGGCGGTTAATCAAGTCGAAGGTGAAAACTTGTGGCTCAACCATAAGCTTGCCTACGAAACTGAATAACTAGAGTACTGGAGAGGTGGACGGAACTACACGAGTAGAGGTGAAATTCGTAGATATGTGTAGGAATGCCGATGATGAAGATAGTTCACTGGACAGAAACTGACGCTGAAGTGCGAAAGCTAGGGGAGCAAACAGG
## 1259                                                         ACGTAGGTGGCAAGCGTTGTCCGGAATTACTGGGCGTAAAGGGCGCGTAGGCGGCACCTTAAGTCAGATGTGAAAACCCAGGGCTTAACCTTGGGACTGCATTTGAAACTGAGGAGCTAGAGTGCCGGAGAGGAAAGCGGAATTCCTAGTGTAGCGGTGAAATGCGTAGATATTAGGAGGAACACCAGTGGCGAAGGCGGCTTTCTGGACGGTAACTGACGCTGAGGCGCGAAAGCGTGGGGAGCAAACAGG
## 1261                                                       ACGTAGGGTGCAAGCATTATCCGGAGTGACTGGGCGTAAAGAGTTGCGTAGGTGGACAAGTAAGCGAATAGTGAAATCTGGGGGCTCAACCTCACAGACTATTATTCGAACTGCTTGTCTCGAGAATGGTAGAGGTAACTGGAATTTCTAGTGTAGGAGTGAAATCCGTAGATATTAGAAGGAACACCAATGGCGTAGGCAGGTTACTGGACCATTTCTGACACTGAGGCACGAAAGCGTGGGGAGCGAACCGG
## 1265                                                         ACGGAGGGGGCTAGCGTTGTTCGGAATGACTGGGCGTAAAGGGCGCGTAGGCGGTGTCTTAAGTGAGGCGTGAAAGCCCCGGGCTTAACCCGGGAGGTGCGTTTCATACTGAGATGCTAGAGTGCGAGAGAGGAAAGTGGAATTCCTAGTGTAGAGGTGAAATTCGTAGATATTAGGAAGAACACCAGAGGCGAAGGCGGCTTTCTGGCTCGTAACTGACGCTGAGGCGCGAAAGCGTGGGGAGCAAACAGG
## 1266                                                         ACGGAGGGTGCAAGCGTTATCCGGATTCACTGGGTTTAAAGGGTGCGTAGGTGGCTTTGTAAGTCAGTGGTGAAAGCCCGGAGCTCAACTCCGGAACTGCCATTGATACTGCTTAGCTTGAATCAACTTGAGGTGGATGGAATATTACATGTAGCGGTGAAATGCTTAGATATGTAATAGAACACCGATTGCGAAGGCAGTTCACTAAGGTTGTATTGACACTGAGGCACGAAAGCGTGGGGATCAAACAGG
## 1268                                                         ACGGAGGGTGCAAGCGTTAATCGGAATTACTGGGCGTAAAGCGCACGCAGGCGGTTTGTTAAGTCAGATGTGAAATCCCCGCGCTTAACGTGGGAACTGCATTTGAAACTGGCAAGCTAGAGTCTTGTAGAGGGGGGTAGAATTCCAGGTGTAGCGGTGAAATGCGTAGAGATCTGGAGGAATACCGGTGGCGAAGGCGGCCCCCTGGACAAAGACTGACGCTCAGGTGCGAAAGCGTGGGGAGCAAACAGG
## 1270                                                        ACGGAGGGTGCAAGCGTTATCCGGAATCACTGGGTTTAAAGGGTGCGTAGGCGGTTTGATAAGTCAGTTGTGAAATTCCCTCGCTCAACGAGTGGGACTGCGATTGATACTGTTGAACTTGAATCAGGTTGAGGTAGGCGGAATGTGGCATGTAGCGGTGAAATGCATAGATATGCCATAGAACACCAATTGCGAAGGCAGCTTGCTGGGCTTTGATTGACGCTGAGGCACGAAAGCGTGGGGAGCAAACAGG
## 1272                                                         ACGGAGGGTGCAAGCGTTATCCGGATTTATTGGGTTTAAAGGGTCCGTAGGCGGACCTATAAGTCAGTGGTGAAATCTTCCGGCTCAACCGGGAAACTGCCATTGATACTGTAGGTCTTGAGTATGTATGAAGTTGGCGGAATGTGTGGTGTAGCGGTGAAATGCTTAGATATCACACAGAACACCGATTGCGAAGGCAGCTGACTAATACATAACTGACGCTGAGGGACGAAAGTGTGGGGATCAAACAGG
## 1276                                                         ACGAAGGGGGCTAGCGTTGTTCGGAATTACTGGGCGTAAAGCGCACGTAGGCGGATATTTAAGTCAGGGGTGAAATCCCGGGGCTCAACCCCGGAACTGCCTTTGATACTGGGTATCTAGAGTATGGAAGAGGTGAGTGGAATTCCGAGTGTAGAGGTGAAATTCGTAGATATTCGGAGGAACACCAGTGGCGAAGGCGGCTCACTGGTCCATTACTGACGCTGAGGTGCGAAAGCGTGGGGAGCAAACAGG
## 1277                                                         ACGGAGGGGGCTAGCGTTGTTCGGAATTACTGGGCGTAAAGAGCGCGTAGGCGGTTTGGTAAGTTGGAAGTGAAATCCCGGGGCTTAACCTCGGAATTGCTTTCAAAACTGCCAATCTAGAGTGTAGTAGGGGATGATGGAATTCCTAGTGTAGAGGTGAAATTCTTAGATATTAGGAGGAACACCGGTGGCGAAGGCGGTCATCTGGGCTACAACTGACGCTGATGCGCGAAAGCGTGGGGAGCAAACAGG
## 1278                                                         ACATAGGGTGCAAGCGTTGTCCGGAATTATTGGGCGTAAAGAGCTCGTAGGTTGTTTGTTACGTCGGATGTGAAAACCTGAGGCTCAACCTCAGGCCTGCATTCGATACGGGCAAACTAGAGTTTGGTAGGGGAGACTGGAATTCCTGGTGTAGCGGTGGAATGCGCAGATATCAGGAGGAACACCAATGGCGAAGGCAGGTCTCTGGGCCAATACTGACACTGAGGAGCGAAAGCGTGGGGAGCGAACAGG
## 1279                                                         ACGTAGGGTGCAAGCGTTAATCGGAATTACTGGGCGTAAAGCGTGCGCAGGCGGTTATGTAAGACAGAGGTGAAATCCCCGGGCTCAACCTGGGAACTGCCTTTGTGACTGCATAGCTAGAGTACGGTAGAGGGGGATGGAATTCCGCGTGTAGCAGTGAAATGCGTAGATATGCGGAGGAACACCGATGGCGAAGGCAGTCCCCTGGGATAAGACTGACGCTCATGCACGAAAGCGTGGGGAGCAAACAGG
## 1280                                                         ACGAAGGGTGCGAGCGTTGTTCGGAATGACTGGGCGTAAAGGGTTCGTAGGCGGGAATACAAGTCAAGTGTGAAATCCCCAGGCTCAACCTGGGACGTGCACTTGAAACTGTATTTCTTGAGTCGAGGAGAGGGTAATGGAATTGCTGGTGTAGGAGTGACATCCGTAGAGATCAGCAGGAACACCGGAGGCGAAGGCGATTACCTGGCCGAAGACTGACGCTGAGGAACGAAAGCGTGGGGAGCAAACAGG
## 1281                                                         ACGGAGGGTGCAAGCGTTAATCGGAATAACTGGGCGTAAAGCGCACGCAGGCGGTTAGATAAGTCAGATGTGAAATCCCCGGGCTCAACCTGGGAACTGCATTTGAAACTGTCTGACTAGAGTCTTGTAGAGGGGGGTAGAATTCCAGGTGTAGCGGTGAAATGCGTAGAGATCTGGAGGAATACCGGTGGCGAAGGCGGCCCCCTGGACAAAGACTGACGCTCATGCACGAAAGCGTGGGGAGCAAACAGG
## 1282                                                         ACGGAGGGTGCTAGCGTTGTTCGGAATCACTGGGCGTAAAGGGCGTGTAGGCGGTCTGCTAAGTCTGATGTGAAATCCCCTCGCTCAACGAGGGAACTGCGTCGGATACTGGTAGACTTGAGTACCGGAGAGGAAGGTGGAATTCCCGGTGTAGCGGTGAAATGCGTAGATATCGGGAGGAACATCGGTGGCGAAGGCGGCCTTCTGGACGGATACTGACGCTGAGACGCGAAAGCGTGGGGAGCAAACAGG
## 1283                                                         ACGGAGGGTGCAAGCGTTAATCGGAATAACTGGGCGTAAAGCGCACGCAGGCGGTTTGCTAAGCTAGATGTGAAAGCCCCGGGCTCAACCTGGGAACTGCATTTAGAACTGGCGAACTAGAGTCTTGTAGAGGGGGGTAGAATTCCAGGTGTAGCGGTGAAATGCGTAGAGATCTGGAGGAATACCGGTGGCGAAGGCGGCCCCCTGGACAGAGACTGACGCTCAGGTGCGAAAGCGTGGGGAGCAAACAGG
## 1284                                                         ACGTAGGTGGCAAGCGTTGTCCGGAATTATTGGGCGTAAAGCGCGCGCAGGCGGTCCTTTAAGTCTGATGTGAAAGCCCACGGCTCAACCGTGGAGGGTCATTGGAAACTGGGGGACTTGAGTGCAGAAGAGGAAAGTGGAATTCCACGTGTAGCGGTGAAATGCGTAGAGATGTGGAGGAACACCAGTGGCGAAGGCGACTTTCTGGTCTGTAACTGACGCTGAGGCGCGAAAGCGTGGGGAGCAAACAGG
## 1285                                                         ACGAAGGGGGCTAGCGTTACTCGGAATTACTGGGCGTAAAGGGCGCGTAGGCGGCTCTGTAAGTCAGGCGTGAAATTCCTGGGCTCAACCTGGGGGCTGCGCTTGAGACTGTGGGGCTAGAGGATGGAAGAGGGTCGTGGAATTCCCAGTGTAGAGGTGAAATTCGTAGATATTGGGAAGAACACCGGTGGCGAAGGCGGCGACCTGGTCCATTACTGACGCTGAGGCGCGACAGCGTGGGGAGCAAACAGG
## 1286                                                       ACAGAGGGTGCAAGCGTTATCCGGATTTATTGGGCGTAAAGCGTTTCGTAGGTGGTTTTGTAAGTTAAACTTCAAAGACTCGGGCTCAACTCGGGGAAGGGGTTTAATACTGCAGGACTTAGAGAGTTTTAGGGGCAATCGGAACTGTTGGTGTAGGGGTGAAATCCGTTGATATCAACAGGAACGTCAAGGGCGTAGGCAGATTGCTGGGAAATTTCTGACACTGAGGAACGAAAGCTAGGGGAGCGAAAGGG
## 1289                                                         ACGAAGGACCCGAGCGTTATCCGGATTTACTGGGTTTAAAGGGTGCGTAGGTGGACTTTTAAGTCAGTGGTGAAAGCCTCCCGCTCAACGGGAGAACTGCCATTGATACTGATAGTCTTGAGTATAGTTGAGGTAGGCGGAATATGTCATGTAGTGGTGAAATACTTAGATATGACATAGAACACCAATTGCGAAGGCAGCTTGCTAAGCTATAACTGACACTGAGGCACGAAAGCGTGGGGAGCAAACAGG
## 1291                                                         ACAGAGGGTGCAAGCGTTGCTCGGATTTACTGGGCGTAAAGCGTACGCAGACGGCATCAAAGGTCGGGTGTGAAAGCCCGGGGCTCAACCCCGGAATTGCATCCGAAACCGTGAAGCTAGAGTATGGGAGAGGGAAGCGGAATTCCTGGTGTAGAGGTGAAATTCGTAGATATCAGGAGGAACATCTGTGGCGAAGGCGGCTTCCTGGCCCATTACTGACGTTCAGGTACGAAAGCGTGGGTAGCAAACAGG
## 1292                                                         ACGGGAGTGGCAAGCGTTATCCGGAATTATTGGGCGTAAAGCGTCCGCAGGCGGCCGTACAAGTCTGTCGTTAAAGCGTGGAGCTTAACTCCATTTAAGCGATGGAAACTGTAAGGCTAGAGTGTGGTAGGGGCAGAGGGAATTCCCGGTGTAGCGGTGAAATGCGTAGATATCGGGAAGAACACCAGTGGCGAAGGCGCTCTGCTGGGCCATAACTGACGCTCATGGACGAAAGCCAGGGGAGCGAAAGGG
## 1293                                                         ACGTAGGGGGCGAGCGTTGTCCGGAATTACTGGGCGTAAAGCGCACGCAGGCGGGTTGTTAAGTCGGCTGTGAAAAGCACGGGCTCAACCTGTGTCTTGCGGTCGATACTGGCAGCCTCGAGTACGAGAGAGGGAAGTGGAATTCCCGGTGTAGCGGTGAAATGCGTAGATATCGGGAGGAACACCAGTGGCGAAGGCGGCTTCCTGGCTCGTGACTGACGCTCATGTGCGAAAGCTGGGGGAGCGAACGGG
## 1294                                                         ACGAGGGGTCCTAGCGTTGTTCGGAATCATTGGGCGTAAAGCGCTCGTAGGTGGCTTAGTAAGTCGGGTGTGAAAGCCCCGGGCTCAACCCGGGAAGTGCACTCGAAACTGCATAGCTTGAGTACGGTAGAGGTGAGTGGAATTCCAGGTGTAGTGGTGAAATACGTAGATATCTGGAGGAACATCGGTGGCGAAGGCGGCTCACTGGACCAGTACTGACACTGAGGAGCGAAAGCATGGGGATCAAACAGG
## 1295                                                         ACGAGAGGTGCAAGCGTTATTCGGAATTATTGGGCGTAAAGGGCGCGTAGGCGGCGCTATAAGTTTTTCGTTTAATTCTCCAGCCTAACTGGAGCCATGCGAAAAAGACTGTAGTGCTAGAGTATAATAGAGAGAAGTGGAATTTTCGGAGTAGCGGTTAAATGCGTAGATCTCGAAAGGAACGCCAATGGCGAAGGCAGCTTCTTGGGTTATTACTGACGCTGAGGCGCGAAGGCATGGGGAGCAAACAGG
## 1297                                                         ACGAGGGATCCTAGCGTTGTTCGGAATCATTGGGCGTAAAGCGGATGTAGGTGGCTTTGTAAGTCAGGTGTGAAAGCCCCGGGCTCAACCCGGGAAGTGCATTTGATACTGCGAAGCTTGAGTGCTGGAGAGGTTACTAGAATACCTGGTGTAGTGGTGAAATACGTAGATATCAGGTGGAATACCGGAGGCGAAGGCGGGTAACCAGCCAGACACTGACACTCAGATCCGAAAGTGTGGGGATCAAACAGG
## 1298                                                         ACAGAGGGTGCAAACGTTGCTCGGAATTACTGGGCGTAAAGGGCGTGTAGGCGGGAAGGTAAGTCGGGCGTGAAATCCCCAAGCTCAACTTGGGAACTGCACTCGAAACTACCTTTCTTGAGTGCCGGAGAGGAAAGCGGAATTCCTGGTGTAGAGGTGAAATTCGTAGATATCAGGAGGAACACCAGTGGCGAAGGCGGCTTTCTGGACGGTGACTGACGCTGAGACGCGAAAGCGTGGGGAGCAAACAGG
## 1299                                                         ACGGAGGGTGCAAGCGTTACTCGGAATTACTGGGCGTAAAGCGCGTGTAGGTGGTTTTGTAAGTCTGGTGTGAAATCCCGGGGCTCAACCCCGGAAGTGCATTGGATACTGCAAGACTAGAGTGCAGGAGAGGATGGTGGAATTCCAGGTGTAGAGGTGAAATTCGTAGATATCTGGAGGAACATCGGAGGCGAAGGCGACCATCTGGACTGCAACTGACACTGAGACGCGAAAGCGTGGGGAGCAAACAGG
## 1300                                                         ACATAGGGTGCAAGCGTTGTCCGGAATTATTGGGCGTAAAGAGCTCGTAGGTGGTTCGATACGTCGGATGTGAAAATCAGGGGCTCAACCCCTGACCTGCATTCGATACGGTCGAGCTAGAGTTTGGTAGGGGAGACTGGAATTCCTGGTGTAGCGGTGGAATGCGCAGATATCAGGAGGAACACCGATGGCGAAGGCAGTCCCCTGGGATAAGACTGACGCTCATGCACGAAAGCGTGGGGAGCAAACAGG
## 1301                                                         ACGAGGGGTCCTAGCGTTGTTCGGAATCACTGGGCGTAAAGCGCATGTAGGCGGCTTTGTAAGTCAGTTGTGAAAGCCCTGGGCTTAACCCAGGAAGTGCATCTGATACTGCAAAGCTTGAGTATGGGAGAGGGTAGTAGAATTCCTGGTGTAGTGGTGAAATACGTAGATATCAGGAGGAATACCGGTGGCGAAGGCGGCTACCTGGCCCAATACTGACGCTGAGATGCGAAAGCGTGGGGATCAAACAGG
## 1302                                                         ACGTAGGGTGCGAGCGTTAATCGGAATTACTGGGCGTAAAGGGTGCGTAGGCGGATATATAAGTTAGATGTGAAAAGCCTGGGCTTAACCTAGGTCGTGCATTTAAGACTGTATGTCTAGAGTTTGTAAGAGGGGGGTAGAATTCCAAGTGTAGCAGTGAAATGCGTAGAGATTTGGAGGAATACCGATGGCGAAGGCAGCCCCCTGGTATAGAACTGACGCTGAGGCACGAAAGCGTGGGTAGCAAACAGG
## 1304                                                         ACAGAGGGGGCAAGCGTTATTCGGAATTATTGGGCGTAAAGGGCGCGTAGGCGGCCTTGTAAGTCTTGGGTGAAATCCCTCAGCTCAACTGAGGAACTGCCTCGGATACTGCAGGGCTTGAGGCCGGGAGAGGGTAGTGGAATTCCCAGTGTAGCGGTGAAATGCGTAGATATTGGGAGGAACACCGGTGGCGAAGGCGGCTACCTGGACCGGTTCTGACGCTGATGCGCGAAAGCGTGGGGAGCAAACAGG
## 1306                                                       ACGGGAGGAACAAGCGTTACTCATGGTAACTTGGTGTTTAGGGTGCCGCGGCGGTTTGTGTGGAAAGAAAGGGAGAAAGCACAGCAGGAATGGTGGAAGGTCCTTGGTAATCCGCAAACTAGAGTTTGAATAGGGGATATGGTTATGGGTAGGGAAGCAATGAAATGCCGAGAAACTACTCGGAGCTCCAGCGGCGCAGGCGCGTATCCATCTGAAACTGACGCTAACGCACTAAAGCGTGGGGTTCAAACAGG
## 1308                                                         ACGTAGGTGGCGAGCGTTACTCGGAATTACTAGGCGTAAAGCGCGTGTAGGCGGGAGGTTAAGTCTGCTGTTAAATCTCATGGCTCAACCATGAAATGCCGGCGGATACTGGCCTTCTTGAATTCGGTAGAGGTGACTGGAATTCCGTGTGTAGCGGTGAAATGCGTAGATATACGGAAGAACGCCAAAGGCGAAGGCAGGTCACTGGGCCGCAATTGACGCTGAGACGCGAAAGCTAGGGGAGCAAACAGG
## 1310                                                         ACATAGGCTTCAAGCGTTGTCCGGATTTATTGGGCGTAAAGAGTTCGTAGGCGGTCGAGTAAGTCGGGTGTGAAAATTCTGGGCTCAACCCAGAGACGCCACCCGATACTGCTTAACTTGAGTTCGATAGGGGAGTGGGGAATTCCTAGTGTAGCGGTGAAATGCGCAGATATTAGGAGGAACACCGGTGGCGAAGGCGCCACTCTGGATCGACACTGACGCTGAGGAACGAAAGCATGGGTAGCAAACAGG
## 1311                                                         ACAGAGGGTGCAAGCGTTGCTCGGAATCATTGGGCGTAAAGGGCAAGTAGGTGGTCTCGTTTGTCTGAGGTGAAATCCCTGGGCTTAACCTAGGAACTGCCTCAGAAACGGCGAGACTGGAGTCTCGGAGAGGGTCGCGGAATTCCCGGTGTAGCGGTGAAATGCGTAGAGATCGGGAGGAATACCAGAGGCGAAAGCGGCGACCTGGACGAGTACTGACACTCAACTGCGAAAGCGTGGGGAGCAAACAGG
## 1313                                                        ACAGAGGGTGCAAGCGTTATCCGGATTTATTGGGCGTAAAGCGTTTCGTAGGTTGTTTTGTAAGTTATGTTTTAAAGACCACGGCTTAACCGGGGGAAGGGACATAATACTGCAAGACTTGAGAATTTTAGGGGCAATCGGAACTGTTGGTGTAGGGGTGAAATCCGTTGATATCAACAGGAACGTCAAGGGCGTAGGCAGATTGCTGGGAAATATCTGACACTGAGGAACGACAGCTAAGGGAGCGAAAGGG
## 1314                                                       ACGTGGGGTGCAAGCGTTATCCGGATTTACTGGGCGTAAAGCGTTCTGTAGGCGGTTTTGTAAGTATATGGTTAAAGGCTGCGGCTCAACCGTAGACATGCTGTATAAACTACAATGACTAGATGGTTGTCGGGAGTATCGGAATTCTCGGTGGAGGGGTGAAATCCGTTGATATCGAGAGGAACACCAAAGGCGAAGGCAGATACTTAGGCAATTTATGACGCTGATAGACGACAGCTAGGGGAGCGAAAGGG
## 1315                                                         ACGAAGGATGCTAGCGTTACTCGGAATTACTGGGCGTAAAGAATATGTAGGCTGACTGTTAAGTTGATAGTGAAATCCCCGAGCCTAACTCGGGAACTGCTCTCAAAACTGATAGTCTAGAATATGGTAGAGAGTAAGGGAATTCTTGGTGTAGGGGTAAAATCCGTAGATATCAGGAGGAAGACCGGAAGCGAAAGCGCTTACTTGGGCCATCATTGACGCTGAGATATGAAAGCGTGGGGATCGAACAGG
## 1316                                                         ACGAAGGGTGCAAGCGTTACTCGGAATTACTGGGCGTAAAGCGTGCGTAGGTGGTTGTTTAAGTCTGATGTGAAAGCCCTGGGCTCAACCTGGGAATGGCATTGGATACTGGATAGCTAGAGTGCGGTAGAGGGTAGTGGAATTCCCGGTGTAGCAGTGAAATGCGTAGAGATCGGGAGGAACATCCGTGGCGAAGGCGACTACCTGGACCAGCACTGACACTGAGGCACGAAAGCGTGGGGAGCAAACAGG
## 1317                                                         ACGAAGGACCCAAGCGTTATCCGGATTCATTGGGTTTAAAGGGTGCGTAGGCGGATTGATAAGTCAGTGGTGAAAGCCCGCAGCTTAACTGCGGAACTGCCATTGATACTGTTGATCTTGAATGTGTTTGATGTGGGCGGAATATGACATGTAGCGGTGAAATGCTTAGATATGTCATAGAACACCGATTGCGAAGGCAGCTCACAAAGCCACTATTGACGCTGAGGCACGAAAGCGTGGGGATCAAACAGG
## 1320                                                         ACGTAGGTGGCGAGCGTTATTCGGAATCACTGGGCGTAAAGGGTCCGTAGGCGGTCTGTCAAGTCTGATGTGAAATCTTGGGGCTCAACCCCAAACGTGCATTGGGTACTGACGGACTGGAGTGCATCAGAGGCAAGCGGAATTTGTGGTGTAGCGGTGGAATGCGTAGATATCACAAGGAACGCCAAAGGCGAAAGCAGCTTGCTGGGATGTCACTGACGCTGAGGGACGAAGGTCAGGGGAGCAAAAAGG
## 1321                                                         ACAGAGGGTGCAAGCGTTATTCGGAATTACTGGGCGTAAAGAGCGCGTAGGCTGTTTGATAAGTTTGATGTGAAAGCCCTGGACTCAATTCAGGAAGTGCATCGAATACTGTCAGACTAGAGTATAGGAGAGGAGAACGGAATTCCTGGTGTAGAGGTGAAATTCGTAGATATCAGGAGGAACAACAGTGGCGAAGGCGGTTCTCTGGACTATTACTGACGCTGAGGCGCGAAAGTGTGGGGAGCAAACAGG
## 1323                                                         ACGTAGGGAGCGAGCGTTGTCCGGAATTACTGGGTGTAAAGGGAGTGTAGGCGGGAAAACAAGTCAGATGTGAAAACTATGGGCTCAACCTGTAGACTGCATTTGAAACTATTTTTCTTGAGTGAAGTAGAGGTAAGCGGAATTCCTAGTGTAGCGGTGAAATGCGTAGATATTAGGAGGAACATCAGTGGCGAAGGCGGCTTACTGGGCTTTTACTGACGCTGAGGCTCGAAAGCGTGGGGAGCAAACAGG
## 1326                                                         ACGAACTGTGCGAACGTTATTCGGAATCACTGGGCTTACAGGGTGCGTAGGCGGTTTTCCAAGTCAGGTGTGAAATCCCCCAGCTCAACTGGGGAACTGCGCTTGAAACTGGATCACTTGAGGATGCTAGAGGTAGACGGAACTTCCGGTGGAGCGGTGAAATGTGTTGAGATCGGAAGGAACACCAGAGGCGAAGGCGGTCTACTGGGGCATATCTGACGCTGAGGCACGAAAGCCAGGGGAGCAAACGGG
## 1328                                                        ACGTAGGATCCAAGCGTTATCCGGATTTATTGGGCGTAAAGAGTTGCGTAGGCGGCAGAGTAAGCAGGTAGTGAAAGAGTATGGCTTAACCATATAAACATTACTTGAACTGCTCAGCTAGAGAGCGGAAGAGGTAGCTGGAATTATTAGTGGAGGAGTAATATCCGTAGATATTAGTAAGAACACCAATGGCGAAGGCAGGCTACTGGTCCGTTTCTGACGCTCAGGCACGAAAGCGTAGGTAGCAAACAGG
## 1330                                                         ACGAAGGGGGCAAGCGTTGTTCGGAATTACTGGGCGTAAAGCGCGCGTAGGCGGCGTCGTCAGTCAGAGGTGAAATCCCAGGGCTCAACCTTGGAATTGCCTTTGATACTGCGATGCTTGAGTTCGAGAGAGGGTGGCGGAATACCCAGTGTAGAGGTGAAATTCGTAGATATTGGGTAGAACACCAGTGGCGAAGGCGGCCACCTGGCTCGATACTGACGCTGAGGTGCGAAAGCGTGGGGAGCAAACAGG
## 1331                                                         ACGAAGGTCCCAAGCGTTGTTCGGAATCATTGGGCGTAAAGCGAGTGCGGGTGGCTCTTTAAGTCAGAAGTGAAAGCCCAGAGCTTAACTCTGGAAGTGCTTTTGATACTGGAGAGCTTGAATGTGGCAGAGGGTGCTAGAATACCTAATGTAGTGGTGAAATACGTAGATATTAGGTGGAACACCGGTGGCGAAGGCGGGCACCTGGGCCAACATTGACACTTAGACTCGAAAGCGTGGGGATCAAACAGG
## 1332                                                         ACGGAGGGTGCAAGCGTTATCCGGAATCATTGGGTTTAAAGGGTCCGCAGGCGGATTTATAAGTCAGTGGTGAAAGCCTACAGCTTAACTGTAGAACTGCCATTGATACTGTAAGTCTTGAATTCGGTCGAAGTGGGCGGAATGTGTAGTGTAGCGGTGAAATGCTTAGATATTACACAGAACACCGATAGCGAAGGCAGCTCACTAGGCCTGAATTGACGCTCATGGACGAAAGCGTGGGGAGCAAACAGG
## 1335                                                         ACGTAGGTCTCGAGCGTTATCCGGAATTACTGGGCGTAAAGCGTCTGTAGCCGGGTATATAAGTCTGTCTTTAAATATCCTGATTCAATCGGGAAAAGGGGATAGATACTGTATACCTAGAGGATTTTAGAGGTTAGTGGAATTTCCGGTGGAGCGGTGAAATGTGTTGATATCGGAAGGAATGCCGAAAGCGAAAGCAGCTAACTATAAAATATCTGACGGTCAGAGACGAAAGTTTGGGTAGCAAACGGG
## 1336                                                           ACGAAAGGGGCAAGCGTTATCCGGAATTATTGGGCGTAAAGGGTTTGTAGGCGGTCCAAGAAGTATGCAATGAAAGCACTTTAAAAAGGAGTAACTGTTGCATAGCCCTTAGGACTTGAGGTCAAAAGACGAGAATCGAATTTCGGGTGGAGCGATAAAATGCAAAGATATCCGAAGGAAGACCAAAAGCGAAGGCAGTTCTCGAGGTTGAACCTGACGTTGAAAAACGAAAGCGTGGGGAGCAAACGGG
## 1337                                                         ACGTAGGGTGCAAGCGTTAATCGGAATTACTGGGCGTAAAGCGTGCGCAGGCGGTTTCGTAAGCTTGATGTGAAATCCCCGGGCTCAACCTGGGAACTGCATTGAGGACTGCGAGACTAGAATACGGCAGAGGGGGGTGGAATTCCACGTGTAGCAGTGAAATGCGTAGAGATGTGGAGGAACACCGATGGCGAAGGCAGCCCCCTGGGCCTGTATTGACGCTCATGCACGAAAGCGTGGGGAGCAAACAGG
## 1338                                                         ACGTAGGGGGCAAGCGTTATCCGGATTTACTGGGTGTAAAGGGAGCGTAGGCGGCAAGGCAAGTCTGATGTGAAAACCCGGGGCTCAACCCCGGGACTGCATTGGAAACTGCTTAGCTAGAGTGTCGGAGAGGTAAGTGGAATTCCTAGTGTAGCGGTGAAATGCGTAGATATTAGGAGGAACACCAGTGGCGAAGGCGACTTACTGGACGATAACTGACGCTGAGGCTCGAAAGCGTGGGGAGCAAACAGG
## 1339                                                         ACGAAGGGGGCGAGCGTTGTTCGGAATCACTGGGCGTAAAGGGCACGTAGGCGGCCGTGCAAGTTGGGCGTGAAAGCCCCGGGCTCAACCCGGGATGTGCGCTCAAGACTGCATGGCTCGAGTTCGGAAGAGGGTGGCGGAATTCCCAGTGTAGAGGTGAAATTCGTAGATATTGGGAAGAACACCGGTGGCGAAGGCGGCTACCTGGTCCGATACTGACGCTGAGGTGCGAAAGCGTGGGGAGCAAACAGG
## 1340                                                        CCCAGCGCCACGAGTGGTTATCACGTTTATTGGGCCTAAAGCGTTCGTAGCCGGTTTGGTACATCTCTTGTGAAATTGTTCTGCTTAACAGGACAGCGTGCAGGAGAGACGGCCAGACTCGAGACTGGGAGGGGTTAGAAGTATGTCGTGGGGACTGGTAAAATGGGATAATCCACGATAGACTACCGATGGCGAAGGCATCTAACCAGAACAGTTCTGACGGTGAGGAACGAAAGCCAGGGGAGCGAACCGG
## 1341                                                         ACGTAGGGTGCAAGCGTTAATCGGAATTACTGGGCGTAAAGCGTGCGCAGGCGGTTATGTAAGACAGAGGTGAAATCCCCGGGCTCAACCTGGGAACTGCCTTTGTGACTGCATAGCTAGAGTACGGTAGAGGGGGATGGAATTCCGCGTGTAGCAGTGAAATGCGTAGATATGCGGAGGAACACCGATGGCGAAGGCAGCCTCCTGGGCCAGTACTGACGCTCATGCACGAAAGCGTGGGGAGCAAACAGG
## 1343                                                         ACGGAGGATGCGAGCGTTATCCGGAATCATTGGGTTTAAAGGGTCCGTAGGCGGTTTAATAAGTCAGTGGTGAAATCTGGTCGCTCAACGATCAAACTGCCATTGATACTGTTAGACTTGAATAATTGTGAAGTAACTAGAATATGTAGTGTAGCGGTGAAATGCTTAGATATTACATGGAATACCAATTGCGAAGGCAGGTTACTAACAATTTATTGACGCTGATGGACGAAAGCGTGGGGAGCGAACAGG
## 1344                                                         ACGTAGGTGGCGAGCGTTATCCGGAATTATTGGGCGTAAAGAGCGCGCAGGTGGTTGATTAAGTCTGATGTGAAAGCCCACGGCTTAACCGTGGAGGGTCATTGGAAACTGGTCGACTTGAGTGCAGAAGAGGGAAGTGGAATTCCATGTGTAGCGGTGAAATGCGTAGAGATATGGAGGAACACCAGTGGCGAAGGCGGCTTCCTGGTCTGTAACTGACACTGAGGCGCGAAAGCGTGGGGAGCAAACAGG
## 1345                                                         ACGAAGGGTGCAAGCGTTGCTCGGAATTATTGGGCGTAAAGGGTAGGTAGGTGGTGATACAAGTCTGGAGTGAAATCCCTGAGCTCAACTCAGGACGTGCTTTGGAAACTGTATCACTAGAGTGCTAGAGAGGTTCGTGGAATTCCTGGTGTAGCGGTGAAATGCGTAGAGATCAGGAGGAACATCTGCGGCGAAGGCGGCGAACTGGATAGTTACTGACACTCAACTACGAAAGCGTGGGGAGCAAACAGG
## 1346                                                         ACGGAGGGTGCGAGCGTTAATCGGAATTACTGGGCGTAAAGGGTGCGTAGGTGGTTTGATAAGTTATCTGTGAAAGCCCTGGGCTCAACCTGGGAACTGCAGATAAGACTGTTAGACTCGAGTATAGGAGAGGGTAGTGGAATTTCCGGTGTAGCGGTGAAATGCGTAGAGATCGGAAGGAACACCAGTGGCGAAGGCGGCTACCTGGCCTAATACTGACACTGAGGCACGAAAGCGTGGGGAGCAAACAGG
## 1347                                                         ACGGAGGGAGCTAGCGTTGTTCGGAATTACTGGGCGTAAAGCGCACGTAGGCGGCGACTCAAGTCAGAGGTGAAAGCCTGGAGCTCAACTCCAGAACTGCCTTTGAAACTAGGTCGCTAGAATCTTGGAGAGGCGAGTGGAATTCCGAGTGTAGAGGTGAAATTCGTAGATATTCGGAAGAACACCAGTGGCGAAGGCGACTCGCTGGACAAGTATTGACGCTGAGGTGCGAAAGCGTGGGGAGCAAACAGG
## 1350                                                         ACAGAGAGTGCAAACGTTGTTCGGAATTACTGGGCTTAAAGCGCACGTAGGCTGCGTCGCAAGTGTCAGGTGAAATCCCACGGCTTACCCGTGGAACTGCCTGGCAGACTGCGATGCTAGAGTGTGGTAGGGGAGAGCGGAACTCCTGGTGGAGCGGTGAAATGCGTAGATATCAGGAGGAACACCGGTGGCGAAGGCGGCTCTCTGGTCCACAACTGACGCTGAGGTGCGAAAGCTAGGGGAGCAAACGGG
## 1352                                                         ACGAAGGGGGCTAGCGTTGCTCGGAATTACTGGGCGTAAAGGGAGCGTAGGCGGACTGTTTAGTCAGAGGTGAAAGCCCAGGGCTCAACCTTGGAATTGCCTTTGATACTGGCAGTCTTGAGTACGGAAGAGGTATGTGGAACTCCGAGTGTAGAGGTGAAATTCGTAGATATTCGGAAGAACACCAGTGGCGAAGGCGACATACTGGTCCGTTACTGACGCTGAGGCTCGAAAGCGTGGGGAGCAAACAGG
## 1353                                                         ACGAAGGGTGCAAGCGTTACTCGGAATTACTGGGCGTAAAGGGTGCGTAGGTGGTTTTTTAAGTCTGCTGTGAAATCCCCGGGCTCAACCTGGGAATGGCAGTGGATACTGGAAAGCTGGAGTGCGGTAGAGGGTAGTGGAATTCCCGGTGTAGCAGTGAAATGCGTAGAGATCGGGAGGAACACCAGTGGCGAAGGCGGCTACCTGGACCAGCACTGACACTGAGGCACGAAAGCGTGGGGAGCAAACAGG
## 1354                                                         ACGTAGGGTGCAAGCGTTAATCGGAATTACTGGGCGTAAAGCGTGCGCAGGTGGTTCGTTAAGACAGTTGTGAAATCCCCGGGCTTAACCTGGGAACTGCAATTGTGACTGGCGGACTAGAGTTTGGCAGAGGGGGGTGGAATTCCTGGTGTAGCAGTGAAATGCGTAGATATGCGGAGGAACACCGATGGCGAAGGCAATCCCCTGGGCCTGTACTGACGCTCATGCACGAAAGCGTGGGGAGCAAACAGG
## 1355                                                         ACAGAGGTCTCAAGCGTTGTTCGGAATCACTGGGCGTAAAGGGTGCGTAGGTGGCGTGGCAAGTCAGATGTGAAAGCCCGGGGCTCAACCTCGGAATTGCATCCGATACTGCCATGCTAGAGTACTGAAGAGGTGACTAGAATTCTAGGTGTAGCAGTGAAATGCGCAGATATCTAGAGGAATACCAACGGCGAAGGCAGGTCACTGGGCAGTTACTGACACTGAGGCACGAAGGCCAGGGGAGCAAACGGG
## 1356                                                         ACGTATGGAGCAAGCGTTATCCGGATTTACTGGGTGTAAAGGGAGTGTAGGTGGCCAGGCAAGTCAGAAGTGAAAGCCCGGGGCTCAACCCCGGGACTGCTTTTGAAACTGCAGGGCTAGAGTGCAGGAGGGGCAAGTGGAATTCCTAGTGTAGCGGTGAAATGCGTAGATATTAGGAGGAACACCAGTGGCGAAGGCGGCTTGCTGGACTGTAACTGACACTGAGGCTCGAAAGCGTGGGGAGCAAACAGG
## 1357                                                         ACGAGTACCCCGAGCATTATCCGGAATTATTGGGCGTAAAGGGTGAGTAGGCGGTGCAATTAGTCGTGTGTTAAATCCCAGAGCTTAACTTTGGATTCGCACACGAAACGGTTGCACTAGAGGGTGTGAGAGGTGAATGGAACTCATAGTGTAGGGGTGAAATCCGTTGATATTATGGGGAACACCAAATGCGAAGGCAATTCACTGGCACATTTCTGACGCTCAATCACGAAAGCGTAGGTAGCGAATGGG
## 1364                                                         ACGTAGGGTGCGAGCGTTGTCCGGAATTACTGGGCGTAAAGAGCTCGTAGGTGGTTTGTCGCGTTGTTCGTGAAATCTCACGGCTTAACTGTGAGCGTGCGGGCGATACGGGCAGACTGGAGTACTGCAGGGGAGACTGGAATTCCTGGTGTAGCGGTGGAATGCGCAGATATCAGGAGGAACACCGGTGGCGAAGGCGGGTCTCTGGGCAGTAACTGACGCTGAGGAGCGAAAGCGTGGGGAGCGAACAGG
## 1365                                                         ACGAAGGTGGCGAGCGTTACCCGGATTGACTGGGCGTAAAGCGTCCGCAGACGGACAACCAAGTCTTTGGTTAAATCCTGAGGCTCAACCTCAGGACCGCTAAAGATACTGTTTGTCTAGAGGCTGGGAGAGGCAAGCGGAATTACTGGTGTAGTAGTAAAATGCGTTAATATCAGTAAGAACACCAAAAGCGAAGGCAGCTTGCTAGAACAGTCCTGACGTTCAGGGACGAAAGCGTGGGGAGCGAATGGG
## 1368                                                         ACGGAGGGTGCAAGCGTTATCCGGAATCACTGGGTTTAAAGGGTGCGTAGGCGGATTATTAAGTCAGTGGTGAAATCCTGTCGCTTAACGATAGAATTGCCATTGATACTGATAATCTTGAATTGGGTTGAGGTTAGCGGAATGTGACATGTAGCGGTGAAATGCATAGATATGTCATGGAACACCAATTGCGAAGGCAGCTAGCTGGGCCTTGATTGACGCTGAGGCACGAAAGCGTGGGTAGCGAACAGG
## 1369                                                         ACAGAGGGGGCTAGCGTTGTTCGGAATGACTGGGCGTAAAGGGCGCGTAGGTGGTATGACAAGTCAGGTGTGAAAGACCCAGGCTTAACCTGGGAACGGCATTTGAGACTGTCATGCTTGAGTCTGGGAGAGGATAGCGGAATTCCTAGTGTAGAGGTGAAATTCGTAGATATTAGGAAGAACACCGGTGGCGAAGGCGGCTATCTGGAACAGAACTGACACTGAGGCGCGAAAGCGTGGGGAGCAAACAGG
## 1370                                                         ACGGAGGGTGCAAGCGTTGTTCGGAATTACTGGGCGTAAAGCGTACGTAGGCGGTTGAATAAGTCAAATGTTAAAGCCCGGGGCTCAACCCCGAGGATGCATTTGATACTGTTCGACTAGAGGGCGATAGAGGAAAGCGGAATTCCGAGTGTAGCGGTGAAATGCGTAGATATTCGGAGGAACATCGGTAGCGAAAGCGGCTTTCTGGGTCGCTCCTGACGCTGAGGTACGAAAGCGTGGGTAGCAAACGGG
## 1371                                                         ACAGAGGCCACGAGCGTTAGTCGGAATCACTGGGCTTAAAGGGTGCGTAGGCGGGCCCGCAAGCGTCTTGTGAAATCCCATGGCTCAACCATGGAATCGCAGGGCGAACTGCGGGTCTTGAGGCAGGTAGGGGCCGGAAGAACAGTAGGTGGAGCGGTGAAATGCGTAGATATCTACTGGAATGCCGATGGGGAAGCCGTCCGGCTGGGCCTGTCCTGACGCTGAGGCACGAAAGCGTGGGGAGCAAACAGG
## 1372                                                         ACGGAGGGTGCAAACGTTGCTCGGAATTATTGGGCGTAAAGCGCACGTAGGCGGTTCGTTATGTCGGATGTGAAAGCCCTGGGCTTAACCCAGGAAGTGCATCCGAAACTGGCGAGCTTGAGTACGGAAGAGGGTCTCGGAATTCCCGGTGTAGAGGTGAAATTCGTAGATATCGGGAGGAACACCAGTGGCGAAGGCGGAGACCTGGGCCGATACTGACGCTGAGGTGCGAAAGCGTGGGGAGCAAACAGG
## 1374                                                        ACAGAGACCCCAAGCGTTATCCGGAATTATTGGGCGTAAAGGGTGAGTAGGTGGTTTTATTAGTCAGATGTTAAATTTCTCGGGCTTAACCTGAGAACTGCATTTGAAACGGTAGAACTAGAATTTTGGAGAGGTCTGTGGAACTATATGTGTAGGGGTAAAATCCGTTGATATATATGGGAACACCTAAAGCGAAGGCAGCAGACTGGCCAAACATTGACACTGAATCACGAAAGCGTGGGTAGCGATACGG
## 1376                                                        ACGGGGGGTGCAAGCGTTGTCCGGAATGATTGGGCGTAAAGCATCTGTAGGCGGTTTCTAAAGTCAACTGTTAAAGCCCAGGGCTTAACTTTGGAAAAGCGGTTGAGTACTTAGGAACTTGAGTTTGGTAGGGGTAAAGGGAATTCCTAGTGGAGCGGTGAAATGCACAGAGATTAGGAAGAACACCGATGGCGAAAGCACTTTACTGGGCCAAAACTGACGCTGAGAGATGAAAGTTGGGGGAGCGAATAGG
## 1377                                                         ACGTAGGGTGCGAGCGTTAATCGGAATTACTGGGCGTAAAGCGTGCGCAGGCGGCGTCGCAAGTCAGGCGTGAAATCCCCGAGCTTAACTTGGGAATTGCGCTTGAAACTACGATGCTTGAGTGTGGCAGAGGGAGGTGGAATTCCACGTGTAGCGGTGAAATGCGTAGATATGTGGAGGAACACCGATGGCGAAGGCAGCCTCCTGGGCCAACACTGACGCTCATGCACGAAAGCGTGGGGAGCAAACAGG
## 1378                                                         ACAGAGACTGCAAGCGTTACTCGGATTCACTGGGCGTAAAGGGAGCGCAGGCGGGCGGGTGTGTCATATGTGAAATCCCGGGGCTCAACCCCGGGGCTGCATCTGAAACTACCTGTCTAGAGATTCGGAGGGGTAAGCGGAATTCCTGGTGGAGCAGTGAAATGCGTAGATATCAGGAGGAACACCAACGGCGAAGGCAGCTTACTGGACGAAATCTGACGCTCAGGCTCGAAAGCATGGGGAGCAAACAGG
## 1380                                                         ACAGAGACCTCGAGCGTTATCCGGAATTACTGGGCGTAAAGCGCGAGCAGCCGGTTGAGTTAGTCCGATGTTAAATCCCAAGGCTTAACTTTGGAACCGCATTGGAAACGGCTCAACTCGAGTGGGTAAGAGGCACGCGGAACGTGCAGTGTAGGGGTGAAATCCATTGATATTGCACGGAACACCAAAAGCGAAGGCAGCGTGCTAGTGCTCAACTGACGGTCATTCGCGAAAGCGTGGGGATCAAAAAGG
## 1381                                                         ACGTAGGGGGCAAGCGTTATCCGGATTTACTGGGTGTAAAGGGAGCGTAGACGGTGTGGCAAGTCTGATGTGAAAGGCATGGGCTCAACCTGTGGACTGCATTGGAAACTGTCATACTTGAGTGCCGGAGGGGTAAGCGGAATTCCTAGTGTAGCGGTGAAATGCGTAGATATTAGGAGGAACACCAGTGGCGAAGGCGGCTTACTGGACGGTAACTGACGTTGAGGCTCGAAAGCGTGGGGAGCAAACAGG
## 1382                                                        ACGAACCGTCCAAACGTTATTCGGAATCACTGGGCTTAAAGGGCGCGTAGGCGGCCGAGCGGGTCGTGGGTGAAATCCCCCAGCTTAACTGGGGAACTGCCCTTGATACCACTGCGGCTCGAGGAAGGAAGGGGCAATCGGAACTGTCGGTGGAGCGGTGAAATGCGTTGATATCGACAGGAACGCCGGTGGCGAAAGCGGATTGCTGGTCCTTTTCTGACGCTGAGGCGCGAAAGCCAGGGGAGCAAACGGG
## 1384                                                         ACGAAGGGGGCTAGCGTTGTTCGGATTTACTGGGCGTAAAGCGCTCGTAGGTGGTTTGACAAGTCAGTGGTGAAATCCCAAGGCTCAACCTTGGAACTGCCATTGAAACTGTCAGACTGGAATCTCAGAGGGGGTGGCGGAATTGCTAATGTAGGGGTGAAATCCGTAGATATTAGCAGGAACACCGATGGCGAAGGCAGCCACCTGGATGAGTATTGACACTGAGGAGCGAAAGCGTGGGGATCAAACAGG
## 1385                                                         ACGGAGGATCCAAGCGTTATCCGGATTTATTGGGTTTAAAGGGTGCGTAGGCGGAATAGTAAGTCAGTGGTGAAAGCCTGTCGCTTAACGACAGAACTGCCATTGATACTGCTAATCTTGAGTATAATTGAAGTGGGCGGAATGTGTCGTGTAGCGGTGAAATGCTTAGATATGACACAGAACACCAATTGCGAAGGCAGCTCACTAAACTATAACTGACGCTGAGGCACGAAAGCGTGGGGATCAAACAGG
## 1387                                                         ACAGAGGTCTCAAGCGTTGTTCGGAATCACTGGGCGTAAAGGGTGCGTAGGTGGCGTGGTAAGTCAGATGTGAAAGCCCGGGGCTCAACCTCGGAACTGCATCCGATACTACCATGCTAGAGTACTGAAGAGGAAACTAGAATTCTCGGTGTAGCAGTGAAATGCGTAGATATCGAGAGGAATACCAAAGGCGAAGGCAGGTTTCTTGGCAGTTACTGACACTGAGGCACGAAGGCCAGGGGAGCGAACGGG
## 1388                                                         ACGTAGGGGGCAAGCGTTATCCGGATTTACTGGGTGTAAAGGGAGCGTAGACGGCAAGGCAAGTCTGATGTGAAAACCCAGGGCTTAACCCTGGGACTGCATTGGAAACTGTCTGGCTCGAGTGCCGGAGAGGTAAGCGGAATTCCTAGTGTAGCGGTGAAATGCGTAGATATTAGGAAGAACACCAGTGGCGAAGGCGGCTTACTGGACGGTAACTGACGTTGAGGCTCGAAAGCGTGGGGAGCAAACAGG
## 1389                                                         ACGTAGGATGCAAGCGTTGTCCGGATTTATTGGGCGTAAAGAGTTCGTAGGCGGTTTGTTAAGTCTGATGTTAAAGACCGGGGCTCAACCTCGGAAATGCATTGGATACTGGCAGACTGGAGTGCAGTAGAGGCTAGTGGAATTCCCAGTGTAGCGGTGAAATGCGTAGATATTGGGAAGAACACCGGTGGCGTAGGCGACTAGCTGGGCTGTAACTGACGCTGAGGAACGAAAGCCAGGGGAGCGAATGGG
## 1391                                                         ACGTAGGGTGCAAGCGTTGTCCGGAATTATTGGGCGTAAAGAGCTCGTAGGCGGTTTGTCACGTCTGCTGTGAAATTTCGAGGCTCAACCTCGAACTTGCAGTGGGTACGGGCAGGCTAGAGTGCGGTAGGGGAGATGGGAATTCCTGGTGTAGCGGTGGAATGCGCAGATATCAGGAGGAACACCAATGGCGAAGGCACATCTCTGGGCCGTAACTGACGCTGAGGAGCGAAAGCGTGGGGAGCAAACAGG
## 1394                                                         ACGAAGGGGGCTAGCGTTGCTCGGAATTACTGGGCGTAAAGGGCGCGTAGGCGGATAGTTTAGTCAGAGGTGAAAGCCCAGGGCTCAACCTTGGAATTGCCTTTGATACTGGCTATCTTGAGTATGGAAGAGGTATGTGGAACTCCGAGTGTAGAGGTGAAATTCGTAGATATTCGGAAGAACACCAGTGGCGAAGGCGACATACTGGTCCATTACTGACGCTGAGGCGCGAAAGCGTGGGGAGCAAACAGG
## 1396                                                         ACAGAGGGTGCGAACGTTGCTCGGAATTACTGGGCGTAAAGCGCGTGTAGGCGGGCTAGCAAGTCAGATGTGAAATCCCCGGGCTCAACCCGGGAACTGCATCTGAAACTGCTGGCCTTGAGTGCCGGAGAGGGTGGCGGAATTCCTGGTGTAGAGGTGAAATTCGTAGATATCAGGAGGAACATCAGTGGCGAAGGCGGCCACCTGGACGGCAACTGACGCTGAGACGCGAAAGCGTGGGGAGCAAACAGG
## 1397                                                         ACGTAGGGTGCAAGCGTTAATCGGAATTACTGGGCGTAAAGCGTGCGCAGGCGGTTATATAAGACAGATGTGAAATCCCCGGGCTCAACCTGGGAACTGCATTTGTGACTGTATAGCTAGAGTACGGTAGAGGGGGATGGAATTCCGCGTGTAGCAGTGAAATGCGTAGATATGCGGAGGAACACCGATGGCGAAGGCAGTCCCCTGGGATAAGACTGACGCTCATGCACGAAAGCGTGGGGAGCAAACAGG
## 1400                                                         ACGGAGGGTGCGAGCGTTATCCGGAATCACTGGGTTTAAAGGGTGCGTAGGCGGCTAGGTAAGTCAGGGGTGAAAGCTTCCCGCTCAACGGGAGAACTGCCTTTGATACTGCCTGGCTCGAATTGGGTTGAGGCTGGCGGAATGTGGCATGTAGCGGTGAAATGCTTAGATATGCCATAGAACACCGATTGCGAAGGCAGCCAGCCAAGCCTTGATTGACGCTGAGGCACGAAAGCGTGGGGAGCGAACAGG
## 1401                                                          ACGTAGGTCCCGAGCGTTGTCCGGATTTATTGGGCGTAAGGCGAGCGCAGGCGGTTTGATAAGTCTGAAGTTAAAGGCTGTGGCTCAACCATAGTTCGCTTTGGAAACTGTCAAACTTGAGTGCAGAAGGGGAGAGTGGAATTCCATGTGTAGCGGTGAAATGCGTAGATATATGGAGGAACACCGGTGGCGAAAGCGGCTCTCTGGTCTGTAACTGACGCTGAGGCTCGAAAGCGTGGGGAGCAAACAGG
## 1403                                                        CCCAGCGGTCCAAGTCGCAGCCACATTTGTTGGGTCTAAAACATCCGTAGCTTGCTTTTCAAGTCTTTTGTGAAATCGGGACTCTTAAGGTTCCGGCGTGCAAAAGATACTGTTAAGCTAGAGACCGGGAGGCGTAAGAAGTACGTATAGGGTAGCGGTAAAATGCGTTGATCCTATGTGGACTCACAGTCGCGAAGGCGTCTTACGAGAACGGTTCTGACAGTGAGGGATGAAGGCTAGGGGCGCAAAACGG
## 1404                                                        ACGAGGGGAGCAAGTGTTATACAGTCAAACTAGGCGTAAAGGGTTCTGCGGCGGAGTAATGAGAGATCTTCAGAGATACGGTGGTTGTTACCCATCTGCATGAAGATGACCATTTCTCTTAGTGTTATGTAGGGGTTGGAAGGACTCCGCGCAAAGCGTTGCAATGCGATGACCCGCGGGGGCCAACCAGTAGAGAAGTCGTCCAACTAGACATGACAGACGCTTAGGAACAAAAGCATGGGAAGCAAATGGG
## 1406                                                         ACGTAGGGTGCAAGCGTTAATCGGAATTACTGGGCGTAAAGCGTGCGCAGGCGGTTATGCAAGACAGATGTGAAATCCCCGGGCTCAACCTGGGAACTGCATTTGTGACTGCATAGCTAGAGTACGGTAGAGGGGGATGGAATTCCGCGTGTAGCAGTGAAATGCGTAGATATGCGGAGGAACACCGATGGCGAAGGCAATCCCCTGGGCCTGTACTGACGCTCATGCACGAAAGCGTGGGGAGCAAACAGG
## 1408                                                         ACGAACCGTGCGAACGTTGTTCGGAATCACTGGGCTTAAAGGGTGCGTAGGCGGAATGGCAAGTCGGAGGTGAAAGCCCCCAGCTTAACTGGGGAAGTGCCCTCGATACTGCTGTTCTCGAGAGAGGTAGGGGCGTGTGGAACTTCCGGTGGAGCGGTGAAATGCGTAGATATCGGAAGGAACGCCGGTGGCGAAAGCGATGCGCTGGACCTTTTCTGACGCTGAGGCACGAAAGCTAGGGGAGCGAACGGG
## 1409                                                         ACGAGGGGTGCAAGCGTTGTTCGGAATCACTGGGCGTAAAGCGCGTCTAGGCGGGACAGAAAGTCTGATGTGAAAGTTCTTGGCTCAACCAAGAAAATGCGTCGGAGACTTCTGATCTTGAGTCATGGAGGGATGGGCGGAATTCCGGGTGTAGCAGTGAAATGCGTAGAGATCCGGAGGAACATCAGAGGCGAAGGCGGCTCATTGGCCATGTACTGACGCTAAAGCGCGAAAGCGTGGGTAGCAAACAGG
## 1410                                                         ACGGAGGGTGCAAGCGTTATCCGGATTCACTGGGTTTAAAGGGTGCGTAGGTGGATCAGTAAGTCAGTGGTGAAAGCCCCGGGCTCAACTTGGGAATTGCCATTGATACTGCTGGTCTTGAGTTCGGTTGAGGTAGGCGGAATAAGTCATGTAGCGGTGAAATGCTTAGATATGACTTAGAACACCGATTGCGAAGGCAGCTTACCAAGCCGACACTGACACTGAGGCACGAAAGCGTGGGGATCAAACAGG
## 1412                                                         ACGTAGGGTGCGAGCGTTAATCGGAATTACTGGGCGTAAAGCGTGCGCAGGTGGTTGTGTAAGTCAGATGTGAAATCCCCGGGCTCAACCTGGGAACTGCATTTGAGACTGCACGGCTAGAGGATGGCAGAGGGGGGTAGAATTCCACGTGTAGCAGTGAAATGCGTAGAGATGTGGAGGAATACCGATGGCGAAGGCAGCCCCCTGGGCTATTTCTGACACTCATGCACGAAAGCGTGGGGAGCAAACAGG
## 1417                                                         ACGAAGGTGGCAAGCGTTACTCGGAATTACTAGGCGTAAAGGGCAGGTAGGTGGTTCGGCAAGTCTGTTGTGGAAGCTCCCGGCTTAACTGGGAGAGGCCAACGGAAACTGCCGGACTTGAGTGTAGGAGAGGGTATTGGAATTCCCGGTGTAGCGGTGAAATGCGCAGAGATCGGGAGGAACACCAATGGCGAAAGCAGATACCTGGACTATTACTGACACTAAGCTGCGAAAGCTAGGGGAGCAAACAGG
## 1418                                                         ACGTAGGGTGCGAGCGTTAATCGGAATTACTGGGCGTAAAGCGTGCGCAGGCGGTTGTATAAGTCAGATGTGAAATCCCCGGGCTCAACCTGGGAACTGCATTTGAGACTGTACGGCTAGAGTGTGGCAGAGGGGGGTGGAATTCCACGTGTAGCAGTGAAATGCGTAGAGATGTGGAGGAACACCGATGGCGAAGGCAGCCCCCTGGGCCAATACTGACGCTCATGCACGAAAGCGTGGGGAGCAAACAGG
## 1419                                                        ACGTAGGACCCAAGCGTTATCCGGAATTACTGGGCGTAAAGAGTTGCGTAGGCGGCTTGTTAAGCAAGATGTGAAATCGTGAGGCTCAACCTTATACACATATTTTGAACTGGCAAGCTAGAGTATAGAAGAGGTCACTGGAATTCCAAGTGTAGGAGTGAAATCCGTAGATATTTGGAGGAACACCGATGGCGTAGGCAGGTGACTGGTCTATTACTGACGCTAAGGCACGAAAGCGTGGGGAGCAAACGGG
## 1421                                                         ACGAAGGTGGCGAGCGTTACTCGGAATTACTAGGCGTAAAGCGTGGGCAGGCGGTTTGGTAAGTCTGCTGTGAAAGCCCCGGGCTCAACCCGGGGAGGTCAGTGGATACTGCCGGACTTGGATGTGGGAGAGGTGACTGGAATTCCTGGTGTAGCGGTGAAATGCGTAGATATCAGGAGGAACACCGATGGCGAAAGCAGGTCACTGGACCACTATCGACGCTCATCCACGAAAGCTGGGGGATCAAACAGG
## 1423                                                         ACGGAGGGTGCGAGCGTTTATCGGAATTACTGGGCGTAAAGCGCGCGTAGGCGGCTTGATAAGTCGGATGTGAAATCCCCGGGCTCAACCTGGGAACTGCATCCGATACTGTTGAGCTGGAGTACGATAGAGGATGGTAGAATTTCTAGTGTAGCGGTGAAATGCGTAGATATTAGAAGGAATACCGGTGGCGAAGGCGGCCATCTGGATCGATACTGACGCTGAGGTGCGAAAGCGTGGGGATCAAACAGG
## 1425                                                         ACAGAGAGTGCGAACGTTGTTCGGAATCACTGGGCATAAAGCGCACGTAGGCGGCACCATAAGTGCGAGGTGAAATCCCACGGCTTACCCGTGGAACTGCTTTGCAGACTGTGGTGCTCGAGGATGGTAGGGGAGAGCGGAACTCTTGGTGGAGCGGTGAAATGCGTAGAGATCAAGAGGAACACCGGTGGCGAAGGCGGCTCTCTGGTCCATATCTGACGCTGAGGTGCGAAAGCTAGGGGAGCAAACGGG
## 1427                                                         ACGTAGGTGGCAAGCGTTATCCGGATTTACTGGGTGTAAAGGGCGCGTAGGCGGGAAGGCAAGTCAGATGTGAAATCCAGGGGCTCAACCCCTGAACTGCATTTGAAACTGCTTTTCTTGAGTGTCGGAGAGGTAGATGGAATTCCTAGTGTAGCGGTGAAATGCGTAGATATTAGGAGGAACACCAGTGGCGAAGGCGATCTACTGGACGATAACTGACGCTGAGGCGCGAAAGCGTGGGGAGCAAACAGG
## 1428                                                         ACAGAGAGTGCAAGCGTTAATCGGAATTATTGGGTGTAAAGGGTGTGTAGGTGGATAAAAAAGTTATATGTGAAATCCCTAAGCTCAACTTAGGAACTGCATGTAATACTTTTTATCTAGAGTATTACAGAGGAAAGTAGAATTTCCGGTGTAGCGGTGAAATGCGTAGATATCGGAAGGAATACCAGAGGCGAAGGCGACTTTCTGGGTAATTACTGACACTGATACACGAAAGCGTGGGGAGCAAACAGG
## 1429                                                         ACGAAGGGTGCAAGCGTTGCTCGGAATTATTGGGCGTAAAGGGTAGGTAGGTGGTCTTATTTGTCTAGGGTGAAATCCCAGGGCTTAACCCTGGAAGTGCCTTAGAAACGGTAGGACTAGAGTTCTAGAGAGGGTCGTGGAATTCCCGGTGTAGCGGTGAAATGCGTAGAGATCGGGAGGAACACCAGAAGCGAAGGCGACGACCTGGATAGATACTGACACTCAACTACGAAAGCGTGGGGAGCAAACAGG
## 1430                                                         ACGGAGGATGCGAGCGTTATCCGGATTTATTGGGTTTAAAGGGTGCGTAGGCCGACTTTTAAGTCAGCGGTGAAATTTTCCGGCTTAACCGGGACCTTGCCGTTGAAACTGATAGTCTTGAGTGTAAATGAGGTAGGCGGAATGTGTGGTGTAGCGGTGAAATGCTTAGATATCACACAGAACTCCGATTGCGAAGGCAGCTTACTGGCATACAACTGACGCTGAGGCACGAAAGTGTGGGTATCAAACAGG
## 1432                                                         ACGTAGGGTGCGAGCGTTAATCGGAATTACTGGGCGTAAAGCGTGCGCAGGCGGTTTCGTAAGACAGACGTGAAATCCCCGGGCTCAACCTGGGAACTGCGTTTGTGACTGCGAGGCTAGAGTATGGCAGAGGGGGGTGGAATTCCACGTGTAGCAGTGAAATGCGTAGAGATGTGGAGGAACACCGATGGCGAAGGCAGTCCCCTGGGATAAGACTGACGCTCATGCACGAAAGCGTGGGGAGCAAACAGG
## 1434                                                         ACGGAGGTGGCAAGCGTTACTCGGAATTATTGGGTGTAAAGGGCAAGTAGGCGGTTTATTAAGTGGGTGGTGAAATACTCCGGCTCAATCGGAGAACAGCCTTCCAAACTGATAGGCTTGAGTGTGACAGAGGAAATTGGAATTCTCGGTGTAAGGGTGAAATCTGTAGATATCGAGAAGAACACCAGCAGCGAAGGCGAATTTCTGGGTCACTACTGACGCTAAATTGCGAAAGCTAGGGGAGCAAACAGA
## 1436                                                         ACGGAGGGTGCAAGCGTTATCCGGATTCACTGGGTTTAAAGGGTGCGTAGGTGGGTTGGTAAGTCAGTGGTGAAATCCCCGAGCTTAACTTGGGAACTGCCATTGATACTATTAATCTTGAATACCGTGGAGGTCGGCGGAATATGTCATGTAGCGGTGAAATGCTTAGATATGACATAGAACACCAATTGCGAAGGCAGCTGGCTACACGAGTATTGACACTGAGGCACGAAAGCGTGGGGATCAAACAGG
## 1443                                                         ACGAAGGGTGCTAGCGTTGTTCGGAATTACTGGGCGTAAAGGGCGCGTAGACGGTTAGATAAGTTAGATGTGAAAGCCCTAGGCTTAACCTAGGATGTGCATTTAATACTGTTTAGCTAGAGTACAAGAGGGGAAAATGGAATTGCGTGTGTAGAGGTGAAATTCGTAGATATACGCAGGAACACCGGTGGCGAAGGCGATTTTCTGGCTTGATACTGACGTTAAGGCGCGAAAGCGTGGGGAGCAAACAGG
## 1444                                                         ACGTAGGGTGCGAGCGTTAATCGGAATCACTGGGCGTAAAGCGCTCGTAGGCGGTTCGGTCAGTCAGATGTGAAAGCCCTCGGCTTAACCGGGGAACTGCATTTGATACTGCCAGACTTGAGTGTCGGAGAGGGTGGCGGAATTCCGGGTGTAGGAGTGAAATCCGTAGATATCCGGAGGAACACCAGTGGCGAAGGCGGCCACCTGGACGACAACTGACGCTGAGGAGCGAAAGCGTGGGGAGCAAACAGG
## 1449                                                                                                              ACAGAAGGGGCAAGCATCTTGCAGAATGACTCGGCGAAGAGCGTGGTTTTGCAAAGTCGAATGAGACAAATTGTTTTGGAATTCTCAAAGAATGAGTCAAATCATTGGAGATTGGGAGGAACGCCAAAGGTGAAGACAGAAACACACAACATTCAAAAGCAGACCTACGGAAGCCCAGGGAGCAAAACGGATTAGATAC
## 1452                                                         ACGTAGGGTGCAAGCGTTGTCCGGAATTATTGGGCGTAAAGAGCTCGTAGGCGGTTTGTCACGTCTGCTGTGAAAATCCGAGGCTCAACCTCGGACTTGCAGTGGGTACGGGCAGGCTAGAGTGCGGTAGGGGAGACGGGAATTCCTGGTGTAGCGGTGGAATGCGCAGATATCAGGAGGAACACCGATGGCGAAGGCACGTCTCTGGGCCGTAACTGACGCTGAGGAGCGAAAGCGTGGGGAGCGAACAGG
## 1453                                                        ACGTAGAGTACAAGCGTTATCCGGATTTATTGGGCGTAAAGCGTATCGCAGGTGGAAATGTGCGTGGCGGGTGTAAGCCTTGGGCTTAACTCAAGAACCGCTCGTCATACGGCATTTCTGGAGTTTGTTGGAGGGAACTGGAATTCTTCATGGAGGGGTGAAATCCGTAGATATGAAGAGGAACGCCAAGGGCGAAGGCAAGTTCCTACGACAATACTGACACTCATGAACGACAGCTAGGGGAGCAAAAGGG
## 1454                                                         ACGAAGGGTGCTAGCGTTGTTCGGAATCATTGGGCGTAAAGCGTACGTAGGCGGTTTAGTAAGTCTGTCGTGAAATACCCGAGCTTAACTTGGGAATTGCGATAGAAACTGCTTTGCTGGAGTATAGTGGGGGATAGTGGAATTCCTAGTGTAGGGGTGAAATCCGTAGAGATTAGGAGGAACATCAGTGGCGAAGGCGACTATCTACGCTATTACTGACGCTAAGGTACGAAAGCGTGGGGAGCAAACAGG
## 1456                                                        ACGGAGGATCCAAGCGTTATCCGGATTTACTGGGCGTAAAGAGTTGCGTAGGTGGTTTGTTAAGTGAATAGTGAAATCTGGTGGCTCAACCATCAACACATTATTCAAACTGACAAACTCGAGAATGAGAGAGGTCACTGGAATTCCTTGTGTAGGAGTGAAATCCGTAGATATAAGGAGGAACACCAATGGCGTAGGCAGGTGACTGGCTCATTTCTGACACTGAGGCACGAAAGCGTGGGGAGCGAACGGG
## 1457                                                         ACGAAGGGTGCAAGCGTTAATCGGAATTACTGGGCGTAAAGCGCGCGTAGGTGGTTCAGCAAGTTGGATGTGAAAGCCCTGGGCTCAACCTGGGAACTGCATCCAAAACTACTGAGCTAGAGTACGGTAGAGGGTAGTGGAATTTCCTGTGTAGCGGTGAAATGCGTAGATATAGGAAGGAACACCAGTGGCGAAGGCGACTACCTGGACTGATACTGACACTGAGGTGCGAAAGCGTGGGGAGCAAACAGG
## 1460                                                         ACGTAGGGTGCAAGCGTTAATCGGAATTACTGGGCGTAAAGCGAGCGCAGGCGGTTATGTAAGACAGATGTGAAATCCCAGGGCTTAACCTTGGAACTGCATTTGTGACTGCATGGCTAGAGTGTGTCAGAGGGGGGTGGAACTCCACGTGTAGCAGTGAAATGCGTAGAGATGTGGAAGAACACCGATGGCGAAGGCAGCCCCCTGGGATAACACTGACGCTCAGGCTCGAAAGCGTGGGGAGCAAACAGG
## 1462                                                         ACGTAGGTGACAAGCGTTATCCGGAATTACTGGGCGTAAAGCGCACGCAGGCGGTGTTATAAGTCTGATGTGAAACCTTCTGGCTTAACCAGAAGACGTCGTTGGATACTGTAACACTTGAGGCGATGAGAGGAATGCGGAATTCTCGGTGTAATGGTGGAATATGTAGATATCGAGAGGAACATCTGTGGCGAAAGCGGCATTCTAGCATTAGCCTGACGCTCATGTGCGAAAGCGTGGGGAGCAAACGGG
## 1464                                                         ACGAAGGTCCCAAGCGTTATCCGGATTTATTGGGTTTAAAGGGTGCGTAGGCGGACTATTAAGTCAGTGGTGAAAGCCCCAAGCTTAACTTGGGAACTGCCATTGATACTGATGGTCTTGAGTACATTTGAGGTGGGCGGAATGTGTCATGTAGCGGTGAAATGCTTAGATATGACACAGAACACCGATTGCGAAGGCAGCTCACTAAACTGTAACTGACGCTGAGGCACGAAAGCGTGGGGAGCAAACAGG
## 1466                                                         ACGGAGGGGGCTAGCGTTGTTCGGAATTACTGGGCGTAAAGCGTACGTAGGCGGTGTCTCAAGTCAGAGGTGAAAGCCTGGAGCTCAACTCCAGAACTGCCTTTGAAACTAGGACGCTAGAATTGCGGAGAGGTGAGTGGAATTCCGAGTGTAGAGGTGAAATTCGTAGATATTCGGAAGAACACCAGTGGCGAAGGCGGCTCACTGGACGCATATTGACGCTGAGGTACGAAAGCGTGGGGAGCAAACAGG
## 1467                                                        ACGGAGGATCCAAGCGTTATCCGGAATTACTGGGCGTAAAGAGTTGCGTAGGTGGCATTGTAAGTTAGTAGTGAAAGCGTGTGGCTCAACCATACTCACATTATTAAAACTGCAAAGCTAGAGTATATGAGAGGTAGCTGGAATTTCTAGTGTAGGAGTGAAATCCGTAGATATTAGAAGGAACACCGATGGCGTAGGCAGGCTACTGGCGTATTACTGACACTAAGGCACGAAAGCGTGGGGAGCGAACAGG
## 1470                                                         ACGAAGGATCCAAGCGTTGTCCGGATTTACTGGGTTTAAAGGGAGCGTAGGCGGTCTCATAAGTCAGTGGTGAAAGCCTTCAGCTTAACTGAAGAATTGCCATTGAAACTGCGAGACTCGAGTATGGTTGAGGTCACTGGAATATAACATGTAGCGGTGAAATGCTTAGATATGTTATAGAACACCAATTGCGAAGGCAGGTGACTAAGCCATAACTGACGCTGAGGCTCGAAAGCGTGGGGAGCAAACAGG
## 1471                                                         ACAGAGGGTGCAAACGTTGCTCGGAATTATTGGGCGTAAAGCGCGTGTAGGCGGCCCGTTAAGTCGGTTGTGAAAGCCCATGGCTCAACCATGGAAGTGCGACTGAAACTGGCGGGCTGGAGTCCTGAAGAGGGTGGCGGAATTCCCAGTGTAGAGGTGAAATTCGTAGATATTGGGAGGAACACCGGTGGCGAAGGCGGCCACCTGGGCAGGCACTGACGCTGAGACGCGAAAGCGTGGGGAGCAAACAGG
## 1472                                                         ACGGAGGGTGCAAGCGTTAATCGGAATTACTGGGCGTAAAGCGAGTGTAGGCGGCTTCGTAAGTCGGATGTGAAAGCCCTGGGCTTAACCTGGGAATTGCATTCGATACTGCGCGGCTAGAGTCTGATAGAGGGAAGTGGAATTCCAGGTGTAGCGGTGAAATGCGTAGATATCTGGAGGAACACCAGTGGCGAAGGCGGCTTCCTGGATCAAGACTGACGCTGAGGCTCGAAAGCGTGGGGATCAAACAGG
## 1473                                                         ACGTAGGATCCGAGCGTTGTCCGGAATTACTGGGTGTAAAGGGTGCGTAGGCGGACTTGTGCGTCAGAGGTGAAATCCACGGGCTTAACCCGTGGGGTGCCTTTGATACGGCAAGTCTTGAGTGCGAGAGAGGTGGGTGGAATTCCTGGTGTAGCAGTGAAATGCGTAGATATCAGGAGGAACACCGATGGCGAAGGCAGCCCACCGGCTCGCAACTGACGCTGAGGCACGAAAGCGTGGGGATCAAACAGG
## 1476                                                         ACGAGAGGTGCAAGCGTTATTCGGAATTATTGGGCGTAAAGGGTGCGTAGACGGTTTTTTAAGTCTCTTGTTTAATTCTCCGGCCTAACTGGGGGTCAGCGAGAGATACTGAATAACTAGAGAATGAAAGAGGGAAGTGGAATTCTCGGAGTAGCGGTTAAATGCGTAGATCTCGAGAGGAACACCGATGGCGAAAGCAGCTTCCTGGTTCATATCTGACGTTCAAGCACGAAAGCGTGGGGAGCAAACAGG
## 1479                                                          CAGAGGTGGCAAGCGTTGTTCGGAATTACTGGGCGTAAAGGGCGCGTAGGCGGCCTTGTAAGTCTCGTGTGAAAGCCCTCGGCTTAACTGAGGATCTGCACGGGATACTGCGAGGCTTGAGTTCGGGAGAGGAAAGCGGAATTCCGGGTGTAGCGGTGAAATGCGTAGATATCCGGAGGAACACCAGTGGCGAAGGCGGCTTTCTGGACCGACACTGACGCTGAGGCGCGAAAGCTAGGGGAGCAAACGGG
## 1484                                                          ACGTAGGTGGCAAGCGTTGTCCGGAATTATTGGGCGTAAAGGGAGCGCAGGTGGGAATATCAGTCTGTCTTAAAAGTTCGGGGCTCAACCCCGTGATGGGATGGAAACTATATTTCTTGAGTGCAGGAGAGGAAAGTGGAATTCCTAGTGTAGCGGTGAAATGCGTAGATATTAGGAGGAACACCAGTGGCGAAGGCGACTTTCTGGACTGTAACTGACACTGAGGCTCGAAAGCCAGGGTAGTGAACGGG
[truncated: 384,798 more chars]
