# Supplementary material for: Hurricane Harvey Impacts on Water Quality and Microbial Communities in Houston, TX Waterbodies
Source: Front Microbiol. 2022 Jun 14;13:875234. doi: 10.3389/fmicb.2022.875234 (PMC9239555; doi:10.3389/fmicb.2022.875234)
Supplement: Supplementary file 15 [file Data_Sheet_15.ZIP › S15 HHdisharge.html]

USGSflow


# USGSflow

#### Michael G. LaMontagne

#### 2022-04-28

## R Markdown

Some of the data that you have obtained from this U.S. Geological
Survey database  
may not have received Director’s approval. Any such data values are
qualified  
as provisional and are subject to revision. Provisional data are
released on the  
condition that neither the USGS nor the United States Government may be
held liable for any damages resulting from its use.

additional info: https://help.waterdata.usgs.gov/policies/provisional-data-statement

File-format description: https://help.waterdata.usgs.gov/faq/about-tab-delimited-output  
Automated-retrieval info: https://help.waterdata.usgs.gov/faq/automated-retrievals

Contact: gs-w\_support\_nwisweb@usgs.gov  
retrieved: 2022-04-25 10:52:24 EDT (nadww01)

Data for the following 1 site(s) are contained in this file USGS
08076997 Clear Ck at Mykawa St nr Pearland, TX

## Packages

```
library(lubridate)  #work with dates
```

```
## 
## Attaching package: 'lubridate'
```

```
## The following objects are masked from 'package:base':
## 
##     date, intersect, setdiff, union
```

```
library(ggplot2)
```

## Import

```
usgs8076997 <- as.data.frame(read.table("usgs8076997.csv", sep=",", header = TRUE, check.names = TRUE))
head(usgs8076997)
```

```
##   agency_cd site_no       datetime tz_cd g140509_00065 gage_metere
## 1      USGS 8076997 8/20/2017 0:00   CDT         31.17    9.500616
## 2      USGS 8076997 8/20/2017 0:15   CDT         31.17    9.500616
## 3      USGS 8076997 8/20/2017 0:30   CDT         31.17    9.500616
## 4      USGS 8076997 8/20/2017 0:45   CDT         31.17    9.500616
## 5      USGS 8076997 8/20/2017 1:00   CDT         31.17    9.500616
## 6      USGS 8076997 8/20/2017 1:15   CDT         31.17    9.500616
##   d140510_00060 discharge           Date Symbol
## 1          8.95 0.2534358 2017/8/20/0:00       
## 2          8.95 0.2534358 2017/8/20/0:15       
## 3          8.95 0.2534358 2017/8/20/0:30       
## 4          8.95 0.2534358 2017/8/20/0:45       
## 5          8.95 0.2534358 2017/8/20/1:00       
## 6          8.95 0.2534358 2017/8/20/1:15
```

## Import and format data

```
usgs8076997$DateHour = as.POSIXct(usgs8076997$Date, format="%Y/%m/%d/%H:%M")
usgs8076997$Symbol = as.POSIXct(usgs8076997$Symbol, format="%Y/%m/%d/%H:%M")
# Most basic bubble plot
p <- ggplot(usgs8076997, aes(x=DateHour, y=discharge)) + theme_classic() +
  geom_line() + xlab("") + ylab("Discharge (cubic meter /sec)") + geom_vline(xintercept = usgs8076997$Symbol,linetype="dotted", color = "blue", size=0.5 ) +
annotate('text', x = as.POSIXct("2017-08-20 CDT"), y = 60, label = 'pre') +
annotate('text', x = as.POSIXct("2017-08-25 CDT"), y = 140, label = 'a') +
annotate('text', x = as.POSIXct("2017-08-30 CDT"), y = 60, label = 'HH') +
annotate('text', x = as.POSIXct("2017-08-30 CDT"), y = 140, label = 'b') +
annotate('text', x = as.POSIXct("2017-09-08 CDT"), y = 140, label = 'c') +
annotate('text', x = as.POSIXct("2017-09-12 CDT"), y = 60, label = 'post') +
annotate('text', x = as.POSIXct("2017-09-18 CDT"), y = 140, label = 'd') +
annotate('text', x = as.POSIXct("2017-09-25 CDT"), y = 140, label = 'e') +
annotate('text', x = as.POSIXct("2017-10-02 CDT"), y = 140, label = 'f') +
annotate('text', x = as.POSIXct("2017-10-17 CDT"), y = 140, label = 'g') +
annotate('text', x = as.POSIXct("2017-10-30 CDT"), y = 140, label = 'h')
p
```

```
## Warning: Removed 7382 rows containing missing values (geom_vline).
```

## Import and format data

```
ggsave("Fig_S10_disharge.tiff", device = 'tiff', dpi=300, width = 6, height = 4, units = "in")
```

```
## Warning: Removed 7382 rows containing missing values (geom_vline).
```
