# Supplementary figures and images for: Hurricane Harvey Impacts on Water Quality and Microbial Communities in Houston, TX Waterbodies
Source: Front Microbiol. 2022 Jun 14;13:875234. doi: 10.3389/fmicb.2022.875234 (PMC9239555; doi:10.3389/fmicb.2022.875234)

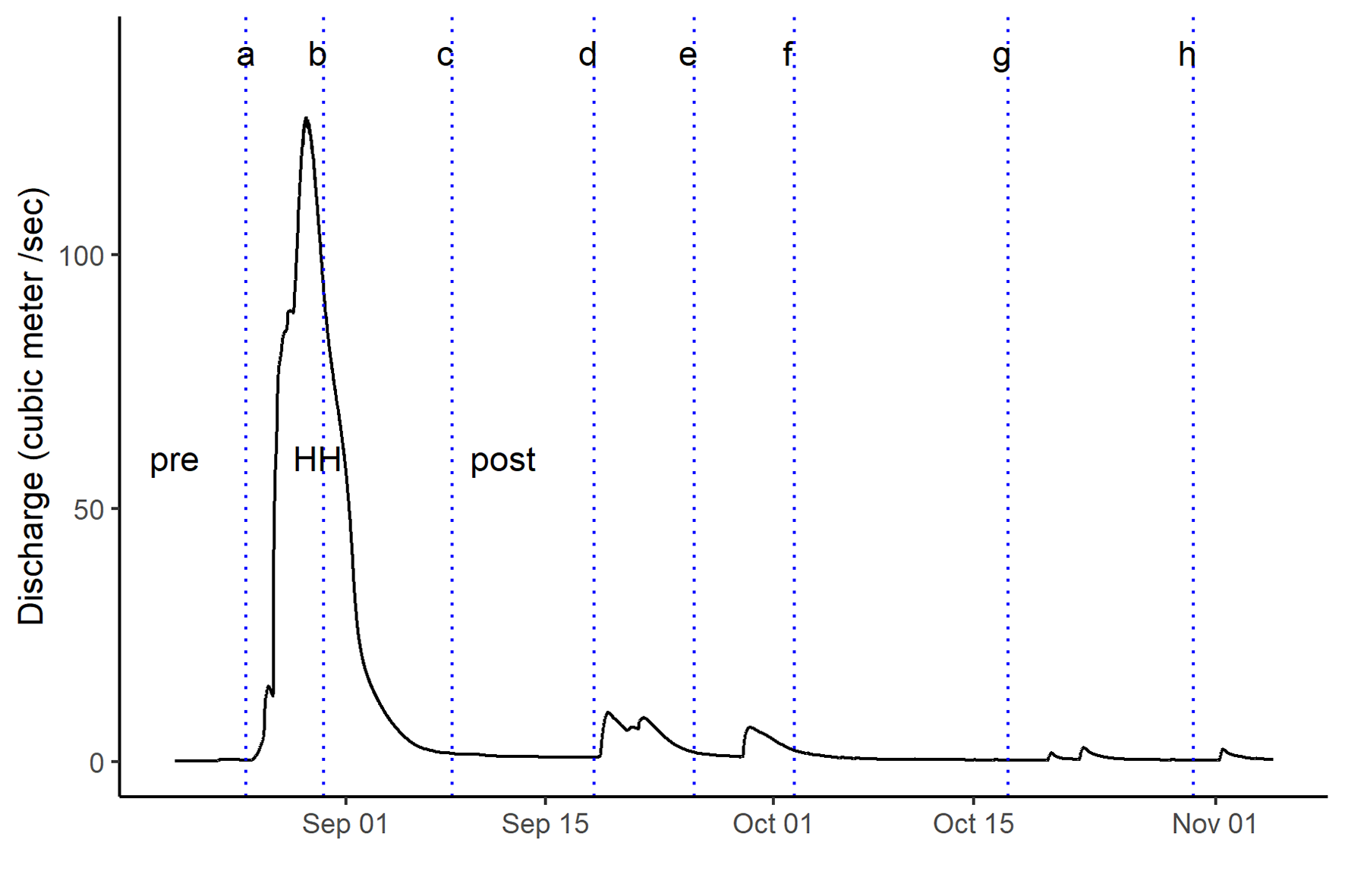

Supplement: Supplementary Figure 1 — Stream flow for Clear Creek. Data was downloaded for USGS station 08076997 from https://waterdata.usgs.gov/nwis/inventory?agency_code=USGS&site_no=08076997. Vertical lines indicate sampling dates. Lower case letters next to lines indicates dates, where a = August 25th, b = August 30th etc. Figure was generated with scripts in Supplementary File 15. [file Image_1.TIFF]

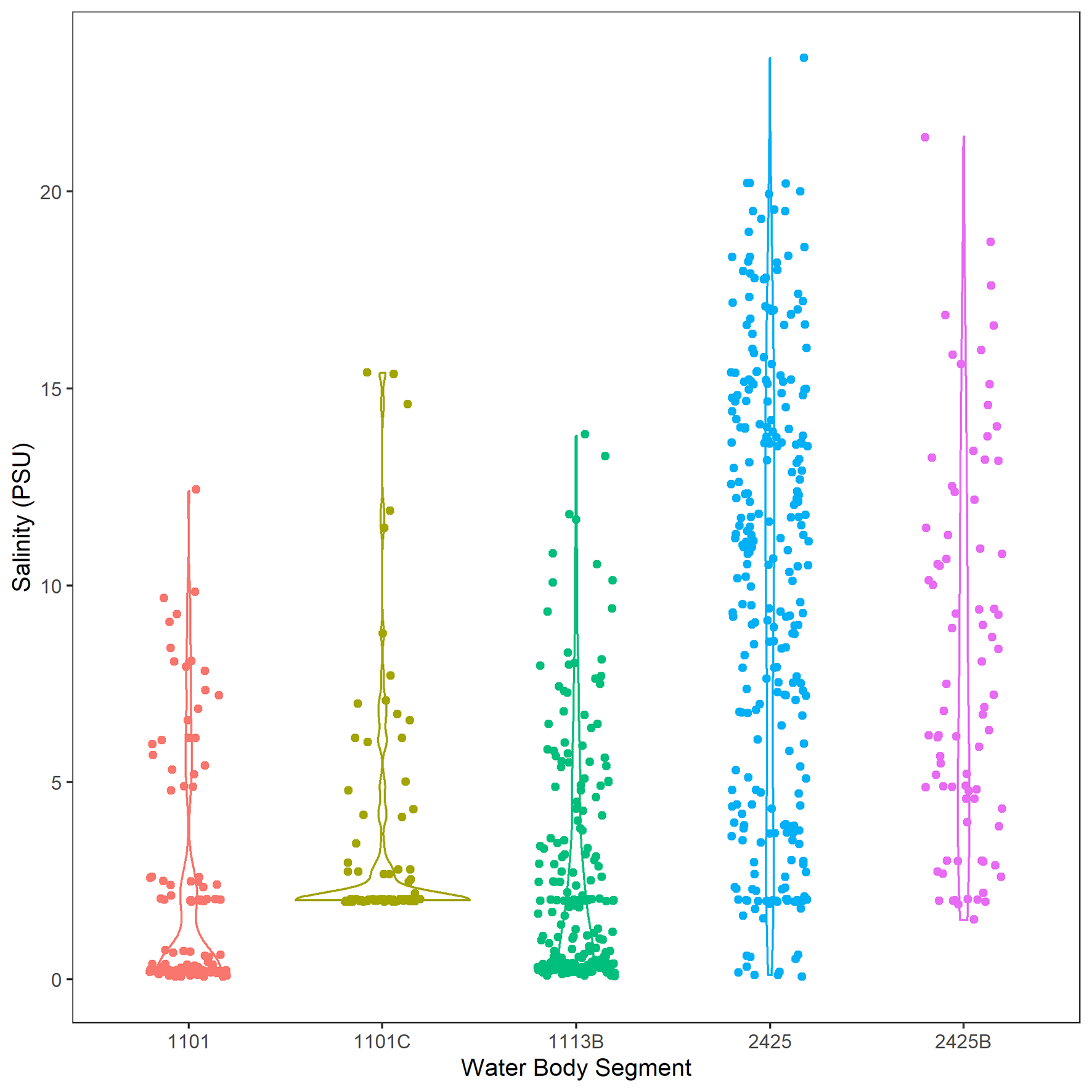

Supplement: Supplementary Figure 2 — Historical salinity data for Clear Lake system. Stations correspond to segments in Figure 1. Data is from 2011 to 2021. Figure was generated with scripts in Supplementary File 2. [file Image_2.TIFF]

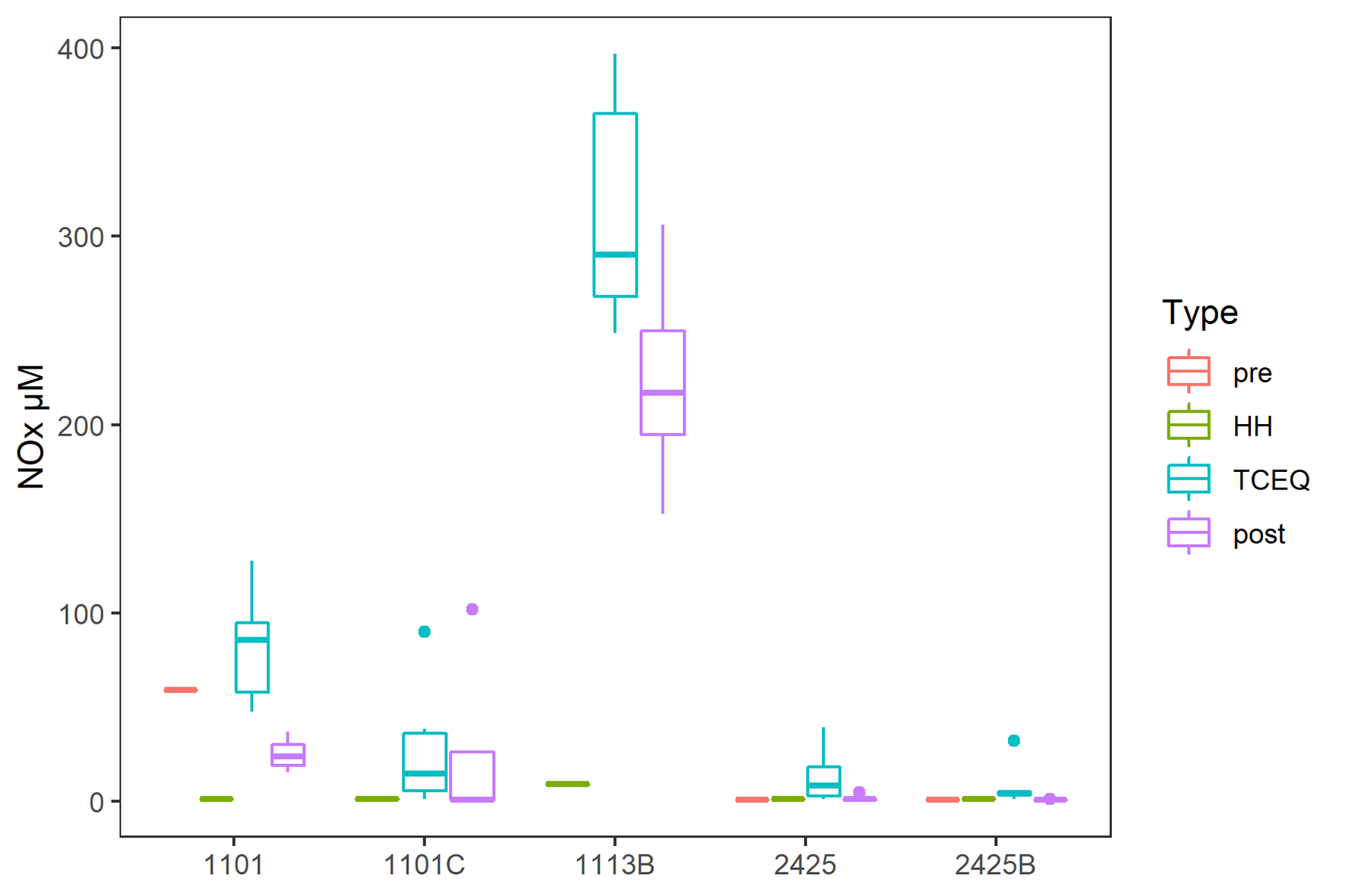

Supplement: Supplementary Figure 3 — Nitrate/nitrite concentration by water body segments and sample type. Type and segment are as Figure 2. Figure was generated with scripts in Supplementary File 3 [file Image_3.TIFF]

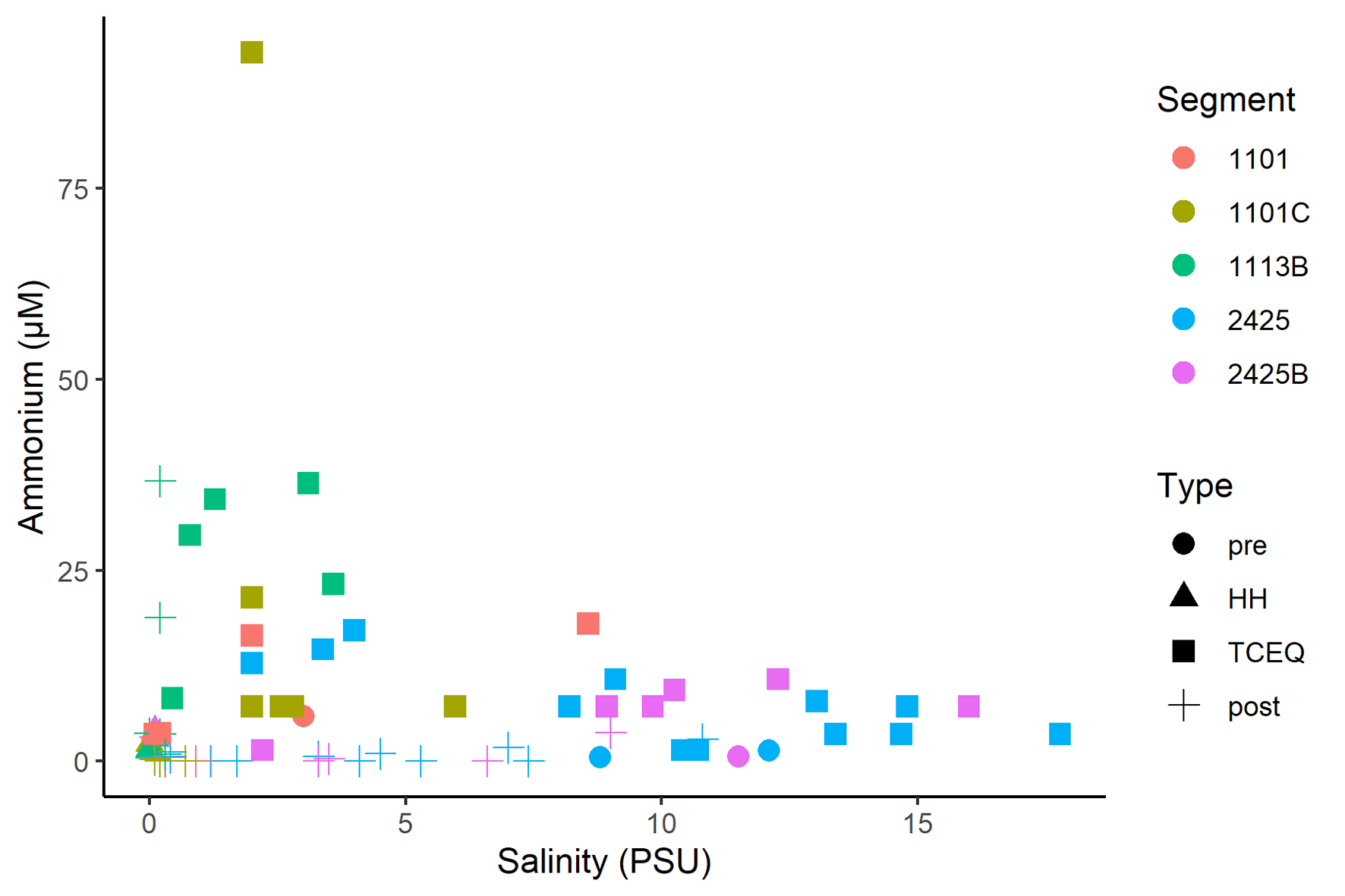

Supplement: Supplementary Figure 4 — Mixing diagram of salinity vs. ammonium for the Clear Lake system. Symbols are as Figure 3. Figure was generated with scripts in Supplementary File 3. [file Image_4.TIFF]

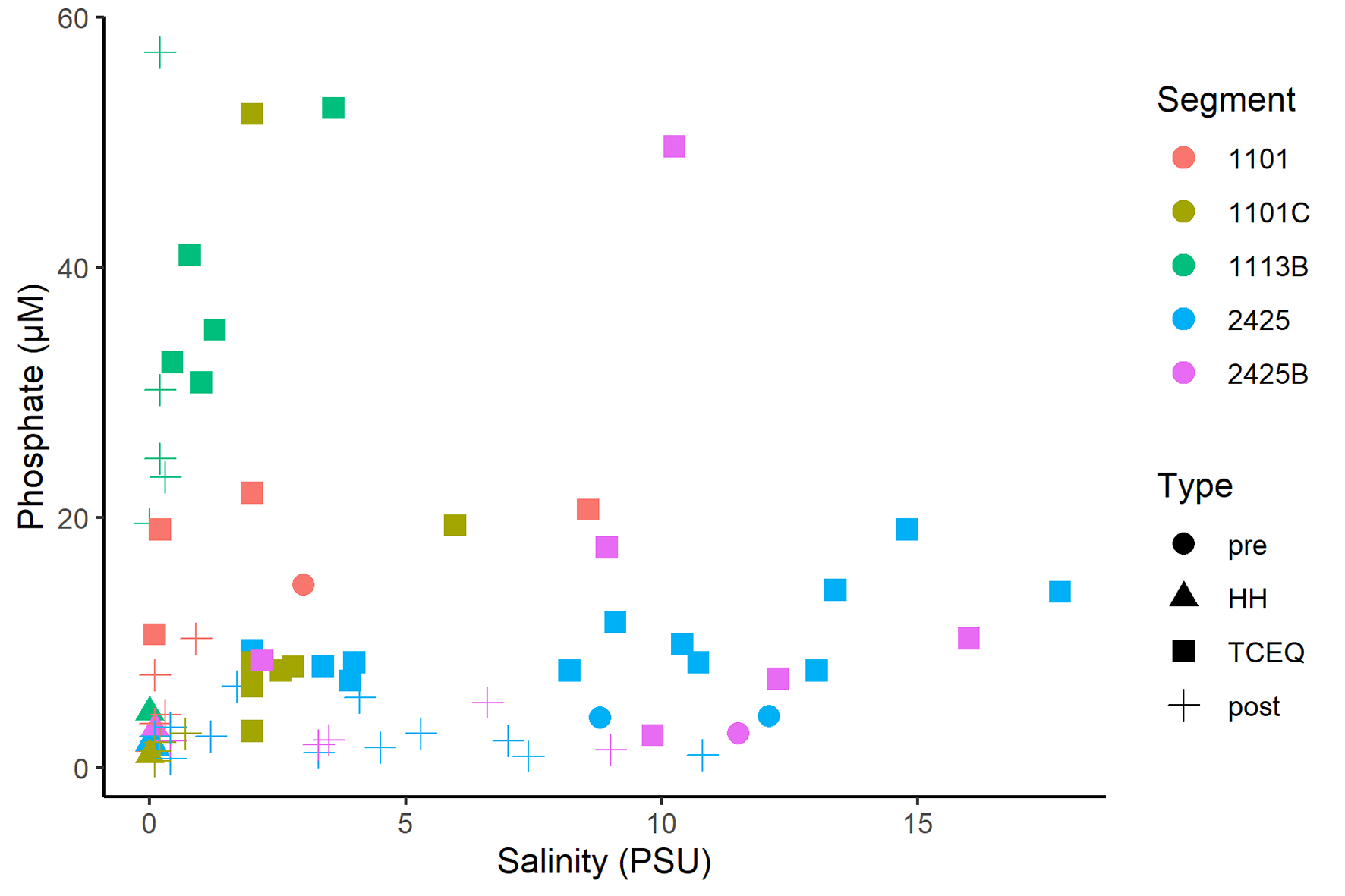

Supplement: Supplementary Figure 5 — Mixing diagram of salinity vs. phosphate for the Clear Lake system. Symbols are as Figure 3. Figure was generated with scripts in Supplementary File 3. [file Image_5.TIFF]

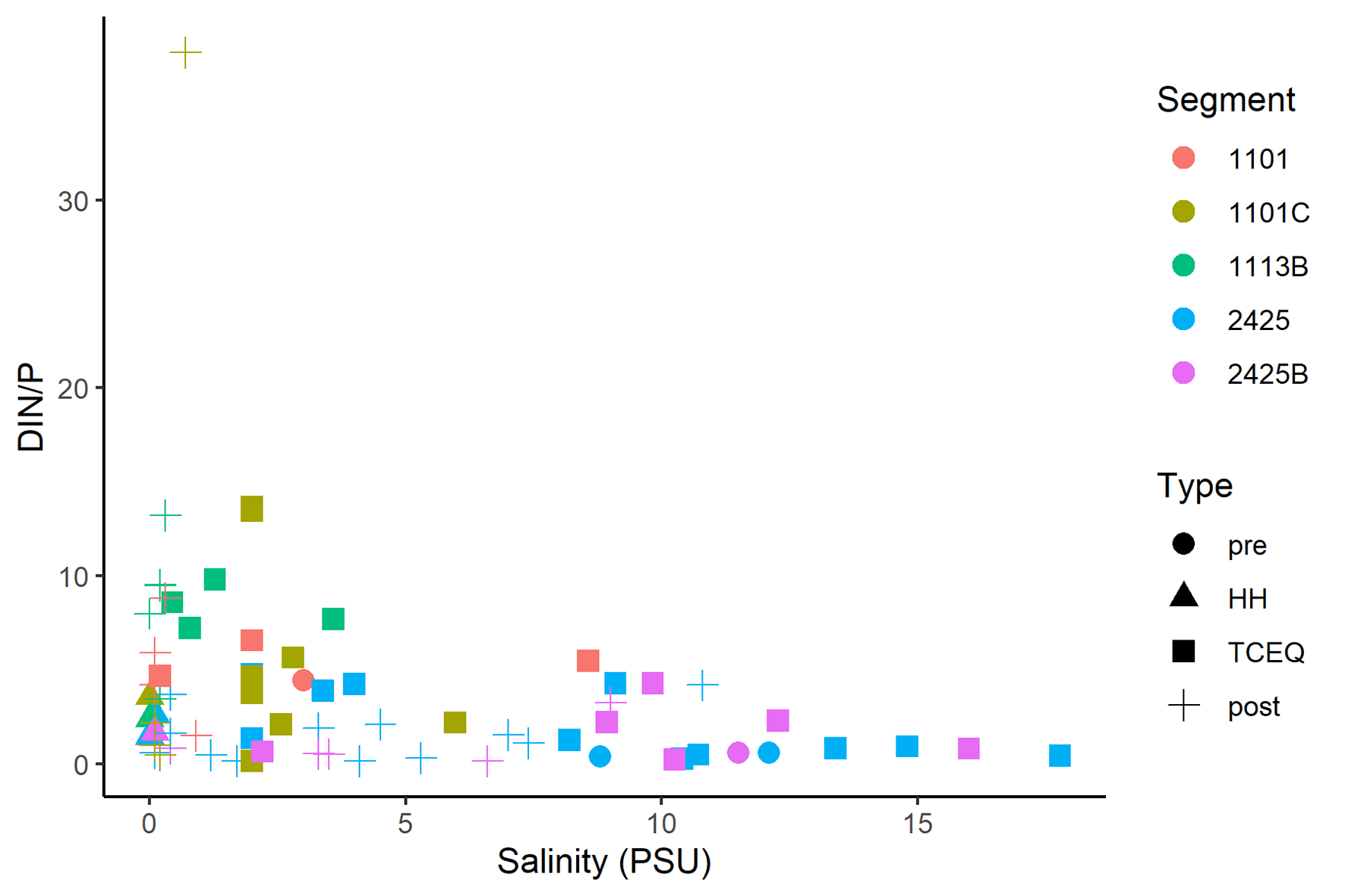

Supplement: Supplementary Figure 6 — Mixing diagram of N/P ratio vs. phosphate for the Clear Lake system. Symbols are as Figure 3. Figure was generated with scripts in Supplementary File 3. [file Image_6.TIFF]

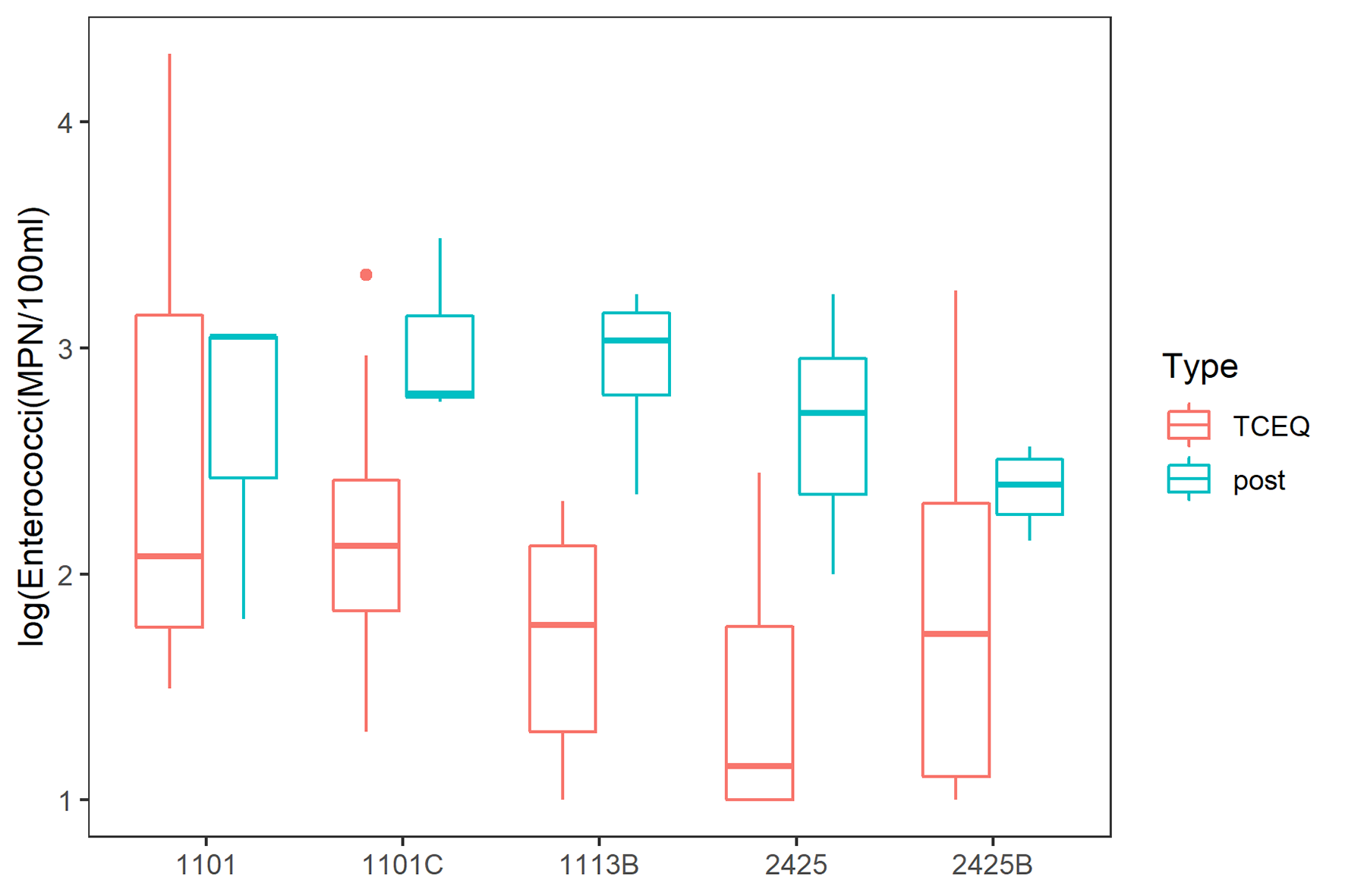

Supplement: Supplementary Figure 7 — Comparison of historical Enterococci concentrations vs. concentrations following Hurricane Harvey. Symbols are as Figure 3. Figure was generated with scripts in Supplementary File 3. [file Image_7.TIFF]

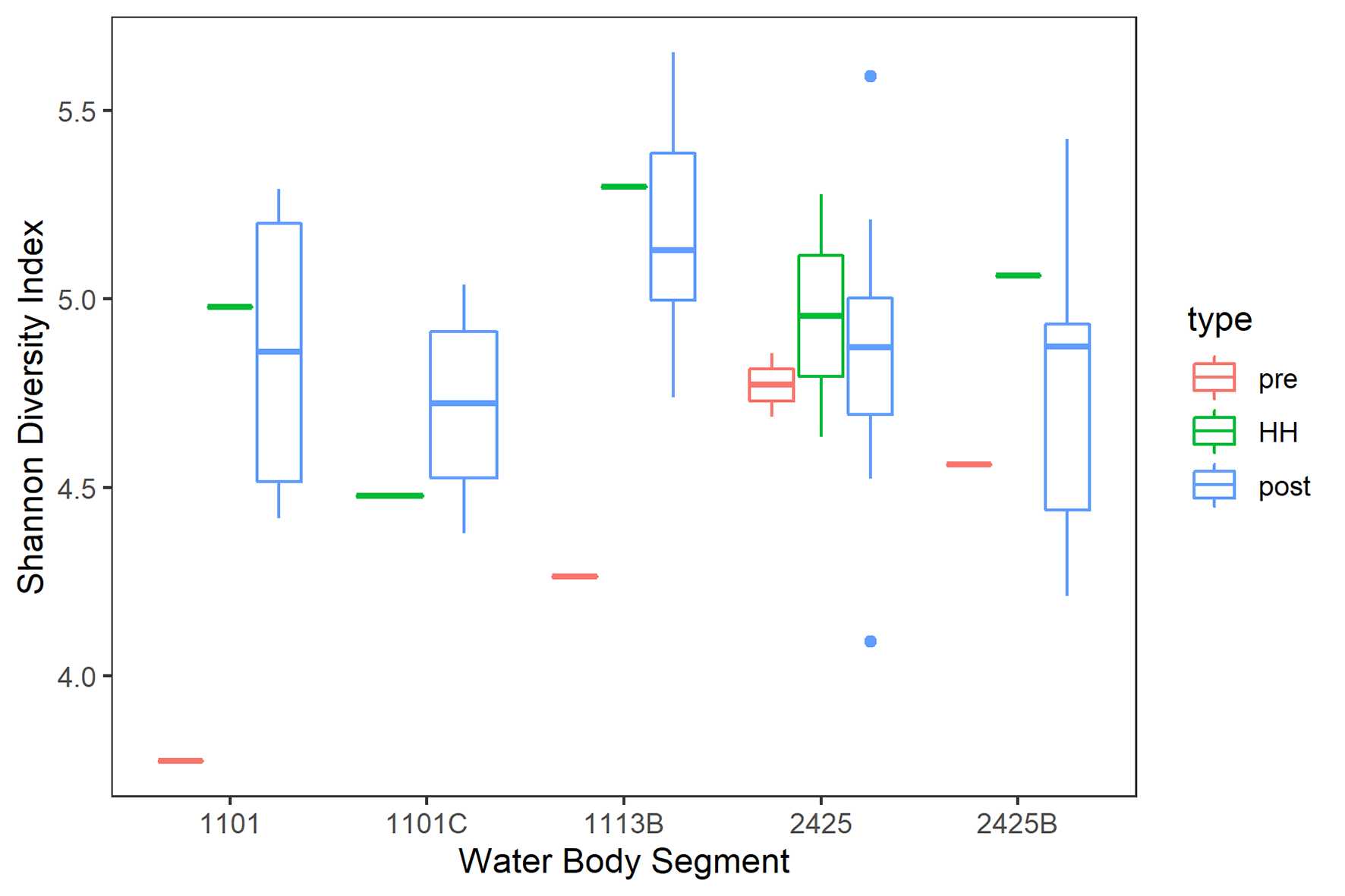

Supplement: Supplementary Figure 8 — Alpha diversity of bacterial communities in the Clear Lake system before and after Hurricane Harvey. Shannon diversity indices were calculated by targeted metagenomic analysis, as described in Methods. Boxes, whiskers and horizontal lines are described in Figure 2. Sample types are described in Figure 3. Figure was generated with scripts in Supplementary File 5. [file Image_8.TIFF]

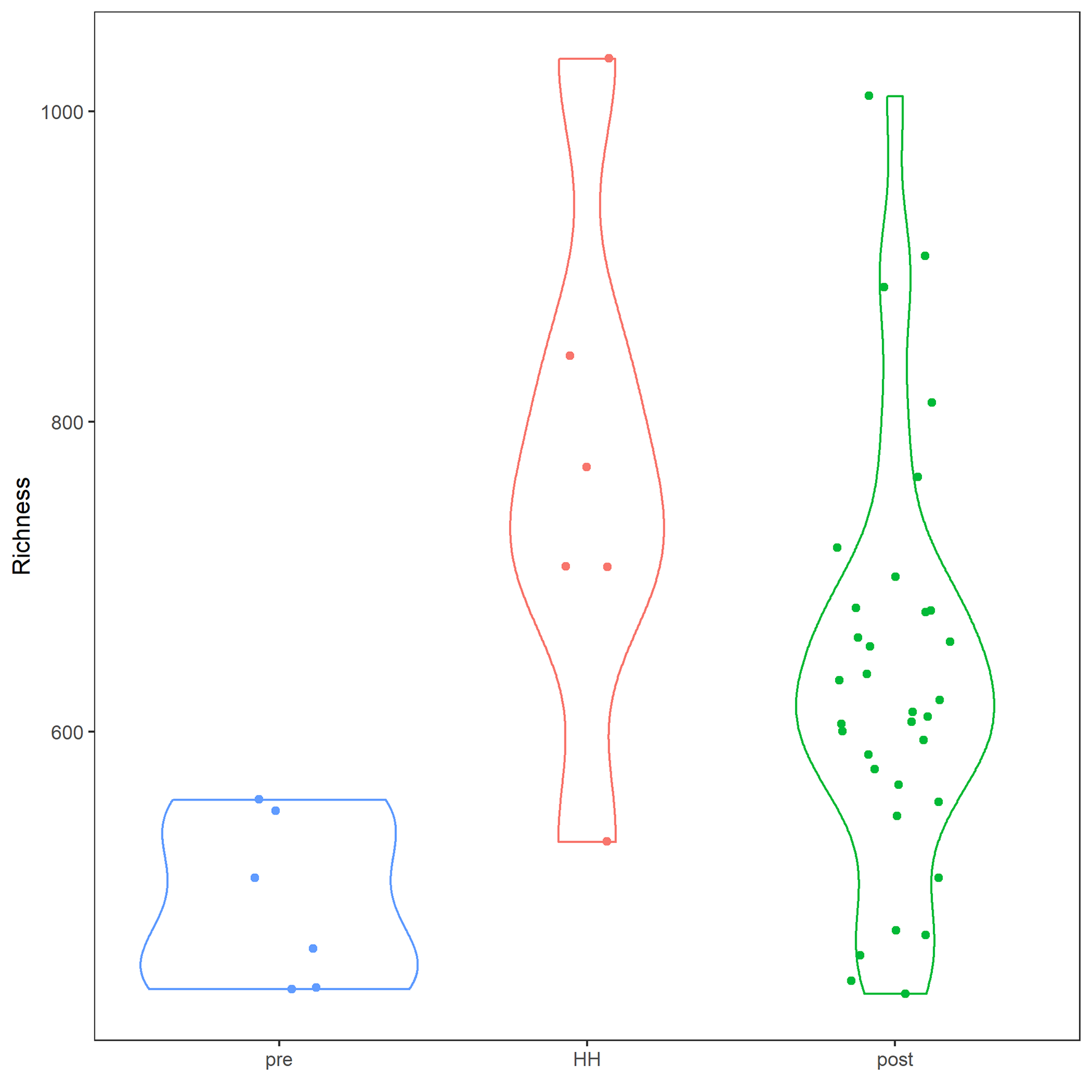

Supplement: Supplementary Figure 9 — Microbial richness in Clear Lake system before and after Hurricane Harvey. X-axis indicates number of amplicon sequence variants. Categories correspond to before (pre), immediately after (HH) and more than a week after (post). Figure was generated with scripts in Supplementary File 5. [file Image_9.TIFF]

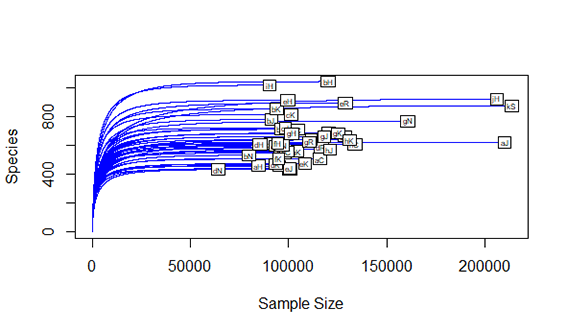

Supplement: Supplementary Figure 10 — Rarefaction analysis of richness by number of reads. Symbols are as Figure 9. Figure was generated with scripts in Supplementary File 5. [file Image_10.TIFF]

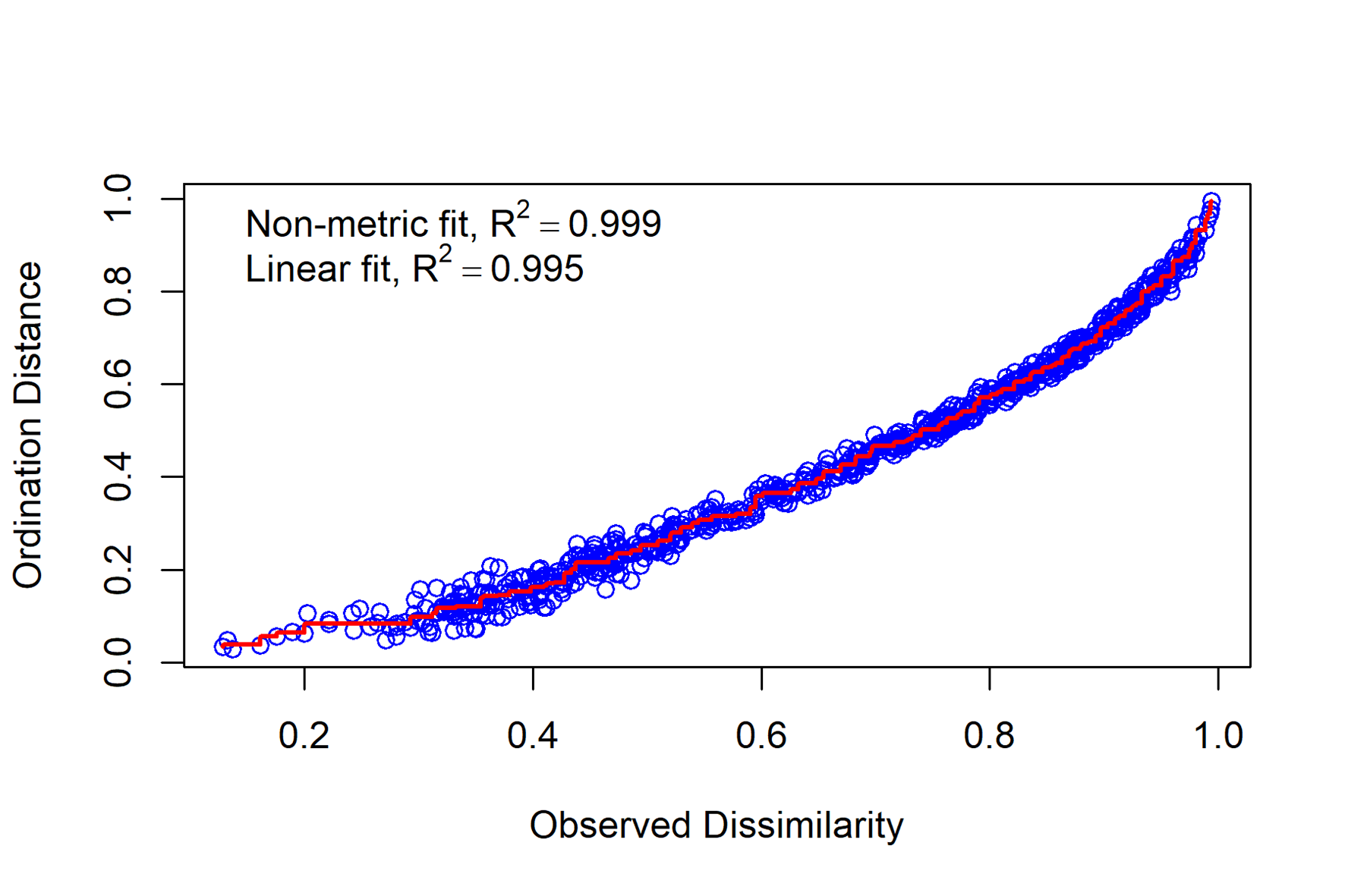

Supplement: Supplementary Figure 11 — Shepards diagram showing fit of NMDS to dissimilarity of any two pairs of samples. Figure was generated with scripts in Supplementary File 8. [file Image_11.TIFF]

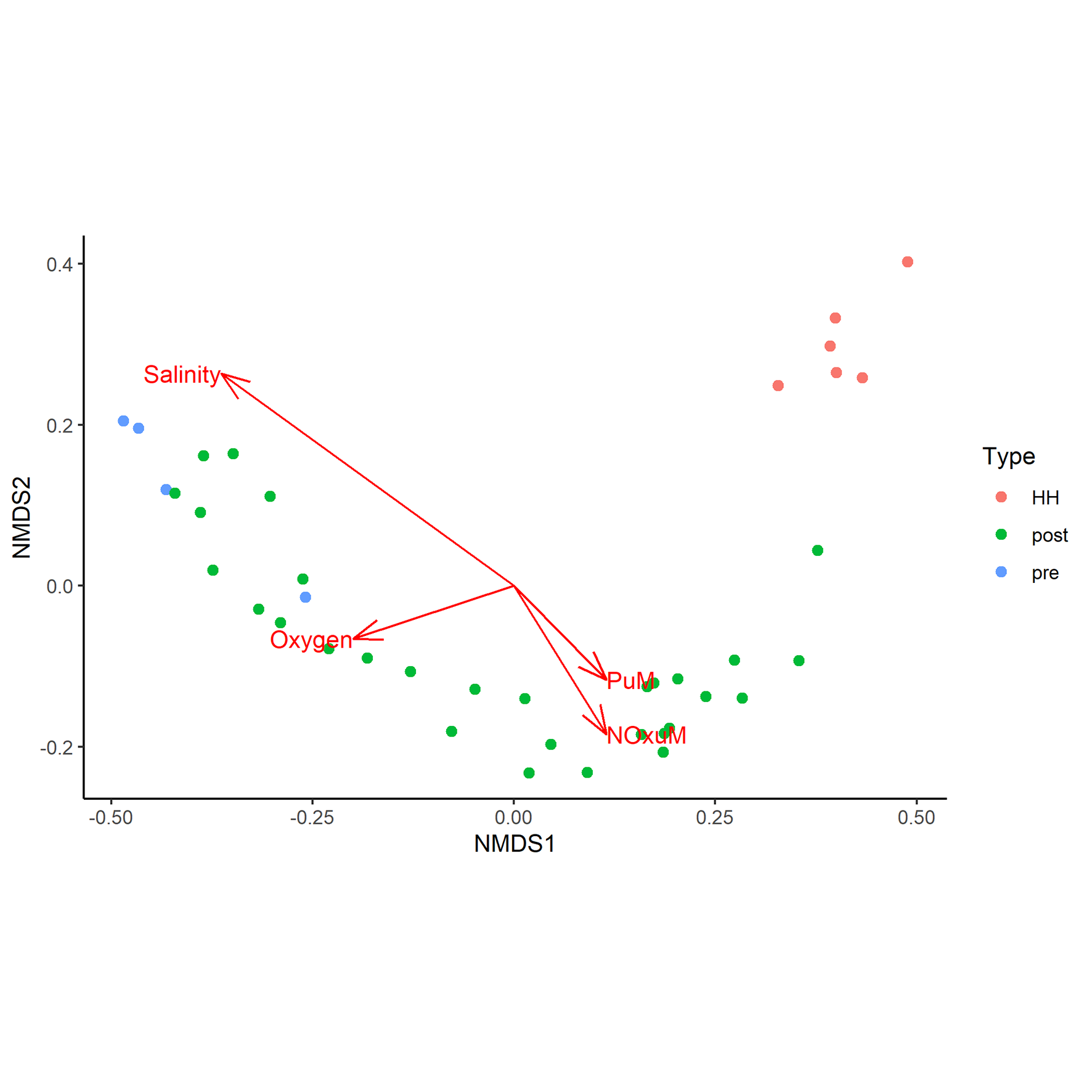

Supplement: Supplementary Figure 12 — Fit of environmental data to NMDS model of microbial community structure. Stress value for model was 0.032, which suggests an excellent fit. Environmental variables that showed a significant (P < 0.10) relationship with community structure are shown. Only samples with DIN data available are shown. Note conductivity and salinity vectors were practically identical to each other and would overlap, so only salinity vector is shown. Figure was generated with scripts in Supplementary File 8. [file Image_12.TIFF]

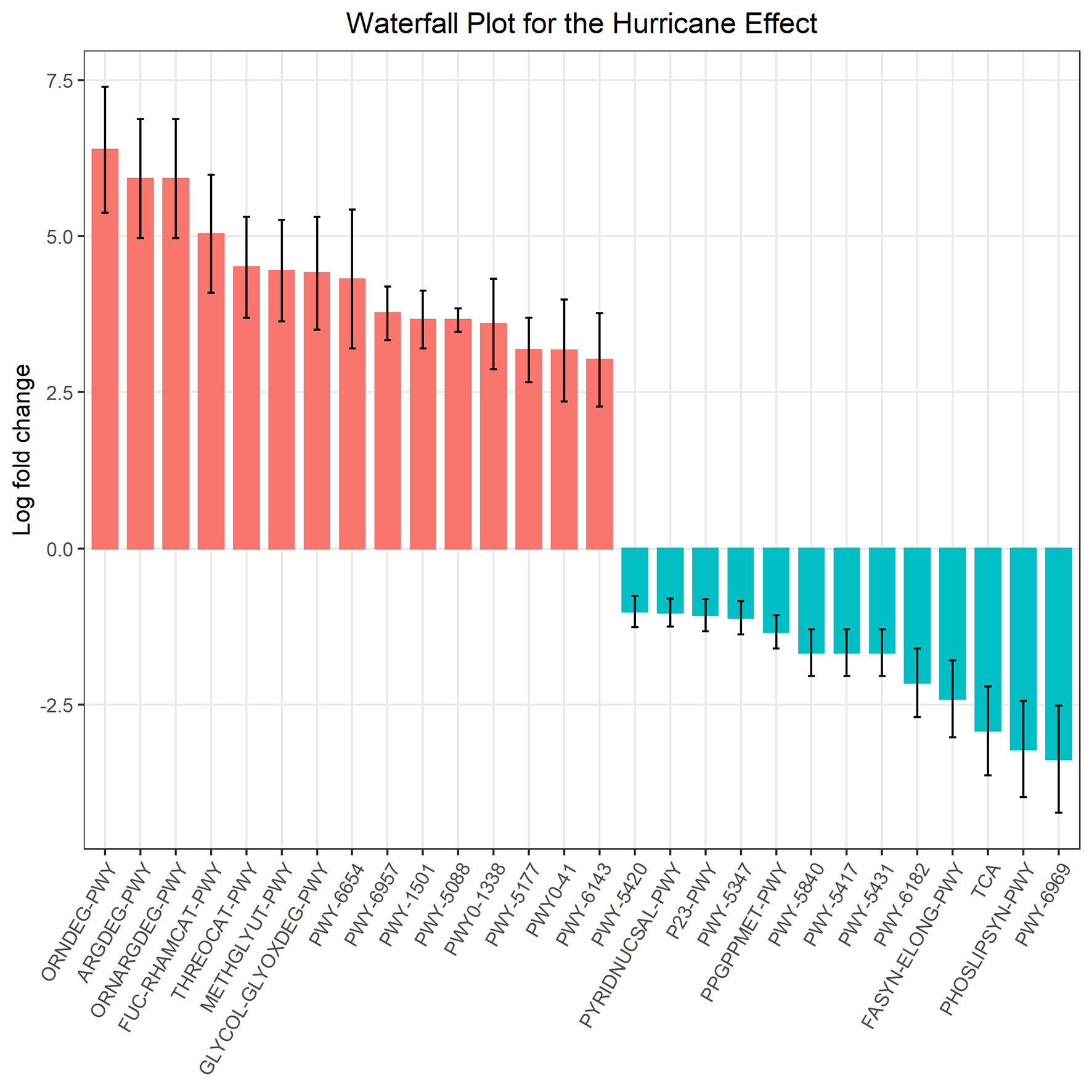

Supplement: Supplementary Figure 13 — Waterfall plot showing pathways enriched (red) or depleted (teal) in comparison of samples collected before or immediately after Hurricane Harvey. Figure was generated with scripts in Supplementary File 15. [file Image_13.TIFF]

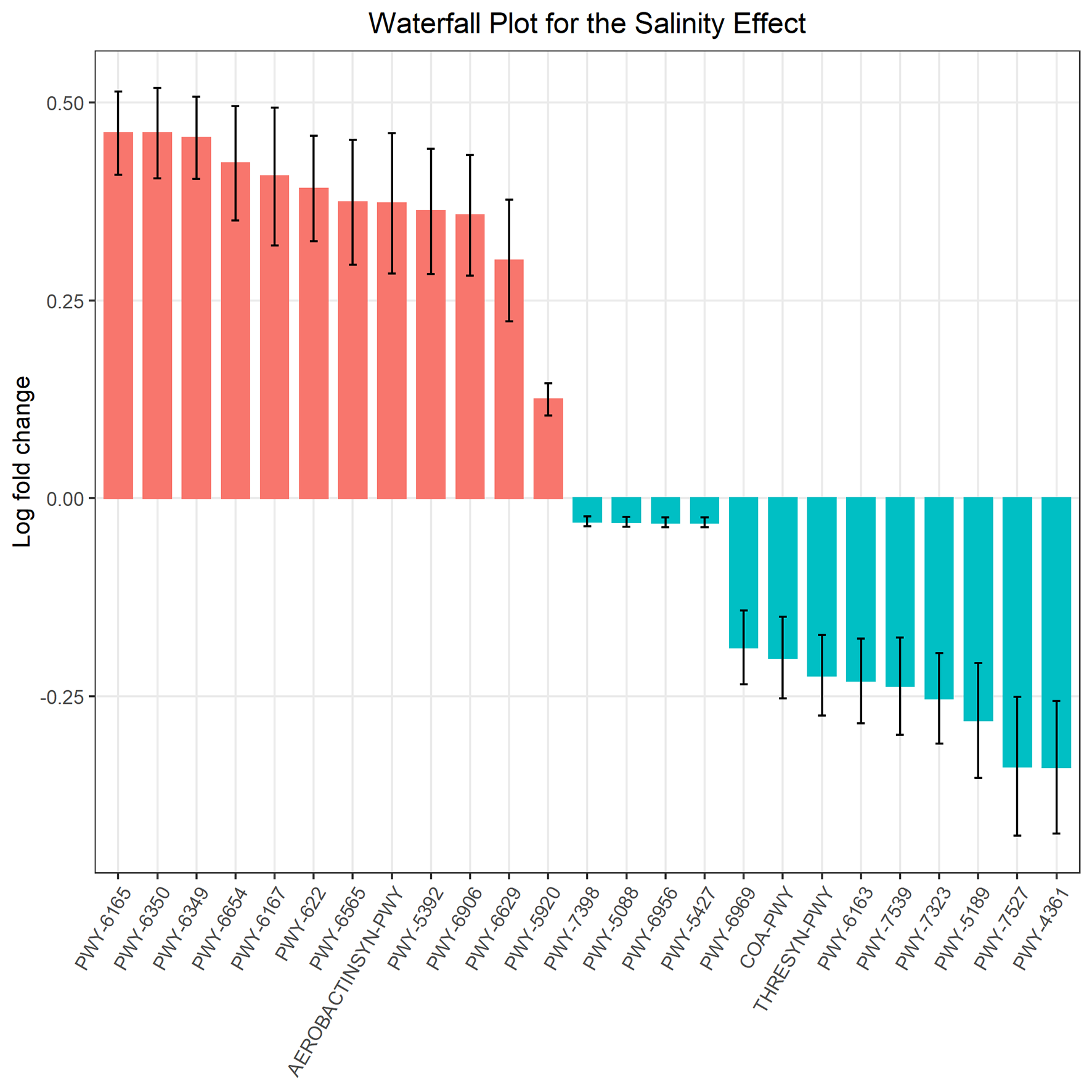

Supplement: Supplementary Figure 14 — Waterfall plot showing pathways enriched (red) or depleted (teal) as function of salinity concentration. Figure was generated with scripts in Supplementary File 15. [file Image_14.TIFF]

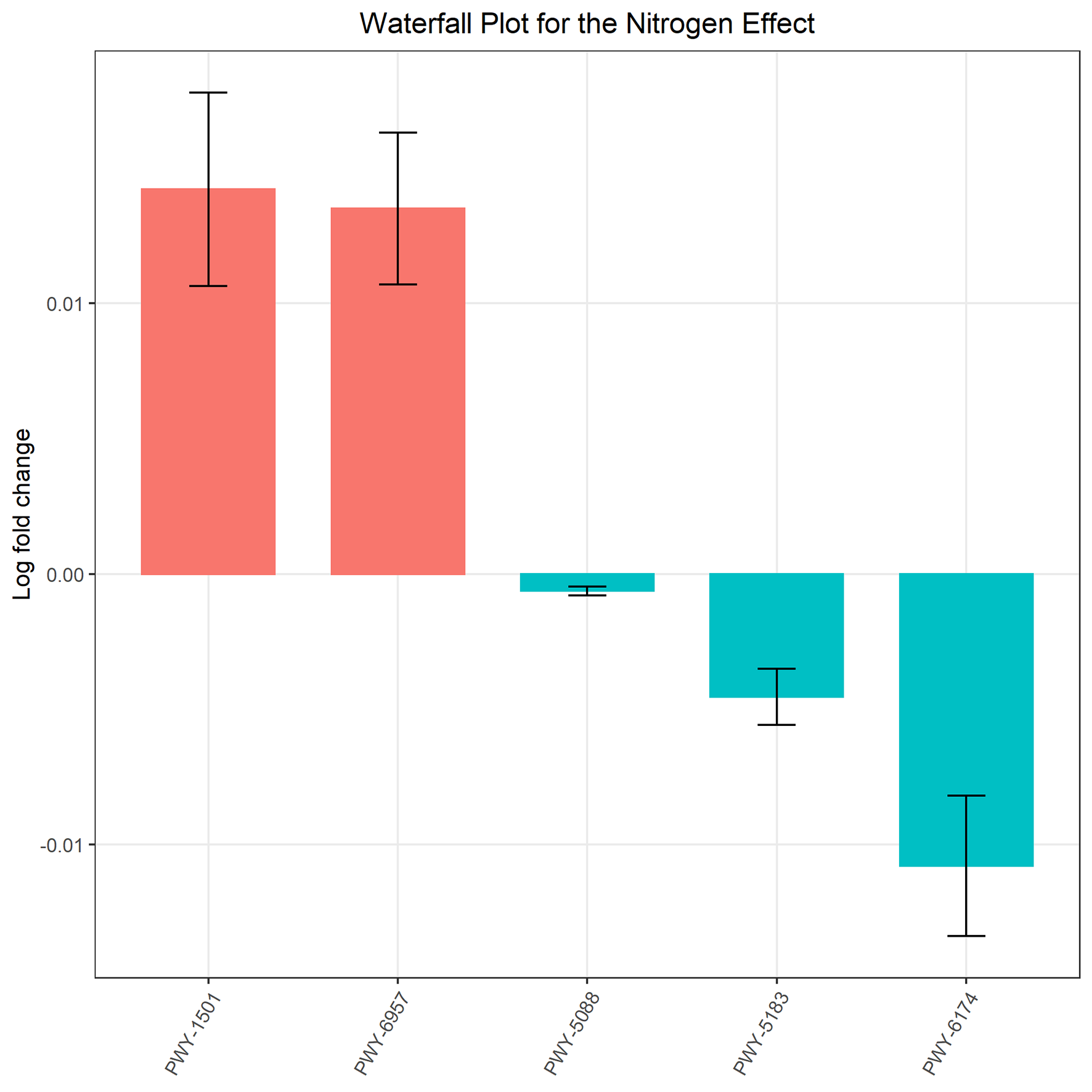

Supplement: Supplementary Figure 15 — Waterfall plot showing pathways enriched (red) or depleted (teal) as function of nitrate concentration. Figure was generated with scripts in Supplementary File 15. [file Image_15.TIFF]
